# Structure–Property Relationships in Novel Series of Photoswitchable Local Anesthetic Ethercaine Derivatives: Emphasis on Biological and Photophysical Properties

**DOI:** 10.3390/ijms26073244

**Published:** 2025-03-31

**Authors:** Alexey Noev, Daria Likhobabina, Janna Sutemieva, Anna Plyutinskaya, Dmitry Cheshkov, Natalia Morozova, Aleksandra Vinokurova, Yuriy Vasil’ev, Nikita Suvorov, Elena Nemtzova, Andrei Pankratov, Elena Filonenko, Petr Shegay, Andrey Kaprin, Mikhail Grin

**Affiliations:** 1 Department of Chemistry and Technology of Biologically Active Compounds, Medicinal and Organic Chemistry, Institute of Fine Chemical Technologies, MIREA-Russian Technological University, 86 Vernadsky Avenue, 119571 Moscow, Russia; syrinda@mail.ru (D.L.); zhanna22052002@mail.ru (J.S.); dcheshkov@gmail.com (D.C.); y_vasiliev@list.ru (Y.V.); suvorov.nv@gmail.com (N.S.); andreimnioi@yandex.ru (A.P.); derkul23@yandex.ru (E.F.); michael_grin@mail.ru (M.G.); 2 P. Hertsen Moscow Oncology Research Institute—Branch of the National Medical Research Radiological Centre of the Ministry of Health of the Russian Federation, 2nd Botkinsky pr. 3, 125284 Moscow, Russia; anna2031@rambler.ru (A.P.); n.b.morozova@yandex.ru (N.M.); nemtz@yandex.ru (E.N.); dr.shegai@mail.ru (P.S.); kaprin@mail.ru (A.K.); 3 State Scientific Research Institute of Chemistry and Technology of Organoelement Compounds, 38 Shosse Entuziastov, 105118 Moscow, Russia; 4 State Budgetary Educational Institution School, 1329 Moscow, Russia; aleksvinokyrova@mail.ru; 5 Department of Operative Surgery and Topographic Anatomy, I.M. Sechenov First Moscow State Medical University (Sechenov University), Trubetskaya St. bldg. 8\2, 119435 Moscow, Russia

**Keywords:** ethercaine, photopharmacology, photoswitchable local anesthetics, azobenzenes, structure–activity relationship, pain

## Abstract

The insufficient selectivity of existing local anesthetics can lead to serious adverse effects. Considering the widespread use of this class of drugs, the development of new local anesthetics that do not cause side effects is an important task. One approach to address this issue is the use of photopharmacology, which enables the creation of agents with light-controlled biological activity. Several examples of azobenzene-based photoswitchable blockers of voltage-gated sodium (Na_v_) channels have been described so far. These compounds can be used as light-controlled local anesthetics, one of which is ethercaine, synthesized by our group earlier. However, systematic studies of the “structure-activity” relationship in the series of light-controlled local anesthetics based on azobenzene are absent in the literature. The aim of this study was to obtain new derivatives of ethercaine and investigate their photophysical and biological properties. A total of 14 new derivatives were synthesized, and their structure was confirmed by various physicochemical analysis methods. The Z-E isomerization half-lifes were determined for all the synthesized compounds. The cytotoxic effect on normal cells was studied in vitro using human dermal fibroblasts (DF2). The local anesthetic activity of all the synthesized compounds was evaluated in vivo on a model of surface anesthesia in both darkness and under UV light irradiation. Based on the results obtained, conclusions were drawn regarding the potential of the proposed substances, and optimal pathways for structural modification were identified.

## 1. Introduction

**Figure 1:**
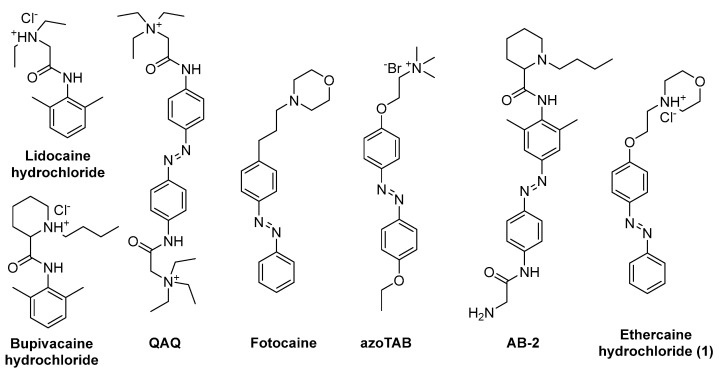
Chemical structures of known photoswitchable Na_v_ channels blockers: QAQ, fotocaine, azoTAB, ethercaine hydrochloride (**1**), and azobupivacaine-2 (AB2).

The clinical use of non-selective local anesthetics can lead to severe adverse effects that pose significant risks to the patient’s life and health [1,2,3]. Accidental entry of local anesthetics into the systemic bloodstream may result in respiratory and cardiac arrest. These concerns highlight the urgent need to develop new drugs whose local anesthetic activity can be externally controlled. The photopharmacological approach, which involves introducing light-sensitive chemical groups into the drug molecular structure to modulate biological activity, provides the means for spatial and temporal control over drug action using light [4,5,6,7,8,9]. When such molecules are irradiated, they undergo photoisomerization (in case of reversible photopharmacological agents) [4,5,6] or light-induced cleavage of sensitive fragments (photodegradable prodrugs), leading to the formation of active compounds [7,8,9]. One of the main classes of photoisomerizable molecules used in photopharmacology includes azobenzenes and their heterocyclic derivatives. The presence of a double bond in the azo group enables the existence of E- and Z-isomers, which can interconvert under light irradiation. The difference in structure between these isomers allows for the design of drugs that interact with biological targets only in one of the two forms. Light-controlled photopharmacological agents based on azobenzene and its heterocyclic derivatives have been shown to interact with various biological targets, such as GPCR receptors, enzymes, etc. [10,11,12,13]. Compounds that act on ion channels and are controlled by light have garnered significant interest [14,15,16]. Previously, our group and others have developed light-controlled blockers of voltage-gated sodium (Na_v_) channels that can be used as local anesthetics and antiarrhythmics (Figure 1) [17,18,19,20,21,22,23,24]. However, systematic studies on the structure–activity relationships among these compounds are still absent in the literature.

All the structures shown in Figure 1 have similar pharmacophoric elements characteristic of local anesthetics: a lipophilic aromatic part, a hydrophilic fragment containing a protonatable nitrogen atom, and a linker connecting the hydrophobic and hydrophilic fragments. Varying the latter or the linker while maintaining the pharmacophore sites may improve the biological properties of the compound, including local anesthetic activity and compound safety.

This study aimed to broaden the understanding of the influence of chemical structure on the biological (local anesthetic activity and cytotoxicity) and photophysical properties within a series of novel derivatives of the light-controlled, local anesthetic ethercaine.

## 2. Results and Discussion

Like all azobenzene-based photopharmacological agents, ethercaine can exist in two (E and Z) forms, of which the *E*-form is thermodynamically more favorable; therefore, this isomer is predominantly present in ethercaine solutions in the dark. When irradiated with ultraviolet light (usually 365 nm), ethercaine quickly transforms into a less stable Z-isomer, which eventually reverts to the *E*-form in the dark or when irradiated with blue light [23]. Since ethercaine exhibits local anesthetic properties in the E-form, which almost completely disappear upon transition to the Z-isomer upon irradiation with UV light, possible methods of its use are the preliminary deactivation of ethercaine with the introduction of an inactive isomer into the body and subsequent activation in the body [23,24,25]. Another possible clinical application is the possibility of rapid deactivation in the body when such a need arises. Thus, the development of new derivatives of ethercaine with more stable Z-isomers makes it possible to increase control over biological activity in the body. In addition, an important parameter is the difference in local anesthetic activity in E- and Z-forms.

Thus, the main biological property investigated in this work was the local anesthetic activity of the new derivatives in the dark (with a predominance of the E-form) and under irradiation (with a predominance of the Z-form). The evaluation of local anesthetic activity was conducted on the rabbit cornea using the Regnier method. The quantitative measure of anesthetic potency in this method is the Regnier index (RI), which ranges from 13 (no anesthesia within 60 min after administration) to 1300 (complete anesthesia for 60 min) [23,24,25]. RI values in darkness (451 ± 40) and under UV light irradiation (469 ± 37) for a 2% solution of lidocaine, used as a reference drug, were obtained previously and are independent of light exposure [23]. A 4% aqueous solution of Kolliphor^®^ ELP, used for solubilizing ethercaine and its derivatives and lacking intrinsic local anesthetic activity, was employed as a negative control [25]. In vitro cytotoxicity studies were conducted to initially assess the safety of the compounds.

As one of the main physicochemical properties of photopharmacological agents, the Z-E half-life was investigated, which determines the stability of the Z-isomer. The Z-E half-lifes were measured using UV/Vis spectrophotometry, converting the compound to the Z-isomer by irradiating with UV light and observing the change in absorption intensity due to reverse Z-E relaxation.

**Figure 2:**
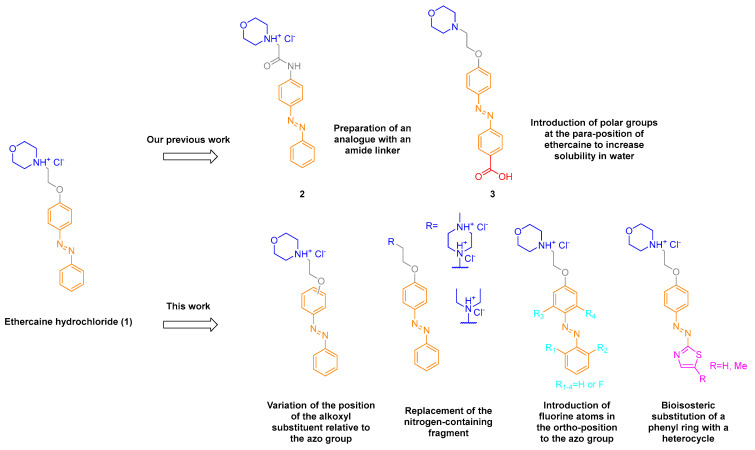
Main directions of the ethercaine modification in previous and current studies. The colors indicate the key elements of the structures. The blue color indicates hydrophilic fragments containing tertiary or quaternary nitrogen atoms. The gray color indicates the linker. The lipophilic part is indicated in orange. New structural elements are shown in cyan and magenta.

Previously, to investigate the influence of linker nature on biological activity, we synthesized an ethercaine analog with an amide linker (**2**) and a para-carboxy derivative of ethercaine (**3**) to enhance water solubility (Figure 2) [24]. On a surface anesthesia model, it was demonstrated that the local anesthetic activity of the amide derivative **2** was lower compared to ethercaine. Therefore, in this current study, ethercaine (**1**), containing an ether-based linker, was chosen as the starting point.

The following structural modifications were chosen: altering the linker position, varying the nitrogen-containing fragment, introducing fluorine atoms in the ortho-positions of the azobenzene, and synthesizing ethercaine derivatives based on a thiazole scaffold (Figure 2).

### 2.1. Influence of the Substituent Position in Azobenzene on the Physicochemical and Biological Properties of Ethercaine Derivatives

All the examples of light-controlled Na_v_ channel blockers shown in Figure 1 contain substituents in the para-positions of the azobenzene core. To investigate the influence of a substituent position on physicochemical and biological properties, we synthesized regioisomers of ethercaine containing a 2-(*N*-morpholino)-ethoxy substituent in various positions of the benzene ring. The synthesis of the hydrochloride of para-substituted ethercaine (**1**) has been previously described and involves classical reactions of diazotization, azo coupling, subsequent alkylation, and the formation of the corresponding hydrochloride [23,25].

**Scheme 1:**
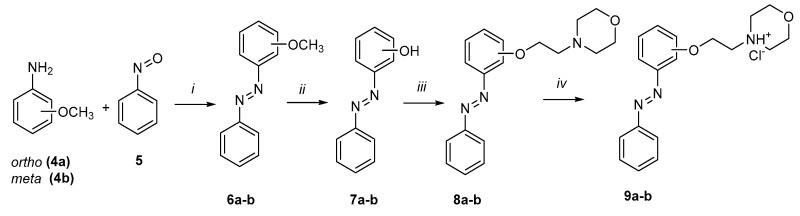
Reagents and conditions: (i) acetic acid, MeOH, RT, 72 h; (ii) BBr_3_, DCM, −78 °C, then RT, 16 h; (iii) *N*-(2-chloroethyl)-morpholine hydrochloride, ACN, K_2_CO_3_, KI, reflux, 6 h; (iv) HCl/Et_2_O, Et_2_O, 0–5 °C, then RT, 30 min.

The hydrochlorides of the ortho- and meta-isomers of ethercaine (**9a**–**b**) were synthesized via the corresponding hydroxyazobenzenes (**7a**–**b**) (Scheme 1). Attempts to obtain the latter through the direct reaction of nitrosobenzene (**5**) with the corresponding aminophenols resulted in trace amounts of the target compounds and the formation of byproducts. Therefore, 2- and 3-methoxyazobenzenes (**6a**–**b**) were first synthesized using Baeyer–Mills reaction and subsequently demethylated using BBr_3_.

The results of studies on Z-E isomerization half-lifes, in vitro cytotoxicity, and in vivo local anesthetic activity are presented in Table 1.

Among the regioisomers of ethercaine, local anesthetic activity in darkness (with *E*-form predominance) increases in the order ortho < meta < para, with RI values of 390 ± 39, 503 ± 85, and 644 ± 30, respectively. Under UV light irradiation (365 nm), the RI for derivatives **9a**–**b** decreased by 5–8 times, indicating a photoswitchable local anesthetic effect. The para-substituted ethercaine **1** exhibited a 19-fold decrease in RI under UV light. Thus, the more elongated derivatives appear to have greater local anesthetic activity compared to the more curved Z-isomers and the ortho-derivative.

Cytotoxicity toward normal cells was evaluated using human dermal fibroblasts (DF2). For compounds **1** and **9b**, cytotoxicity was comparable, with IC_50_ values of 0.21 mM and 0.24 mM, respectively. Compound **2** displayed low cytotoxicity (IC_50_ > 0.2 mM) but showed poor solubility in aqueous solutions at concentrations above 200 µM. Compound **9a** exhibited no cytotoxicity within the tested concentration range (IC_50_ > 1 mM).

The Z-E isomerization half-lifes (τ_1_/_2_) for the meta- and ortho-derivatives **9a** and **9b** were significantly longer than those of the parent para-derivative **1**. Furthermore, τ_1_/_2_ in an aqueous 4% Kolliphor ELP solution exceeded the corresponding values in DMSO. This may be due to the asymmetric distribution of electron density in the molecule or to the spatial stabilization of the Z-form by the π-cationic interaction of the protonated nitrogen atom in morpholine with the lower phenyl ring of azobenzene. The latter is supported by a significant increase in the Z-E half-life in an aqueous solution (a protic solvent in which nitrogen protonation is possible).

### 2.2. Influence of the Nitrogen-Containing Fragment in the Ethercaine Structure on Physicochemical and Biological Properties

**Scheme 2:**
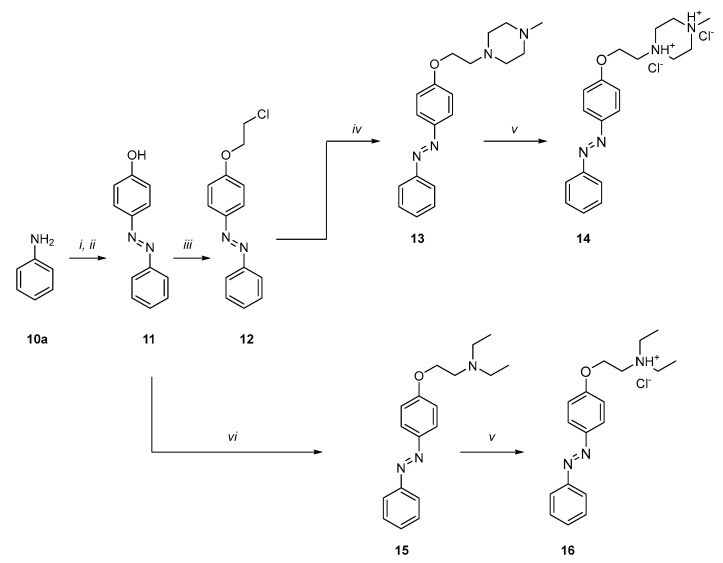
Reagents and conditions: (i) HCl, NaNO_2_, H_2_O, 0–5 °C, 30 min; (ii) phenol, KOH/H_2_O, 0–5 °C, 1 h; (iii) 1-bromo-2-chloroethane, K_2_CO_3_, ACN, reflux, 24 h; (iv) *N*-methylpiperazine, KI, K_2_CO_3_, ACN, reflux, 24 h; (v) HCl/Et_2_O, Et_2_O, 0–5 °C, then RT, 30 min; (vi) *N*-(2-chloroethyl)-*N*,*N*-diethylamine hydrochloride, K_2_CO_3_, KI, acetonitrile, reflux, 3 h.

One of the key fragments in the structure of local anesthetics is the tertiary or quaternary nitrogen atom, which is responsible for binding to the amino acid residues of the pore domain in Na_v_ channels. Derivatives containing *N*-methylpiperazine (**14**) and *N*,*N*-diethylamine (**16**) fragments were synthesized (Scheme 2). The synthesis of compound **13** was performed via a precursor **12** using sequential alkylation reactions followed by treatment with HCl/Et_2_O. The *N*,*N*-diethylamino derivative **16** was obtained through the alkylation of 4-hydroxyazobenzene with *N*-(2-chloroethyl)-*N*,*N*-diethylamine hydrochloride followed by treatment with HCl/Et_2_O.

The results of the study on Z-E isomerization half-lifes, in vitro cytotoxicity, and in vivo local anesthetic activity of compounds **14** and **16** are presented in Table 2.

Compound **14** exhibits similar Regnier index values both under irradiation and in the absence of it, which limits their use as photoswitchable agents. On the other hand, this feature can be used in the future as a starting point for the development of compounds that have local anesthetic activity only in the Z-form. The activity of compound **16**, similar to ethercaine, is present in the dark and decreases upon irradiation with UV light. However, the RI value is decreased in the dark (470 ± 15) and increased upon irradiation (168 ± 73) compared to the values for ethercaine (1) (644 ± 30 and 33 ± 15, respectively), which makes compound **16** less promising for use as a light-controlled local anesthetic. Additionally, compounds **14** and **16** showed increased cytotoxicity compared to the parent ethercaine **1**. An interesting feature is the increased Z-E half-life for compounds **14** and **16** relative to ethercaine **1** (Table 2). Since the changes in structure do not affect the azobenzene fragment and linker, the changes in the stability of the Z-isomer are probably due to spatial effects, such as the π-cationic interaction of the protonated nitrogen and one of the phenyl rings of azobenzene.

### 2.3. Fluorine-Containing Ethercaine Derivatives

**Scheme 3:**
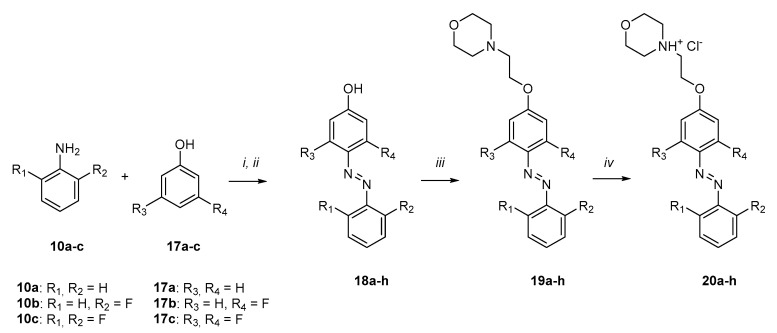
Reagents and conditions: (i) HCl, NaNO_2_, H_2_O, 0–5 °C, 30 min; (ii) **17a**–**c**, KOH/H_2_O, 0–5 °C, 1 h; (iii) *N*-(2-chloroethyl)-morpholine hydrochloride, ACN, K_2_CO_3_, KI, reflux, 6 h; (iv) HCl/Et_2_O, Et_2_O, 0–5 °C, then RT, 30 min.

An important requirement for photoswitchable agents based on azobenzene is the Z-E isomerization half-life (τ_1_/_2_). Compounds with long τ_1_/_2_ can be considered as agents with high spatiotemporal control. Previously, Bléger and colleagues demonstrated the possibility of increasing the Z-E isomerization half-lifes by introducing fluorine atoms in the ortho-positions relative to the azo group [26,27]. We synthesized ortho–fluoro derivatives of ethercaine **20a**–**h** with different numbers and positions of fluorine atoms in azobenzene. The synthesis was carried out using diazotization reactions of the starting anilines **10a**–**c** and azo coupling with the corresponding phenols **17a**–**c**, followed by alkylation and hydrochloride formation (Scheme 3).

The results of the study on the Z-E isomerization half-lifes, in vitro cytotoxicity, and in vivo local anesthetic activity of compounds **20a**–**h** are presented in Table 3.

All the studied ortho–fluoro-containing ethercaine derivatives **20a**–**h** exhibited photoswitchable, local anesthetic activity in vivo. However, the Regnier index for these compounds in the dark was lower than that for the unsubstituted ethercaine, while the RI decreased as the number of fluorine atoms increased. However, the introduction of fluorine atoms into the upper phenyl ring of ethercaine resulted in a more pronounced increase in the Z-E half-life relative to similar derivatives substituted at the lower phenyl ring. This may indicate a lower tolerance to the introduction of substituents into the lower phenyl ring of the azobenzene fragment. The cytotoxic activity against human dermal fibroblasts DF2 for compounds **20a**–**g** remained similar to that of ethercaine but was lower for the tetraortho–fluorine-substituted derivative **20h**, which could be explained by its increased stability to azo bond cleavage.

The obtained data suggest that ortho-fluoro derivatives of ethercaine can be considered as promising agents in cases where a long-lived Z-isomer is required. Moreover, to achieve high values of Z-E half-life while maintaining satisfactory local anesthetic activity, it is sufficient to introduce two fluorine atoms into positions 2 and 6 of the azobenzene fragment of ethercaine.

### 2.4. Thiazole-Containing Derivatives of Ethercaine

Heterocyclic compounds are widely used in drug design and constitutes the structure of many clinically approved drugs [28,29,30]. A modern approach in the development of new photoswitchable compounds is the substitution of one or two phenyl rings of azobenzene with heterocycles. This strategy helps to overcome the limitations of azobenzene derivatives, including low water solubility and metabolic stability. Among the known heterocyclic photoswitchable compounds, phenylazothiazole derivatives are particularly noteworthy [31,32]. The bioisosteric replacement of the phenyl ring with a thiazole ring is justified in terms of increasing water solubility and reducing the risk of forming toxic aniline as a metabolite of ethercaine when the azo bond is cleaved in the body.

**Scheme 4:**
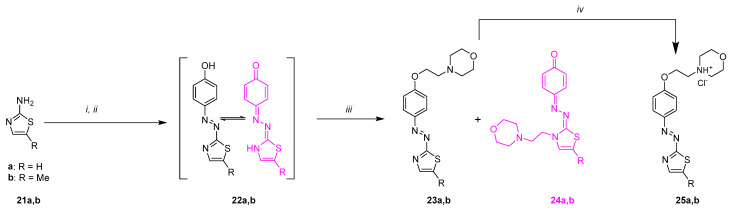
Reagents and conditions: (i) HCl, NaNO_2_, H_2_O, 0–5 °C, 30 min; (ii) phenol, KOH/H_2_O, 0–5 °C, 1 h; (iii) *N*-(2-chloroethyl)-morpholine hydrochloride, DMF, K_2_CO_3_, KI, reflux, 6 h; (iv) HCl/Et_2_O, Et_2_O, 0–5 °C, then RT, 30 min. Structures of by-products are shown in purple.

It is known that introducing a methyl group at position 5 of 2-aminothiazole can reduce the formation of toxic metabolites [33]. Therefore, in addition to the ethercaine derivative based on 2-aminothiazole (**25a**), a compound **25b** was synthesized, which contains a methyl group at position 5 of the thiazole ring (Scheme 4). The 4-hydroxy derivatives **22a**–**b** were obtained by diazotization of 2-aminothiazoles **21a**–**b**, followed by the azo-coupling reaction of the obtained diazonium salts with phenol in an alkaline medium.

**Figure 3:**
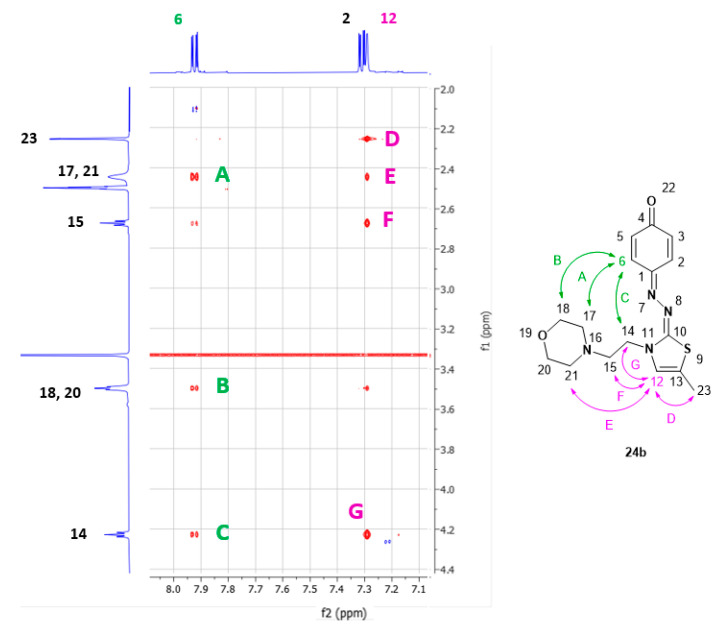
Fragment of the ^1^H,^1^H-NOESY NMR spectrum of compound **24b**. Each signal is marked with a capital Latin letter. The signals associated with 6-H are indicated in green, with 12-H–in purple.

When synthesizing 4-hydroxyphenylazothiazoles **22a**–**b**, a mixture of two compounds was observed by TLC in the product, which could not be separated by column chromatography. As a result of their alkylation, isomers **23a** and **24a** with the same molecular weight were obtained and isolated. Tautomerism was suggested for compound **22a**, leading to the formation of *O*- and *N*-alkylation products. Similarly, the reaction proceeded with 2-(4-hydroxyphenylazo)-5-methylthiazole **22b**, resulting in the alkylation of products **23b** and **24b**. The proposed structures of the compounds are shown in Scheme 4. A similar observation had previously been made for phenylazobenzothiazole derivatives [34]. In our work, compounds **23a**–**b** and **24a**–**b** were studied using mass spectrometry and ^1^H, ^13^C{^1^H}, ^1^H,^1^H-NOESY and ^1^H,^13^C-HMBC NMR spectroscopy (Figures S119–S140, Supplementary Data). Additionally, ^1^H-^15^N HMBC spectra were recorded for compounds **23b** and **24b** (Figures S128 and S139, Supplementary Data). Figure 3 shows a fragment of the ^1^H,^1^H-NOESY NMR spectrum of compound **24b**, where the observed correlation peaks indicate spatial proximity of the methylene protons of the linker and the morpholine ring with the proton of the six-membered ring (cross-peaks A–C) and with the proton at position 4 of the thiazole (cross-peaks D–G).

In the ^1^H,^13^C-HMBC spectrum of compound **24b**, cross-peaks are observed between the 14-CH_2_ protons of the methylene linker and the carbon atoms 12-C and 10-C of the five-membered heterocycle (Figure S138, Supplementary Data). In the ^1^H, ^15^N-HMBC spectrum of compound **24b**, cross-peaks appear between the signals of the 14-CH_2_ and 15-CH_2_ protons of the methylene linker and the 11-N nitrogen atom of the heterocycle (Figure S139, Supplementary Data).

It should also be noted the significant difference in the ^15^N chemical shift values of the atom 11-N in the heterocycle in compounds **23b** (329 ppm) and **24b** (156 ppm). The obtained data confirm the structure of compounds **23a**–**b** and **24a**–**b**.

Since compounds **24a**–**b** do not possess the photoisomerization ability, biological studies were conducted only for compounds **25a**–**b**, obtained from compounds **23a**–**b** by treatment with HCl/Et_2_O. The results of these studies are presented in Table 4.

Compound **25a** exhibits good water solubility (>100 mg/mL) and shows light-controlled, local anesthetic effects. However, the latter is reduced by half compared to the baseline ethercaine without irradiation (644 ± 30 and 342 ± 21, respectively), which may be explained by its low lipophilicity. The introduction of a methyl group at position 5 of the thiazole ring results in an increased local anesthetic effect and reduced cytotoxicity towards DF2 fibroblasts, but significantly decreases water solubility compared to the unsubstituted thiazole derivative **25a**. Furthermore, compounds **25a** and **25b** exhibit short Z-*E* half-lifes (12 min and 5 min in DMSO, respectively).

## 3. Materials and Methods

### 3.1. Materials

All the chemicals were obtained from commercial sources and used without further purification unless specified otherwise. For micellar solutions preparation, Kolliphor^®^ ELP was used (BASF, Ludwigshafen, Germany). Silica gel 60 (Merck KGaA, Darmstadt, Germany) was used for column chromatography. Analytical TLC was performed on Kieselgel 60 F245 silica gel aluminum plates (Merck KGaA, Darmstadt, Germany).

Electronic absorption spectra were obtained using a Shimadzu UV1800 UV/VIS spectrophotometer (Shimadzu Corporation, Kyoto, Japan) in a 10 mm thick quartz cell. NMR spectra were obtained on a Bruker DPX300 spectrometer (Bruker Corporation, Billerica, MA, USA) using DMSO-d6, acetone-d6, and CDCl_3_ as solvents. Residual solvents were used as the reference standard for spectra calibration. Coupling constants, when given, are reported in hertz (Hz).

Non-anesthetized, mature male rabbits of a Soviet chinchilla breed weighing 2–3 kg, obtained from the KrolInfo farm (Orekhovo-Zuyevo, Russia), were used in this study. Animals were kept under standard conditions (humidity 50–60%, temperature 19–22 °C). A 12-h lighting cycle was maintained. Each animal was kept in a separate cage. Animals had ad libitum access to standard extruded feed “CHARA” (CJSC “Assortiment-Agro”, Turakovo Village, Moscow region, Russia) and clean drinking water. Water treatment was performed using a “7 TECHNOCOM” block modular system (LLC “7 TECH”, Moscow, Russia). For in vivo, surface anesthesia measurements, an in-house-made anesthesiometer with a fixed length of a nylon filament (l = 1.0 cm, d = 0.125 mm) was used. Statistical analysis was performed, and figures were plotted using the Python programming language’s standard functions and a Matplotlib package (Version 3.8) for Python [35]. All biological data conformed to a normal distribution (Shapiro–Wilk’s W test, *p* > 0.05). All data are expressed as the mean ± standard deviation.

### 3.2. Chemistry

#### 3.2.1. General Method for the Synthesis of Compounds **6a**–**b**

To a solution of nitrosobenzene (**5**) in 100 mL of methanol, compound **4a** or **4b** (depending on the reaction) and 2 mL of acetic acid were added. The reaction mixture was stirred at RT for 72 h. After completion of the reaction (monitored by TLC), an equal volume of a 5% aqueous K_2_CO_3_ solution was added to the reaction mixture, followed by extraction with ethyl acetate from water. The organic layers were combined, washed with brine, and dried over anhydrous Na_2_SO_4_. The solvent was removed under reduced pressure. The product was purified by column chromatography (SiO_2_, hexane/EA).

Synthesis of *o*-methoxyazobenzene (**6a**)

Starting with compound **4a** (1.085 g, 8.810 mmol) and compound **5** (0.788 g, 7.357 mmol), the yield of compound **6a** was 30% (468 mg, yellow-orange amorphous solid).

^1^H NMR spectrum of compound **6a** (300 MHz, CDCl_3_) δ, ppm: 7.92 (m, 2H, 2×CH), 7.67 (m, 1H, 1×CH), 7.58–7.38 (m, 4H, 4×CH), 7.10 (m, 1H, 1×CH), 7.03 (m, 1H, 1×CH), 4.03 (s, 3H, -OCH_3_).

^13^C NMR spectrum of compound **6a** (75 MHz, CDCl_3_) δ, ppm: 157.1, 153.3, 142.4, 132.6, 130.9, 129.1, 123.1, 120.9, 117.1, 112.8, 56.4.

MS (ESI^+^) *m*/*z*: [M+H]^+^, calculated for (C_13_H_13_N_2_O)^+^ 213.1, found 213.1.

Synthesis of *m*-methoxyazobenzene (**6b**)

Starting with compound **4b** (1.278 g, 10.378 mmol) and compound **5** (0.927 g, 8.655 mmol), the yield of compound **6b** was 37% (679 mg, red-brown crystals).

^1^H NMR spectrum of compound **6b** (300 MHz, CDCl_3_) δ, ppm: 7.93 (m, 2H, 2×CH), 7.58–7.46 (m, 6H, 6×CH), 7.06 (m, 1H, 1×CH), 3.91 (s, 3H, -OCH_3_).

^13^C NMR spectrum of compound **6b** (75 MHz, CDCl_3_) δ, ppm: 160.4, 154.0, 152.7, 131.2, 129.9, 129.2, 123.0, 118.0, 117.3, 105.8, 55.6.

MS (ESI^+^) *m*/*z*: [M+H]^+^, calculated for (C_13_H_13_N_2_O)^+^ 213.1, found 213.0.

#### 3.2.2. General Method for the Synthesis of Compounds **7a**–**b**

A solution of compound **6a**–**b** in DCM (5 mL) was cooled to −78 °C. Then, a BBr_3_ solution in DCM (1:7, *v*/*v*) was added to the mixture with stirring, and the reaction was left at RT for 16 h. After the reaction was complete (monitored by TLC), the mixture was poured onto ice, followed by extraction with DCM from water. The combined organic layers were dried over anhydrous Na_2_SO_4_, and the solvent was removed under reduced pressure. The product was purified by column chromatography (SiO_2_, hexane/EA).

Synthesis of *o*-hydroxyazobenzene (**7a**)

Starting from compound **6a** (0.100 g, 0.471 mmol) and BBr_3_ (0.235 g, 0.938 mmol), the yield of compound **7a** was 58% (54 mg, red-orange crystals).

^1^H NMR spectrum of compound **7a** (300 MHz, DMSO-d6) δ, ppm: 11.18 (s, 1H, -OH), 7.98 (m, 2H, 2×CH), 7.76 (m, 1H, 1×CH), 7.65–7.48 (m, 3H, 3×CH), 7.43 (m, 1H, 1×CH), 7.12–6.95 (m, 2H, 2×CH).

^13^C NMR spectrum of compound **7a** (75 MHz, DMSO-d6) δ, ppm: 154.4, 151.4, 138.3, 133.6, 131.3, 129.4, 123.3, 122.6, 119.8, 118.2.

MS (ESI^+^) *m*/*z*: [M+H]^+^, calculated for (C_12_H_11_N_2_O)^+^ 199.1, found 199.0.

Synthesis of *m*-hydroxyazobenzene (**7b**)

Starting from compound **6b** (0.084 g, 0.396 mmol) and BBr_3_ (0.191 g, 0.762 mmol), the yield of compound **7b** was 48% (38 mg, yellow-orange amorphous solid).

^1^H NMR spectrum of compound **7b** (300 MHz, CDCl_3_) δ, ppm: 7.96–7.85 (m, 2H, 2×CH), 7.56–7.49 (m, 4H, 4×CH), 7.44–7.35 (m, 2H, 2×CH), 6.99 (m, 1H, 1×CH), 5.83 (s, 1H, -OH).

^13^C NMR spectrum of compound **7b** (75 MHz, CDCl_3_) δ, ppm: 156.6, 154.0, 152.6, 131.1, 130.2, 129.2, 123.0, 118.4, 117.3, 108.0.

MS (ESI^+^) *m*/*z*: [M+H]^+^, calculated for (C_12_H_11_N_2_O)^+^ 199.1, found 199.1.

#### 3.2.3. General Method for the Synthesis of Compounds **8a**–**b**

To a solution of **7a**–**b** in 10 mL of acetonitrile, *N*-(2-chloroethyl)-morpholine hydrochloride, K_2_CO_3_, and KI were added. The reaction was stirred under reflux for 6 h. After completion of the reaction (monitored by TLC), the solvent was removed under reduced pressure, and then the mixture was dissolved in ethyl acetate and washed with water. The organic layers were combined, washed with brine, and dried over anhydrous Na_2_SO_4_. The solvent was removed under reduced pressure. The product was purified by column chromatography (SiO_2_, EA/MeOH = 15:1).

Synthesis of 2-(2-(*N*-morpholino)-ethoxy)-azobenzene (**8a**)

Starting from compound **7a** (0.185 g, 0.933 mmol), *N*-(2-chloroethyl)-morpholine hydrochloride (0.260 g, 1.397 mmol), K_2_CO_3_ (0.386 g, 2.793 mmol), and KI (0.185 g, 1.114 mmol), the yield of compound **8a** was 82% (238 mg, orange amorphous solid).

^1^H NMR spectrum of compound **8a** (300 MHz, CDCl_3_) δ, ppm: 7.90 (m, 2H, 2×CH), 7.67 (m, 1H, 1×CH), 7.53–7.39 (m, 4H, 4×CH), 7.08 (m, 2H, 2×CH), 4.35 (t, *J* = 5.5 Hz, 2H, 1×CH_2_), 3.71 (m, 4H, 2×CH_2_), 2.91 (t, *J* = 5.5 Hz, 2H, 1×CH_2_), 2.68 (m, 4H, 2×CH_2_).

^13^C NMR spectrum of compound **8a** (75 MHz, CDCl_3_) δ, ppm: 156.4, 153.2, 142.8, 132.5, 131.0, 129.2, 123.0, 121.4, 117.1, 114.7, 68.5, 67.1, 57.7, 54.5.

MS (ESI^+^) *m*/*z*: [M+H]^+^, calculated for (C_18_H_22_N_3_O_2_)^+^ 312.2, found 312.3.

Synthesis of 3-(2-(*N*-morpholino)-ethoxy)-azobenzene (**8b**)

Starting from compound **7b** (0.097 g, 0.489 mmol), *N*-(2-chloroethyl)-morpholine hydrochloride (0.137 g, 0.736 mmol), K_2_CO_3_ (0.203 g, 1.469 mmol), and KI (0.081 g, 0.488 mmol), the yield of compound **8b** was 70% (106 mg, orange amorphous solid).

^1^H NMR spectrum of compound **8b** (300 MHz, CDCl_3_) δ, ppm: 7.91 (m, 2H, 2×CH), 7.61–7.39 (m, 6H, 6×CH), 7.05 (m, 1H, 1×CH), 4.20 (t, *J* = 5.7 Hz, 2H, 1×CH_2_), 3.84–3.63 (m, 4H, 2×CH_2_), 2.84 (t, *J* = 5.7 Hz, 2H, 1×CH_2_), 2.60 (m, 4H, 2×CH_2_).

^13^C NMR spectrum of compound **8b** (75 MHz, CDCl_3_) δ, ppm: 159.4, 153.9, 152.6, 131.2, 129.9, 129.2, 123.0, 118.3, 117.7, 106.3, 66.9, 65.9, 57.6, 54.1.

MS (ESI^+^) *m*/*z*: [M+H]^+^, calculated for (C_18_H_22_N_3_O_2_)^+^ 312.2, found 312.2.

#### 3.2.4. General Method for the Synthesis of Compounds **9a**–**b**

To a solution of compounds **8a**–**b** in 15 mL of Et_2_O, a solution of HCl/Et_2_O (2.4 M) was added with stirring at 0–5 °C. The reaction mixture was stirred for 30 min at RT, after which the product was concentrated under reduced pressure.

Synthesis of the 2-(2-(*N*-morpholino)-ethoxy)-azobenzene hydrochloride (**9a**)

Starting from compound **8a** (0.219 g, 0.703 mmol) and HCl/Et_2_O (590 μL, 2.4 M), the yield of compound **9a** was quantitative (244 mg, orange crystals).

^1^H NMR spectrum of compound **9a** (300 MHz, DMSO-d_6_) δ, ppm: 11.87 (s, 1H, HCl), 7.83 (m, 2H, 2×CH), 7.67–7.51 (m, 5H, 5×CH), 7.36 (m, 1H, 1×CH), 7.13 (t, *J* = 7.7 Hz, 1H, 1××CH), 4.70 (t, *J* = 4.8 Hz, 2H, 1×CH_2_), 3.86 (m, 4H, 2×CH_2_), 3.62 (m, 4H, 2×CH_2_), 3.32 (m, 2H, 1×CH_2_).

^13^C NMR spectrum of compound **9a** (75 MHz, DMSO-d_6_) δ, ppm: 155.2, 152.5, 141.6, 133.2, 131.5, 129.6, 122.5, 121.8, 116.5, 115.1, 64.4, 63.3, 55.0, 52.0.

MS (ESI^+^) *m*/*z*: [M-−HCl+H]^+^, calculated for (C_18_H_22_N_3_O_2_)^+^ 312.2, found 312.2.

Synthesis of the 3-(2-(*N*-morpholino)-ethoxy)-azobenzene hydrochloride (**9b**)

Starting from compound **8b** (0.125 g, 0.401 mmol) and HCl/Et_2_O (340 μL, 2.4 M), the yield of compound **9b** was quantitative (139 mg, light yellow crystals).

^1^H NMR spectrum of compound **9b** (300 MHz, DMSO-d_6_) δ, ppm: 11.61 (s, 1H, HCl), 7.90 (m, 2H, 2×CH), 7.65–7.52 (m, 5H, 5×CH), 7.48 (s, 1H, 1×CH), 7.23 (m, 1H, 1×CH), 4.57 (t, *J* = 5.1 Hz, 2H, 1×CH_2_), 4.04–3.77 (m, 4H, 2×CH_2_), 3.56 (m, 4H, 2×CH_2_), 3.22 (m, 2H, 1×CH_2_).

^13^C NMR spectrum of compound **9b** (75 MHz, DMSO-d_6_) δ, ppm: 158.4, 153.1, 151.8, 131.8, 130.5, 129.6, 122.6, 118.3, 117.2, 106.9, 63.2, 62.5, 54.8, 51.7.

MS (ESI^+^) *m*/*z*: [M−HCl+H]^+^, calculated for (C_18_H_22_N_3_O_2_)^+^ 312.2, found 312.2.

#### 3.2.5. Synthesis of 4-Hydroxyazobenzene (**11**)

Compound **11** was obtained using the procedure described earlier [20].

^1^H NMR spectrum of compound **11** (300 MHz, DMSO-d_6_) δ, ppm: 10.31 (s, 1H, -OH); 7.81 (m, 4H, 4×CH); 7.52 (m, 3H, 3×CH); 6.95 (m, 2H, 2×CH).

^13^C NMR spectrum of compound **11** (75 MHz, DMSO-d_6_) δ, ppm: 161.0, 152.1, 145.2, 130.5, 129.4, 124.9, 122.1, 116.0.

MS (ESI^+^) *m*/*z*: [M+H]^+^, calculated for (C_12_H_11_N_2_O)^+^ 199.1, found 199.1.

#### 3.2.6. Synthesis of 4-(1-Chloro-2-Ethoxy)-Azobenzene (**12**)

To a solution of compound **11** (1.000 g, 5.045 mmol) in acetonitrile (25 mL), K_2_CO_3_ (2.090 g, 15.123 mmol) and 1-bromo-2-chloroethane (1.260 mL, 2.171 g, 15.138 mmol) were added with stirring. The reaction mixture was stirred under reflux for 24 h and then extracted with ethyl acetate from water. The organic layer was washed with and dried over Na_2_SO_4_ (anhydrous). The solvent was removed under reduced pressure. After purification by chromatography (SiO_2_, hexane/EA = 5:1), the yield of compound **12** was 68% (894 mg, orange crystals).

^1^H NMR spectrum of compound **12** (300 MHz, CDCl_3_) δ, ppm: 7.97–7.86 (m, 4H, 4×CH), 7.56–7.42 (m, 3H, 3×CH), 7.03 (m, 2H, 2×CH), 4.30 (t, *J* = 5.8 Hz, 2H, 1×CH_2_), 3.85 (t, *J* = 5.8 Hz, 2H, 1×CH_2_).

^13^C NMR spectrum of compound **12** (75 MHz, CDCl_3_) δ, ppm: 160.7, 152.8, 147.5, 130.6, 129.2, 124.9, 122.7, 115.0, 68.3, 41.8.

#### 3.2.7. Synthesis of 4-(2-(4-Methyl-1-Piperazino)-Ethoxy)-Azobenzene (**13**)

To a solution of compound **12** (516 mg, 1.979 mmol) in acetonitrile (15 mL), K_2_CO_3_ (819 mg, 5.926 mmol), 1-methylpiperazine (220 μL, 199 mg, 1.987 mmol), and KI (329 mg, 1.982 mmol) were added. The reaction mixture was stirred under reflux for 24 h and then extracted with ethyl acetate from water. The organic layer was washed with brine and dried over Na_2_SO_4_ (anhydrous). The solvent was removed under reduced pressure. After purification by chromatography (SiO_2_, DCM/MeOH/NH_4_OH = 90:10:1), the yield of compound **13** was 52% (333 mg, amorphous yellow solid).

^1^H NMR spectrum of compound **13** (300 MHz, DMSO-d_6_) δ, ppm: 7.85 (m, 4H, 4×CH), 7.65–7.46 (m, 3H, 3×CH), 7.13 (m, 2H, 2×CH), 4.17 (t, *J* = 5.8 Hz, 2H, 1×CH_2_), 2.71 (t, *J* = 5.8 Hz, 2H, 1×CH_2_), 2.46 (m, 4H, 2×CH_2_), 2.32 (m, 4H, 2×CH_2_), 2.14 (s, 3H, -CH_3_).

^13^C NMR spectrum of compound **13** (75 MHz, DMSO-d_6_) δ, ppm: 161.3, 152.0, 146.1, 130.8, 129.3, 124.6, 122.3, 115.1, 66.1, 56.5, 54.7, 53.0, 45.8.

MS (ESI^+^) *m*/*z*: [M+H]^+^, calculated for (C_19_H_25_N_4_O)^+^ 325.2, found 325.2.

#### 3.2.8. Synthesis of the 4-(2-(4-Methyl-1-Piperazino)-Ethoxy)-Azobenzene Dihydrochloride (**14**)

To a solution of compound **13** (191 mg, 0.588 mmol) in diethyl ether (32 mL), a solution of HCl/Et_2_O (1.000 mL, 2.4 M) was added with stirring at 0–5 °C. The resulting mixture was stirred at RT for 30 min. The solvent was removed under reduced pressure. The yield of compound **14** was quantitative (233 mg, orange crystals).

^1^H NMR spectrum of compound **14** (300 MHz, DMSO-d_6_) δ, ppm: 12.06 (br s, HCl), 7.87 (m, 2H, 2×CH), 7.85 (m, 2H, 2×CH), 7.62–7.48 (m, 3H, 3×CH), 7.22 (m, 2H, 2×CH), 4.55 (m, 2H, 1×CH_2_), 3.91–3.55 (m, 10H, 5×CH_2_), 2.83 (s, 3H, -CH_3_).

^13^C NMR spectrum of compound **14** (75 MHz, D_2_O) δ (ppm): 159.8, 151.8, 146.6, 131.2, 129.4, 124.5, 122.1, 115.0, 61.6, 55.5, 50.1, 49.0, 42.8.

MS (ESI^+^) *m*/*z*: [M−HCl+H]^+^, calculated for (C_19_H_25_N_4_O)^+^ 325.2, found 325.2.

#### 3.2.9. Synthesis of 4-(2-(*N*,*N*-Diethylamino)-Ethoxy)-Azobenzene (**15**)

To a solution of compound **11** (500 mg, 2.522 mmol) in acetonitrile (15 mL), K_2_CO_3_ (1047 mg, 7.576 mmol), 2-chloro-*N*,*N*-diethylethan-1-amine hydrochloride (1086 mg, 6.311 mmol), and KI (419 mg, 2.524 mmol) were added. The reaction mixture was stirred and heated for 3 h, after which it was extracted with ethyl acetate from water. The organic layer was washed with brine and dried using anhydrous Na_2_SO_4_. The solvent was removed under reduced pressure. After purification by column chromatography (SiO_2_, DCM/MeOH/NH_4_OH = from 200:10:1 to 25:10:1), compound **15** was obtained with an 18% yield (135 mg, amorphous orange solid).

^1^H NMR spectrum of compound **15** (300 MHz, DMSO-d_6_) δ (ppm): 7.94–7.78 (m, 4H, 4×CH), 7.63–7.46 (m, 3H, 3×CH), 7.13 (m, 2H, 2×CH), 4.12 (t, *J* = 6.1 Hz, 2H, 1×CH_2_), 2.80 (t, *J* = 6.1 Hz, 2H, 1×CH_2_), 2.56 (q, *J* = 7.1 Hz, 4H, 2×CH_2_), 0.98 (t, *J* = 7.1 Hz, 6H, 2×CH_3_).

^13^C NMR spectrum of compound **15** (75 MHz, DMSO-d_6_) δ (ppm): 161.3, 152.0, 146.1, 130.7, 129.3, 124.5, 122.2, 115.0, 66.9, 51.2, 47.0, 11.9.

MS (ESI^+^) *m*/*z*: [M+H]^+^, calculated for (C_18_H_24_N_3_O)^+^ 298.2, found 298.2.

#### 3.2.10. Synthesis of 4-(2-(*N*,*N*-Diethylamino)Ethoxy)Azobenzene Hydrochloride (**16**)

To a solution of compound **15** (100 mg, 0.336 mmol) in diethyl ether (10 mL), a solution of HCl/Et_2_O (400 μL, 2.4 M) was added under stirring at 0–5 °C. The resulting mixture was stirred at RT for 30 min. The solvent was removed under reduced pressure. Compound **16** (112 mg, orange crystals) was obtained in quantitative yield.

^1^H NMR spectrum of compound **16** (300 MHz, DMSO-d_6_) δ, ppm: 11.01 (s, 1H, HCl), 7.93 (m, 2H, 2×CH), 7.85 (m, 2H, 2×CH), 7.64–7.49 (m, 3H, 3×CH), 7.20 (m, 2H, 2×CH), 4.54 (t, *J* = 5.1 Hz, 2H, 1×CH_2_), 3.53 (q, *J* = 5.0 Hz, 2H, 1×CH_2_), 3.21 (m, 4H, 2×CH_2_), 1.28 (t, *J* = 7.2 Hz, 6H, 2×CH_3_).

^13^C NMR spectrum of compound **16** (75 MHz, DMSO-d_6_) δ, ppm: 160.2, 151.9, 146.6, 130.9, 129.3, 124.5, 122.2, 115.3, 62.7, 49.5, 46.9, 8.4.

MS (ESI^+^) *m*/*z*: [M−HCl+H]^+^, calculated for (C_18_H_24_N_3_O)^+^ 298.2, found 298.2.

#### 3.2.11. General Procedure for the Synthesis of Compounds **18a**–**h**

To solution of **10a**–**c** in an aqueous HCl (12% *w*/*w*), a solution of NaNO_2_ in water was added dropwise under stirring and cooling (0–5 °C). The reaction mixture was stirred for 30 min at 0–5 °C. Then, a solution of **17a**–**c** in aqueous NaOH (14% *w*/*w*) was added dropwise to the reaction mixture under stirring over 10 min. The reaction was then stirred at 0–5 °C for 1 h. Upon completion, the pH of the reaction mixture was adjusted to 5–6, and the resulting precipitate was filtered and dried. The crude product was purified by column chromatography (SiO_2_, hexane/EA = 5:1).

Synthesis of 2-fluoro-4-hydroxyazobenzene (**18a**).

Starting from compound **10a** (1.000 g, 10.738 mmol) in 12% aqueous HCl (40 mL), NaNO_2_ (0.741 g, 10.740 mmol) in water (10 mL), and compound **17b** (1.204 g, 10.740 mmol) in 14% aqueous NaOH (40 mL), compound **18a** was obtained with a yield of 65% (1.509 g, orange crystals).

^1^H NMR spectrum of compound **18a** (300 MHz, DMSO-d_6_) δ, ppm: 10.79 (s, 1H, -OH), 7.86–7.76 (m, 2H, 2×CH), 7.70 (t, *J* = 8.9 Hz, 1H, 1×CH), 7.64–7.44 (m, 3H, 3×CH), 6.87–6.70 (m, 2H, 2×CH).

^13^C NMR spectrum of compound **18a** (75 MHz, DMSO-d_6_) δ, ppm: 162.6 (d, *J* = 12.1 Hz), 159.4 (d, *J* = 255.9 Hz), 152.3, 133.1 (d, *J* = 6.8 Hz), 130.8, 129.3, 122.2, 118.4 (d, *J* = 2.2 Hz), 112.4 (d, *J* = 2.4 Hz), 103.3 (d, *J* = 21.8 Hz).

MS (ESI^−^) *m*/*z*: [M−H]^−^ calculated for (C_12_H_8_FN_2_O)^−^ 215.1, found: 215.1.

Synthesis of 2,6-difluoro-4-hydroxyazobenzene (**18b**).

Starting from compound **10a** (1.000 g, 10.738 mmol) in 12% aqueous HCl (40 mL), NaNO_2_ (0.741 g, 10.741 mmol) in water (10 mL), and compound **17c** (1.397 g, 10.739 mmol) in 14% aqueous NaOH (40 mL), compound **18b** was obtained with a yield of 75% (1.886 g, orange crystals).

^1^H NMR spectrum of compound **18b** (300 MHz, acetone-d_6_) δ, ppm: 9.83 (s, 1H, -OH), 7.85 (m, 2H, 2×CH), 7.62–7.46 (m, 3H, 3×CH), 6.68 (m, 2H, 2×CH).

^13^C NMR spectrum of compound **18b** (75 MHz, acetone-d_6_) δ, ppm: 161.5 (t, *J* = 14.9 Hz), 158.4 (dd, *J* = 258.5, 7.6 Hz), 154.4, 132.0, 130.1, 125.3 (t, *J* = 9.6 Hz), 123.1, 101.07 (dd, *J* = 23.4, 3.0 Hz).

MS (ESI^−^) *m*/*z*: [M−H]^−^ calculated for (C_12_H_7_F_2_N_2_O)^−^ 233.0, found: 233.1.

Synthesis of 2′-fluoro-4-hydroxyazobenzene (**18c**).

Starting from compound **10b** (1.000 g, 8.999 mmol) in 12% aqueous HCl (40 mL), NaNO_2_ (0.621 g, 9.001 mmol) in water (10 mL), and compound **17a** (0.847 g, 9.000 mmol) in 14% aqueous NaOH (40 mL), compound **18c** was obtained with a yield of 77% (1.498 g, orange crystals).

^1^H NMR spectrum of compound **18c** (300 MHz, DMSO-d_6_) δ, ppm: 10.43 (s, 1H, -OH), 7.82 (m, 2H, 2×CH), 7.66 (m, 1H, 1×CH), 7.55–7.35 (m, 2H, 2×CH), 7.29 (m, 1H, 1×CH), 6.96 (m, 2H, 2×CH).

^13^C NMR spectrum of compound **18c** (75 MHz, DMSO-d_6_) δ, ppm: 161.8, 159.3 (d, *J* = 254.3 Hz), 146.1, 140.4 (d, *J* = 6.8 Hz), 132.7 (d, *J* = 8.2 Hz), 125.7, 125.3 (d, *J* = 3.6 Hz), 117.9, 117.5 (d, *J* = 19.6 Hz), 116.5.

MS (ESI^−^) *m*/*z*: [M−H]^−^ calculated for (C_12_H_8_FN_2_O)^−^ 215.1, found: 215.0.

Synthesis of 2,2′-difluoro-4-hydroxyazobenzene (**18d**).

Starting from compound **10b** (1.000 g, 8.999 mmol) in 12% aqueous HCl (40 mL), NaNO_2_ (0.621 g, 9.001 mmol) in water (10 mL), and compound **17b** (1.009 g, 9.001 mmol) in 14% aqueous NaOH (40 mL), compound **18d** was obtained with a yield of 73% (1.539 g, orange crystals).

^1^H NMR spectrum of compound **18d** (300 MHz, DMSO-d_6_) δ, ppm: 10.87 (s, 1H, -OH), 7.73–7.59 (m, 2H, 2×CH), 7.58–7.49 (m, 1H, 1×CH), 7.43 (m, 1H, 1×CH), 7.30 (m, 1H, 1×CH), 6.85–6.70 (m, 1H, 2×CH).

^13^C NMR spectrum of compound **18d** (75 MHz, DMSO-d_6_) δ, ppm: 163.2 (d, *J* = 12.3 Hz), 161.8 (d, *J* = 178.4 Hz), 158.5 (d, *J* = 176.5 Hz), 140.2 (d, *J* = 6.9 Hz), 133.5 (d, *J* = 6.8 Hz), 132.6 (d, *J* = 8.3 Hz), 124.9 (d, *J* = 3.7 Hz), 118.6 (d, *J* = 1.9 Hz), 117.5, 117.1 (d, *J* = 19.6 Hz), 112.6 (d, *J* = 2.4 Hz), 103.4 (d, *J* = 21.8 Hz).

MS (ESI^−^) *m*/*z*: [M−H]^−^, calculated for (C_12_H_7_F_2_N_2_O)^−^ 233.0, found: 233.1.

Synthesis of 2,2′,6-trifluoro-4-hydroxyazobenzene (**18e**).

Starting from compound **10b** (1.000 g, 8.999 mmol) in 12% aqueous HCl (40 mL), NaNO_2_ (0.621 g, 9.001 mmol) in water (10 mL), and compound **17c** (1.171 g, 9.001 mmol) in 14% aqueous NaOH (40 mL), compound **18e** was obtained with a yield of 70% (1.589 g, orange crystals).

^1^H NMR spectrum of compound **18e** (300 MHz, acetone-d_6_) δ, ppm: 10.00 (s, 1H, OH), 7.68 (m, 1H, 1×CH), 7.62–7.52 (m, 1H, 1×CH), 7.44–7.26 (m, 2H, 2×CH), 6.67 (m, 2H, 2×CH).

^13^C NMR spectrum of compound **18e** (75 MHz, acetone-d_6_) δ, ppm: 162.4 (t, *J* = 14.8 Hz), 160.6 (d, *J* = 255.1 Hz), 158.9 (dd, *J* = 259.3, 8.0 Hz), 142.3 (d, *J* = 7.1 Hz), 133.8 (d, *J* = 8.3 Hz), 125.5 (m), 117.9, 117.9 (d, *J* = 19.6 Hz), 101.1 (dd, *J* = 23.2, 3.1 Hz).

MS (ESI^−^) *m*/*z*: [M−H]^−^, calculated for (C_12_H_6_F_3_N_2_O)^−^ 251.0, found: 251.0.

Synthesis of 2′,6′-difluoro-4-hydroxyazobenzene (**18f**).

Starting from compound **10c** (1.000 g, 7.745 mmol) in 12% aqueous HCl (40 mL), NaNO_2_ (0.534 g, 7.740 mmol) in water (10 mL), and compound **17a** (0.729 g, 7.746 mmol) in 14% aqueous NaOH (40 mL), compound **18f** was obtained with a yield of 68% (1.233 g, orange crystals).

^1^H NMR spectrum of compound **18f** (300 MHz, CDCl_3_) δ, ppm: 7.88 (m, 2H, 2×CH), 7.26 (m, 1H, 1×CH), 7.03 (m, 2H, 2×CH), 6.93 (m, 2H, 2×CH), 5.76 (s, 1H, OH).

^13^C NMR spectrum of compound **18f** (75 MHz, CDCl_3_) δ, ppm: 159.5, 155.8 (dd, *J* = 257.8, 4.5 Hz), 147.8, 131.5 (t, *J* = 10.7 Hz), 129.8 (t, *J* = 10.4 Hz), 125.4, 116.1, 112.6 (m).

MS (ESI^−^) *m*/*z*: [M−H]^−^, calculated for (C_12_H_7_F_2_N_2_O)^−^ 233.0, found: 233.1.

Synthesis of 2,2′,6′-trifluoro-4-hydroxyazobenzene (**18g**).

Starting from compound **10c** (1.000 g, 7.745 mmol) in 12% aqueous HCl (40 mL), NaNO_2_ (0.534 g, 7.740 mmol) in water (10 mL), and compound **17b** (0.868 g, 7.743 mmol) in 14% aqueous NaOH (40 mL), compound **18g** was obtained with a yield of 60% (1.171 g, orange crystals).

^1^H NMR spectrum of compound **18g** (300 MHz, acetone-d_6_) δ, ppm: 11.02 (s, 1H, -OH), 7.63 (t, *J* = 8.8 Hz, 1H, 1×CH), 7.47 (m, 1H, 1×CH), 7.32–7.18 (m, 2H, 2×CH), 6.86–6.72 (m, 2H, 2×CH).

MS (ESI^−^) *m*/*z*: [M−H]^−^, calculated for (C_12_H_6_F_3_N_2_O)^−^ 251.0, found: 251.1.

Synthesis of 2,2′,6,6′-tetrafluoro-4-hydroxyazobenzene (**18h**).

Starting from compound **10c** (1.000 g, 7.745 mmol) in 12% aqueous HCl (40 mL), NaNO_2_ (0.534 g, 7.740 mmol) in water (10 mL), and compound **17c** (1.007 g, 7.741 mmol) in 14% aqueous NaOH (40 mL), compound **18h** was obtained with a yield of 36% (753 mg, orange crystals).

^1^H NMR spectrum of compound **18h** (300 MHz, acetone-d_6_) δ, ppm: 10.07 (s, 1H, -OH), 7.59–7.41 (m, 1H, 1×CH), 7.35–7.13 (m, 2H, 2×CH), 6.75–6.62 (m, 2H, 2×CH).

^13^C NMR spectrum of compound **18h** (75 MHz, acetone-d_6_) δ, ppm: 162.9 (t, *J* = 15.0 Hz), 158.4 (dd, *J* = 260.4, 7.4 Hz), 156.0 (dd, *J* = 257.1, 4.5 Hz), 132.7 (t, *J* = 10.6 Hz), 131.8 (t, *J* = 10.4 Hz), 125.8 (t, *J* = 9.4 Hz), 113.5 (m), 101.1 (dd, *J* = 23.0, 3.0 Hz).

MS (ESI^+^) *m*/*z*: [M+H]^+^, calculated for (C_12_H_7_F_4_N_2_O)^+^ 271.0, found: 271.0.

#### 3.2.12. General Procedure for the Preparation of Compounds **19a**–**h**

A solution of compound **18a**–**f** in acetonitrile was treated with K_2_CO_3_, KI, and *N*-(2-chloroethyl)-morpholine hydrochloride. The reaction mixture was stirred for 6 h under reflux, and the progress of the reaction was monitored by TLC. The mixture was filtered, and the filtrate was concentrated under reduced pressure. The product was extracted with ethyl acetate from water. The organic layer was washed with brine and dried over anhydrous Na_2_SO_4_. The reaction products **19a**–**f** were purified by column chromatography (SiO_2_, EA/MeOH = 5:1).

Preparation of 2-fluoro-4-(2-(*N*-morpholino)-ethoxy)-azobenzene (**19a**).

Starting from compound **18a** (212 mg, 0.980 mmol) in acetonitrile (15 mL), K_2_CO_3_ (406 mg, 2.938 mmol), KI (163 mg, 0.982 mmol), and *N*-(2-chloroethyl)-morpholine hydrochloride (274 mg, 1.472 mmol), the yield of compound **19a** was 61% (197 mg, orange amorphous solid).

^1^H NMR spectrum of compound **19a** (300 MHz, DMSO-d_6_) δ, ppm: 7.92–7.79 (m, 2H, 2×CH), 7.74 (t, *J* = 9.0 Hz, 1H, 1×CH), 7.63–7.47 (m, 3H, 3×CH), 7.13 (dd, *J* = 13.1, 2.6 Hz, 1H, 1×CH), 6.91 (m, 1H, 1×CH), 4.20 (t, *J* = 5.7 Hz, 2H, 1×CH_2_), 3.58 (m, 4H, 2×CH_2_), 2.71 (t, *J* = 5.6 Hz, 2H, 1×CH_2_), 2.47 (m, 4H, 2×CH_2_).

^13^C NMR spectrum of compound **19a** (75 MHz, DMSO-d_6_) δ, ppm: 162.7 (d, *J* = 5.5 Hz), 161.0 (d, *J* = 273.0 Hz), 152.2, 133.9 (d, *J* = 7.0 Hz), 131.2, 129.4, 122.4, 118.2 (d, *J* = 2.1 Hz), 112.1 (d, *J* = 2.8 Hz), 102.7 (d, *J* = 23.5 Hz), 66.4, 66.2, 56.8, 53.6.

MS (ESI^+^) *m*/*z*: [M+H]^+^, calculated for (C_18_H_21_FN_3_O_2_)^+^ 330.2, found 330.1.

Preparation of 2,6-difluoro-4-(2-(*N*-morpholino)-ethoxy)-azobenzene (**19b**).

Starting from compound **18b** (500 mg, 2.135 mmol) in acetonitrile (15 mL), K_2_CO_3_ (885 mg, 6.404 mmol), KI (354 mg, 2.132 mmol), and *N*-(2-chloroethyl)-morpholine hydrochloride (596 mg, 3.203 mmol), the yield of compound **19b** was 64% (474 mg, orange amorphous solid).

^1^H NMR spectrum of compound **19b** (300 MHz, acetone-d_6_) δ, ppm: 7.87 (m, 2H, 2×CH), 7.63–7.45 (m, 3H, 3×CH), 6.80 (m, 2H, 2×CH), 4.24 (t, *J* = 5.7 Hz, 2H, 1×CH_2_), 3.60 (m, 4H, 2×CH_2_), 2.77 (t, *J* = 5.7 Hz, 2H, 1×CH_2_), 2.52 (m, 4H, 2×CH_2_).

^13^C NMR spectrum of compound **19b** (75 MHz, acetone-d_6_) δ, ppm: 162.3 (t, *J* = 14.2 Hz), 158.2 (dd, *J* = 258.3, 7.5 Hz), 154.4, 132.2, 130.1, 125.8 (t, *J* = 9.8 Hz), 123.2, 100.4 (dd, *J* = 24.4, 3.0 Hz), 68.1, 67.4, 57.9, 54.9.

MS (ESI^+^) *m*/*z*: [M+H]^+^, calculated for (C_18_H_20_F_2_N_3_O_2_)^+^ 348.1, found 348.2.

Preparation of 2′-fluoro-4-(2-(*N*-morpholino)-ethoxy)-azobenzene (**19c**).

Starting from compound **18c** (594 mg, 2.747 mmol) in acetonitrile (30 mL), K_2_CO_3_ (1.139 g, 8.242 mmol), KI (456 mg, 2.747 mmol), and *N*-(2-chloroethyl)-morpholine hydrochloride (767 mg, 4.122 mmol), the yield of compound **19c** was 70% (633 mg, orange amorphous solid).

^1^H NMR spectrum of compound **19c** (300 MHz, CDCl_3_) δ, ppm: 7.94 (m, 2H, 2×CH); 7.73 (m, 1H, 1×CH); 7.45–7.35 (m, 1H, 1×CH); 7.30–7.18 (m, 2H, 2×CH); 7.02 (m, 2H, 2×CH); 4.20 (t, *J* = 5.7 Hz, 2H, 1×CH_2_); 3.75 (m, 4H, 2×CH_2_); 2.84 (t, *J* = 5.7 Hz, 2H, 1×CH_2_); 2.60 (m, 4H, 2×CH_2_).

^13^C NMR spectrum of compound **19c** (75 MHz, CDCl_3_) δ, ppm: 161.6, 159.9 (d, *J* = 257.2 Hz), 147.5, 140.9 (d, *J* = 6.9 Hz), 131.8 (d, *J* = 8.1 Hz), 125.2, 124.3 (d, *J* = 3.8 Hz), 117.9, 117.02 (d, *J* = 20.1 Hz), 114.9, 66.9, 66.2, 57.6, 54.2.

MS (ESI^+^) *m*/*z*: [M+H]^+^, calculated for (C_18_H_21_FN_3_O_2_)^+^ 330.2, found 330.3.

Synthesis of 2,2′-difluoro-4-(2-(*N*-morpholino)-ethoxy)-azobenzene (**19d**).

Starting from compound **18d** (1.217 g, 5.196 mmol) in acetonitrile (45 mL), with K_2_CO_3_ (2.154 g, 15.586 mmol), KI (863 mg, 5.199 mmol), and *N*-(2-chloroethyl)-morpholine hydrochloride (1.450 g, 7.792 mmol), compound **19d** was obtained in 57% yield (1.029 g, orange amorphous solid).

^1^H NMR spectrum of compound **19d** (300 MHz, DMSO-d_6_) δ, ppm: 7.76–7.53 (m, 3H, 3×CH), 7.52–7.42 (m, 1H, 1×CH), 7.32 (m, 1H, 1×CH), 7.14 (dd, *J* = 13.0, 2.6 Hz, 1H, 1×CH), 6.92 (dd, *J* = 9.1, 2.6 Hz, 1H, 1×CH), 4.21 (t, *J* = 5.6 Hz, 2H, 1×CH_2_), 3.58 (m, 4H, 2×CH_2_), 2.71 (t, *J* = 5.6 Hz, 2H, 1×CH_2_), 2.47 (m, 4H, 2×CH_2_).

^13^C NMR spectrum of compound **19d** (75 MHz, DMSO-d_6_) δ, ppm: 163.3 (d, *J* = 11.3 Hz), 161.2 (d, *J* = 257.1 Hz), 159.2 (d, *J* = 255.5 Hz), 140.1 (d, *J* = 6.7 Hz), 134.3 (d, *J* = 6.8 Hz), 133.2 (d, *J* = 8.4 Hz), 125.0 (d, *J* = 3.6 Hz), 118.4 (d, *J* = 1.9 Hz), 117.5, 117.3 (d, *J* = 19.5 Hz), 112.3 (d, *J* = 2.6 Hz), 102.7 (d, *J* = 23.3 Hz), 66.4, 66.2, 56.8, 53.6.

MS (ESI^+^) *m*/*z*: [M+H]^+^, calculated for (C_18_H_20_F_2_N_3_O_2_)^+^ 348.1, found 348.2.

Synthesis of 2,2′,6-trifluoro-4-(2-(*N*-morpholino)-ethoxy)-azobenzene (**19e**).

Starting from compound **18e** (500 mg, 1.982 mmol) in acetonitrile (15 mL), with K_2_CO_3_ (822 mg, 5.948 mmol), KI (329 mg, 1.982 mmol), and *N*-(2-chloroethyl)-morpholine hydrochloride (553 mg, 2.972 mmol), compound **19e** was obtained in 51% yield (369 mg, orange amorphous solid).

^1^H NMR spectrum of compound **19e** (300 MHz, acetone-d_6_) δ, ppm: 7.69 (m, 1H, 1×CH), 7.59 (m, 1H, 1×CH), 7.45–7.25 (m, 2H, 2×CH), 6.84 (m, 2H, 2×CH), 4.30 (t, *J* = 5.8 Hz, 2H, 1×CH_2_), 3.62 (m, 4H, 2×CH_2_), 2.80 (t, *J* = 5.7 Hz, 2H, 1×CH_2_), 2.54 (m, 4H, 2×CH_2_).

^13^C NMR spectrum of compound **19e** (75 MHz, CDCl_3_) δ, ppm: 161.2 (t, *J* = 13.4 Hz), 158.3 (dd, *J* = 259.4, 7.4 Hz), 151.8 (d, *J* = 249.6 Hz), 142.3 (d, *J* = 6.9 Hz), 131.1 (d, *J* = 7.7 Hz), 125.5 (t, *J* = 3.7 Hz), 121.8, 117.9, 117.3 (d, *J* = 19.8 Hz), 99.8 (dd, *J* = 24.7, 2.9 Hz), 67.9, 67.4, 57.9, 54.8.

MS (ESI^+^) *m*/*z*: [M+H]^+^, calculated for (C_18_H_19_F_3_N_3_O_2_)^+^ 366.1, found 366.2.

Synthesis of 2′,6′-difluoro-4-(2-(*N*-morpholino)-ethoxy)-azobenzene (**19f**).

Starting from compound **18f** (347 mg, 1.482 mmol) in acetonitrile (20 mL), with K_2_CO_3_ (614 mg, 4.443 mmol), KI (246 mg, 1.482 mmol), and *N*-(2-chloroethyl)-morpholine hydrochloride (413 mg, 2.219 mmol), the yield of compound **19f** was 56% (288 mg, orange amorphous solid).

^1^H NMR spectrum of compound **19f** (300 MHz, CDCl_3_) δ, ppm: 7.92 (m, 2H, 2×CH), 7.34–7.20 (m, 1H, 1×CH), 7.07–7.00 (m, 4H, 4×CH), 4.20 (t, *J* = 5.7 Hz, 2H, 1×CH_2_), 3.77–3.72 (m, 4H, 2×CH_2_), 2.84 (t, *J* = 5.7 Hz, 2H, 1×CH_2_), 2.63–2.57 (m, 4H, 2×CH_2_).

^13^C NMR spectrum of compound **19f** (75 MHz, CDCl_3_) δ, ppm: 162.0, 155.7 (dd, *J* = 257.9, 4.6 Hz), 147.8, 131.5 (t, low intensity), 129.6 (t, *J* = 10.2 Hz), 125.1, 114.9, 112.5 (m), 66.9, 66.2, 57.5, 54.1.

MS (ESI^+^) *m*/*z*: [M+H]^+^, calculated for (C_18_H_20_F_2_N_3_O_2_)^+^ 348.1, found: 348.2.

Synthesis of 2,2′,6′-trifluoro-4-(2-(*N*-morpholino)-ethoxy)-azobenzene (**19g**).

Starting from compound **18g** (946 mg, 3.751 mmol) in acetonitrile (45 mL), with K_2_CO_3_ (1.555 g, 11.252 mmol), KI (623 mg, 3.753 mmol), and *N*-(2-chloroethyl)-morpholine hydrochloride (1.047 g, 5.627 mmol), the yield of compound **19g** was 77% (1.055 g, orange amorphous solid).

^1^H NMR spectrum of compound **19g** (300 MHz, DMSO-d_6_) δ, ppm: 7.68 (t, *J* = 8.9 Hz, 1H, 1×CH), 7.57–7.46 (m, 1H, 1×CH), 7.29 (m, 2H, 2×CH), 7.14 (m, 1H, 1×CH), 6.92 (m, 1H, 1×CH), 4.22 (t, *J* = 5.6 Hz, 2H, 1×CH_2_), 3.58 (m, 4H, 2×CH_2_), 2.71 (t, *J* = 5.6 Hz, 2H, 1×CH_2_), 2.47 (m, 4H, 2×CH_2_).

^13^C NMR spectrum of compound **19g** (75 MHz, DMSO-d_6_) δ, ppm: 163.8 (d, *J* = 11.5 Hz), 161.3 (d, *J* = 258.0 Hz), 154.9 (dd, *J* = 256.9, 4.5 Hz), 134.7 (d, *J* = 7.0 Hz), 131.4 (t, *J* = 10.4 Hz), 130.6 (t, *J* = 10.3 Hz), 117.8 (d, *J* = 1.7 Hz), 113.0 (m), 112.3 (d, *J* = 2.6 Hz), 102.7 (d, *J* = 23.4 Hz), 66.5, 66.2, 56.7, 53.6.

MS (ESI^+^) *m*/*z*: [M+H]^+^, calculated for (C_18_H_19_F_3_N_3_O_2_)^+^ 366.1, found: 366.1.

Synthesis of 2,2′,6,6′-tetrafluoro-4-(2-(*N*-morpholino)-ethoxy)-azobenzene (**19h**).

Starting from compound **18h** (573 mg, 2.121 mmol) in acetonitrile (30 mL), with K_2_CO_3_ (880 mg, 6.368 mmol), KI (352 mg, 2.120 mmol), and *N*-(2-chloroethyl)-morpholine hydrochloride (592 mg, 3.181 mmol), the yield of compound **19h** was 76% (618 mg, orange amorphous solid).

^1^H NMR spectrum of compound **19h** (300 MHz, DMSO-d_6_) δ, ppm: 7.48–7.36 (m, 1H), 7.19 (t, *J* = 8.6 Hz, 2H, 2×CH), 6.86 (m, 2H, 2×CH), 4.09 (t, *J* = 5.6 Hz, 2H, 1×CH_2_), 3.53 (m, 4H, 2×CH_2_), 2.63 (t, *J* = 5.6 Hz, 2H, 1×CH_2_), 2.41 (m, 4H, 2×CH_2_).

^13^C NMR spectrum of compound **19h** (75 MHz, acetone-d_6_) δ, ppm: 163.5 (t, *J* = 14.4 Hz), 158.2 (dd, *J* = 260.2, 7.3 Hz), 156.1 (dd, *J* = 257.7, 4.4 Hz), 132.7 (t, *J* = 10.6 Hz), 132.1 (t, *J* = 10.4 Hz), 126.5 (t, *J* = 9.7 Hz), 113.6 (m), 100.5 (dd, *J* = 24.1, 3.2 Hz), 68.3, 67.5, 57.9, 54.9.

MS (ESI^+^) *m*/*z*: [M+H]^+^, calculated for (C_18_H_18_F_4_N_3_O_2_)^+^ 384.1, found: 384.2.

#### 3.2.13. General Method for the Synthesis of Compounds **20a**–**h**

To the solutions of compounds **19a**–**f** in diethyl ether, a solution of HCl/Et_2_O (2.4 M) was added dropwise with stirring at 0–5 °C. The reaction mixture was then stirred for 30 min at RT. The product was concentrated under reduced pressure.

Synthesis of 2-fluoro-4-(2-(*N*-morpholino)-ethoxy)-azobenzene hydrochloride (**20a**).

Starting from compound **19a** (128 mg, 0.389 mmol) in Et_2_O (20 mL) and HCl/Et_2_O (328 µL, 2.4 M), the yield of compound **20a** was quantitative (142 mg, light orange crystals).

^1^H NMR spectrum of compound **20a** (300 MHz, DMSO-d_6_) δ, ppm: 11.81 (s, 1H, HCl), 7.89–7.82 (m, 2H, 2×CH), 7.79 (t, *J* = 8.9 Hz, 1H, 1×CH), 7.63–7.53 (m, 3H, 3×CH), 7.23 (dd, *J* = 12.8, 2.6 Hz, 1H, 1×CH), 7.00 (m, 1H, 1×CH), 4.60 (t, *J* = 5.0 Hz, 2H, 1×CH_2_), 4.01–3.82 (m, 4H, 2×CH_2_), 3.65–3.44 (m, 4H, 2×CH_2_), 3.22 (s, 2H, 1×CH_2_).

^13^C NMR spectrum of compound **20a** (75 MHz, DMSO-d_6_) δ, ppm: 161.6 (d, *J* = 11.2 Hz), 160.8 (d, *J* = 256.5 Hz), 152.1, 134.4 (d, *J* = 7.1 Hz), 131.5, 129.5, 122.6, 118.4 (d, *J* = 1.2 Hz), 112.2 (d, *J* = 2.7 Hz), 103.2 (d, *J* = 23.7 Hz), 63.3, 63.2, 54.5, 51.6.

MS (ESI^+^) *m*/*z*: [M−HCl+H]^+^, calculated for (C_18_H_21_FN_3_O_2_)^+^ 330.2, found 330.2.

Synthesis of 2,6-difluoro-4-(2-(*N*-morpholino)-ethoxy)-azobenzene hydrochloride (**20b**).

Starting from compound **19b** (160 mg, 0.461 mmol) in Et_2_O (10 mL) and HCl/Et_2_O (389 µL, 2.4 M), the yield of compound **20b** was quantitative (176 mg, light orange crystals).

^1^H NMR spectrum of compound **20b** (300 MHz, DMSO-d_6_) δ, ppm: 11.90 (s, 1H, HCl), 7.86–7.74 (m, 2H, 2×CH), 7.66–7.51 (m, 3H, 3×CH), 7.08 (m, 2H, 2×CH), 4.61 (t, *J* = 5.0 Hz, 2H, 1×CH_2_), 3.99–3.88 (m, 4H, 2×CH_2_), 3.64–3.43 (m, 4H, 2×CH_2_), 3.21 (s, 2H, 1×CH_2_).

^13^C NMR spectrum of compound **20b** (75 MHz, DMSO-d_6_) δ, ppm: 160.0 (t, *J* = 14.4 Hz), 156.5 (dd, *J* = 257.7, 6.9 Hz), 152.7, 131.9, 129.5, 129.0 (t, *J* = 22.8 Hz), 122.3, 100.3 (d, *J* = 24.6 Hz), 63.6, 63.1, 54.3, 51.6.

MS (ESI^+^) *m*/*z*: [M−HCl+H]^+^, calculated for (C_18_H_20_F_2_N_3_O_2_)^+^ 348.1, found 348.1.

Synthesis of 2′-fluoro-4-(2-(*N*- morpholino)-ethoxy)-azobenzene hydrochloride (**20c**).

Starting from compound **19c** (249 mg, 0.756 mmol) in Et_2_O (14 mL) and HCl/Et_2_O (638 µL, 2.4 M), the yield of compound **20c** was quantitative (276 mg, light orange crystals).

^1^H NMR spectrum of compound **20c** (300 MHz, DMSO-d_6_) δ, ppm: 12.05 (s, 1H, HCl), 7.91 (m, 2H, 2×CH), 7.68 (m, 1H, 1×CH), 7.56 (m, 1H, 1×CH), 7.46 (m, 1H, 1×CH), 7.31 (m, 1H, 1×CH), 7.21 (m, 2H, 2×CH), 4.61 (m, 2H, 1×CH_2_), 3.93 (m, 4H, 2×CH_2_), 3.73–3.40 (m, 4H, 2×CH_2_), 3.25 (s, 2H, 1×CH_2_).

^13^C NMR spectrum of compound **20c** (75 MHz, DMSO-d_6_) δ, ppm: 160.6, 159.6 (d, *J* = 254.2 Hz), 146.9, 139.8 (d, *J* = 6.7 Hz), 132.8 (d, *J* = 8.3 Hz), 124.9, 117.4, 117.2 (d, *J* = 20.5 Hz), 115.4, 63.1, 62.8, 54.6, 51.6.

MS (ESI^+^) *m*/*z*: [M−HCl+H]^+^, calculated for (C_18_H_21_FN_3_O_2_)^+^ 330.2, found 330.2.

Synthesis of 2,2′-difluoro-4-(2-(*N*-morpholino)-ethoxy)-azobenzene hydrochloride (**20d**).

Starting from compound **19d** (392 mg, 1.128 mmol) in Et_2_O (10 mL) and HCl/Et_2_O (952 µL, 2.4 M), the yield of compound **20d** was quantitative (433 mg, light orange crystals).

^1^H NMR spectrum of compound **20d** (300 MHz, DMSO-d_6_) δ, ppm: 11.97 (s, 1H, HCl), 7.75 (t, *J* = 8.9 Hz, 1H, 1×CH), 7.66 (m, 1H, 1×CH), 7.58 (m, 1H, 1×CH), 7.47 (m, 1H, 1×CH), 7.33 (m, 1H, 1×CH), 7.23 (dd, *J* = 12.8, 2.6 Hz, 1H, 1×CH), 7.00 (dd, *J* = 9.2, 2.6 Hz, 1H, 1×CH), 4.62 (t, *J* = 4.9 Hz, 2H, 1×CH_2_), 3.99–3.84 (m, 4H, 2×CH_2_), 3.66–3.44 (m, 4H, 2×CH_2_), 3.23 (m, 2H, 1×CH_2_).

^13^C NMR spectrum of compound **20d** (75 MHz, DMSO-d_6_) δ, ppm: 162.0 (d, *J* = 11.3 Hz), 160.9 (d, *J* = 257.4 Hz), 159.1 (d, *J* = 255.7 Hz), 140.0 (d, *J* = 6.8 Hz), 134.7 (d, *J* = 6.9 Hz), 133.2 (d, *J* = 8.5 Hz), 125.0 (d, *J* = 3.8 Hz), 118.5 (d, *J* = 1.7 Hz), 117.5, 117.2 (d, *J* = 19.5 Hz), 112.3 (d, *J* = 2.7 Hz), 103.2 (d, *J* = 23.5 Hz), 63.2, 63.1, 54.4, 51.6.

MS (ESI^+^) *m*/*z*: [M−HCl+H]^+^, calculated for (C_18_H_20_F_2_N_3_O_2_)^+^ 348.1, found 348.2.

Synthesis of 2,2′,6-trifluoro-4-(2-(*N*-morpholino)-ethoxy)-azobenzene hydrochloride (**20e**).

Starting from compound **19e** (126 mg, 0.345 mmol) in Et_2_O (10 mL) and HCl/Et_2_O (291 µL, 2.4 M), the yield of compound **20e** was quantitative (138 mg, light orange crystals).

^1^H NMR spectrum of compound **20e** (300 MHz, DMSO-d_6_) δ, ppm: 12.04 (s, 1H, HCl), 7.61 (m, 2H, 2×CH), 7.48 (m, 1H, 1×CH), 7.33 (m, 1H, 1×CH), 7.09 (m, 2H, 2×CH), 4.63 (t, *J* = 4.9 Hz, 2H, 1×CH_2_), 4.01–3.89 (m, 4H, 2×CH_2_), 3.64–3.48 (m, 4H, 2×CH_2_), 3.22 (m, 2H, 1×CH_2_).

^13^C NMR spectrum of compound **20e** (75 MHz, DMSO-d_6_) δ, ppm: 160.5 (t, *J* = 14.6 Hz), 159.2 (d, *J* = 256.5 Hz), 156.7 (dd, *J* = 258.9, 7.2 Hz), 140.6 (d, *J* = 6.8 Hz), 133.8 (d, *J* = 8.5 Hz), 125.1 (d, *J* = 4.1 Hz), 117.4 (d, *J* = 19.5 Hz), 116.8, 100.4 (m), 63.7, 63.1, 54.3, 51.6.

MS (ESI^+^) *m*/*z*: [M−HCl+H]^+^, calculated for (C_18_H_19_F_3_N_3_O_2_)^+^ 366.1, found 366.1.

Synthesis of 2′,6′-difluoro-4-(2-(*N*-morpholino)-ethoxy)-azobenzene hydrochloride (**20f**).

Starting from compound **19f** (219 mg, 0.630 mmol) in Et_2_O (12 mL) and HCl/Et_2_O (532 µL, 2.4 M), the yield of compound **20f** was quantitative (241 mg, light orange crystals).

^1^H NMR spectrum of compound **20f** (300 MHz, DMSO-d_6_) δ, ppm: 12.00 (s, 1H, OH), 7.90 (m, 2H, 2×CH), 7.51 (m, 1H, 1×CH), 7.37–7.16 (m, 4H, 4×CH), 4.62 (m, 2H, 1×CH_2_), 3.93 (m, 4H, 2×CH_2_), 3.69–3.45 (m, 4H, 2×CH_2_), 3.24 (m, 2H, 1×CH_2_).

^13^C NMR spectrum of compound **20f** (75 MHz, DMSO-d_6_) δ, ppm: 161.0, 153.1 (dd, *J* = 255.9, 3.4 Hz), 147.3, 131.0 (t, *J* = 10.4 Hz), 130.5 (t, *J* = 10.6 Hz), 124.8, 115.5, 113.0 (d, *J* = 20.5 Hz), 63.2, 62.8, 54.6, 51.6.

MS (ESI^+^) *m*/*z*: [M−HCl+H]^+^, calculated for (C_18_H_20_F_2_N_3_O_2_)^+^ 348.1, found 348.2.

Synthesis of 2,2′,6′-trifluoro-4-(2-(*N*-morpholino)-ethoxy)-azobenzene hydrochloride (**20g**).

Starting from compound **19g** (273 mg, 0.747 mmol) in Et_2_O (15 mL) and HCl/Et_2_O (630 µL, 2.4 M), the yield of compound **20g** was quantitative (300 mg, light orange crystals).

^1^H NMR spectrum of compound **20g** (300 MHz, DMSO-d_6_) δ, ppm: 11.98 (s, 1H, HCl), 7.72 (t, *J* = 8.9 Hz, 1H, 1×CH), 7.64–7.46 (m, 1H, 1×CH), 7.37–7.20 (m, 3H, 3×CH), 7.01 (m, 1H, 1×CH), 4.63 (t, *J* = 4.9 Hz, 2H, 1×CH_2_), 4.02–3.83 (m, 4H, 2×CH_2_), 3.66–3.46 (m, 4H, 2×CH_2_), 3.22 (m, 2H, 1×CH_2_).

^13^C NMR spectrum of compound **20g** (75 MHz, DMSO-d_6_) δ, ppm: 162.61 (d, *J* = 11.4 Hz), 161.12 (d, *J* = 258.1 Hz), 154.9 (dd, *J* = 257.1, 4.2 Hz), 135.2 (d, *J* = 6.9 Hz), 131.6 (t, *J* = 10.5 Hz), 130.3 (t, *J* = 9.7 Hz), 118.0 (m), 113.1 (m), 112.5 (d, *J* = 2.9 Hz), 103.2 (d, *J* = 23.5 Hz), 63.4, 63.1, 54.5, 51.6.

MS (ESI^+^) *m*/*z*: [M−HCl+H]^+^, calculated for (C_18_H_19_F_3_N_3_O_2_)^+^ 366.1, found 366.1.

Synthesis of 2,2′,6,6′-tetrafluoro-4-(2-(*N*-morpholino)-ethoxy)-azobenzene hydrochloride (**20h**).

Starting from compound **19h** (96 mg, 0.250 mmol) in Et_2_O (7 mL) and HCl/Et_2_O (211 µL, 2.4 M), the yield of compound **20h** was quantitative (105 mg, light orange crystals).

^1^H NMR spectrum of compound **20h** (300 MHz, DMSO-d_6_) δ, ppm: 11.69 (s, 1H, HCl), 7.48–7.39 (m, 1H, 1×CH), 7.21 (m, 2H, 2×CH), 6.95 (m, 2H, 2×CH), 4.47 (t, *J* = 4.9 Hz, 2H, 1×CH_2_), 3.90 (m, 4H, 2×CH_2_), 3.46 (m, 4H, 2×CH_2_), 3.17 (m, 2H, 1×CH_2_).

^13^C NMR spectrum of compound **20h** (75 MHz, DMSO-d_6_) δ, ppm: 159.4 (t, *J* = 13.8 Hz), 151.9 (dd, *J* = 249.6, 8.5 Hz), 150.9 (dd, *J* = 250.9, 5.2 Hz), 132.2 (t, *J* = 11.0 Hz), 131.1 (t, *J* = 9.9 Hz), 125.4 (t, *J* = 17.2 Hz), 112.8 (dd, *J* = 20.3, 2.9 Hz), 99.8 (dd, *J* = 24.5, 2.9 Hz), 63.5, 63.1, 54.4, 51.6.

MS (ESI^+^) *m*/*z*: [M−HCl+H]^+^, calculated for (C_18_H_18_F_4_N_3_O_2_)^+^ 384.1, found 384.1.

#### 3.2.14. General Procedure for the Synthesis of 4-Hydroxyphenylazothiazoles (**22a**–**b**)

A concentrated aqueous solution of HCl was added to a solution of **21a**–**b** in water with stirring at 0–5 °C. The resulting mixture was stirred at 0–5 °C for 15 min. Subsequently, an aqueous solution of NaNO_2_ was added dropwise to the mixture over 10 min while maintaining the temperature at 0–5 °C, followed by stirring for an additional 30 min under cooling. A solution of phenol in 16% aqueous NaOH was then added dropwise to the diazonium salt solution over 10 min. The reaction mixture was stirred for 1 h at 0–5 °C, after which the pH was adjusted to 5. The precipitated product was filtered and dried. The products were purified by column chromatography.

Synthesis of 2-(4-hydroxyphenylazo)-thiazole (**22a**).

Starting from 2-aminothiazole (**21a**) (5.400 g, 53.924 mmol) dissolved in 30 mL of water, HCl (36.6%, 16 mL), NaNO_2_ (3.720 g, 53.921 mmol) in 18 mL of water, and a solution of phenol (5.075 g, 53.926 mmol) in 16% aqueous NaOH (40 mL), the reaction product was purified by chromatography (SiO_2_, EA/MeOH = 50:1). The yield of compound **22a** was 38% (4.205 g, brown crystals).

^1^H NMR spectrum of compound **22a** (300 MHz, DMSO-d_6_) δ, ppm: 10.72 (s, 1H, -OH), 8.04 (d, *J* = 3.4 Hz, 1H, 1×CH), 7.86 (m, 2H, 2×CH), 7.81 (d, *J* = 3.4 Hz, 1H, 1×CH), 6.98 (m, 2H, 2×CH).

^13^C NMR spectrum of compound **22a** (75 MHz, DMSO-d_6_) δ, ppm: 176.8, 162.9, 144.3, 143.7, 126.2, 121.9, 116.5.

MS (ESI^+^) *m*/*z*: [M+H]^+^, calculated for (C_9_H_8_N_3_OS)^+^ 206.0, found 206.0.

Synthesis of 2-(4-hydroxyphenylazo)-5-methylthiazole (**22b**).

Starting from 2-amino-5-methylthiazole (**21b**) (500 mg, 4.379 mmol) dissolved in 5 mL of water, HCl (36.6%, 5 mL), NaNO_2_ (302 mg, 4.377 mmol) in water (5 mL), and a solution of phenol (412 mg, 4.378 mmol) in 16% aqueous NaOH (15 mL), the reaction product was purified by chromatography (SiO_2_, EA/MeOH = 50:1). The yield of compound **22b** was 83% (796 mg, brown crystals).

^1^H NMR spectrum of compound **22b** (300 MHz, DMSO-d_6_) δ, ppm: 10.65 (s, 1H, -OH), 7.81 (m, 2H, 2×CH), 7.76 (m, 1H, 1×CH), 6.97 (m, 2H, 2×CH), 2.49 (s, 3H, 1×CH_3_).

^13^C NMR spectrum of compound **22b** (75 MHz, DMSO-d_6_) δ, ppm: 174.7, 162.6, 144.3, 141.8, 136.4, 126.0, 116.5, 12.5.

MS (ESI^+^) *m*/*z*: [M+H]^+^ calculated for (C_10_H_10_N_3_OS)^+^ 220.0, found 220.0.

#### 3.2.15. General Procedure for the Preparation of Compounds **23a**–**b** and **24a**–**b**

To a solution of compound **22a**–**b** in DMF, K_2_CO_3_, *N*-(2-chloroethyl)-morpholine hydrochloride and KI were added under stirring. The reaction mixture was heated under reflux with stirring for 3 h, followed by extraction with ethyl acetate from water. The organic layer was washed with brine and dried over anhydrous Na_2_SO_4_. The solvent was removed under reduced pressure. The products were purified by column chromatography.

Preparation of 2-(4-(2-(*N*-morpholino)-ethoxy)-phenylazo)-thiazole (**23a**) and 4-((3-(2-(*N*-morpholino)-ethyl)-thiazol-2(3*H*)-ylidene)-hydrazono)-cyclohexa-2,5-dien-1-one (**24a**).

Starting from compound **22a** (1000 mg, 4.873 mmol) in DMF (30 mL), K_2_CO_3_ (2022 mg, 14.631 mmol), *N*-(2-chloroethyl)-morpholine hydrochloride (1361 mg, 7.314 mmol), and KI (810 mg, 4.879 mmol) after purification by chromatography (SiO_2_, EA/MeOH = 5:1), the yield of compound **23a** was 19% (295 mg, orange crystals), and the yield of compound **24a** was 5% (78 mg, violet amorphous solid).

^1^H NMR spectrum of compound **23a** (600 MHz, DMSO-d_6_) δ, ppm: 8.08 (d, *J* = 3.3 Hz, 1H, 1×CH), 7.94 (m, 2H, 2×CH), 7.86 (d, *J* = 3.3 Hz, 1H, 1×CH), 7.18 (m, 2H, 2×CH), 4.24 (t, *J* = 5.7 Hz, 2H, 1×CH_2_), 3.58 (m, 4H, 2×CH_2_), 2.73 (t, *J* = 5.7 Hz, 2H, 1×CH_2_), 2.48 (m, 4H, 2×CH_2_).

^13^C NMR spectrum of compound **23a** (150 MHz, DMSO-d_6_) δ, ppm: 176.5, 162.8, 145.2, 143.8, 125.7, 122.2, 115.6, 66.1, 66.0, 56.8, 53.5.

MS (ESI^+^) *m*/*z*: [M+H]^+^, calculated for (C_15_H_19_N_4_O_2_S)^+^ 319.1, found 319.0.

^1^H NMR spectrum of compound **24a** (600 MHz, DMSO-d_6_) δ, ppm: 7.95 (m, 1H), 7.53 (d, *J* = 4.5 Hz, 1H), 7.34 (m, 1H), 6.98 (d, *J* = 4.5 Hz, 1H), 6.37 (m, 2H), 4.28 (t, *J* = 6.2 Hz, 2H, 1×CH_2_), 3.50 (m, 4H, 2×CH_2_), 2.70 (t, *J* = 6.2 Hz, 2H, 1×CH_2_), 2.45 (m, 4H, 2×CH_2_).

^13^C NMR spectrum of compound **24a** (150 MHz, DMSO-d_6_) δ, ppm: 186.5, 174.2, 145.5, 139.9, 131.1, 128.9, 126.9, 124.8, 107.8, 66.1, 56.2, 53.1, 44.9.

MS (ESI^+^) *m*/*z*: [M+H]^+^, calculated for (C_15_H_19_N_4_O_2_S)^+^ 319.1, found 319.0.

Preparation of 2-(4-(2-(*N*-morpholino)-ethoxy)-phenylazo)-5-methylthiazole (**23b**) and 4-((5-methyl-3-(2-(*N*-morpholino)-ethyl)-thiazol-2(3*H*)-ylidene)-hydrazono)-cyclohexa-2,5-dien-1-one (**24b**).

Starting from compound **22b** (144 mg, 0.657 mmol) in DMF (5 mL), K_2_CO_3_ (272 mg, 1.968 mmol), *N*-(2-chloroethyl)-morpholine hydrochloride (183 mg, 0.983 mmol), and KI (109 mg, 0.657 mmol) were used. After purification by chromatography (SiO_2_, DCM/MeOH/NH_4_OH = 200:10:1), the yield of compound **23b** was 62% (135 mg, orange crystals), and the yield of compound **24b** was 5% (11 mg, violet amorphous solid).

^1^H NMR spectrum of compound **23b** (600 MHz, DMSO-d_6_) δ, ppm: 7.89 (m, 2H, 2×CH), 7.80 (m, 1H, 1×CH), 7.17 (m, 2H, 2×CH), 4.23 (t, *J* = 5.7 Hz, 2H, 1×CH_2_), 3.58 (m, 4H, 2×CH_2_), 2.73 (t, *J* = 5.7 Hz, 2H, 1×CH_2_), 2.51 (s, 3H, 1×CH_3_), 2.48 (m, 4H, 2×CH_2_).

^13^C NMR spectrum of compound **23b** (150 MHz, DMSO-d_6_) δ, ppm: 174.4, 162.5, 145.2, 142.0, 136.9, 125.5, 115.5, 66.1, 66.0, 56.8, 53.5, 12.4.

MS (ESI^+^) *m*/*z*: [M+H]^+^, calculated for (C_16_H_21_N_4_O_2_S)^+^ 333.1, found 333.1.

^1^H NMR spectrum of compound **24b** (600 MHz, DMSO-d_6_) δ, ppm: 7.92 (m, 1H, 1×CH), 7.31 (m, 1H, 1×CH), 7.29 (m, 1H, 1×CH), 6.35 (m, 2H, 2×CH), 4.23 (t, *J* = 6.2 Hz, 2H, 1×CH_2_), 3.50 (m, 4H, 2×CH_2_), 2.68 (t, *J* = 6.2 Hz, 2H, 1×CH_2_), 2.45 (m, 4H, 2×CH_2_), 2.26 (d, *J* = 1.4 Hz, 3H, 1×CH_3_).

^13^C NMR spectrum of compound **24b** (150 MHz, DMSO-d_6_) δ, ppm: 186.4, 173.6, 145.2, 139.9, 128.8, 127.4, 126.8, 124.7, 120.1, 66.2, 56.4, 53.1, 44.7, 12.7

MS (ESI^+^) *m*/*z*: [M+H]^+^, calculated for (C_16_H_21_N_4_O_2_S)^+^ 333.1, found 333.1.

#### 3.2.16. General Procedure for the Preparation of Compounds **25a**–**b**

To the solutions of compounds **23a**–**b** in Et_2_O, a solution of HCl/Et_2_O (2.4 M) was added dropwise with stirring at 0–5 °C. The resulting mixture was stirred at RT for 30 min. The product was concentrated under reduced pressure.

Synthesis of 2-(4-(2-(*N*-morpholino)-ethoxy)-phenylazo)-thiazole hydrochloride (**25a**).

Starting from compound **23a** (80 mg, 0.251 mmol) in Et_2_O (10 mL) and HCl/Et_2_O (500 μL, 2.4 M), compound **25a** was obtained quantitatively (89 mg, light-orange crystals).

^1^H NMR spectrum of compound **25a** (300 MHz, DMSO-d_6_) δ, ppm: 11.59 (s, 1H, HCl), 8.10 (d, *J* = 3.3 Hz, 1H, 1×CH), 7.99 (m, 2H, 2×CH), 7.90 (d, *J* = 3.3 Hz, 1H, 1×CH), 7.25 (m, 2H, 2×CH), 4.61 (t, *J* = 5.0 Hz, 2H, 1×CH_2_), 4.01–3.70 (m, 6H, 3×CH_2_), 3.64–3.46 (m, 4H, 2×CH_2_).

^13^C NMR spectrum of compound **25a** (75 MHz, DMSO-d_6_) δ, ppm: 176.4, 161.7, 145.7, 144.0, 125.8, 122.6, 115.8, 63.1, 62.8, 54.6, 51.6.

MS (ESI^+^) *m*/*z*: [M−HCl+H]^+^ calculated for (C_15_H_19_N_4_O_2_S)^+^ 319.1, found 319.1.

Synthesis of 2-(4-(2-(*N*-morpholino)-ethoxy)-phenylazo)-5-methylthiazole hydrochloride (**25b**).

Starting from compound **23b** (100 mg, 0.301 mmol) in Et_2_O (20 mL) and HCl/Et_2_O (400 μL, 2.4 M), compound **25b** was obtained quantitatively (110 mg, light-orange crystals).

^1^H NMR spectrum of compound **25b** (300 MHz, DMSO-d_6_) δ, ppm: 11.29 (s, 1H, HCl), 7.95 (m, 2H, 2×CH), 7.83 (d, *J* = 1.2 Hz, 1H, 1×CH), 7.23 (m, 2H, 2×CH), 4.58 (t, *J* = 4.9 Hz, 2H, 1×CH_2_), 4.04–3.74 (m, 4H, 2×CH_2_), 3.55 (m, 4H, 2×CH_2_), 3.22 (m, 2H, 1×CH_2_), 2.52 (m, 3H, 1×CH_3_).

^13^C NMR spectrum of compound **25b** (75 MHz, DMSO-d_6_) δ, ppm: 161.4, 145.8, 142.2, 137.2, 125.5, 122.5, 115.8, 63.1, 62.7, 54.7, 51.7, 12.4.

MS (ESI^+^) *m*/*z*: [M−HCl+H]^+^, calculated for (C_16_H_21_N_4_O_2_S)^+^ 333.1, found 333.1.

#### 3.2.17. Preparation of Micellar Solutions of Compounds

A solution of corresponding compound (50 mg) in dichloromethane (2 mL) was added dropwise to a freshly prepared 4% aqueous solution of Kolliphor^®^ ELP (5 mL) heated to 45 °C with continuous argon bubbling. Bubbling was continued until strong foaming started. A clear orange solution with a concentration of 1.0% was obtained, which was subsequently filtered in turn through a 0.45 µm PTFE filter and then through a 0.22 µm PTFE filter.

### 3.3. Photophysical Property Investigations

Z-E isomerization half-life was determined using UV/Vis spectroscopy. Solutions were prepared and kept in the dark at RT before the experiment. First, the spectrum in the dark was registered (${PSS}_{dark})$, which corresponds to the photostationary state (PSS) with *E*-isomer predominance. Then, the cuvette with the solution was irradiated with UV light (λ = 365 nm) for 5 min to give the PSS with Z-isomer predominance. The corresponding spectrum was registered (${PSS}_{365nm})$. During the subsequent isomerization, the kinetics of changes in the absorption value were recorded at the wavelength of the absorption maximum corresponding to the *E*-isomer ($\lambda_{\max\left(E\right)})$. Then, $\ln\frac{A_{t}}{A_{0}}$ values were calculated using the Equation (1):

(1) $$\ln\frac{A_{t}}{A_{0}}=\ln\frac{{Abs}_{PSS(dark)}-{Abs}_{time}}{{Abs}_{PSS(dark)}-{Abs}_{PSS(365nm)}},$$

where ${Abs}_{PSS(dark)}$, ${Abs}_{PSS(365nm)},$ and ${Abs}_{time}$ are absorbance values at $\lambda_{\max\left(E\right)}$ in the dark at the beginning of the experiment, after UV light (λ = 365 nm) irradiation, and at different time points from 0 to 60 min, respectively. Then, a graph was plotted depending on $\ln\frac{A_{t}}{A_{0}}$ values from time, the trend line was calculated, and a first-order rate constant (*k*) for the thermal back Z-E isomerization reaction was obtained as the slope of the trend line.

For a first-order reaction, half-life $t_{1/2}$ can be calculated using the Equation (2):

(2) $$t_{1/2}=\frac{\ln2}{k}$$

### 3.4. Biology

All manipulations with animals were approved by the Committee of National Medical Research Radiological Centre of the Ministry of Health of the Russian Federation for bioethical control over the maintenance and use of laboratory animals for scientific purposes (Minutes №12 dated 11 March 2024) and performed in accordance with the national and international rules for the humane treatment of animals (European Convention for the Protection of Vertebrate Animals Used for Experimental and Other Scientific Purposes, Council of Europe (ETS 123), Eighth Edition of the Guide for the Care and Use of Laboratory Animals (NRC 2011)) [36]. All materials, methods, and experimental procedures where animals were used are described in accordance with ARRIVE rules [37].

#### 3.4.1. Cytotoxicity Determination In Vitro

The culture of dermal fibroblast cells (DF2) was obtained from the collection of the Institute of Cytology of the Russian Academy of Sciences (Saint Petersburg, Russia). The cells were cultured in plastic vials with a cell growth surface of 25 cm^2^ (Costar, Richmond, VA, USA) on DMEM/F12 medium with the addition of L-glutamine and 10% fetal bovine serum (FBS) (PanEco, Moscow, Russia). The cells were cultured at 37 °C in a humid atmosphere with a content of 5% CO_2_ (Binder CO_2_ incubator, Germany). Cell lines from 3 to 7 passages were used in the work. To conduct experiments to evaluate the cytotoxicity of compounds, cells were sieved into 96-well culture plates at a concentration of 10^5^ cells per ml, incubated at 37 °C (humidified atmosphere, 5% carbon dioxide) for 24–28 h. Further, 50 µL of the studied substances was added. The cells were incubated with the compounds for 24 h at 37 °C. Next, the cytotoxic effect of the substances was evaluated by a colorimetric method using an MTT test and visually, using an inverted microscope. The criterion for evaluating the specific activity of the compound was the value of IC50, the concentration at which 50% death of culture cells was noted.

#### 3.4.2. Preparation of Animals and Arrangement of Groups

For determination of the rabbit eye cornea’s sensitivity using the surface anesthesia method, 12 rabbits with a basic sensitivity threshold (sensitivity threshold of the eye cornea to tactile impact of the anesthesiometer) of 1–2 touches were selected. They were used in experiments repeatedly with an interval of at least 5 days between the experiments. Each eye was considered as a separate experimental point. Three animals were used in each of the experimental groups (*n* = 6). Two animals (*n* = 4) were used in each of the negative control groups and in each of experimental groups with UV irradiation due to the expected lack of the effect and to minimize the number of animals in the experiment. Animals were randomly divided into groups. Before an experiment, the animals were immobilized, leaving the head free. Before each experiment, the animals’ eyelashes were cut out, and the basic threshold of sensitivity of the eye cornea to tactile contact was determined using an anesthesiometer.

#### 3.4.3. Irradiation Modes

The irradiation modes were described earlier [23,25]. Experiments with animals were carried out in a darkened room with a 50W LED light source (λ = 625 nm), which lies outside the absorption bands of the UV/Vis spectrum of the test compound. Before the experiments, solutions of test compounds were irradiated for 5 min with UV light (λ = 365 nm, LED flashlight, 0.5 W).

#### 3.4.4. Determination of the Rabbit Eye Cornea’s Sensitivity Using the Surface Anesthesia Method (Adapted from the Regnier Method)

The adaptation of a model of surface anesthesia on the rabbit’s cornea using the Regnier method [38] for the study of light-controlled local anesthetics was described earlier [23,25]. The test solution (0.2 mL) of the required concentration (2% for the lidocaine or 1% for the ethercaine and its derivatives) was instilled into each conjunctival sac. After that, the sensitivity threshold of the eye cornea to tactile impact was determined. The first determination was performed at the 8th minute of the experiment and then at the 10th, 12th, 15th, 20th, 25th, 30th, 35th, 40th, 45th, 50th, 55th, and 60th min (13 values overall). Each time, the minimum number of touches of the same strength and rhythm causing the eyelids to close (but no more than 100) was recorded. The Regnier index was calculated as the total of 13 values within 60 min using the Equation (3):

(3) $$\mathrm{Regnier}\;\mathrm{index}\;\left(RI\right)=\sum_{i=1}^{13}n_{i}$$

where *n* is the number of touches before the eyelids closed.

## 4. Conclusions

In this work, a series of light-controlled, local anesthetic, ethercaine derivatives were synthesized to investigate the influence of structure modification on physicochemical and biological properties. Previously, we established that replacing the ether linker in the ethercaine molecule with a linker containing an amide bond leads to a slight decrease in local anesthetic activity in a surface anesthesia model.

Among the regioisomers of ethercaine, the local anesthetic activity decreases depending on the position of the linker on the benzene ring in the following order: para > meta > ortho, which can be explained by the greater affinity of the active site for elongated molecules than for bent ones like the ortho-substituted derivative or Z-isomers. Meanwhile, the ortho- and meta-isomers exhibit an increased Z-E isomerization half-life, with this value being significantly higher in aqueous micellar solutions compared to DMSO. Substituting the morpholine ring with *N*,*N*-diethylamine and *N*-methylpiperazine fragments reduces the difference in local anesthetic activity observed with and without irradiation. The significant influence of nitrogen-containing moieties on local anesthetic activity in the Z-form may be exploited in the future for the development of Z-active derivatives.

Introducing fluorine atoms into the ortho-positions of azobenzene increases the stability of the Z-isomer; however, an increase in the number of fluorine atoms results in reduced local anesthetic activity. It was noted that fluorine atoms in the two and six positions of azobenzene in ethercaine contribute more to the increase in the Z-E isomerization half-life than those in the 2′ and 6′ positions. A possible explanation is the greater sensitivity of the active site of the ion channel to changes in the lower ethercaine cycle than in the upper one.

Ethercaine derivatives based on phenylazothiazole demonstrate improved water solubility and a bathochromic shift in the absorption maximum in the electronic spectrum, making these heterocyclic analogs of azobenzene promising candidates for further development of light-controlled compounds. The results obtained indicate that the development of new photopharmacological agents based on heteroazoaromatic compounds allows for a more comprehensive optimization of the properties of the target compounds.

## Figures and Tables

**Table 1:** Studies of the properties of compounds **1**, **2**, **9a**–**b**.

Compound	τ_1_/_2_ (DMSO)	τ_1_/_2_ (Aqueous Kolliphor ELP)	In Vitro Cytotoxicity (mM, DF2)	Local Anesthetic Activity (Regnier Index, Min—13, Max—1300)	
				In the Dark	Under Irradiation (λ = 365 nm)
Lidocaine 2%	-	-	>5	451 ± 40 (*n* = 8) *	469 ± 37 (*n* = 8)
Ethercaine hydrochloride (**1**)	2.1 h	50 min	0.21	644 ± 30 (*n* = 8)	33 ± 15 (*n* = 8)
**2**	30.5 h	31 min	>0.2	512 ± 56 (*n* = 8)	33 ± 6 (*n* = 8)
**9a**	17.2 h	5.2 d	>1	390 ± 39 (*n* = 6)	46 ± 10 (*n* = 4)
**9b**	26.8 h	18.2 d	0.24	503 ± 85 (*n* = 6)	98 ± 33 (*n* = 4)

* Note: All local anesthetic activity data are expressed as the mean ± SD.

**Table 2:** Studies of the properties of compounds **14** and **16**.

Compound	τ_1_/_2_ (DMSO)	τ_1_/_2_ (Aqueous Kolliphor ELP)	In Vitro Cytotoxicity (mM, DF2)	Local Anesthetic Activity (Regnier Index, Min—13, Max—1300)	
				In the Dark	Under Irradiation (λ = 365 nm)
**14**	18.6 h	3.5 h **	0.06	556 ± 48 (*n* = 6) *^,^ **	510 ± 71 (*n* = 4) **
**16**	15.6 h	6.0 h	0.09	470 ± 15 (*n* = 6)	168 ± 73 (*n* = 4)

* Note: All local anesthetic activity data are expressed as the mean ± SD. ** Value obtained for the solution in water.

**Table 3:** Results of the study on the properties of compounds **20a**–**h**.

Compound	R_1_	R_2_	R_3_	R_4_	τ_1_/_2_ (DMSO)	τ_1_/_2_ (Aqueous Kolliphor ELP)	In Vitro Cytotoxicity (mM, DF2)	Local Anesthetic Activity (Regnier Index, Min—13, Max—1300)	
								In the Dark	Under Irradiation (λ = 365 nm)
**20a**	H	H	F	H	7.8 h	2.5 h	0.19	509 ± 34 (*n* = 6) *	64 ± 15 (*n* = 4)
**20b**	H	H	F	F	19.0 d	1.9 h	0.18	538 ± 37 (*n* = 6)	58 ± 8 (*n* = 4)
**20c**	F	H	H	H	3.6 h	24 min	0.19	590 ± 24 (*n* = 6)	70 ± 7 (*n* = 4)
**20d**	F	H	F	H	8.1 h	2.3 h	0.19	419 ± 57 (*n* = 6)	84 ± 8 (*n* = 4)
**20e**	F	H	F	F	29.3 d	2.5 d	0.19	395 ± 72 (*n* = 6)	59 ± 18 (*n* = 4)
**20f**	F	F	H	H	1.0 d	1.0 h	0.21	570 ± 87 (*n* = 6)	76 ± 19 (*n* = 4)
**20g**	F	F	F	H	5.7 d	5.0 h	0.18	437 ± 37 (*n* = 6)	45 ± 9 (*n* = 4)
**20h**	F	F	F	F	49.6 d	11.7 d	0.44	353 ± 10 (*n* = 6)	35 ± 7 (*n* = 4)

* Note: All local anesthetic activity data are expressed as the mean ± SD.

**Table 4:** Results of the study of the properties of compounds **25a**–**b**.

Compound	τ_1_/_2_ (DMSO)	τ_1_/_2_ (Aqueous Kolliphor ELP)	In Vitro Cytotoxicity (mM, DF2)	Local Anesthetic Activity (Regnier Index, Min—13, Max—1300)	
				In the dark	Under Irradiation (λ = 365 nm)
**25a**	12 min	13 min	0.20	342 ± 21 (*n* = 6) *	25 ± 2 (*n* = 4)
**25b**	5 min	4 min	0.44	529 ± 23 (*n* = 6)	42 ± 4 (*n* = 4)

* Note: All local anesthetic activity data are expressed as the mean ± SD.

## Data Availability

The original contributions presented in this study are included in the article and the Supplementary Materials. Further inquiries can be directed to the corresponding author.

## Supplementary Materials

The following supporting information can be downloaded at https://www.mdpi.com/article/10.3390/ijms26073244/s1.

## Abbreviations

The following abbreviations are used in this manuscript:

RI	Regnier index
UV	Ultraviolet
DMSO	Dimethyl sulfoxide
ACN	Acetonitrile
SD	Standard deviation
TLC	Thin layer chromatography
NMR	Nuclear magnetic resonance
