# Supplementary material for: Structure–Property Relationships in Novel Series of Photoswitchable Local Anesthetic Ethercaine Derivatives: Emphasis on Biological and Photophysical Properties
Source: Int J Mol Sci. 2025 Mar 31;26(7):3244. doi: 10.3390/ijms26073244 (PMC11989239; doi:10.3390/ijms26073244)
Supplement: Supplementary file 1 [file ijms-26-03244-s001.zip › ijms-3494039-supplementary.pdf]

Title: Structure-property relationships in novel series of photoswitchable local anesthetic ethercaine derivatives: emphasis on biological and photophysical properties

Authors: A. Noev, D. Likhobabina, J. Sutemieva, A. Plyutinskaya, D. Cheshkov, N. Morozova, A. Vinokurova, Y. Vasil'ev, N. Suvorov, E. Nemtzova, A. Pankratov, E. Filonenko, P. Shegay, A. Kaprin and M. Grin

Supplementary information

1. NMR and MS data

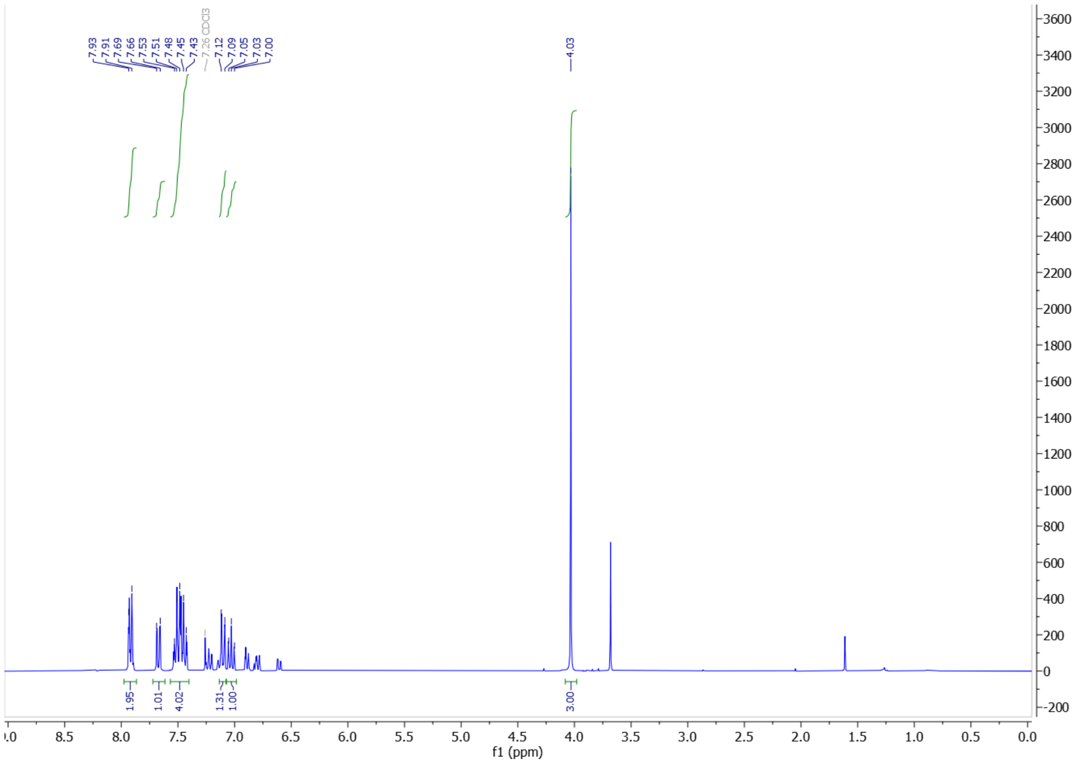

*Figure 1. ^{1}H NMR (300 MHz, $CDCl_3$) spectrum of compound **6a***

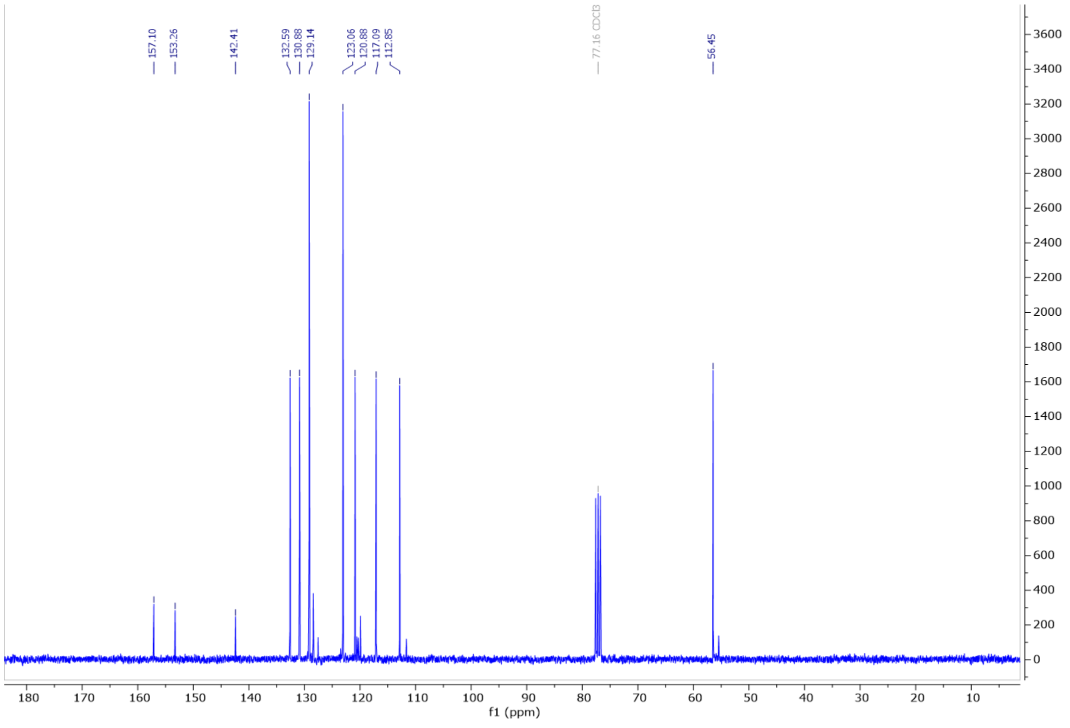

*Figure 2. ^{13}C NMR (75 MHz, $CDCl_3$) spectrum of compound 6**a***

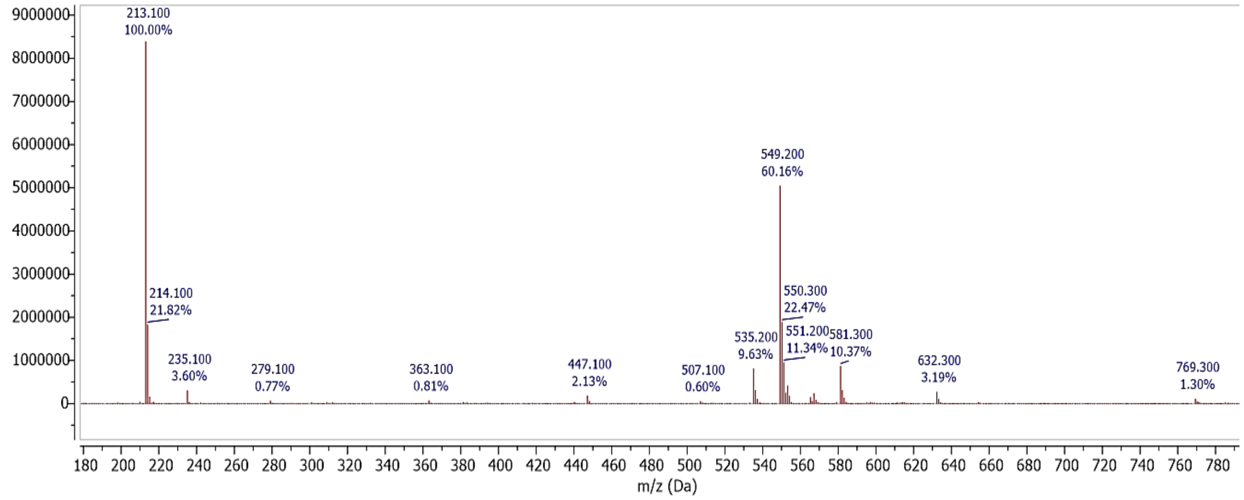

*Figure 3. Mass spectrum ESI$^+$ of compound 6**a***

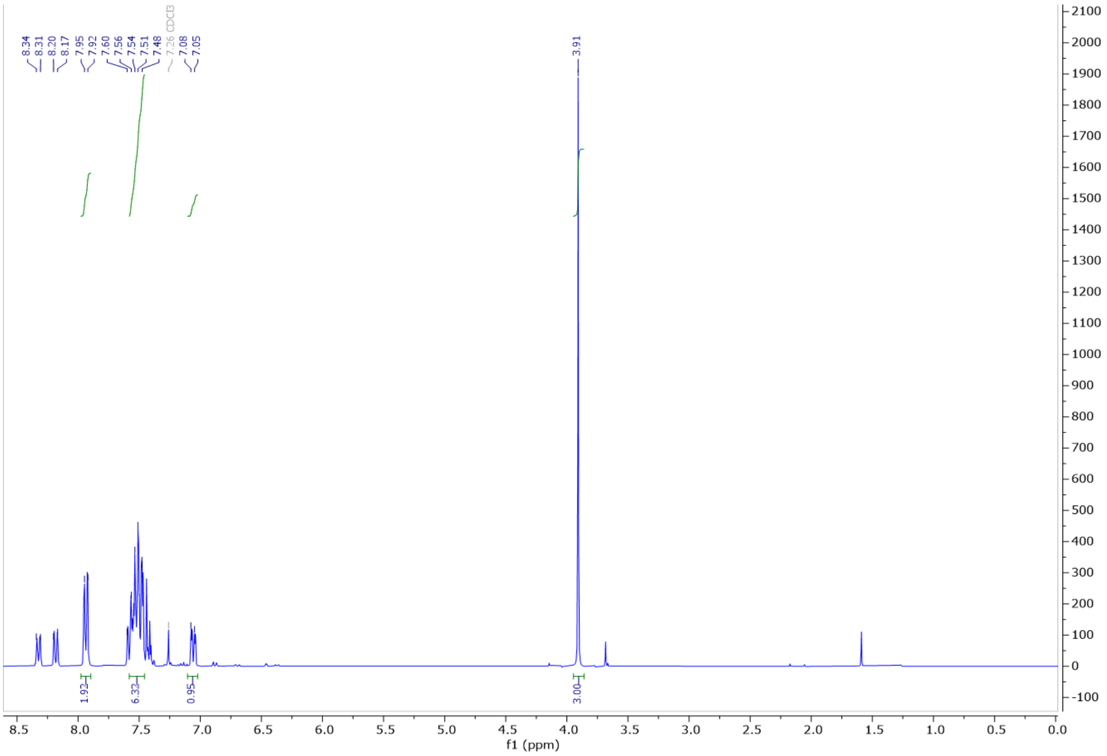

*Figure 4. ^1H NMR (300 MHz, $CDCl_3$) spectrum of compound **6b***

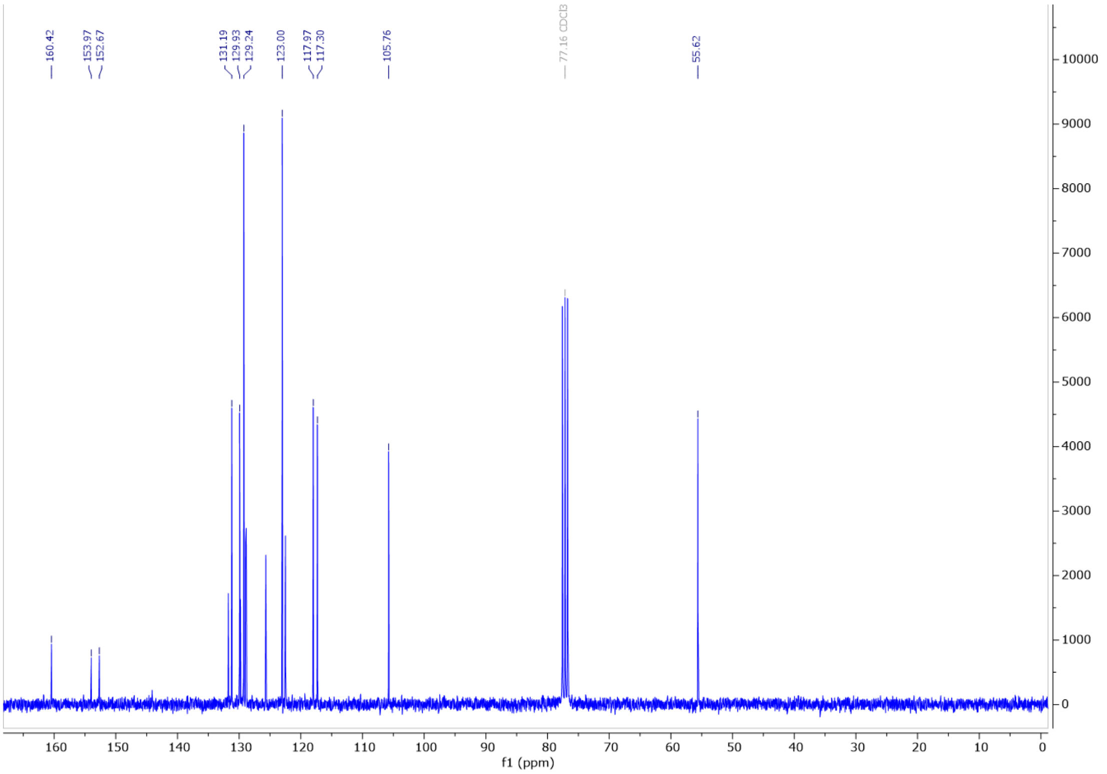

Figure 5. ^{13}C *NMR (75 MHz,* $CDCl_3$*) spectrum of compound* ***6b***

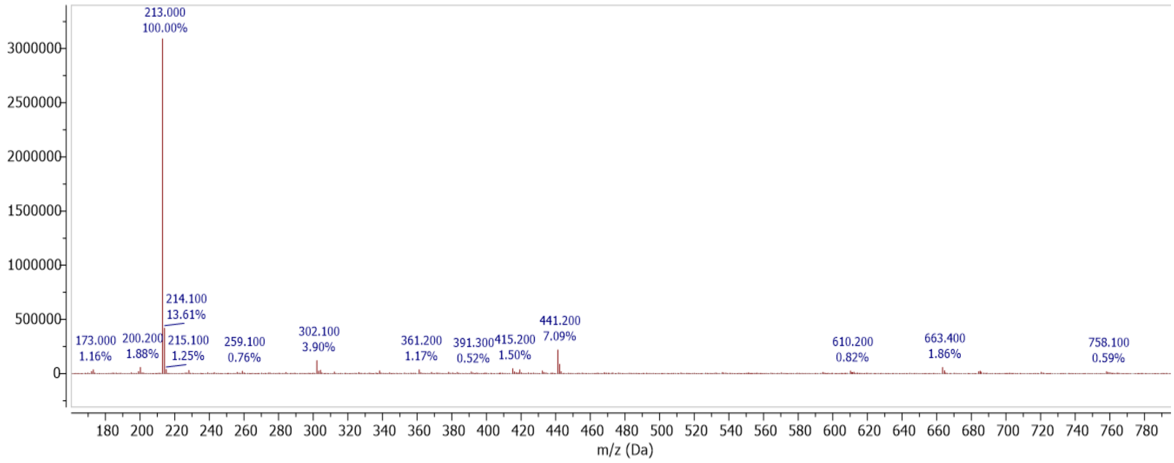

Figure 6. Mass spectrum ESI^+ *of compound* ***6b***

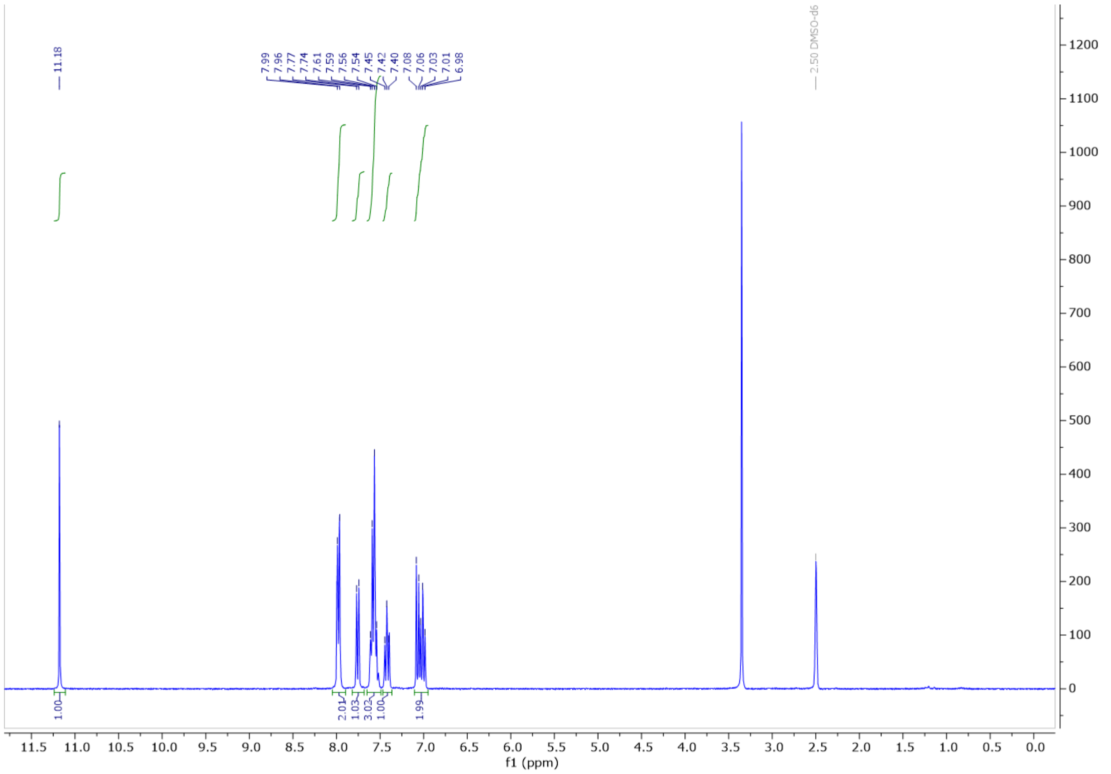

Figure 7. ^{1}H NMR (300 MHz, DMSO-d6) spectrum of compound ***7a***

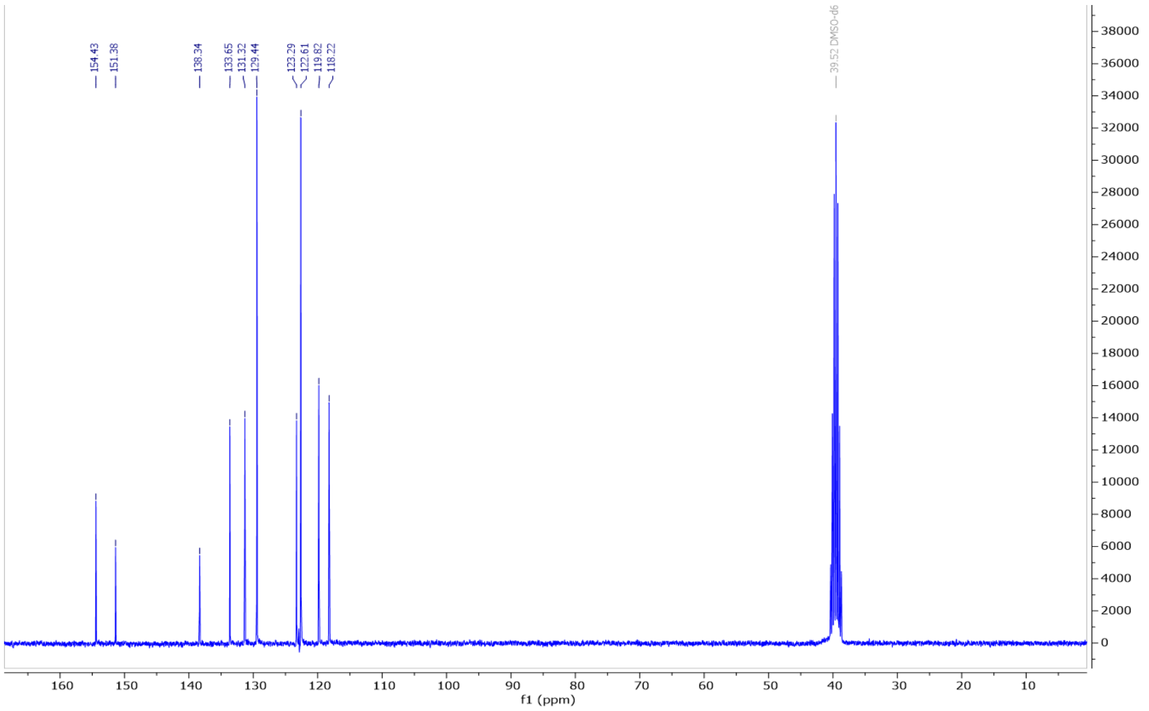

Figure 8. ^{13}C NMR (75 MHz, DMSO-d6) spectrum of compound ***7a***

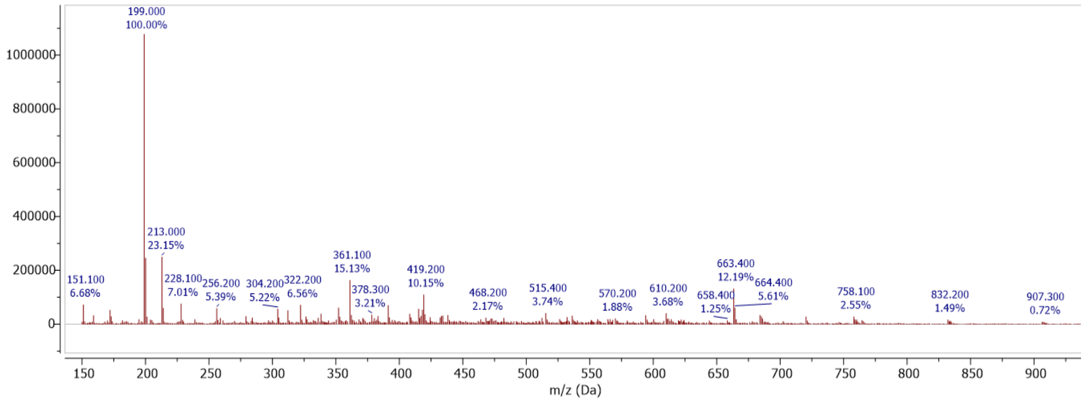

Figure 9. Mass spectrum ESI^{+} of compound ***7a***

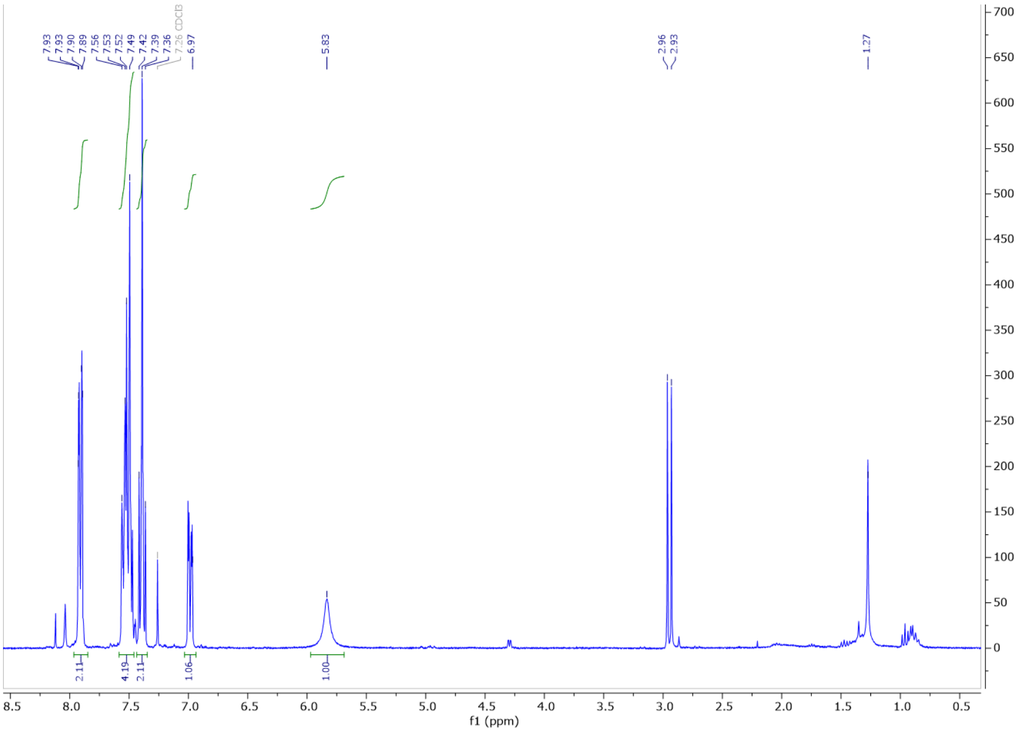

Figure 10. ^{1}H NMR (300 MHz, $CDCl_3$) spectrum of compound ***7b.***

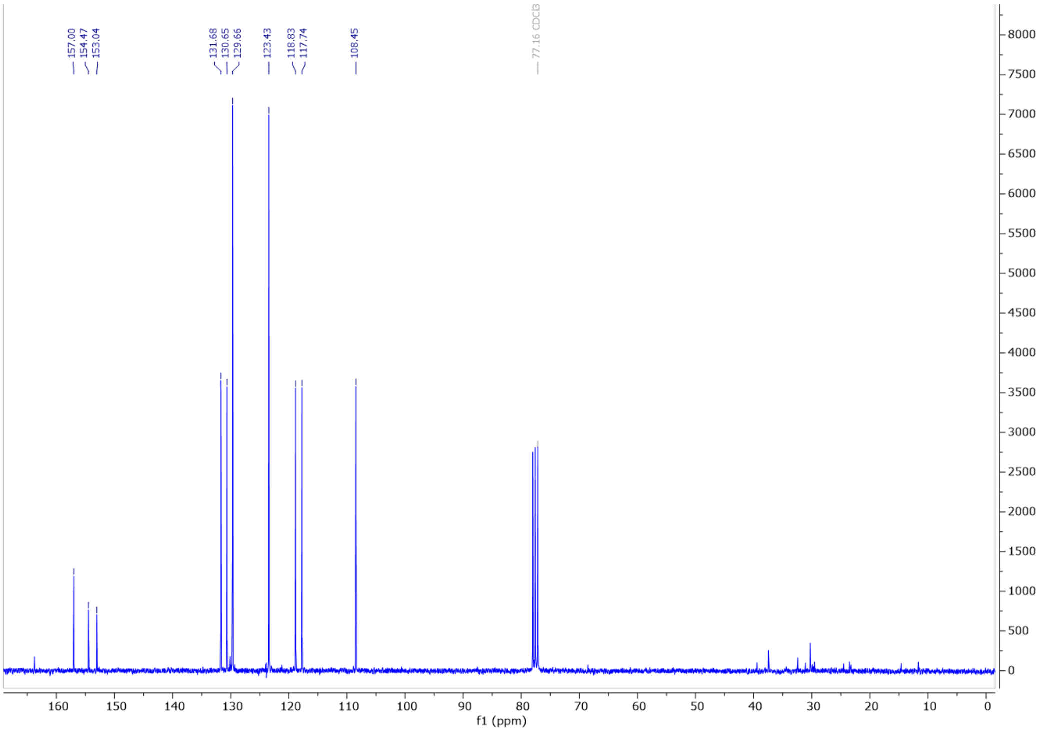

Figure 11. ^{13}C NMR (75 MHz, $CDCl_3$) spectrum of compound ***7b***

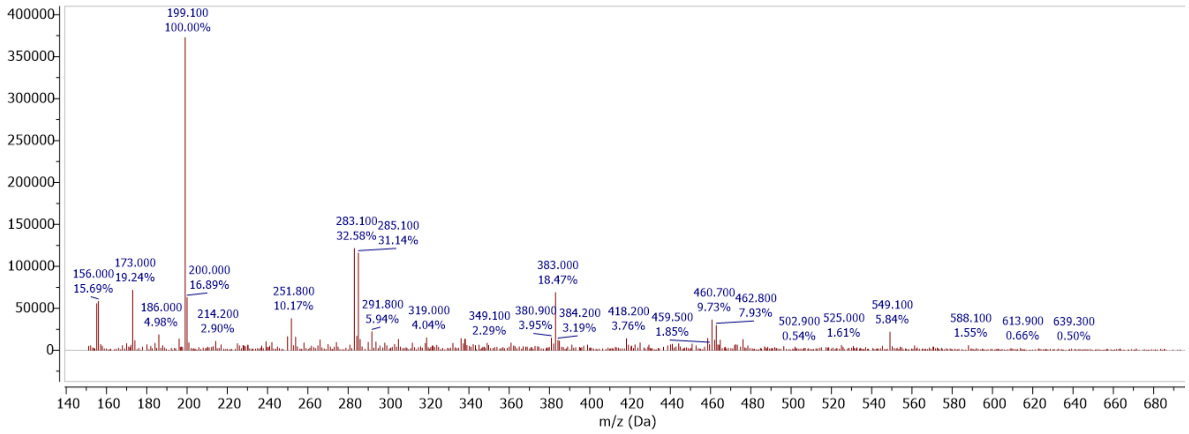

Figure 12. Mass spectrum ESI^{+} *of compound* ***7b***

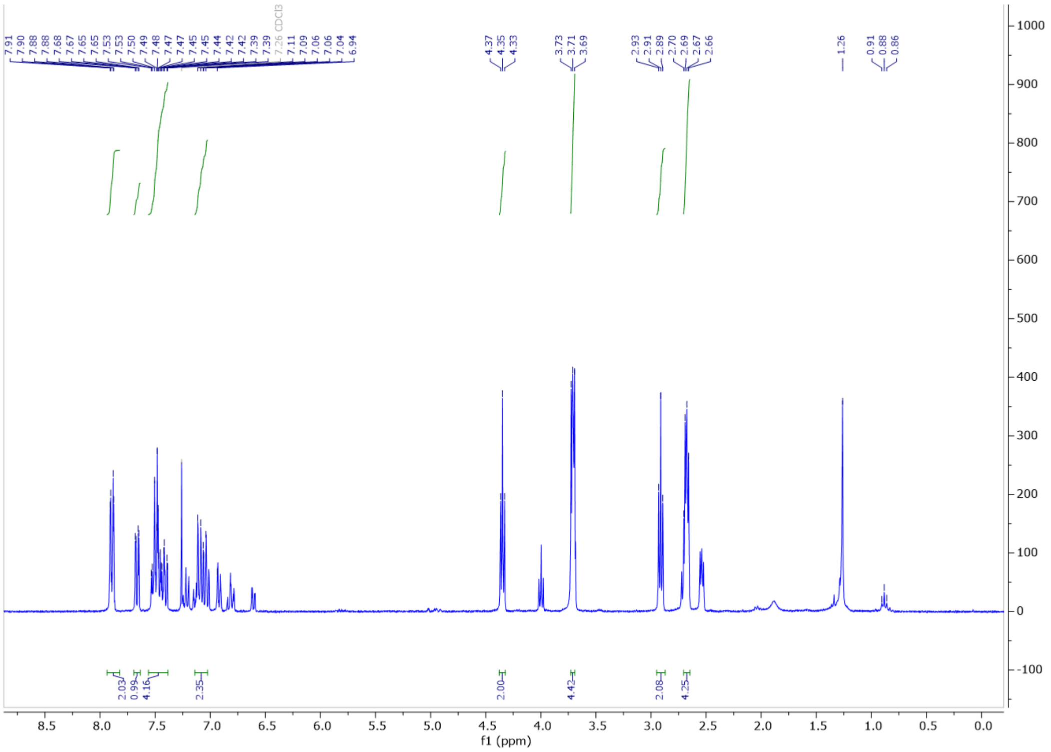

Figure 13. ^{1}*H NMR (300 MHz, CDCl*$_3$*) spectrum of compound* ***8a***

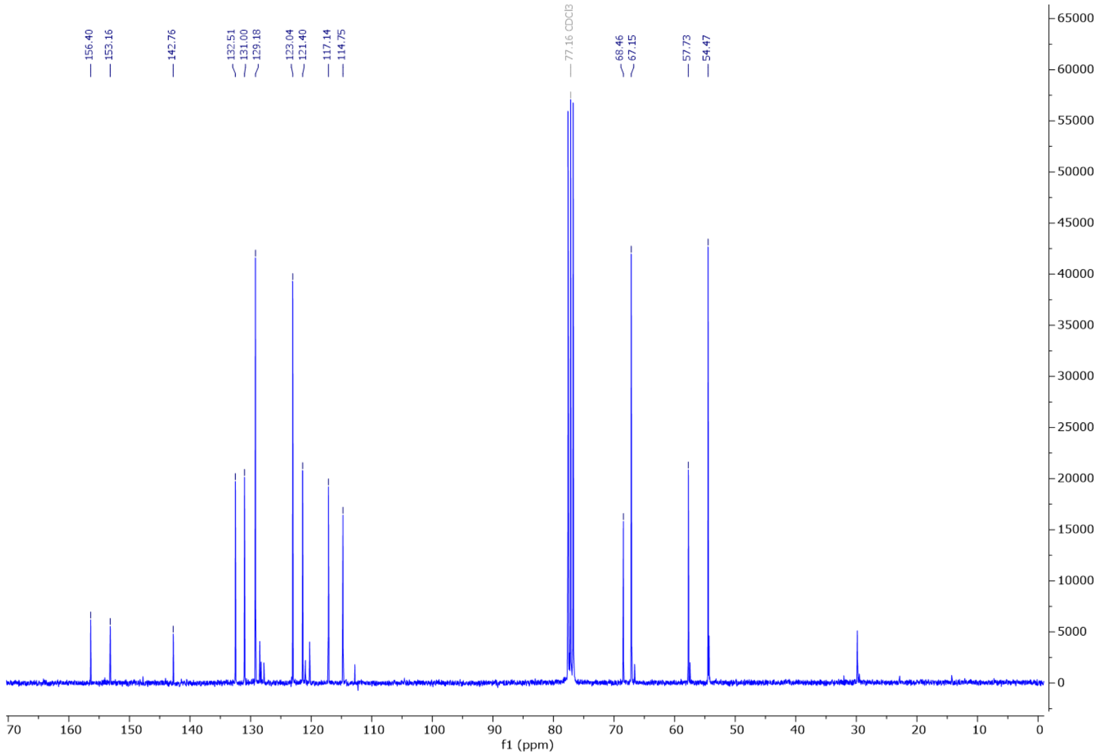

*Figure 14. ^{13}C NMR (75 MHz, $CDCl_3$) spectrum of compound **8a***

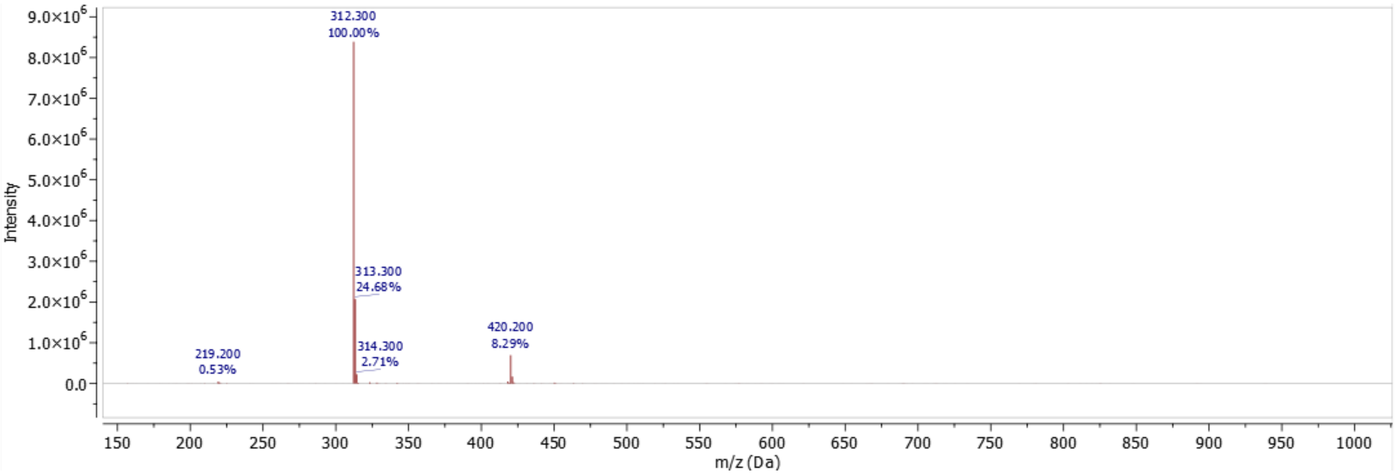

*Figure 15. Mass spectrum MALDI of compound **8a***

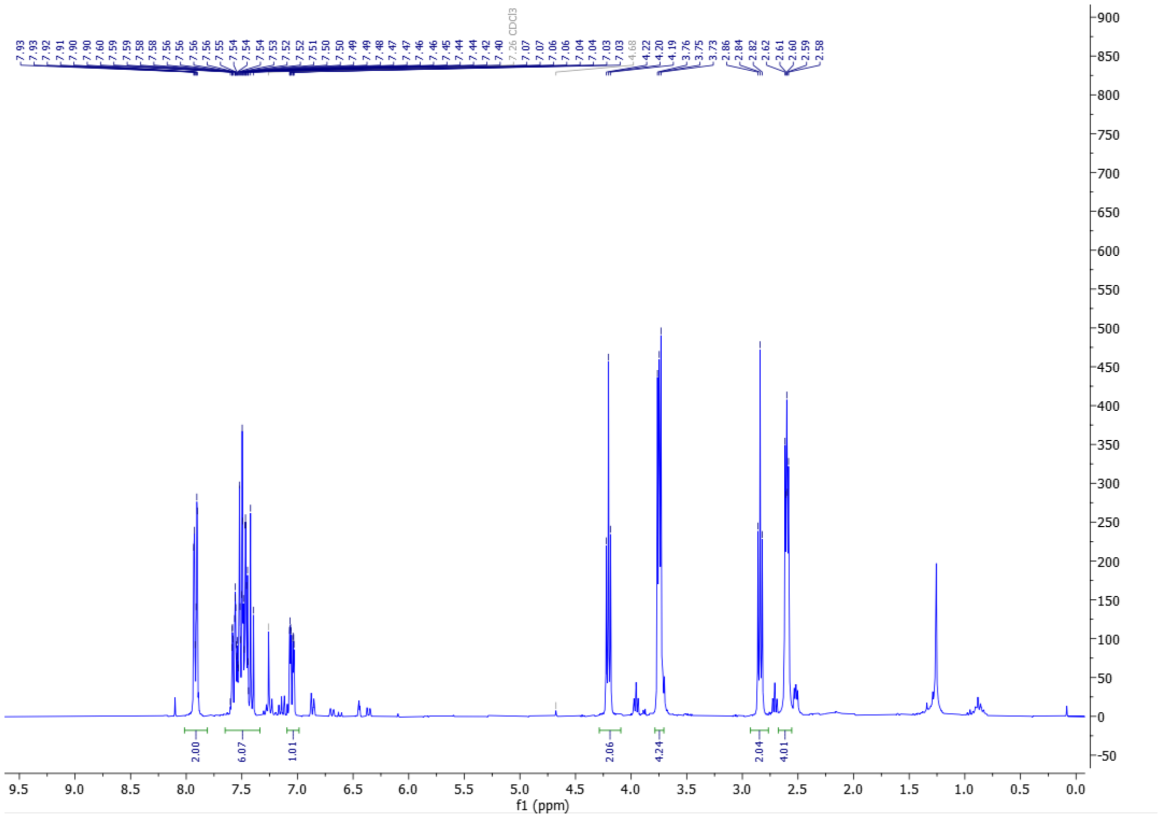

Figure 16. ¹H NMR (300 MHz, $CDCl_3$) spectrum of compound ***8b***

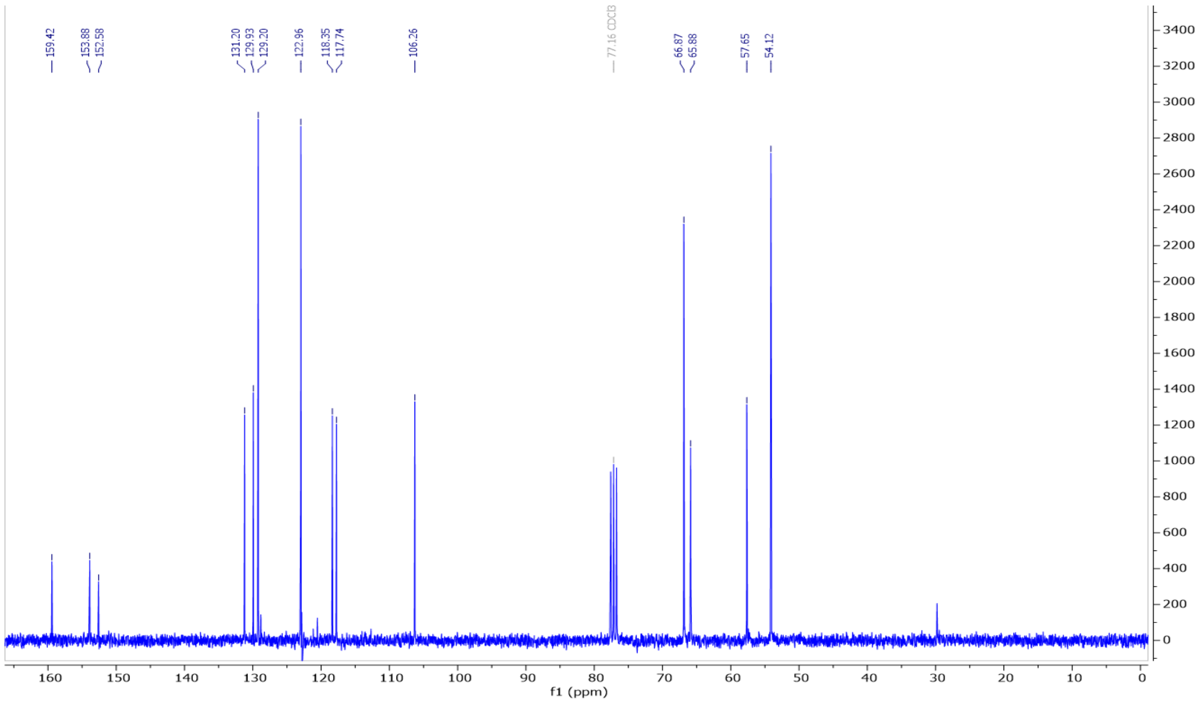

Figure 17. ¹³C NMR (75 MHz, $CDCl_3$) spectrum of compound ***8b***

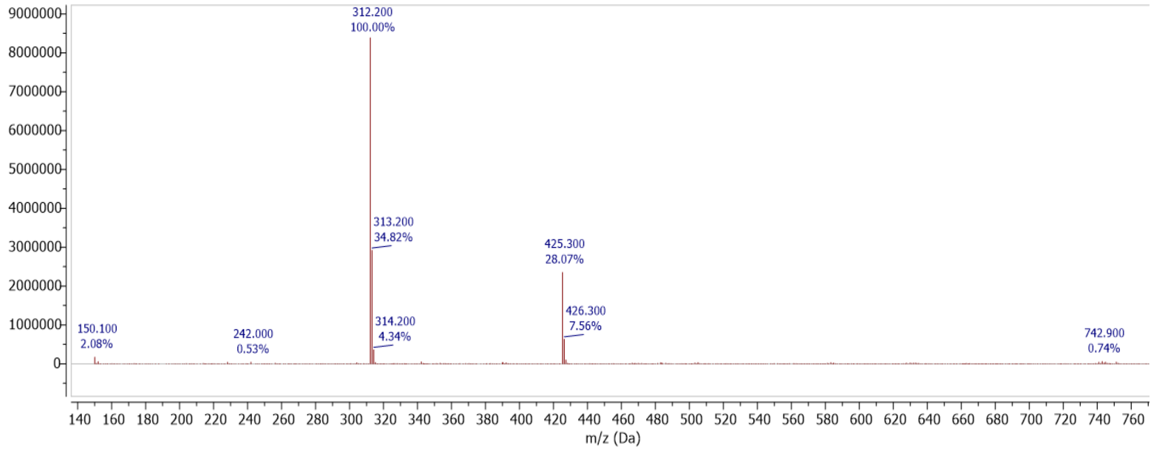

*Figure 18. Mass spectrum ESI$^+$ of compound **8b***

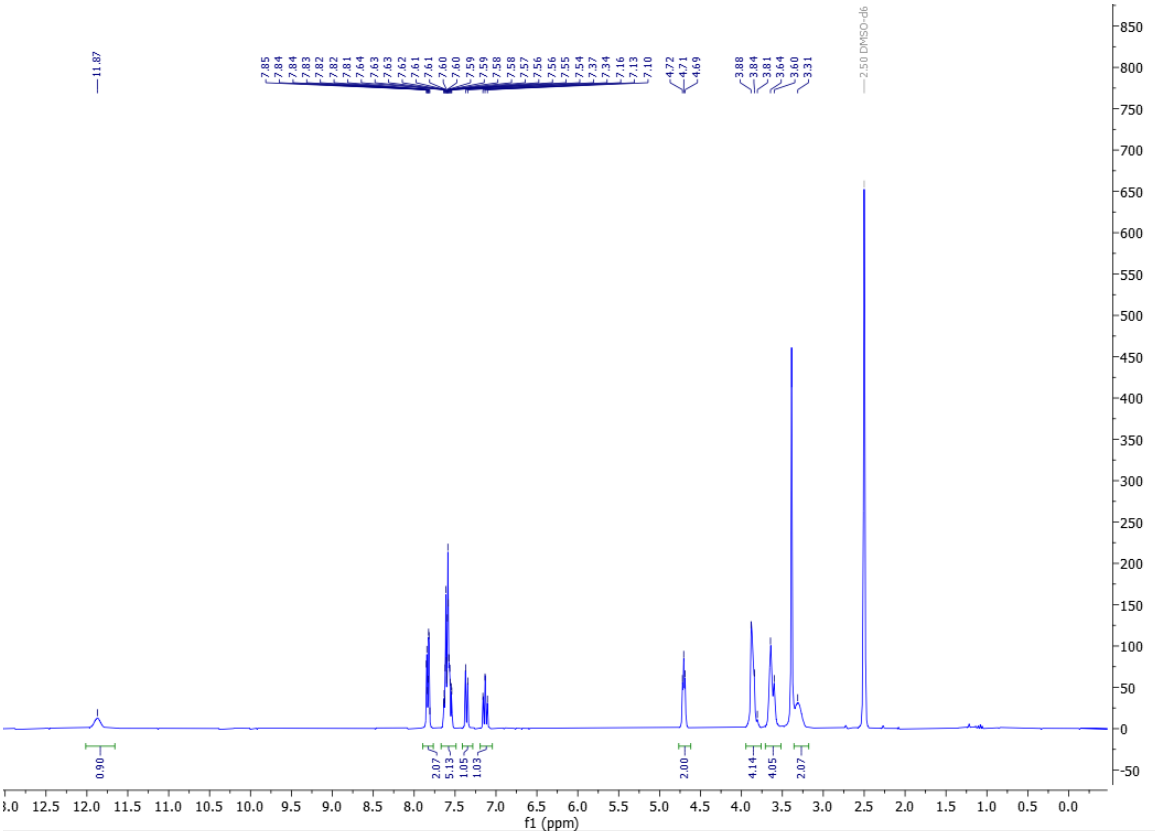

*Figure 19. ^{1}H NMR (300 MHz, DMSO-d6) spectrum of compound **9a***

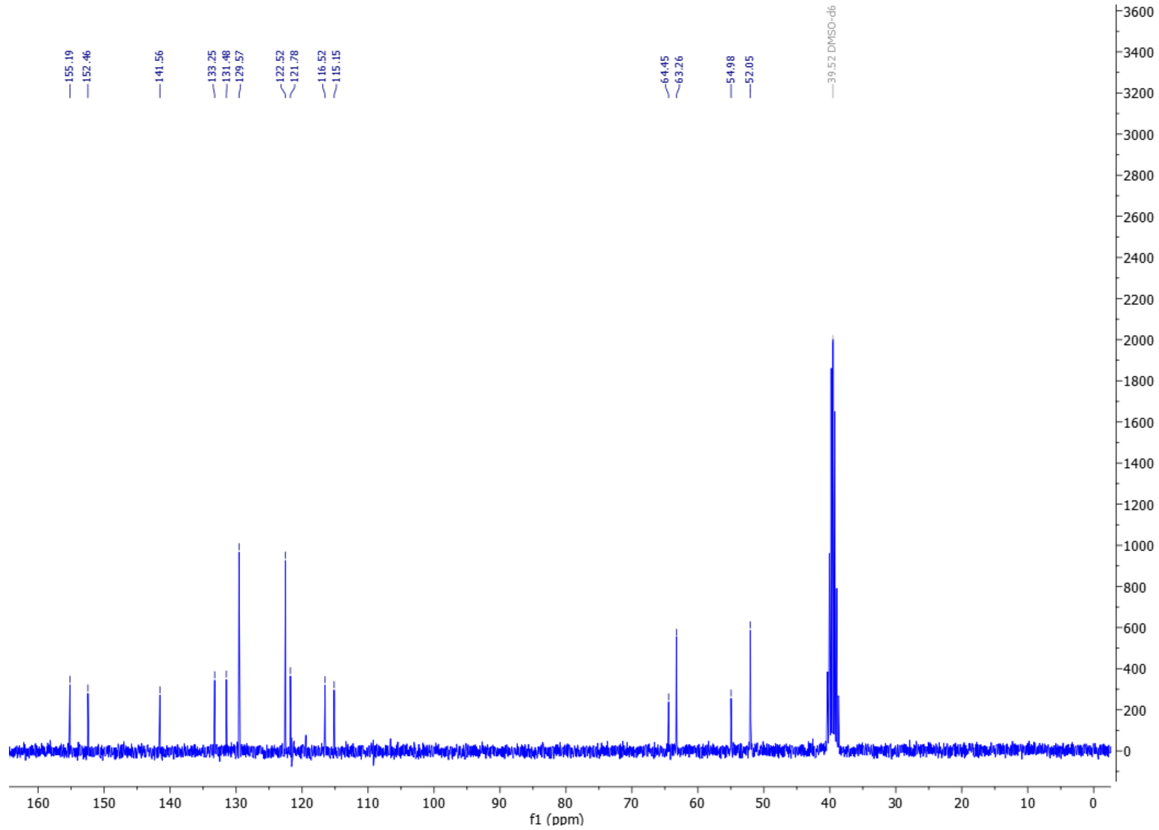

Figure 20. ^13^C NMR (75 MHz, DMSO-d6) spectrum of compound ***9a***

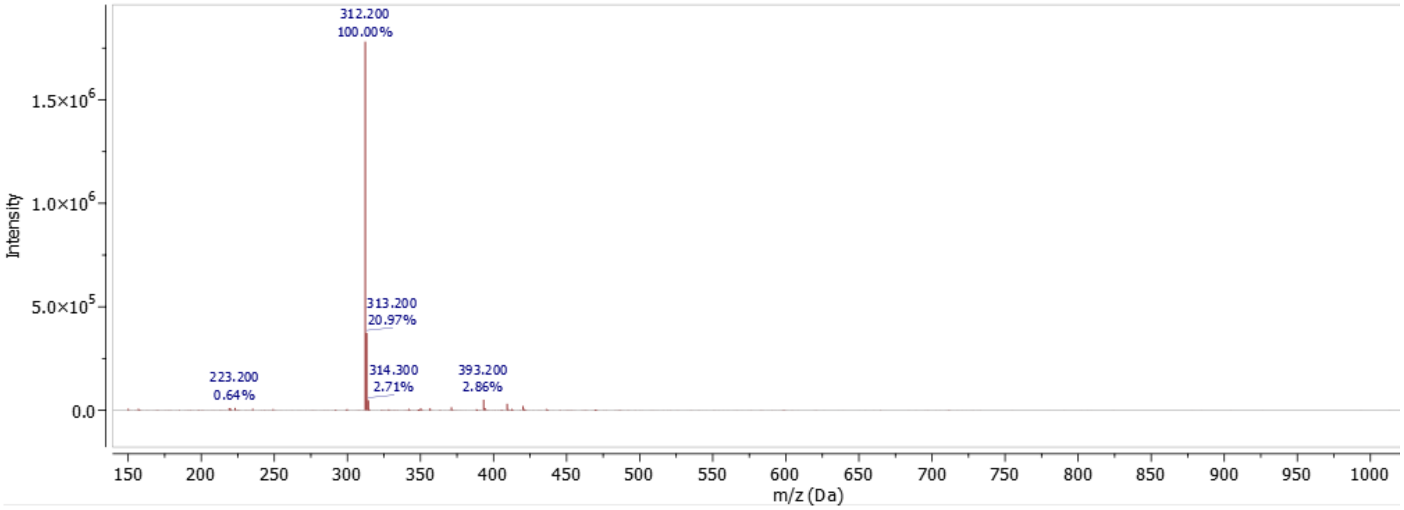

Figure 21. Mass spectrum ESI^+^ of compound ***9a***

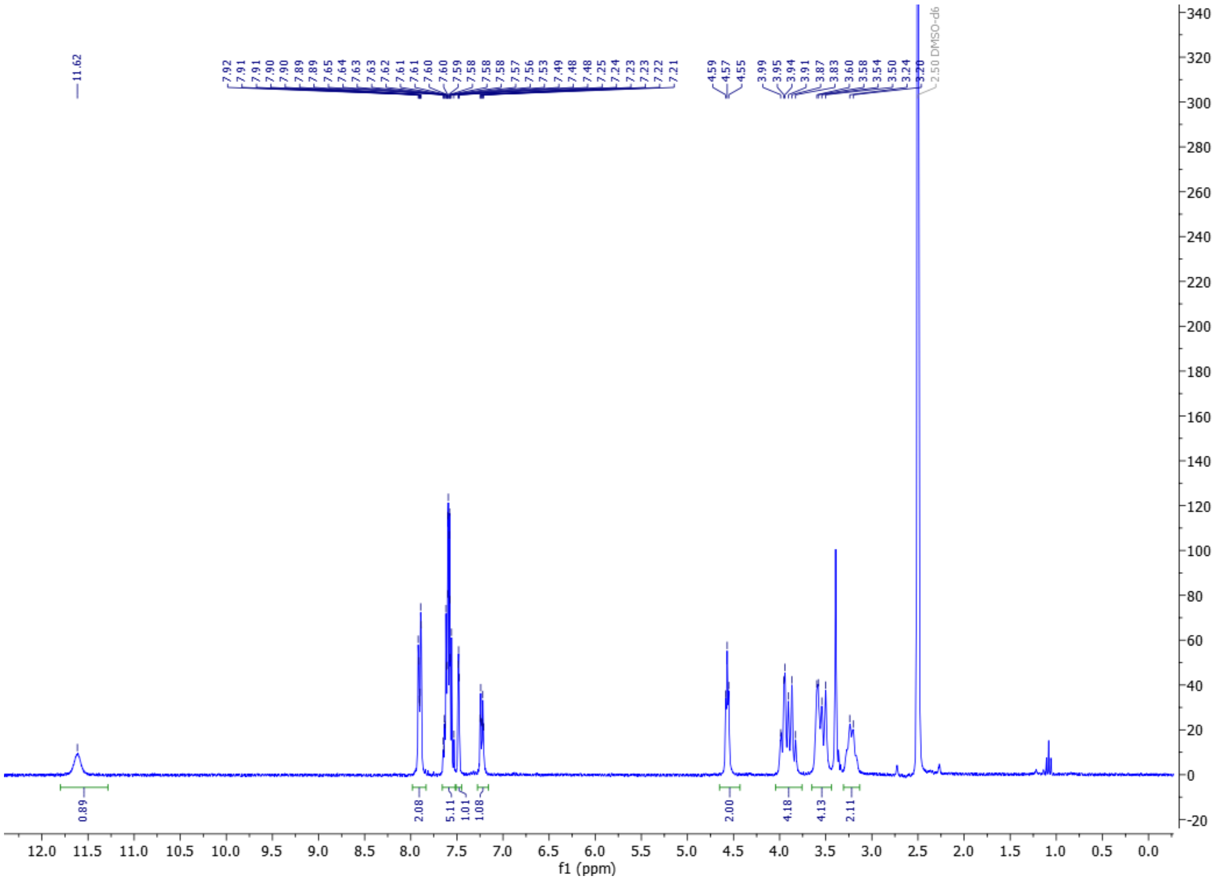

*Figure 22. ^{1}H NMR (300 MHz, DMSO-d6) spectrum of compound **9b***

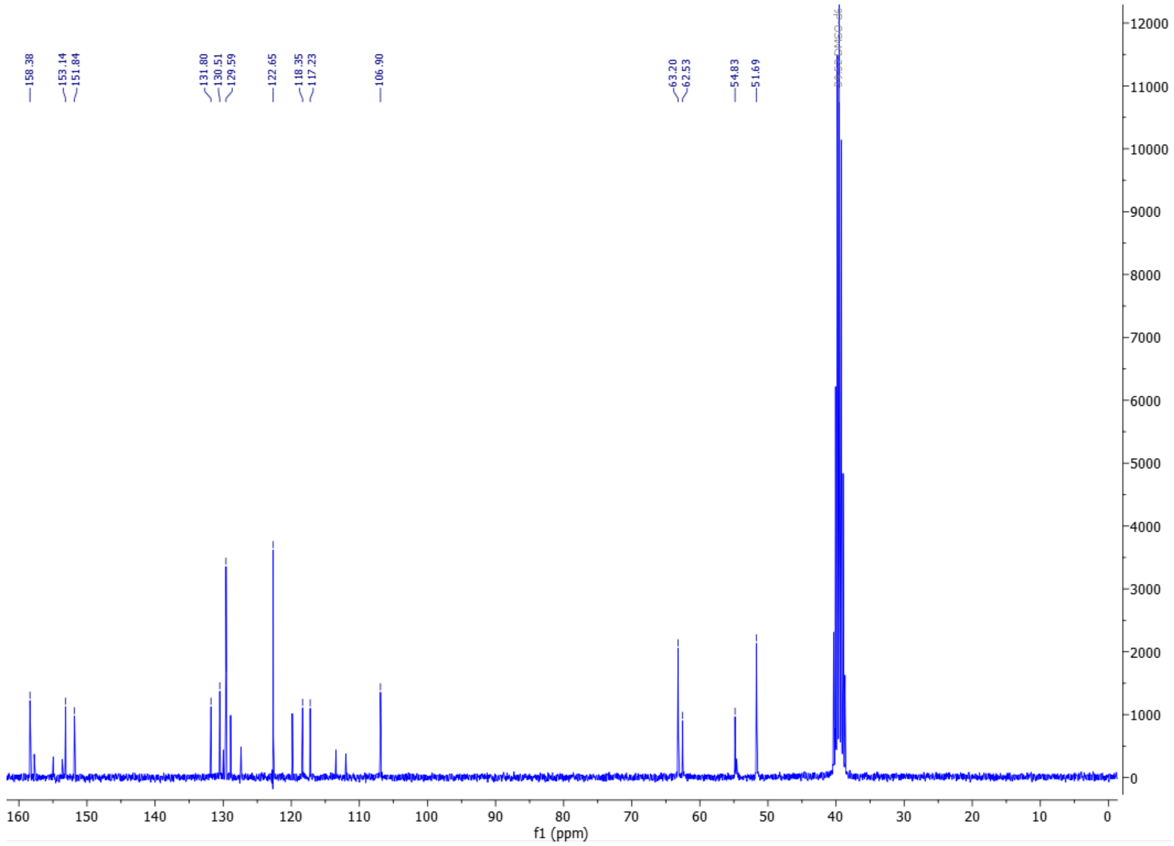

*Figure 23. ^{13}C NMR (75 MHz, DMSO-d6) spectrum of compound **9b***

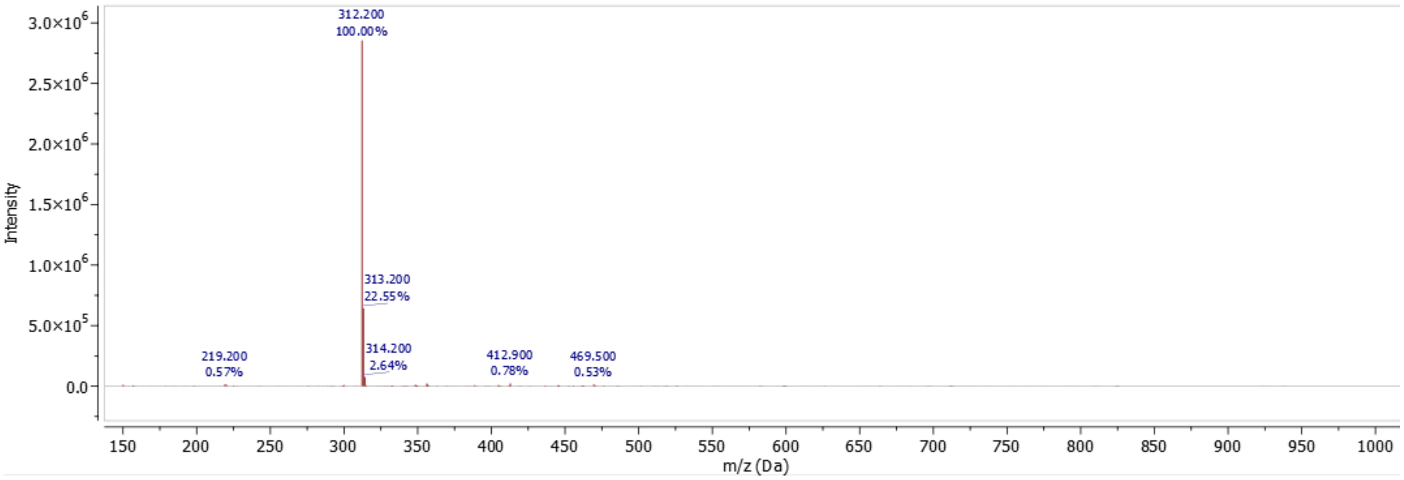

Figure 24. Mass spectrum ESI$^+$ of compound ***9b***

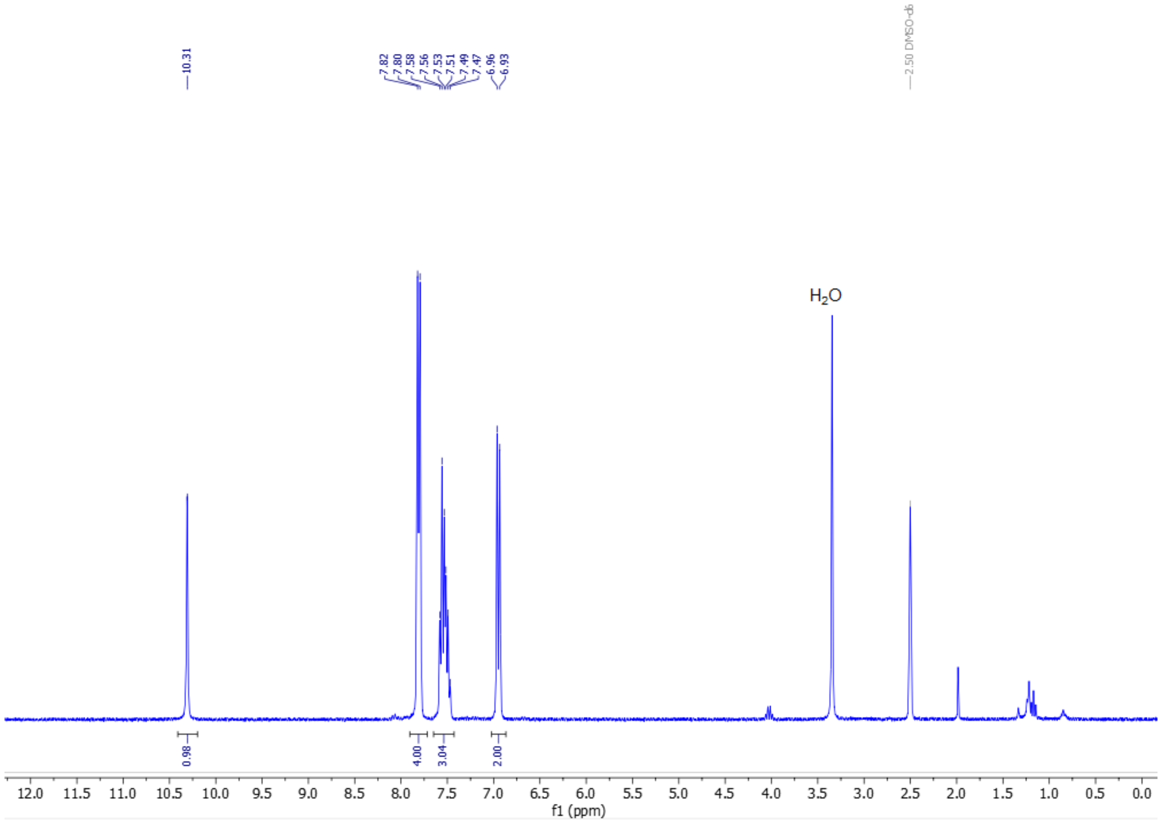

Figure 25. ^{1}H NMR (300 MHz, DMSO-d6) spectrum of compound ***11***

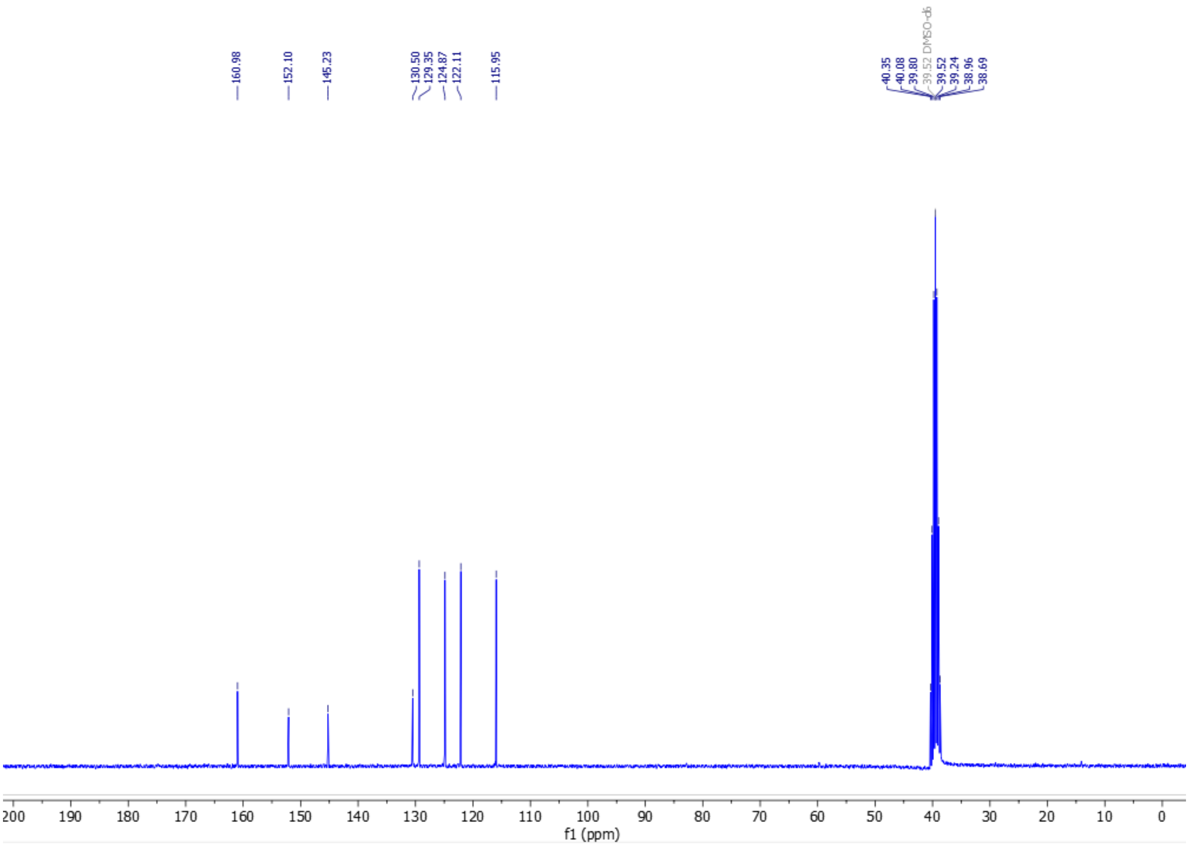

Figure 26. ^{13}C NMR (75 MHz, DMSO-d6) spectrum of compound ***11***

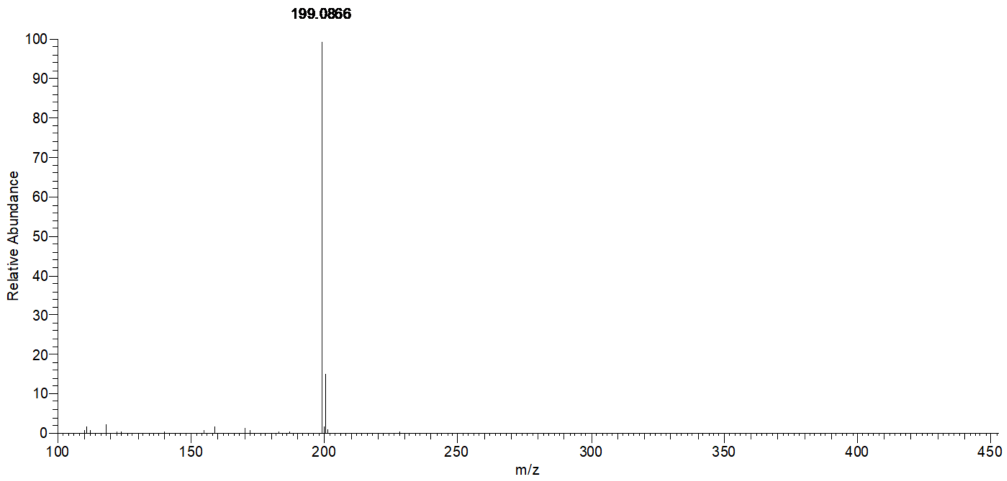

Figure 27. Mass spectrum ESI^{+} of compound ***11***

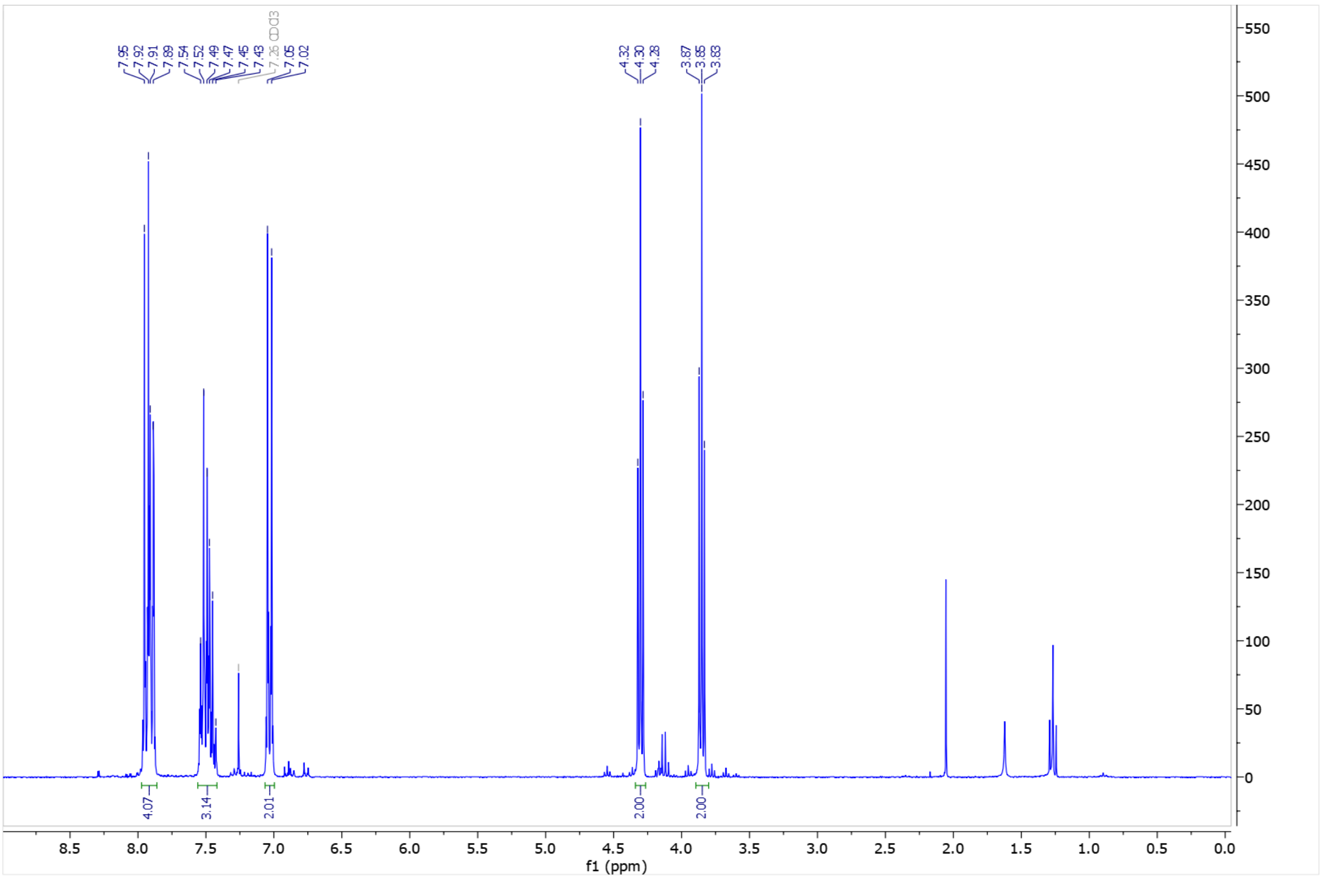

Figure 28. ^{1}H NMR (300 MHz, $CDCl_3$) spectrum of compound **12**

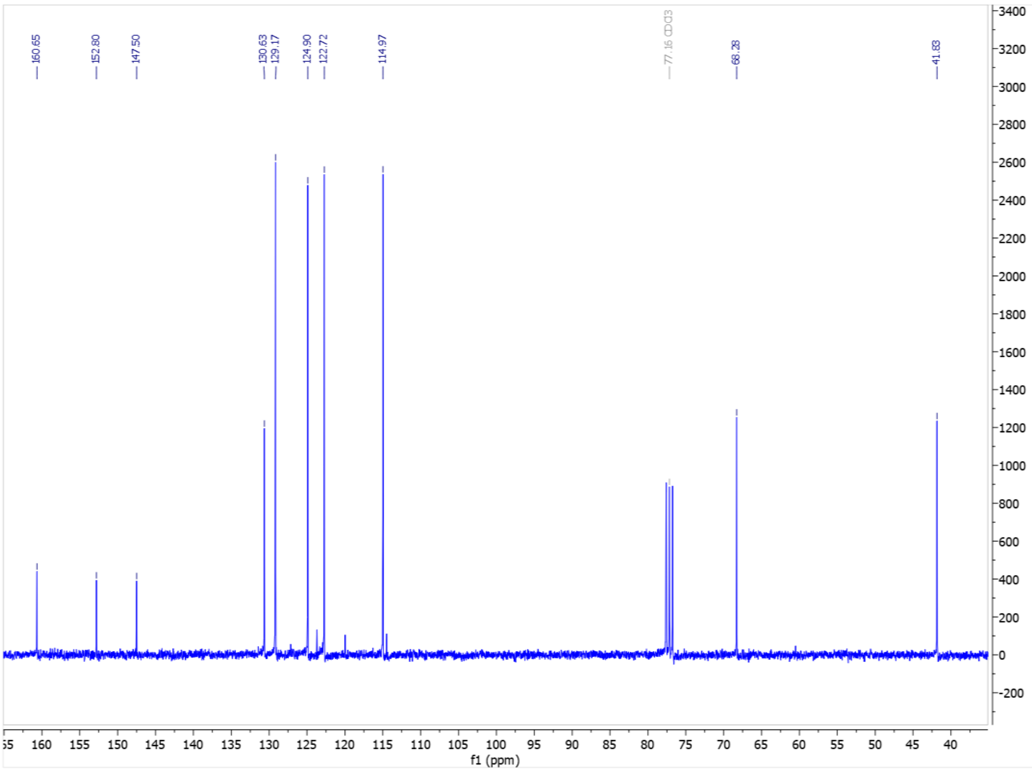

Figure 29. ^{13}C NMR (75 MHz, $CDCl_3$) spectrum of compound **12**

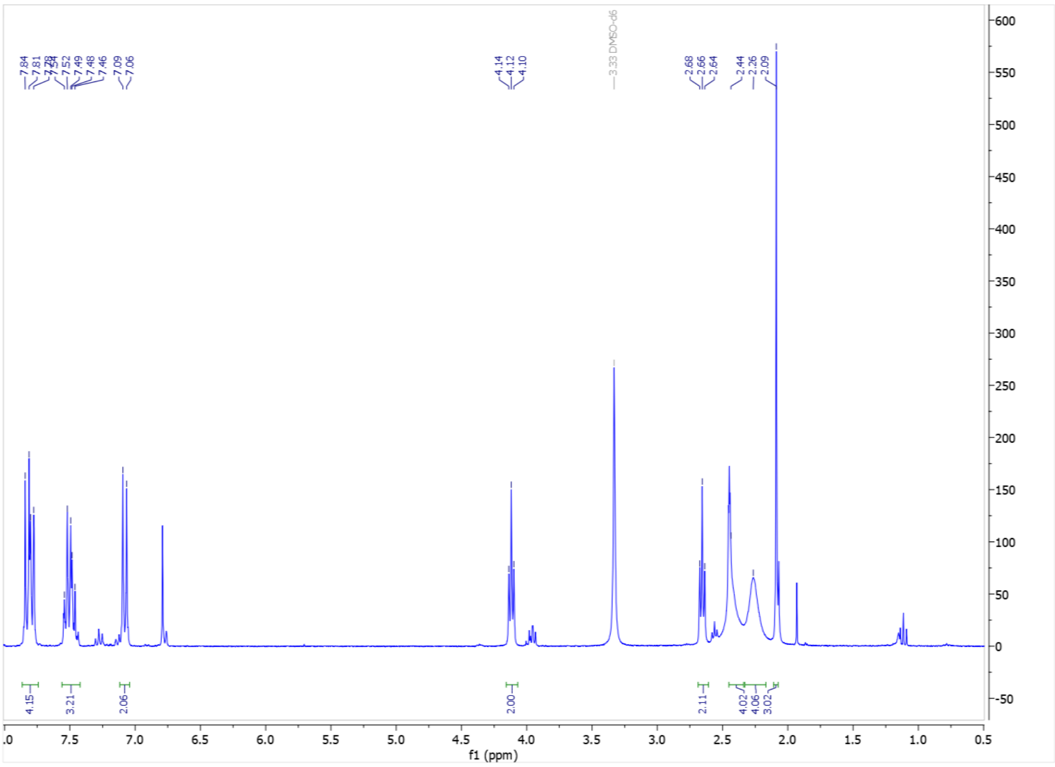

*Figure 30. ¹H NMR (300 MHz, DMSO-d6) spectrum of compound **13***

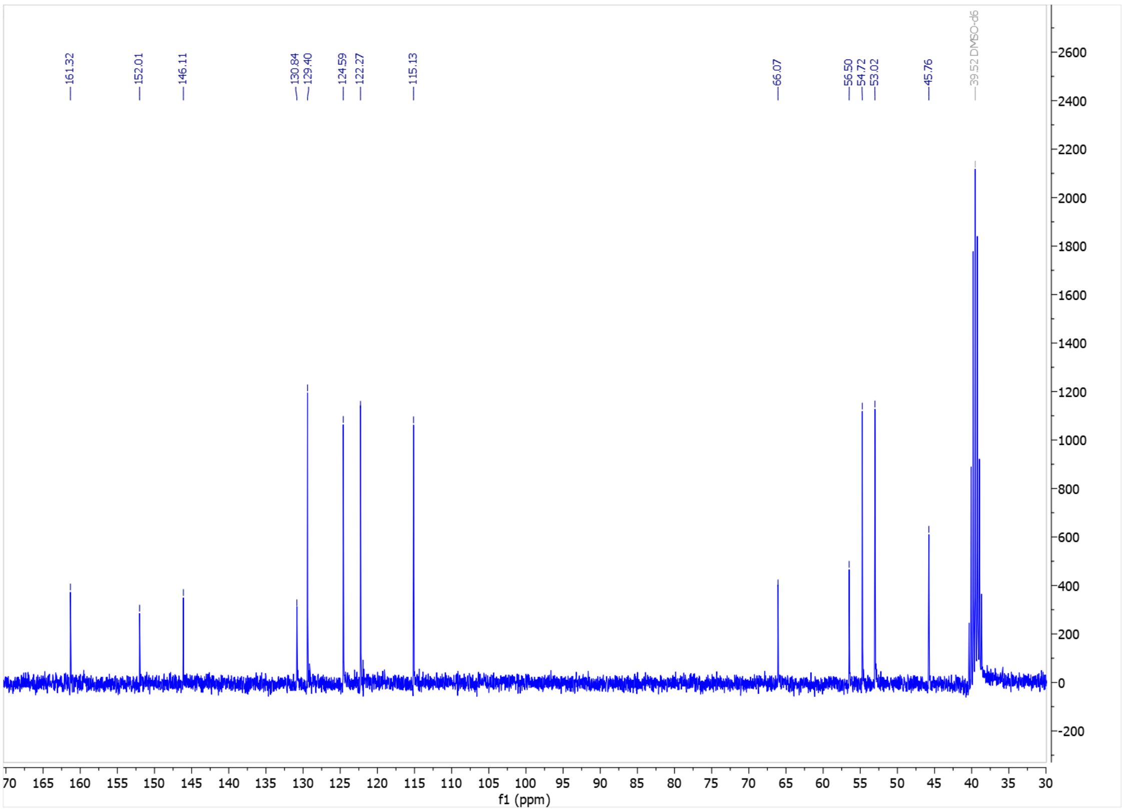

*Figure 31. ¹³C NMR (75 MHz, DMSO-d6) spectrum of compound **13***

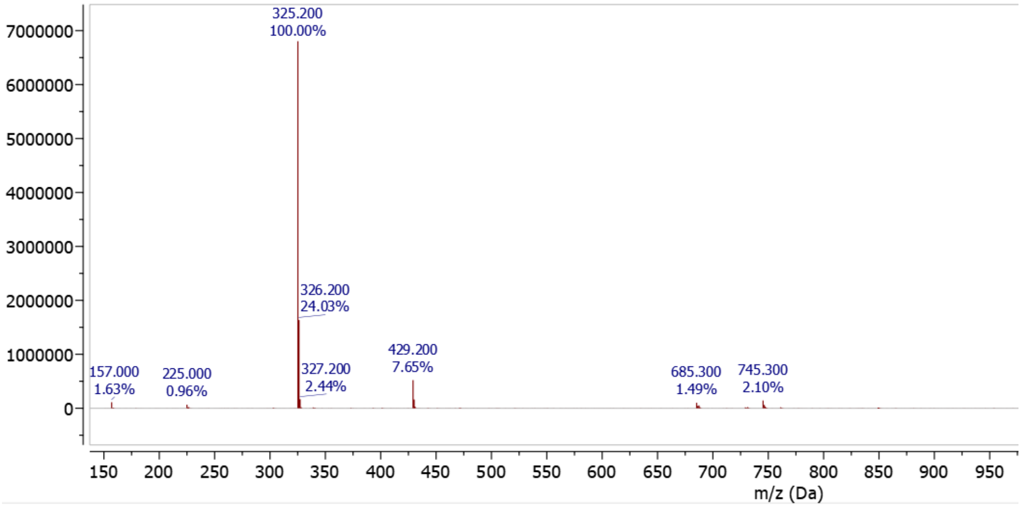

Figure 32. Mass spectrum ESI$^+$ of compound ***13***

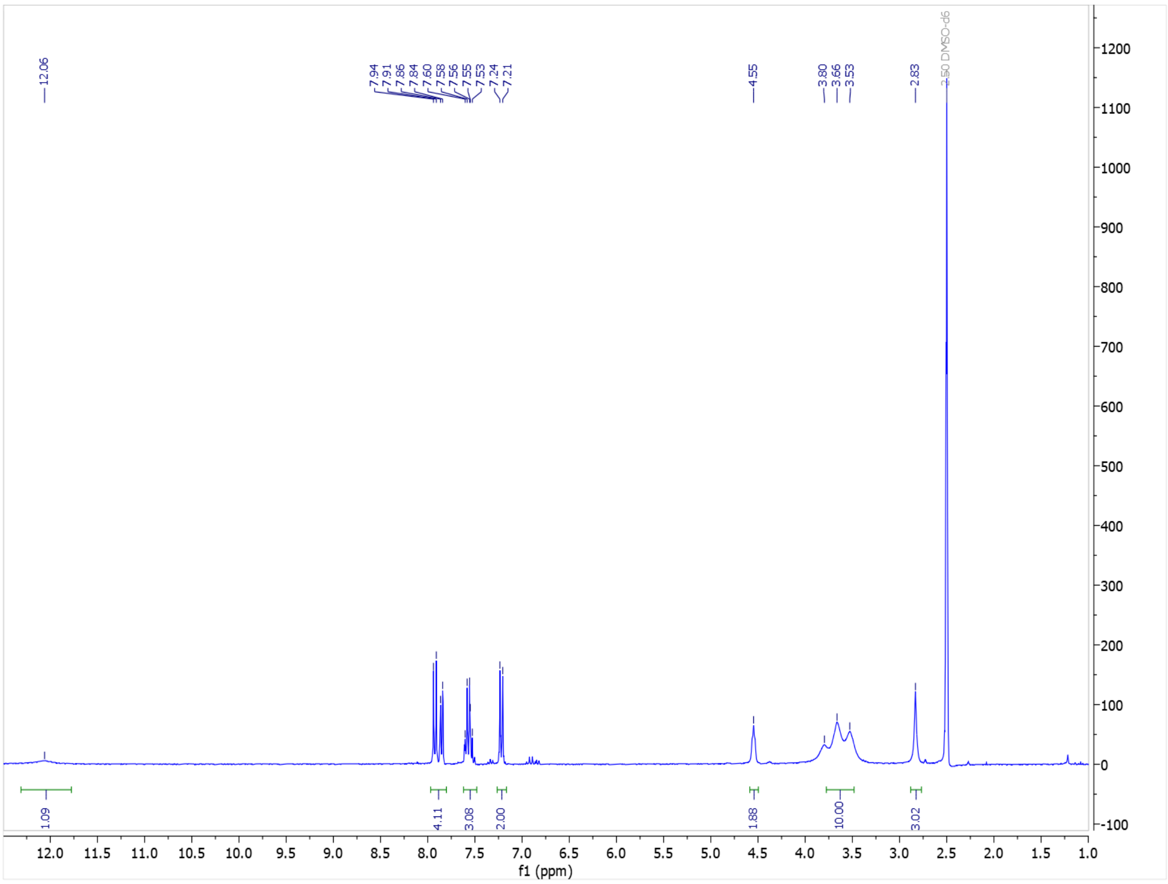

Figure 33. ^{1}H NMR (300 MHz, DMSO-d6) spectrum of compound ***14***

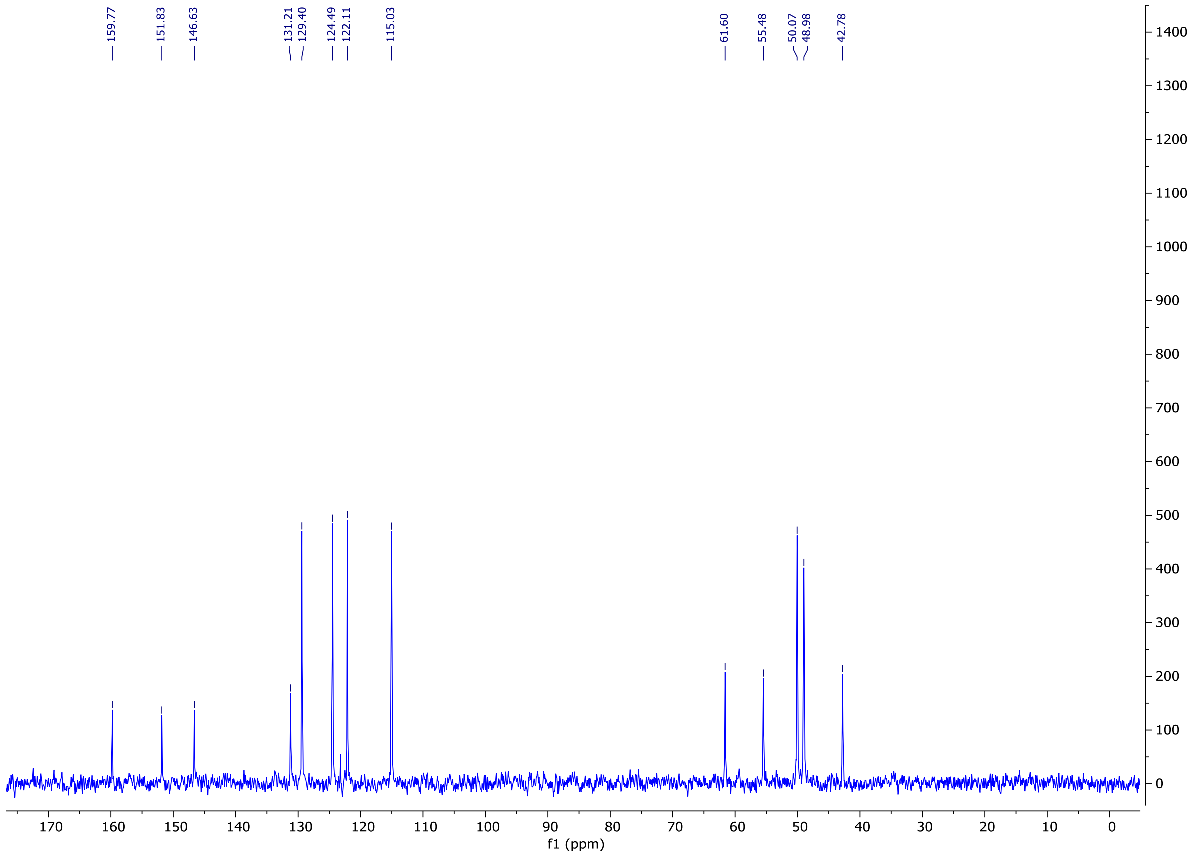

*Figure 34. ^{13}C NMR (75 MHz, D_2O) spectrum of compound **14***

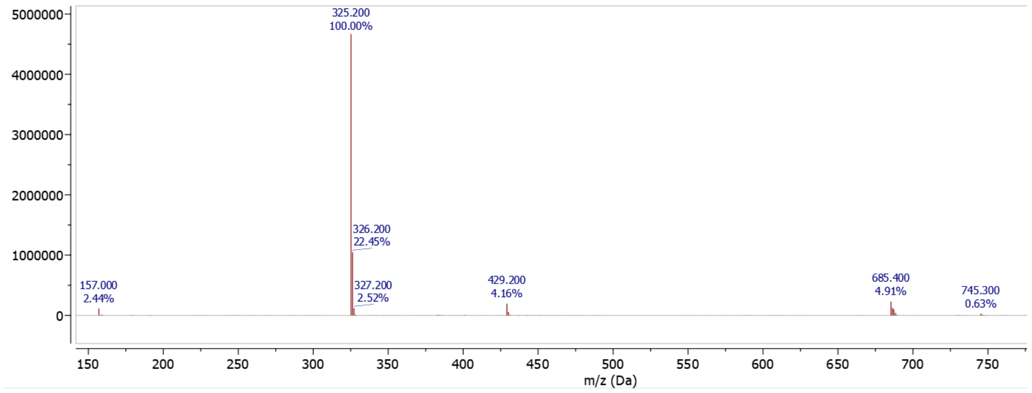

*Figure 35. Mass spectrum ESI^{+} of compound **14***

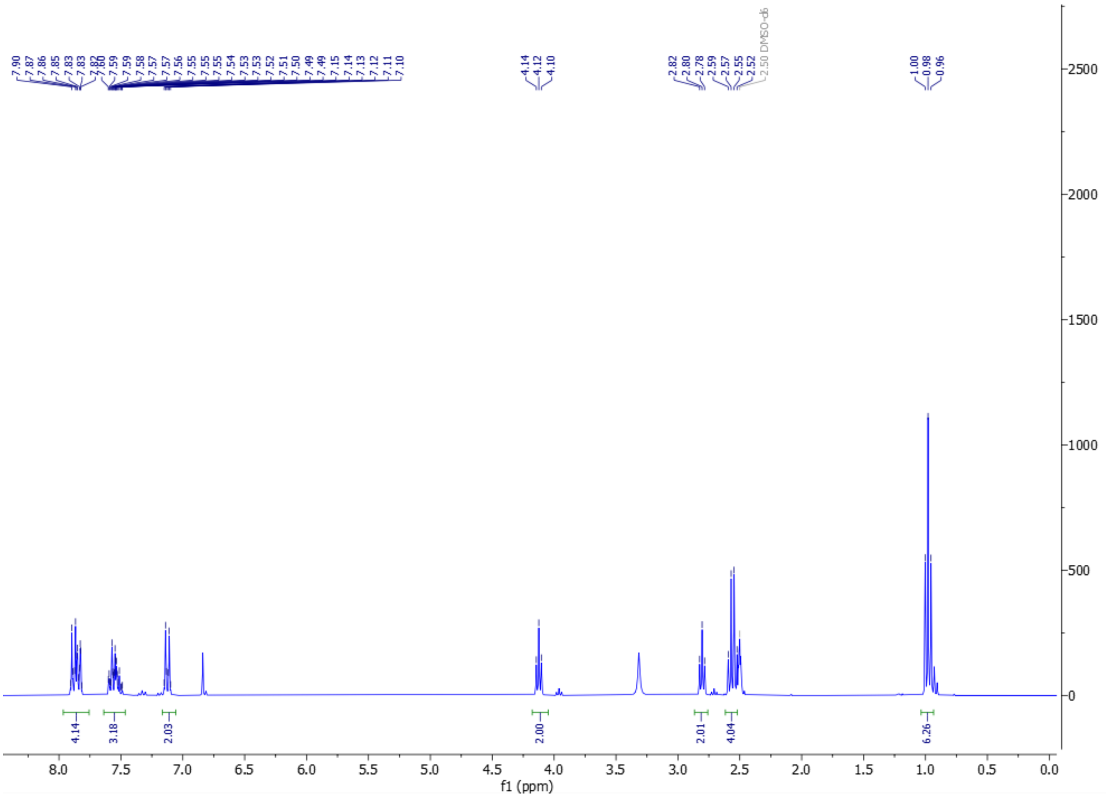

Figure 36. ^{1}H NMR (300 MHz, DMSO-d6) spectrum of compound **15**

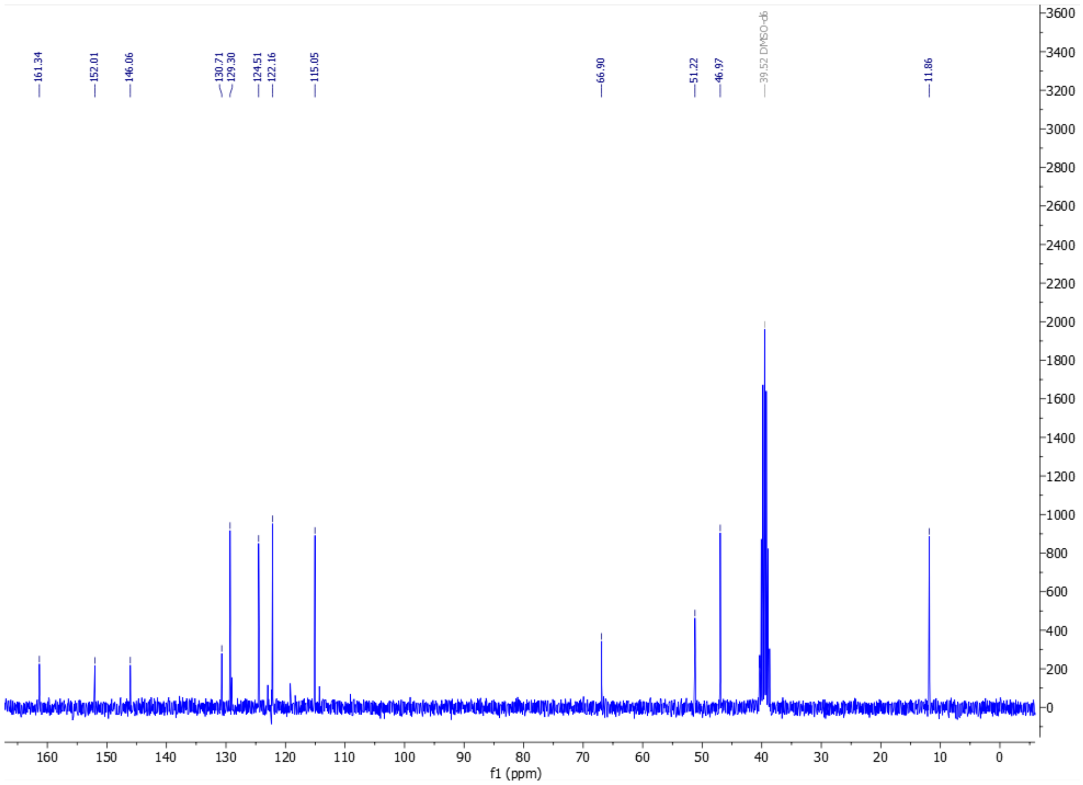

Figure 37. ^{13}C NMR (75 MHz, DMSO-d6) spectrum of compound **15**

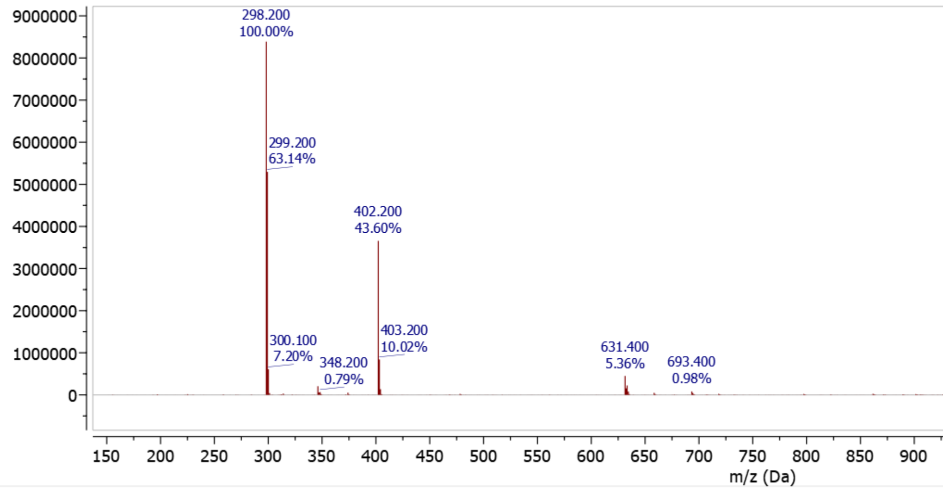

*Figure 38. Mass spectrum ESI$^+$ of compound **15***

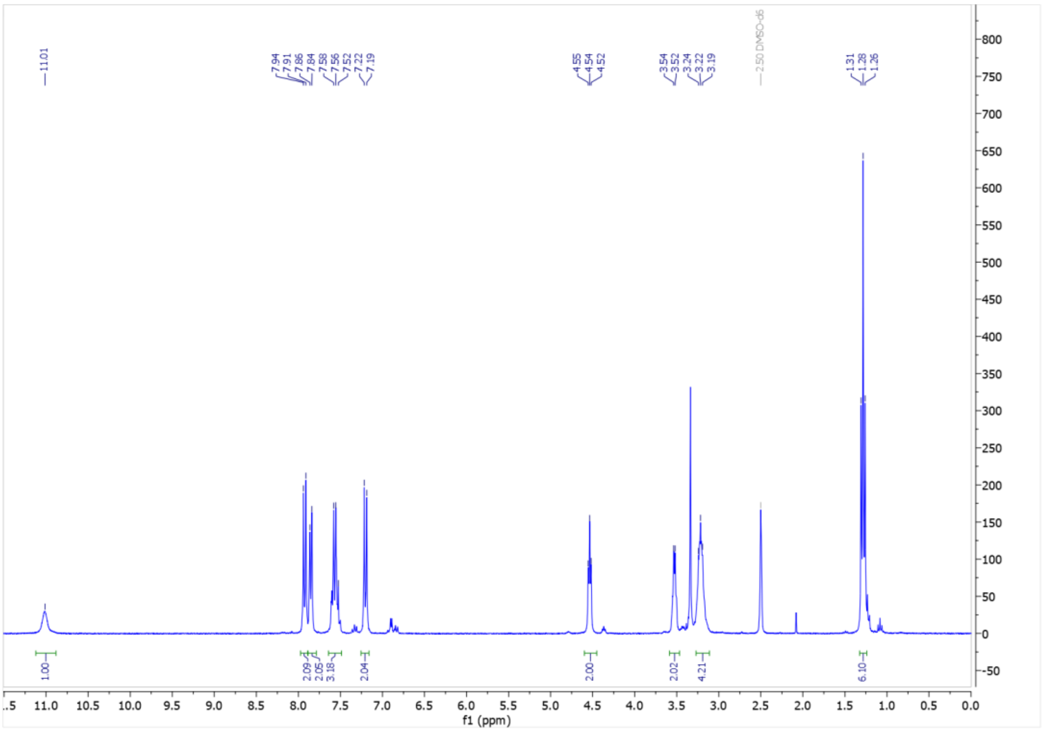

*Figure 39. ^{1}H NMR (300 MHz, DMSO-d6) spectrum of compound **16***

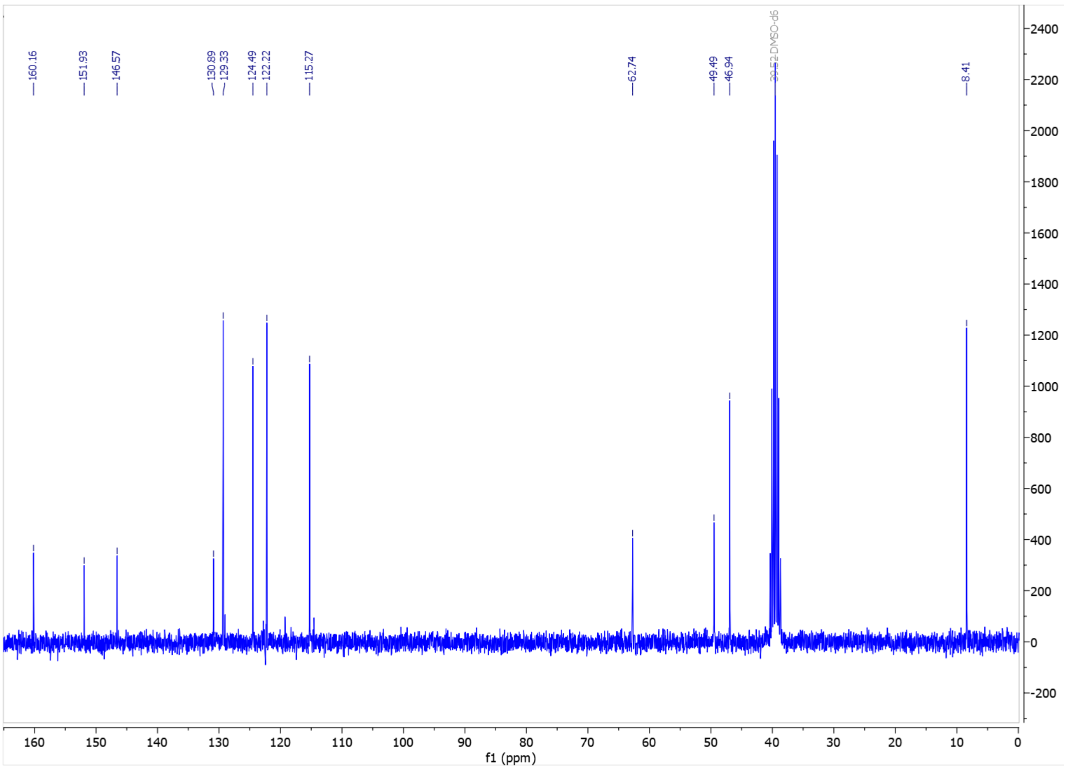

Figure 40. ^{13}*C NMR (75 MHz, DMSO-d6) spectrum of compound* ***16***

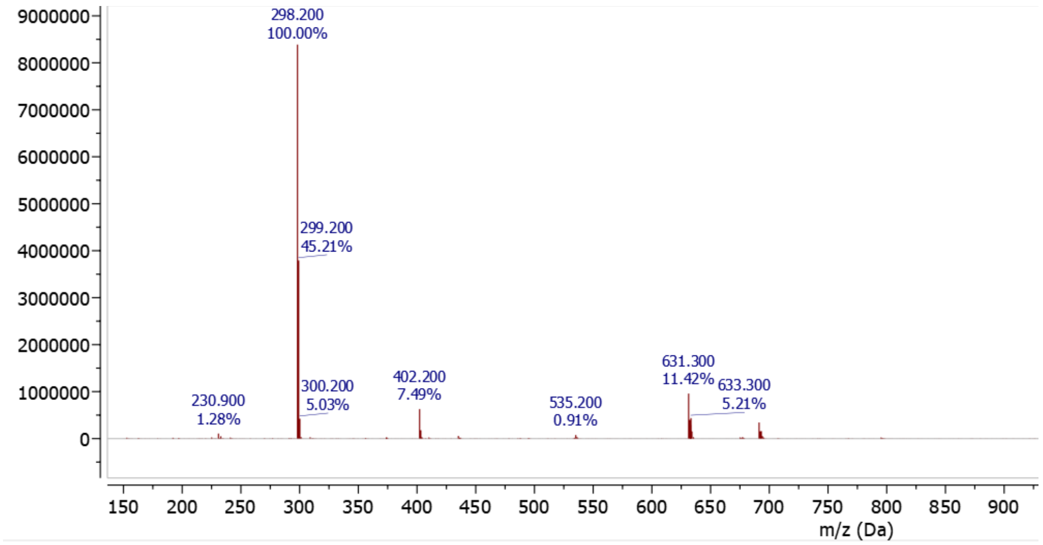

Figure 41. Mass spectrum ESI^{+} *of compound* ***16***

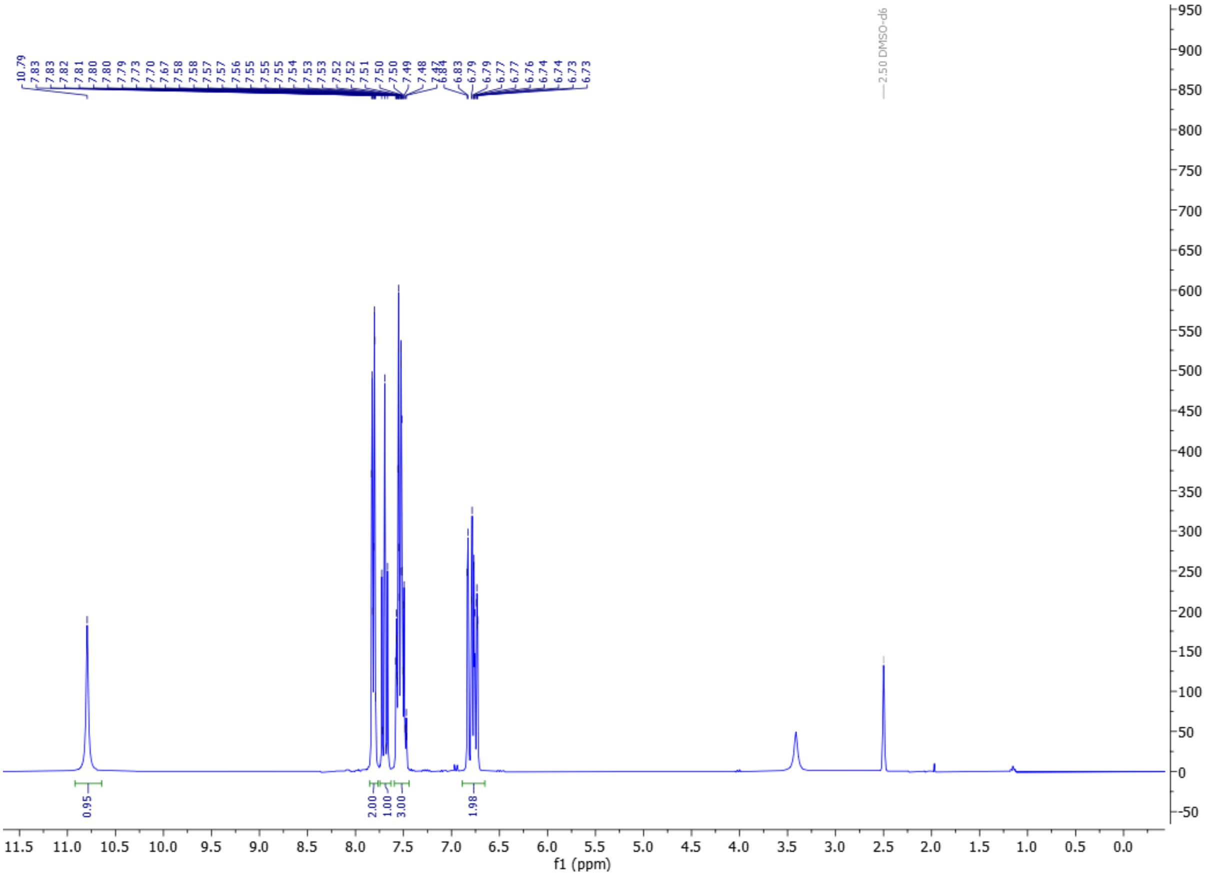

Figure 42. [1]*H NMR (300 MHz, DMSO-d6) spectrum of compound* ***18a***

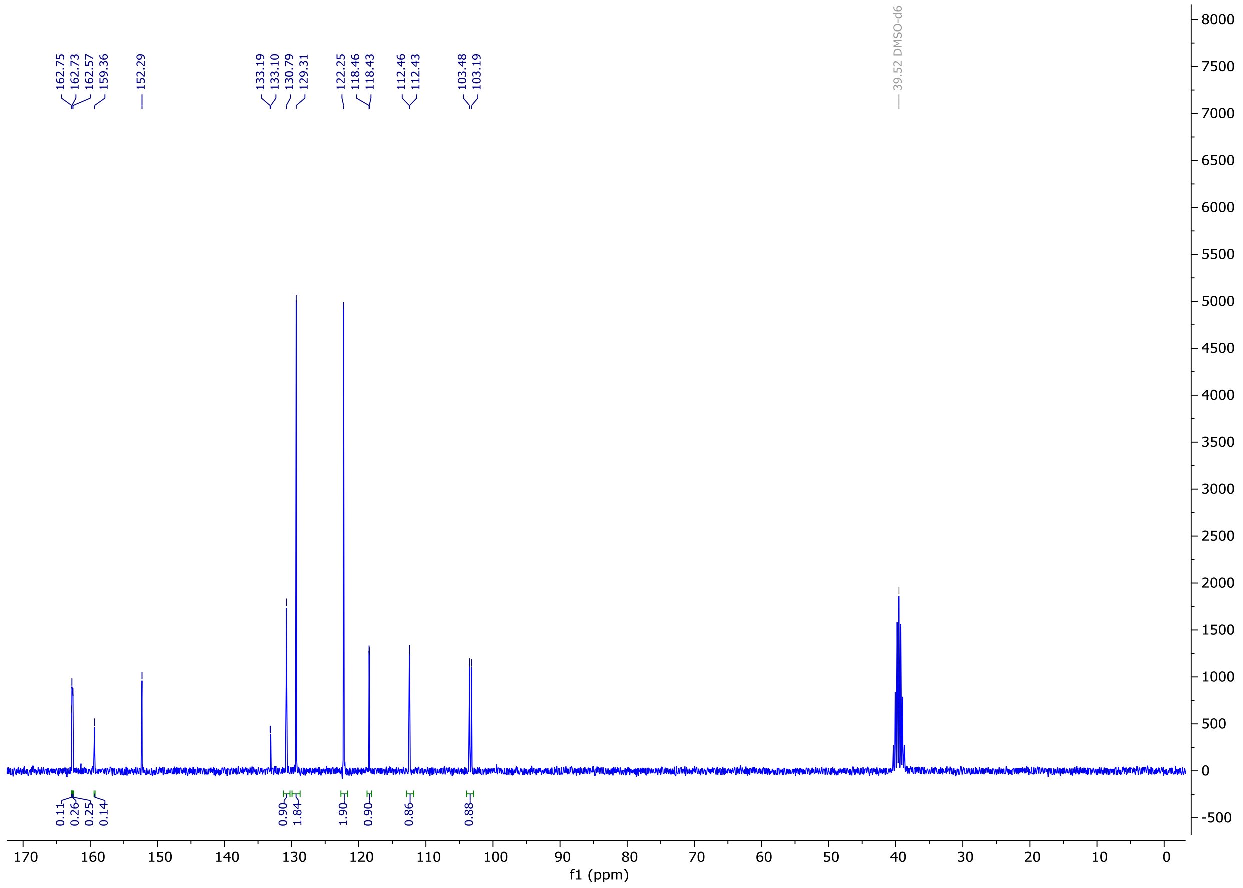

Figure 43. [13]*C NMR (75 MHz, DMSO-d6) spectrum of compound* ***18a***

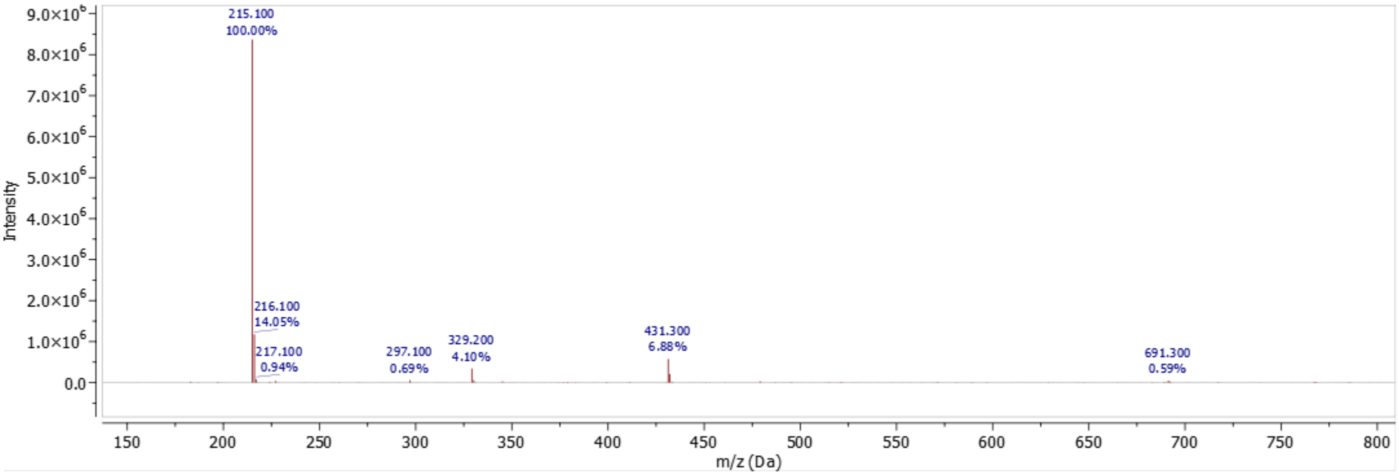

*Figure 44. Mass spectrum ESI$^-$ of compound **18a***

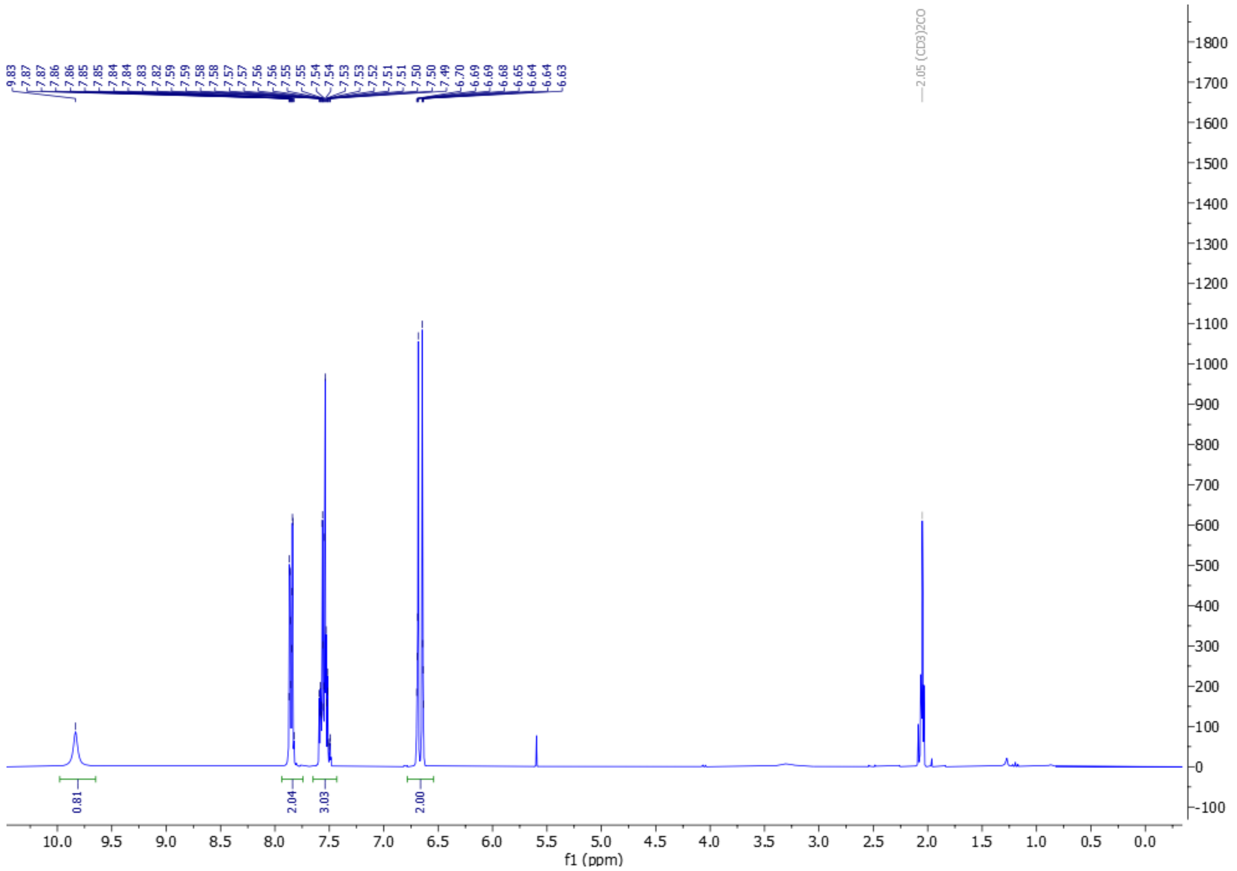

*Figure 45. ^{1}H NMR (300 MHz, acetone-d6) spectrum of compound **18b***

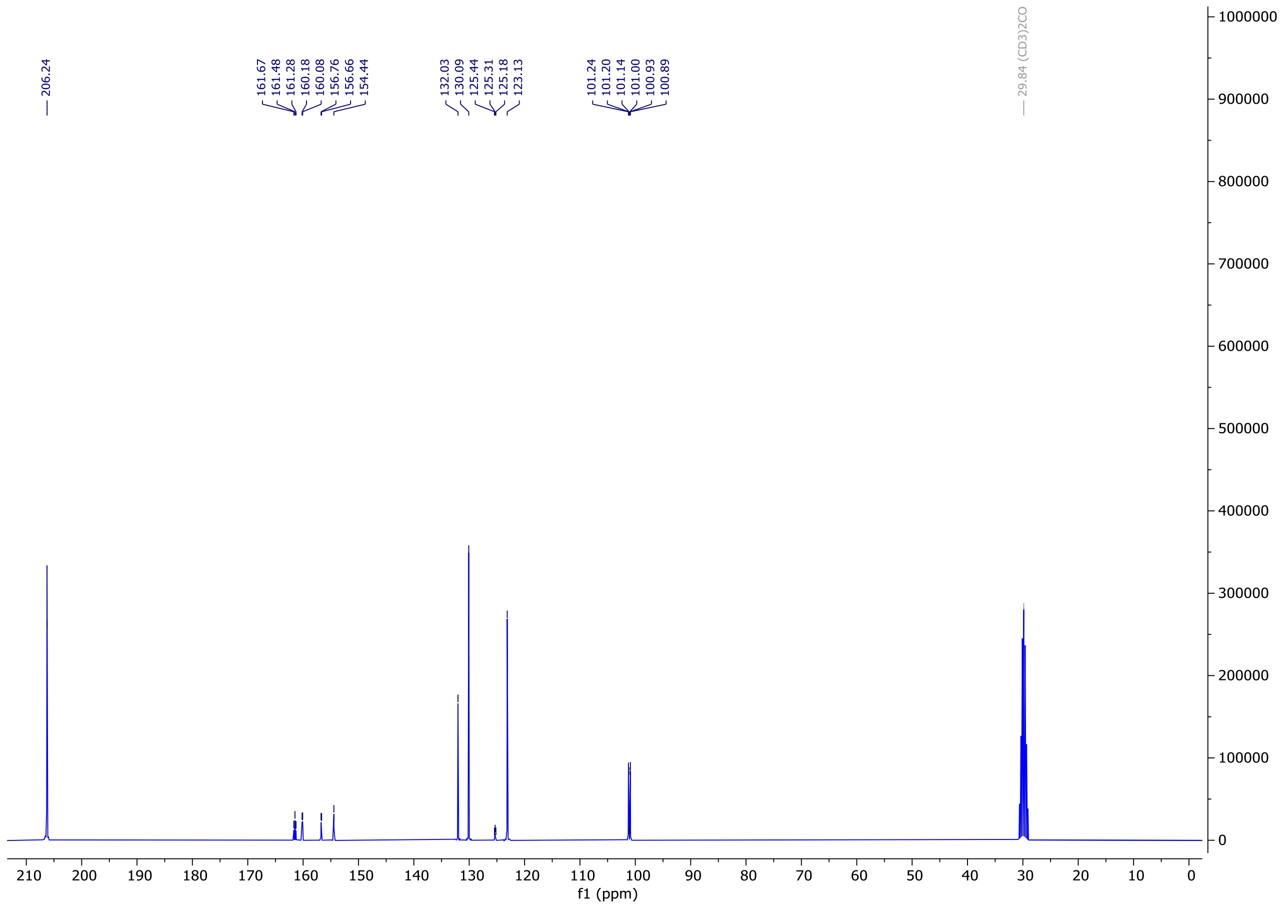

Figure 46. ¹³C NMR (75 MHz, acetone-d6) spectrum of compound **18b**

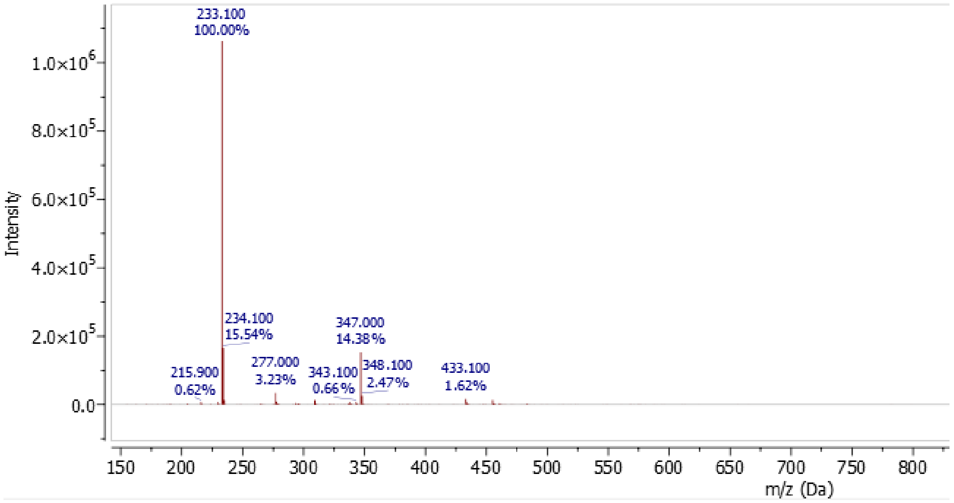

Figure 47. Mass spectrum ESI⁻ of compound ***18b***

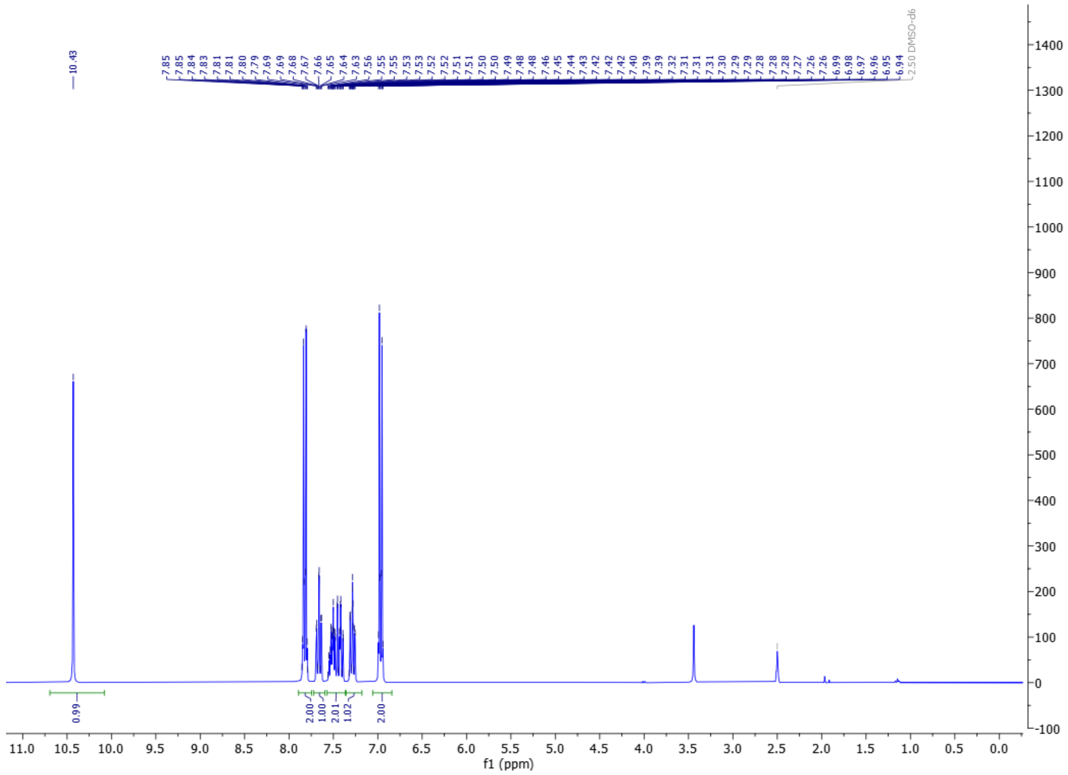

*Figure 48. ^{1}H NMR (300 MHz, DMSO-d6) spectrum of compound **18c***

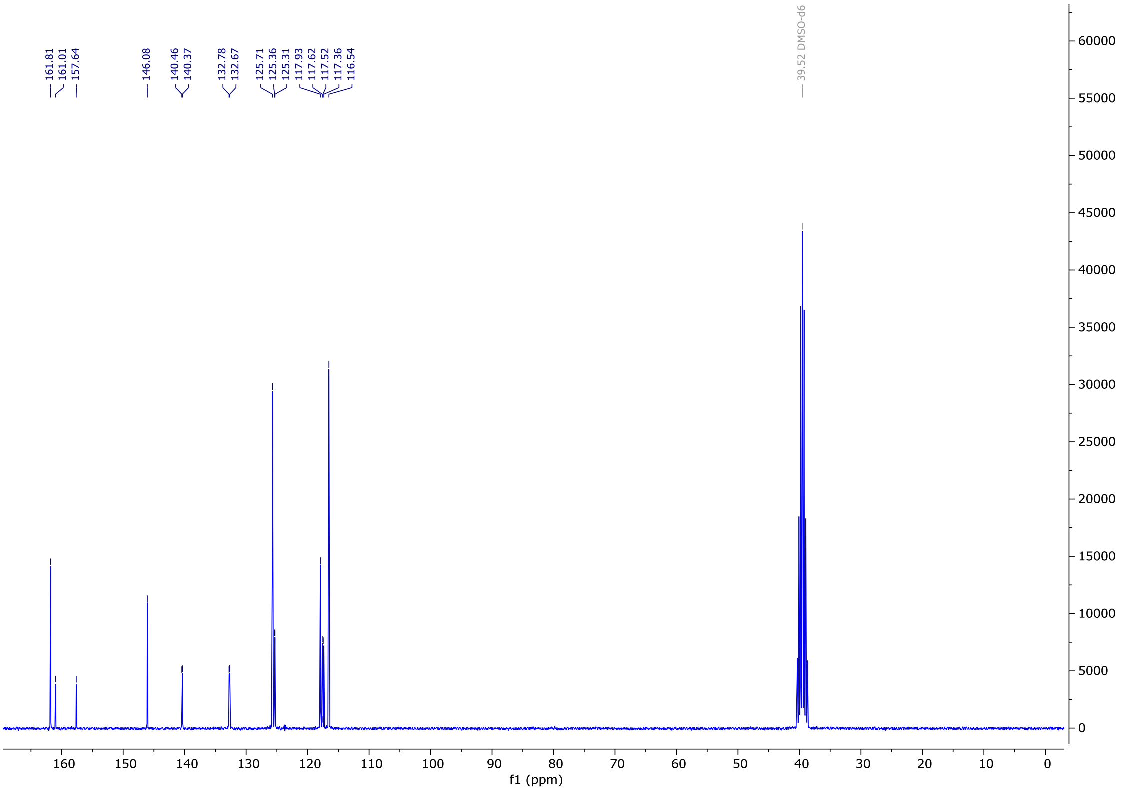

*Figure 49. ^{13}C NMR (75 MHz, DMSO-d6) spectrum of compound **18c***

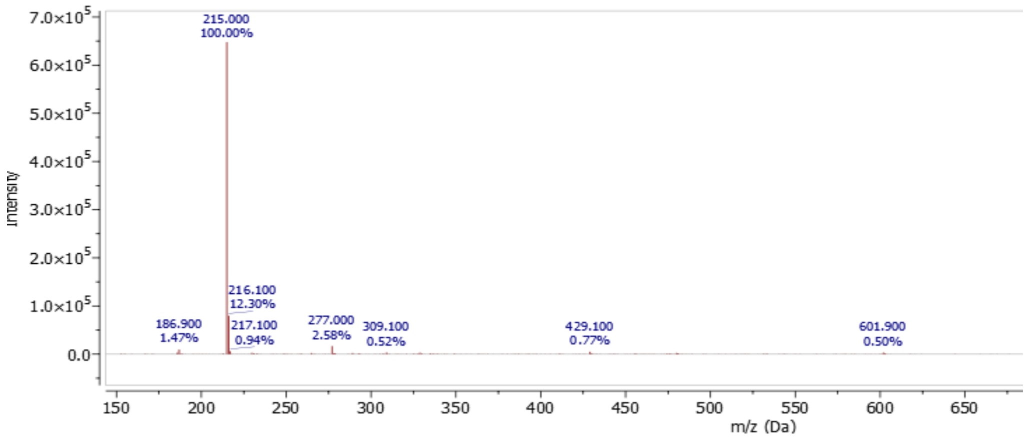

*Figure 50. Mass spectrum ESI⁻ of compound **18c***

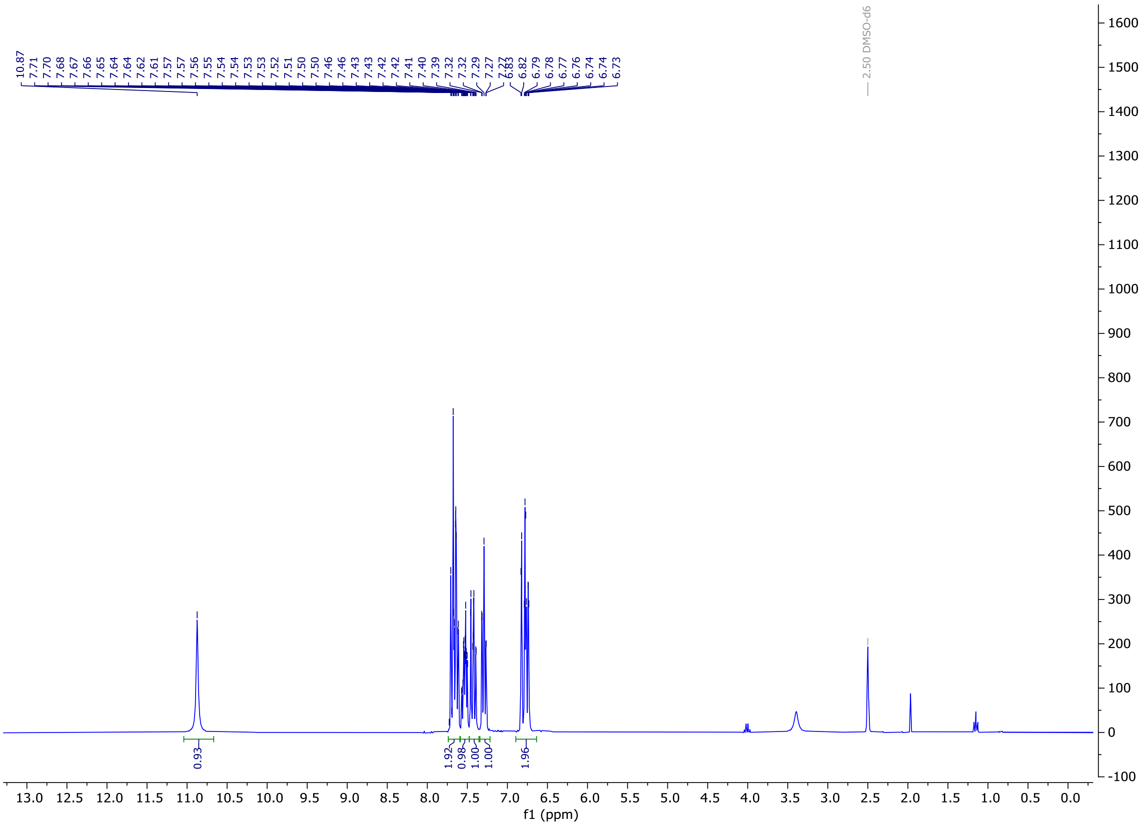

*Figure 51. ^{1}H NMR (300 MHz, DMSO-d6) spectrum of compound **18d***

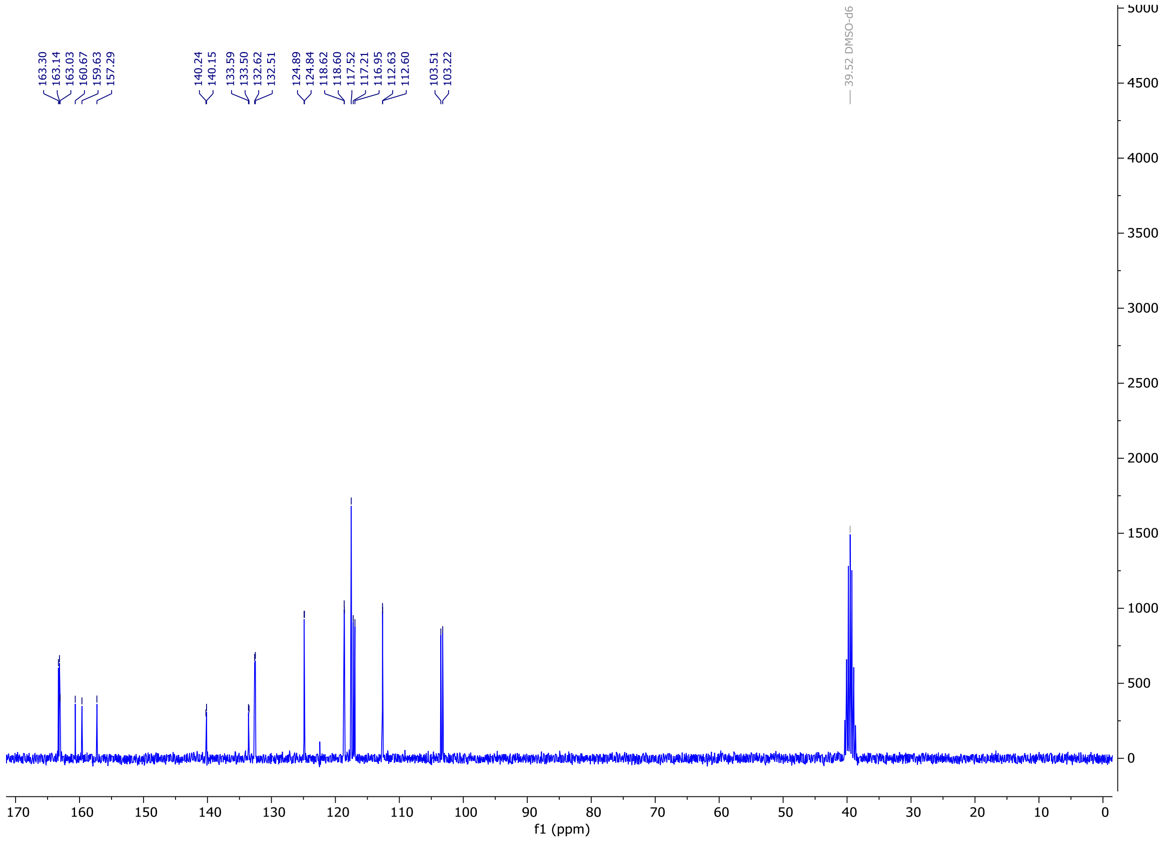

*Figure 52.^{13}C NMR (75 MHz, DMSO-d6) spectrum of compound **18d***

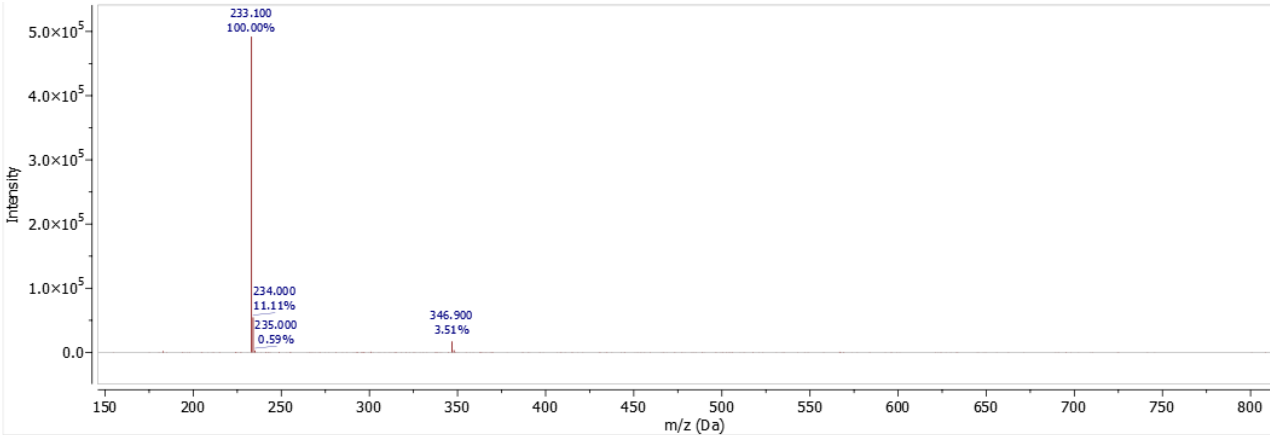

*Figure 53. Mass spectrum ESI- of compound **18d***

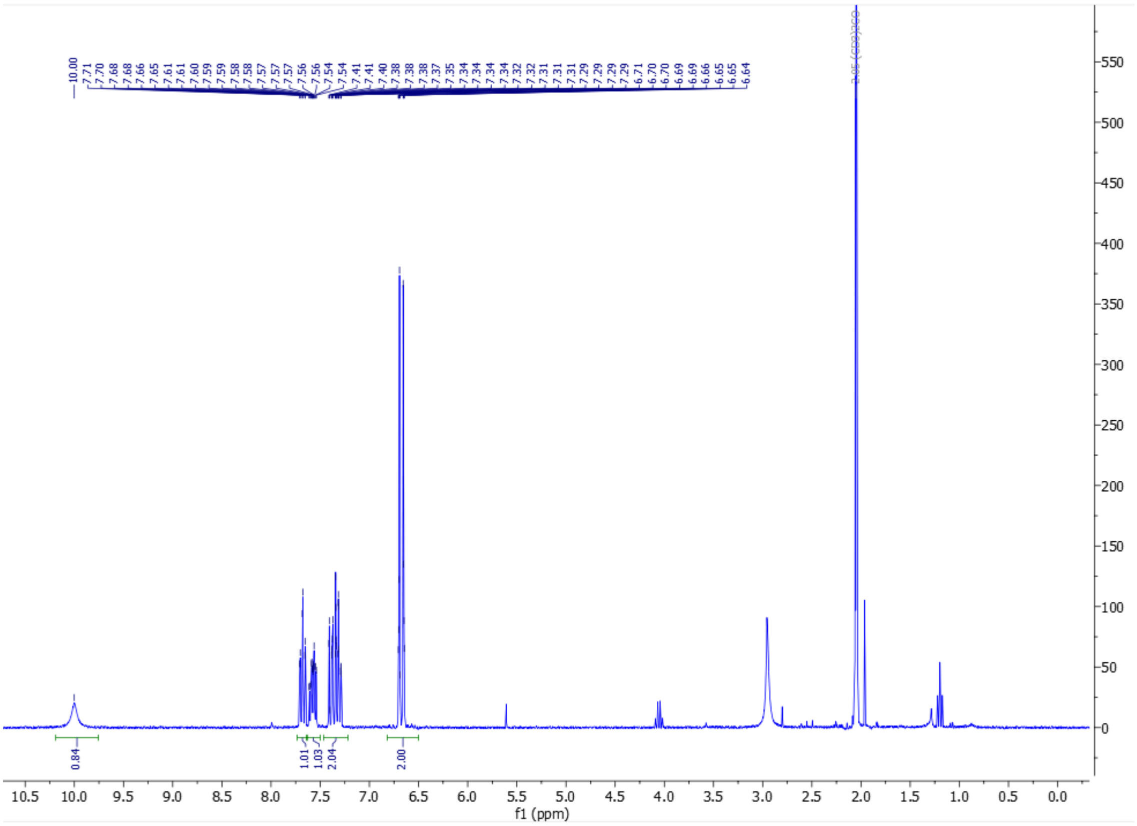

*Figure 54. ^{1}H NMR (300 MHz, acetone-d6) spectrum of compound **18e**.*

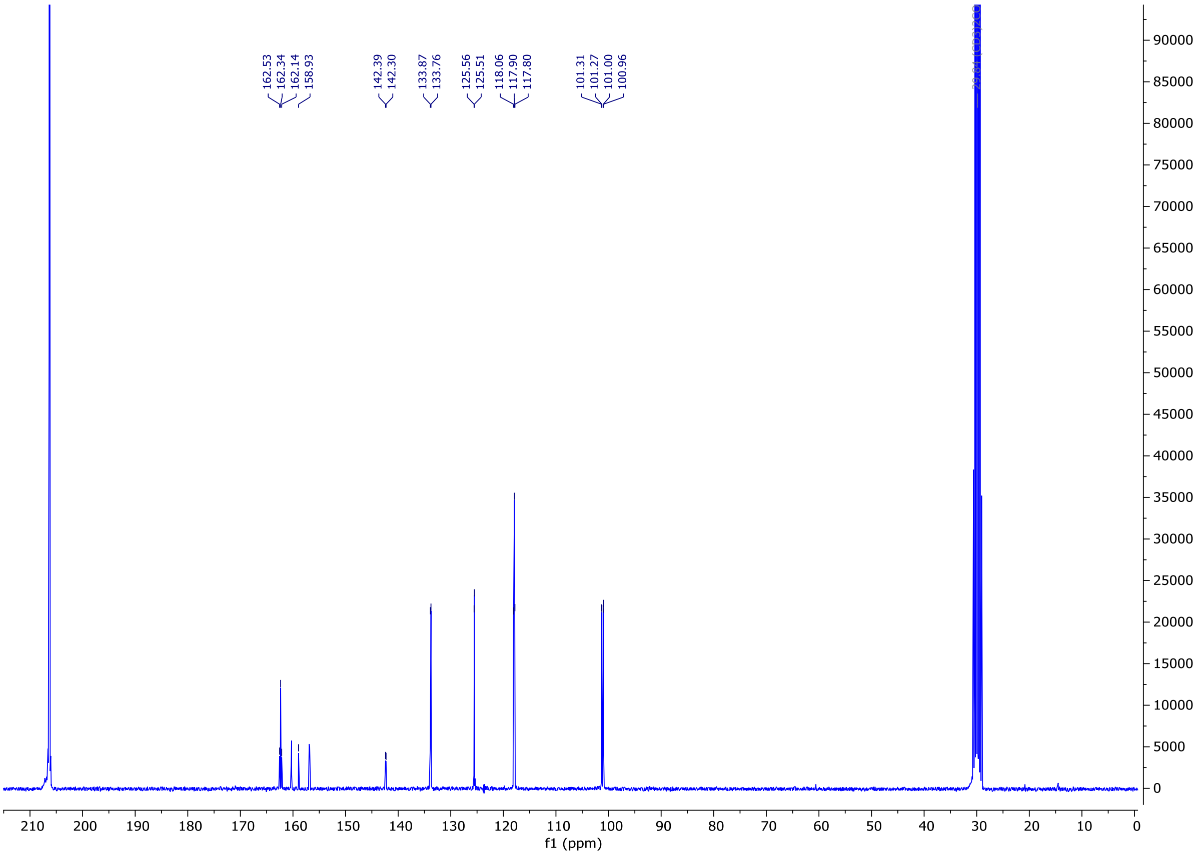

*Figure 55. ^{13}C NMR (75 MHz, acetone-d6) spectrum of compound **18e***

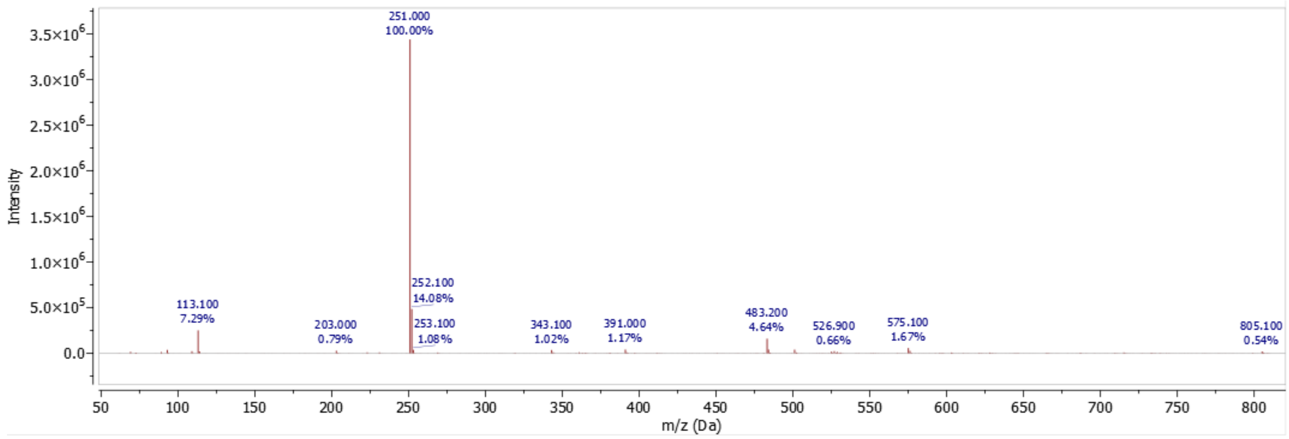

Figure 56. Mass spectrum ESI⁻ of compound ***18e***

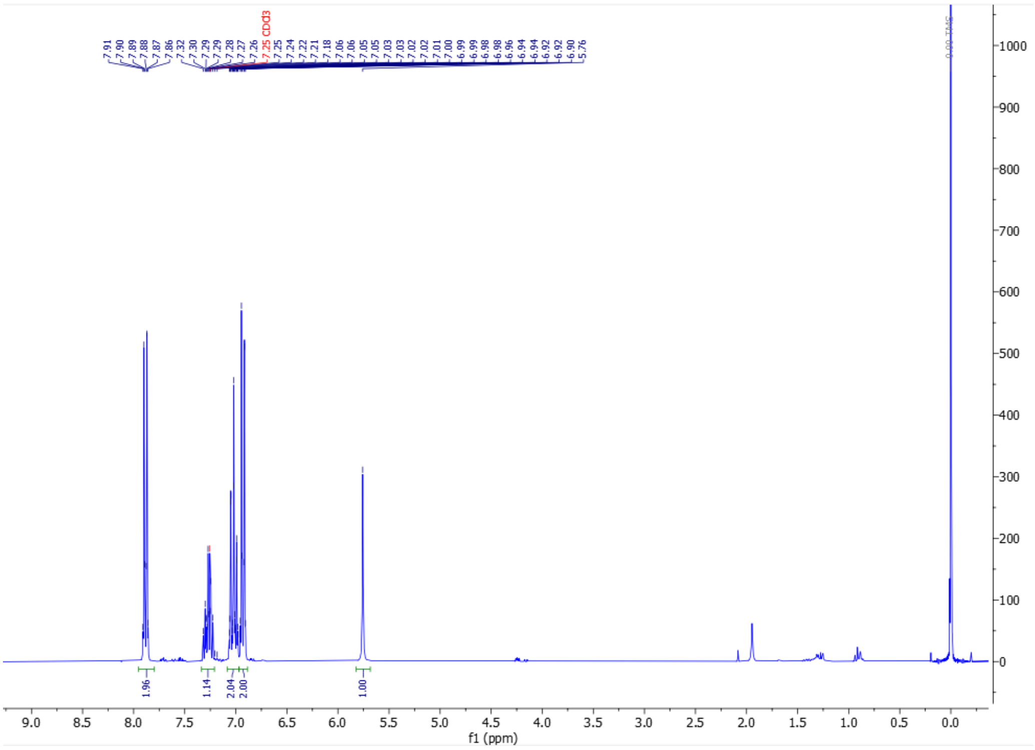

Figure 57. ^{1}*H NMR (300 MHz,* $CDCl_3$*) spectrum of compound* ***18f***

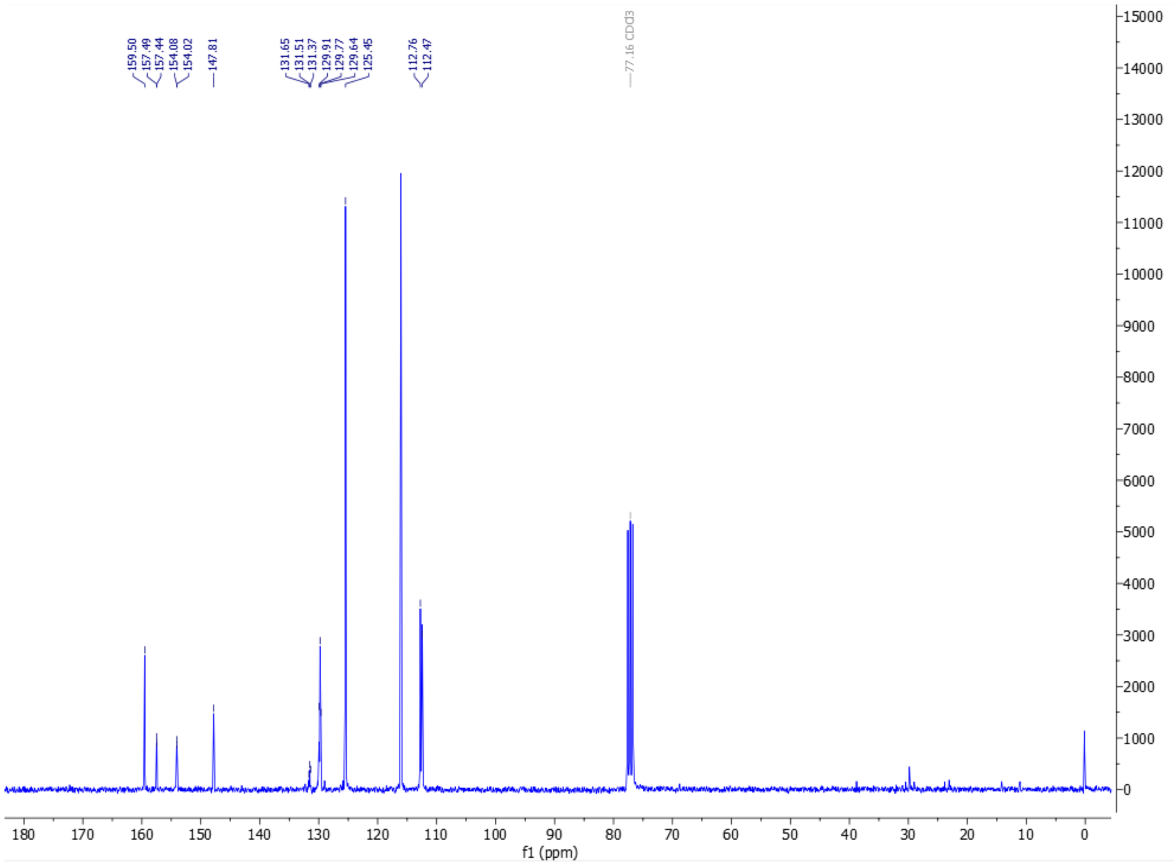

Figure 58. ^{13}C *NMR (75 MHz,* $CDCl_3$*) spectrum of compound* ***18f***

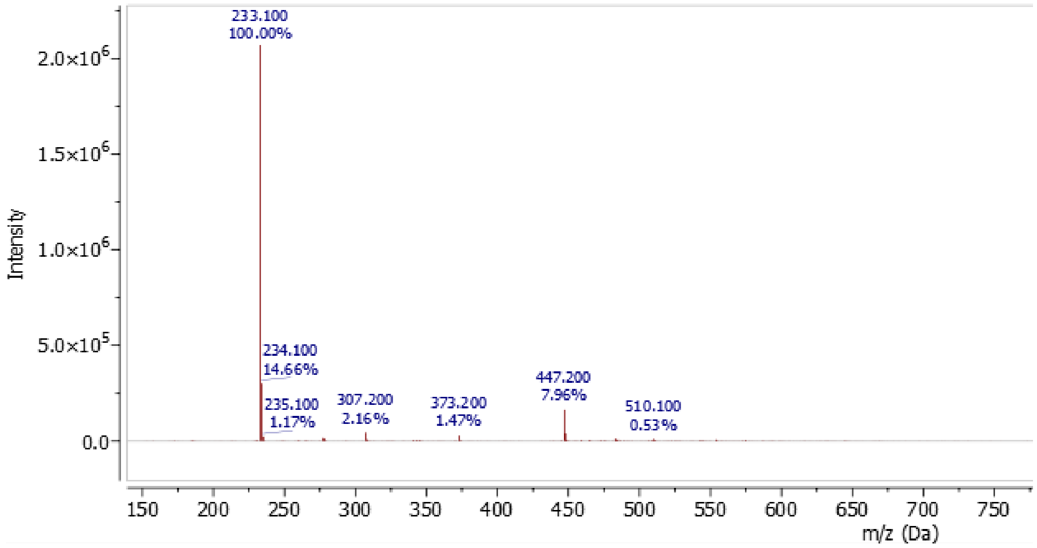

Figure 59. Mass spectrum ESI^{-} *of compound* ***18f***

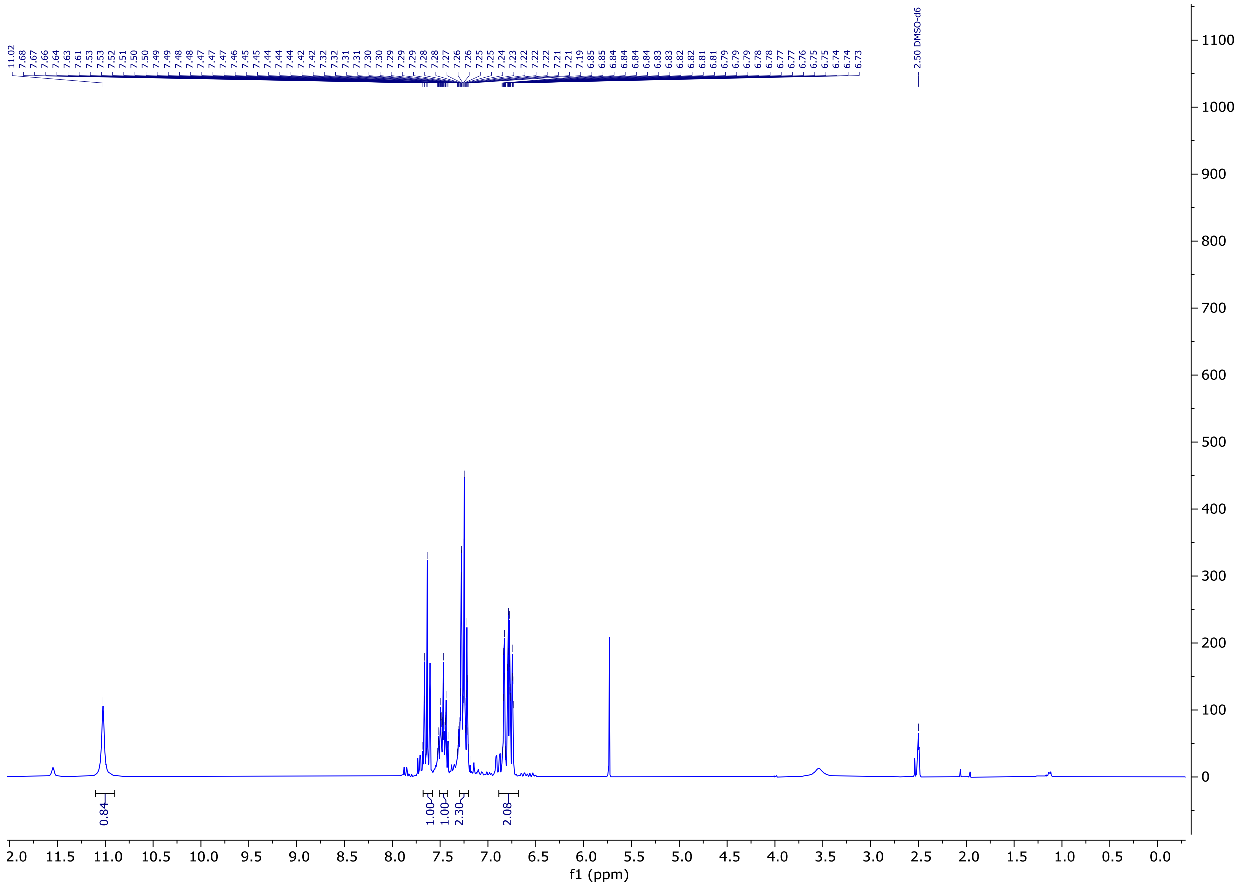

*Figure 60. ^{1}H NMR (300 MHz, DMSO-d6) spectrum of compound **18g***

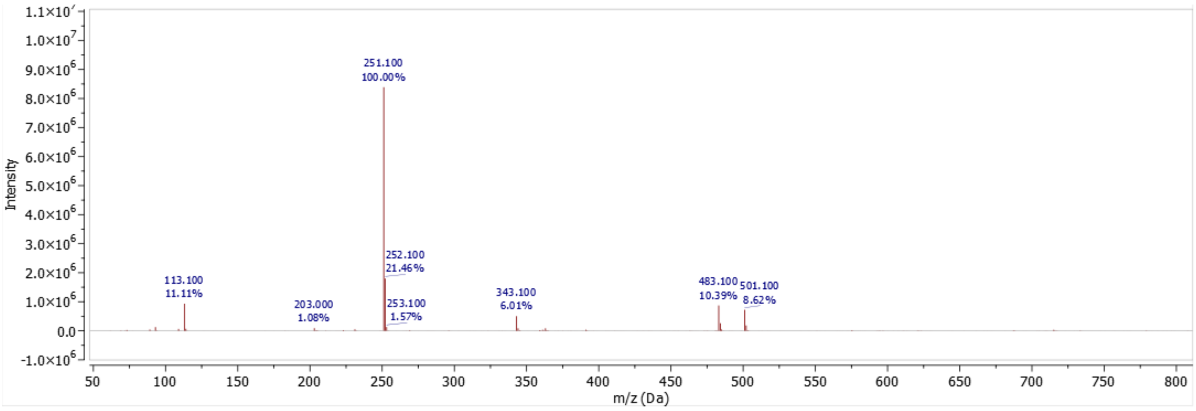

*Figure 61. Mass spectrum ESI- of compound **18g***

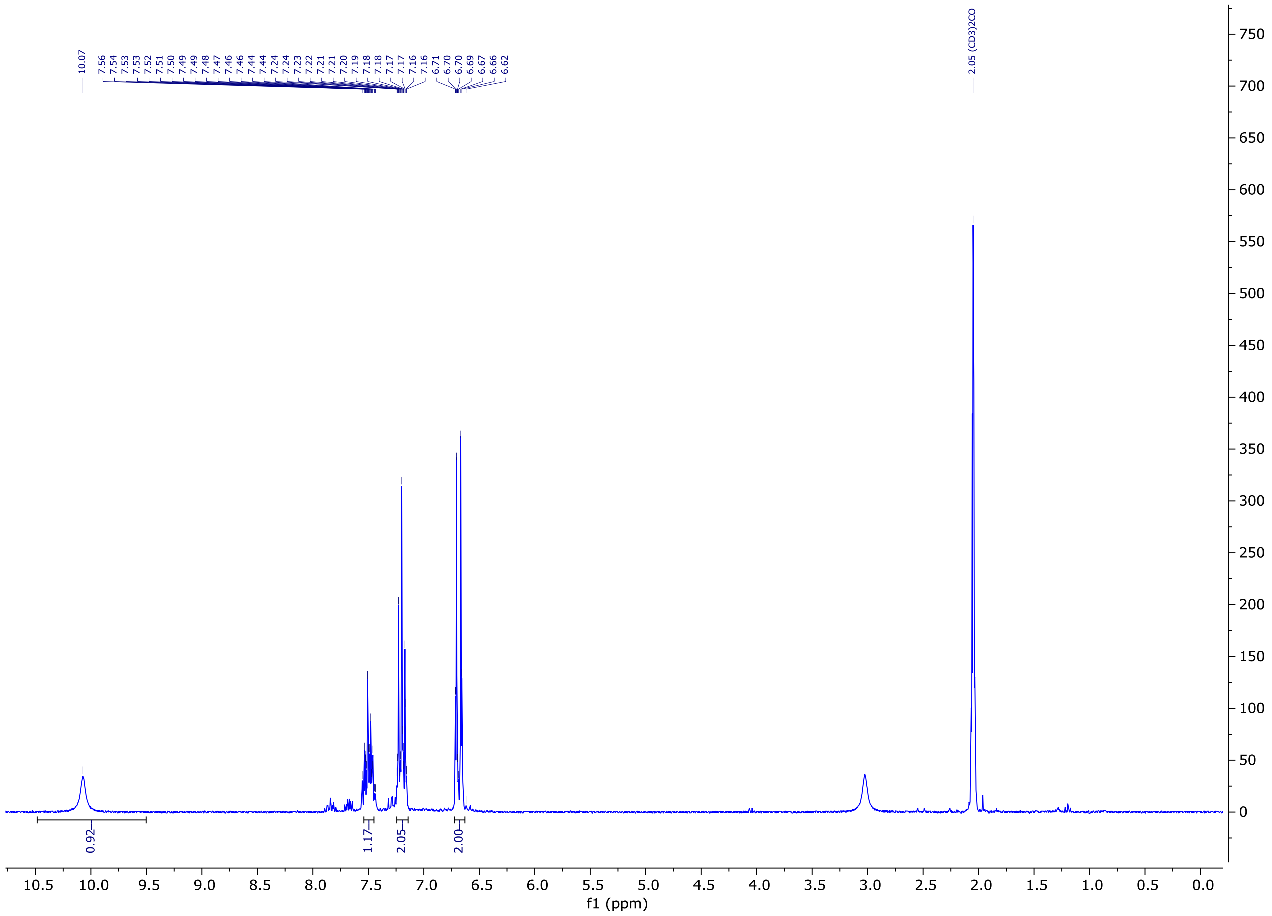

Figure 62. ^{1}H NMR (300 MHz, acetone-d6) spectrum of compound **18h**

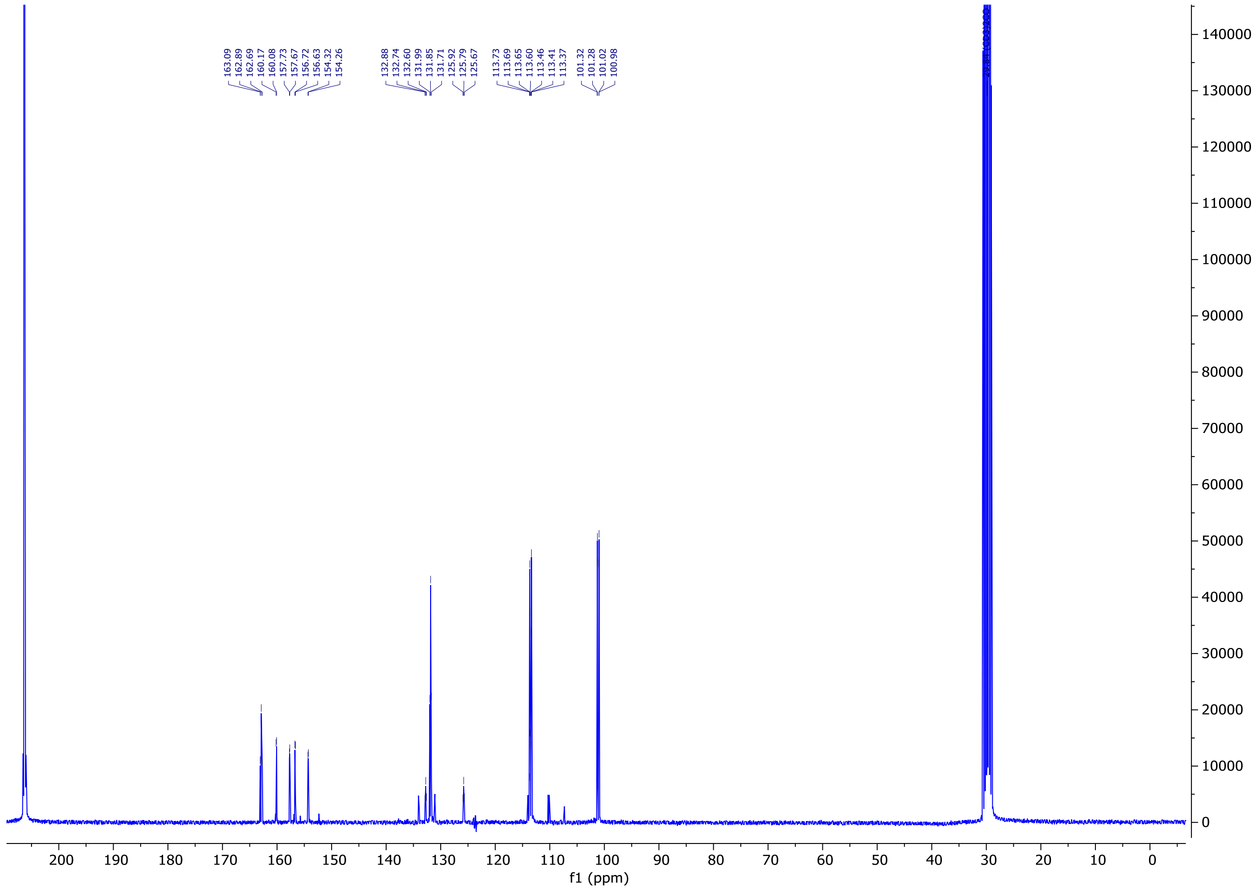

Figure 63. ^{13}C NMR (75 MHz, acetone-d6) spectrum of compound **18h**

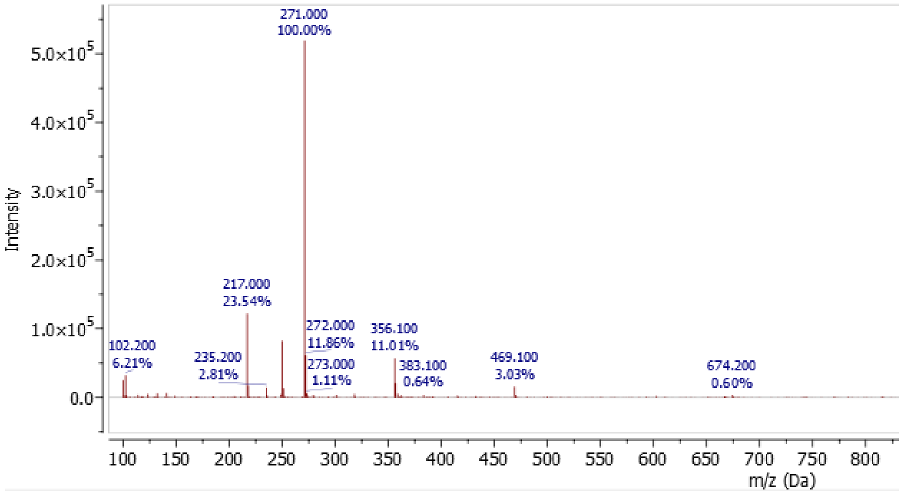

*Figure 64. Mass spectrum ESI^{+} of compound **18h***

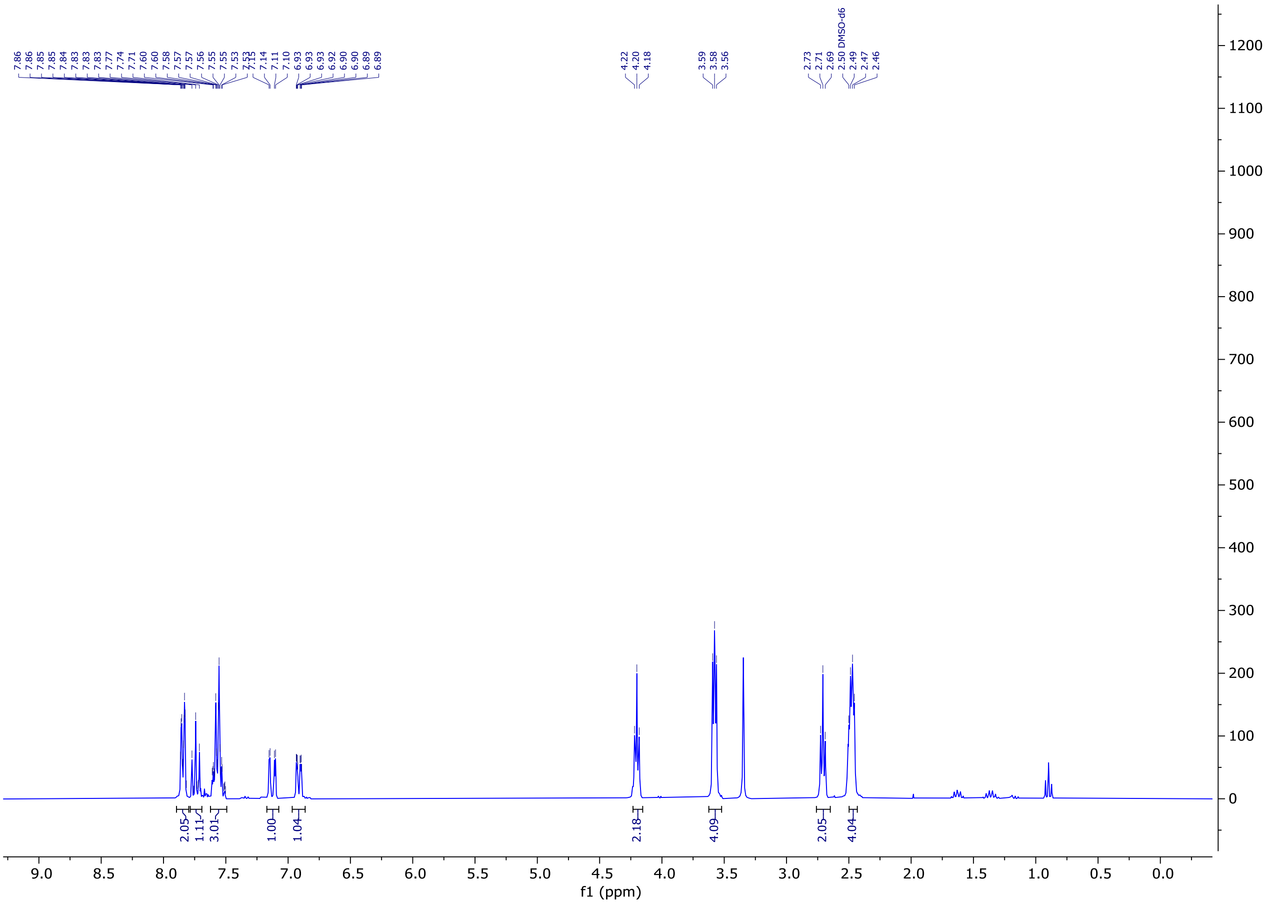

*Figure 65. ^{1}H NMR (300 MHz, DMSO-d6) spectrum of compound **19a***

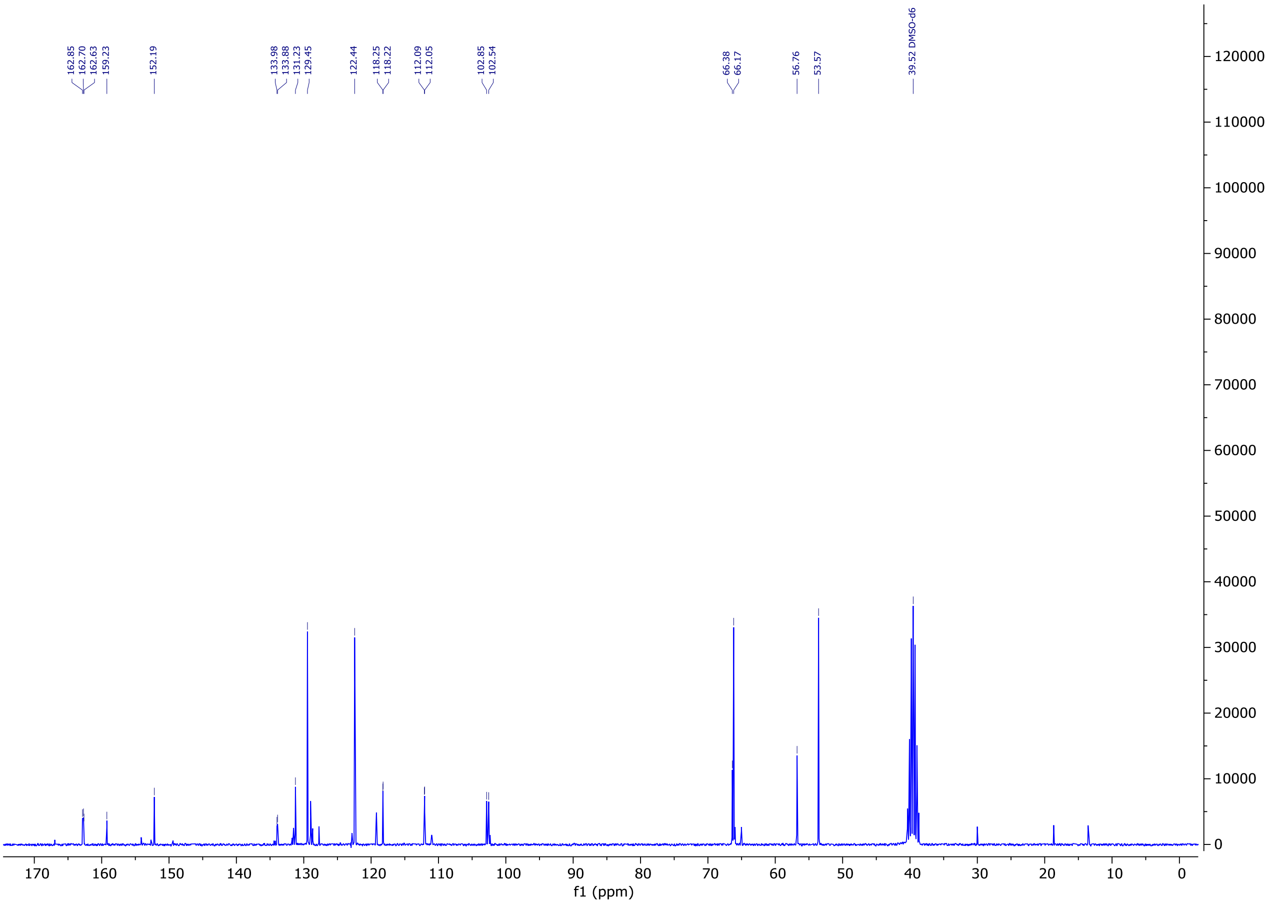

Figure 66. ^{13}C NMR (75 MHz, DMSO-d6) spectrum of compound ***19a***

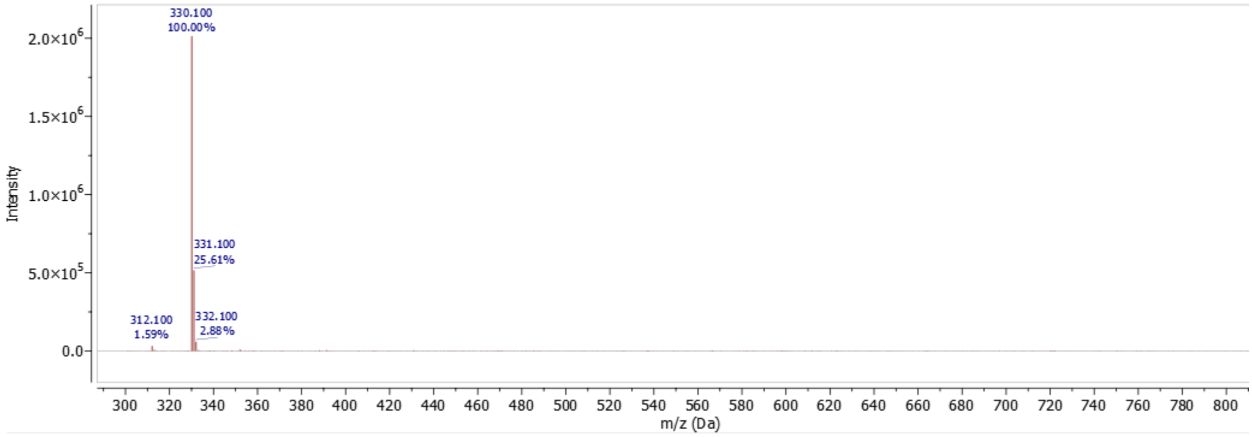

Figure 67. Mass spectrum ESI^{+} of compound ***19a***

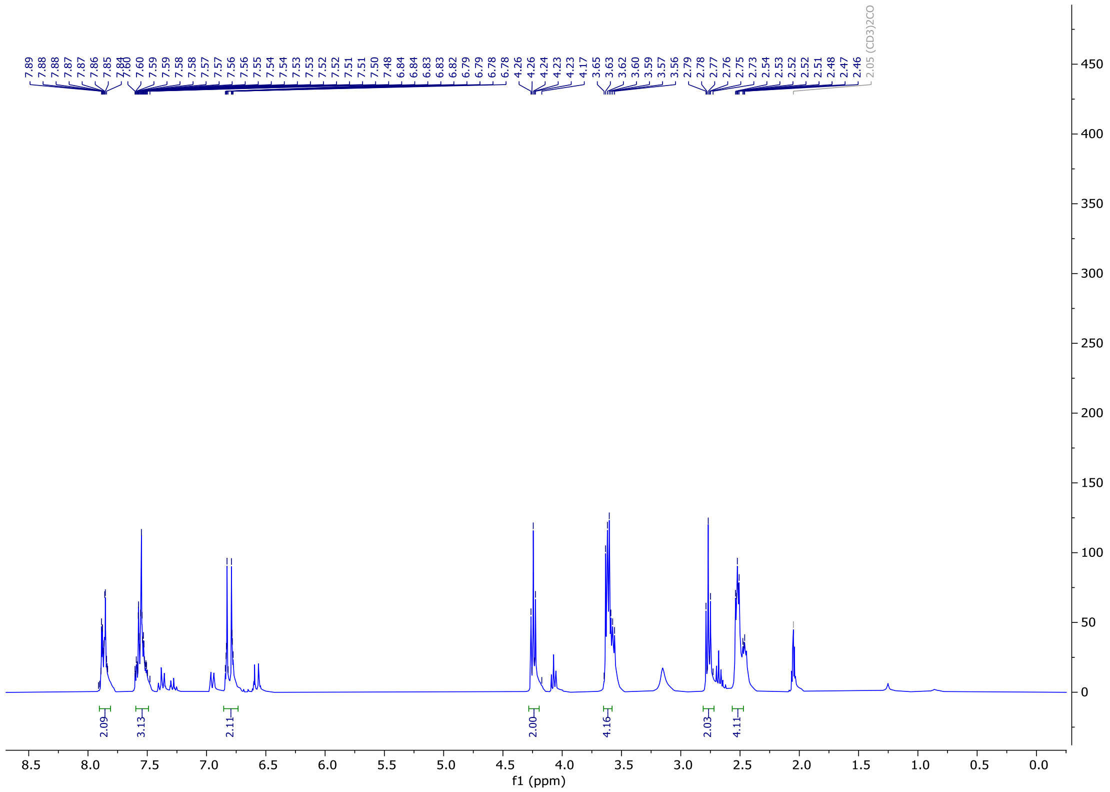

*Figure 68. ^{1}H NMR (300 MHz, acetone-d6) spectrum of compound **19b***

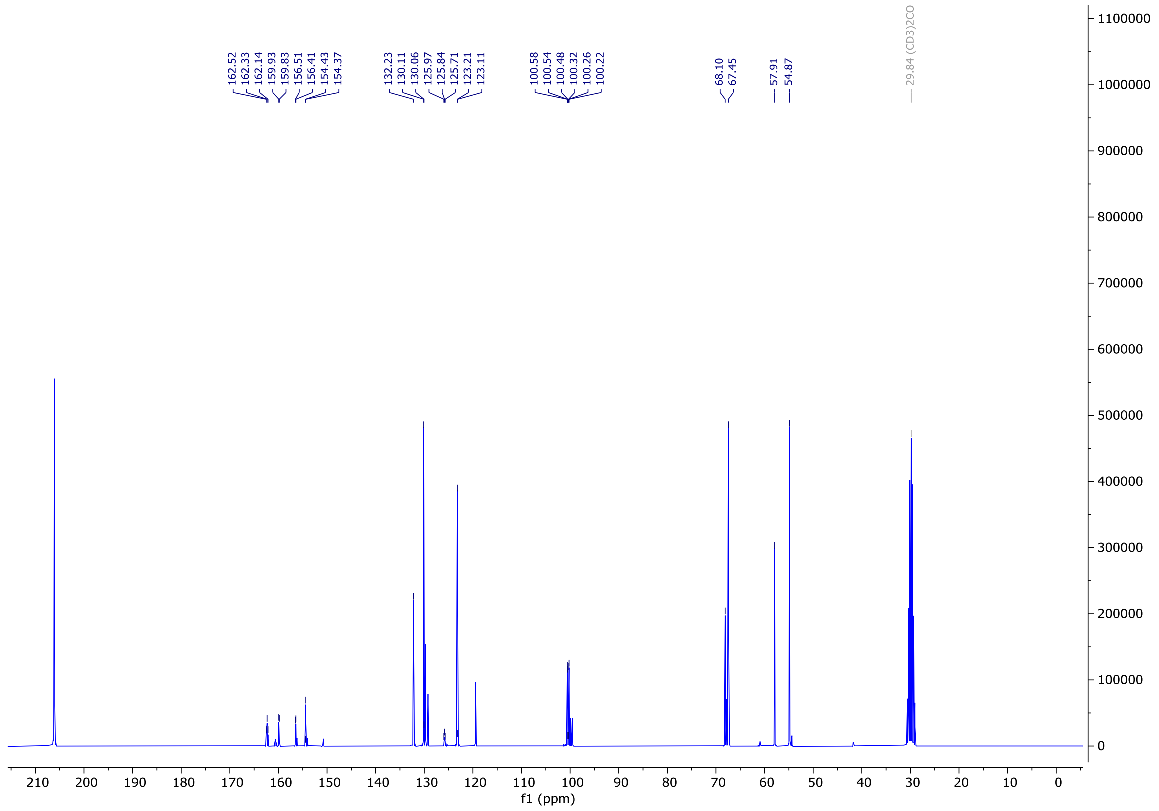

*Figure 69. ^{13}C NMR (75 MHz, acetone-d6) spectrum of compound **19b***

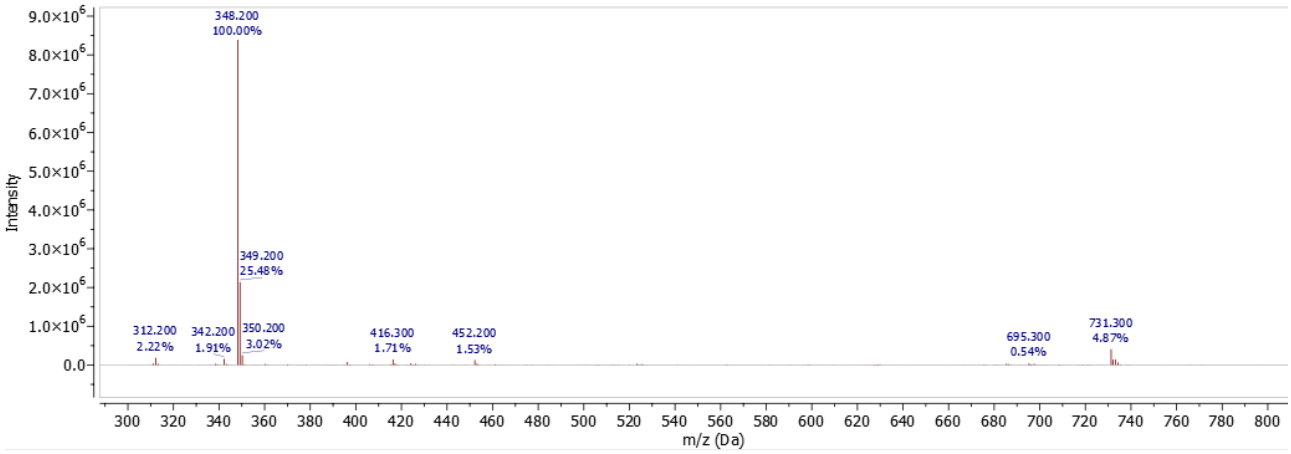

Figure 70. Mass spectrum ESI$^+$ of compound **19b**

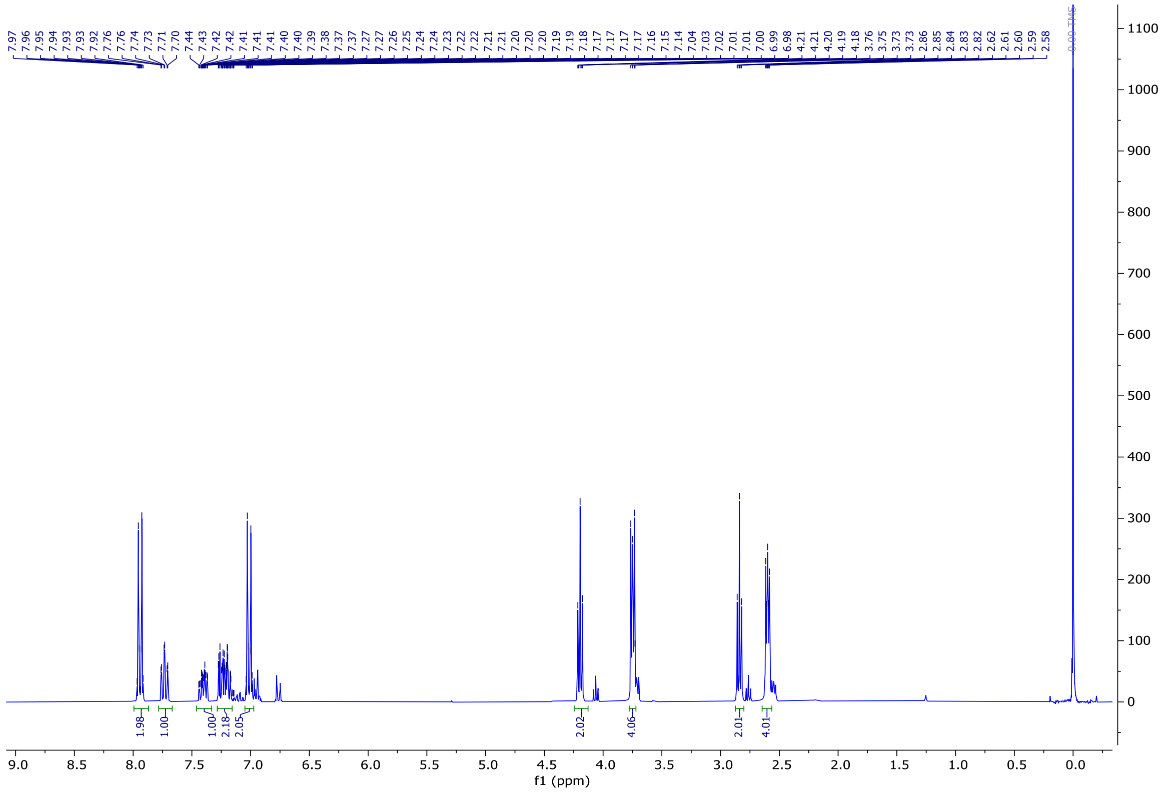

Figure 71. ^{1}H NMR (300 MHz, $CDCl_3$) spectrum of compound **19c**

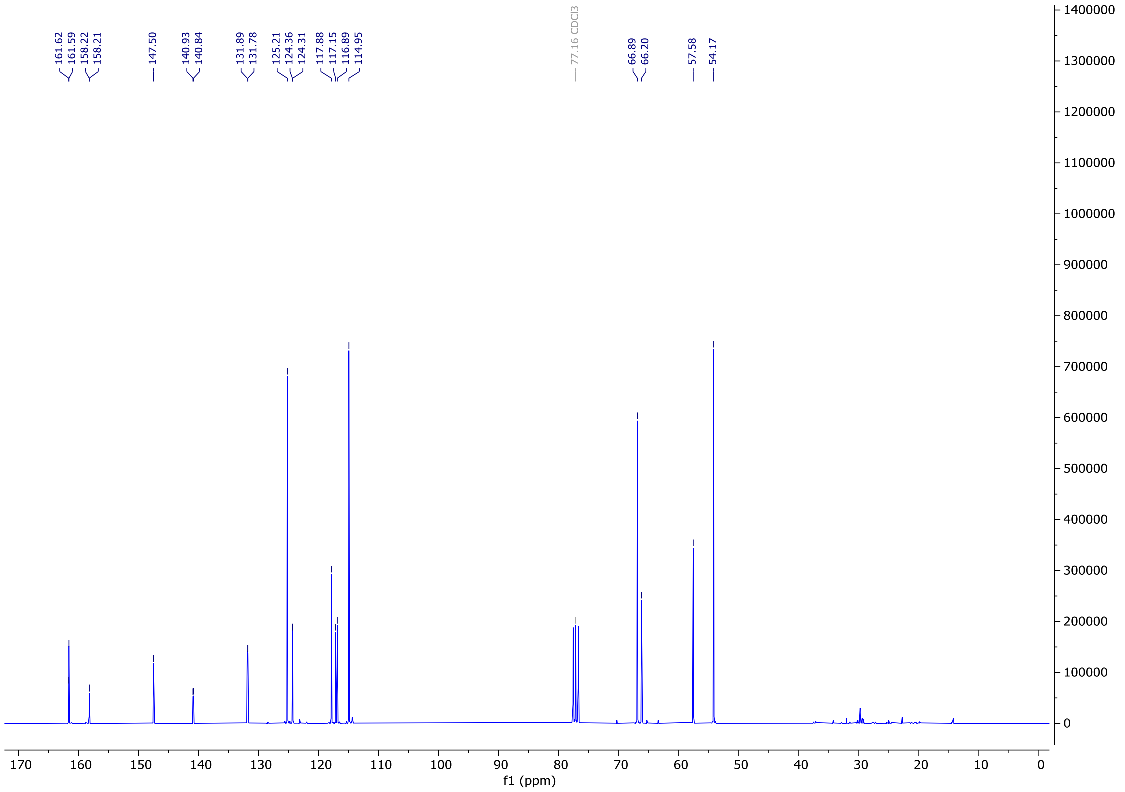

Figure 72. ^{13}C NMR (75 MHz, $CDCl_3$) spectrum of compound ***19c***

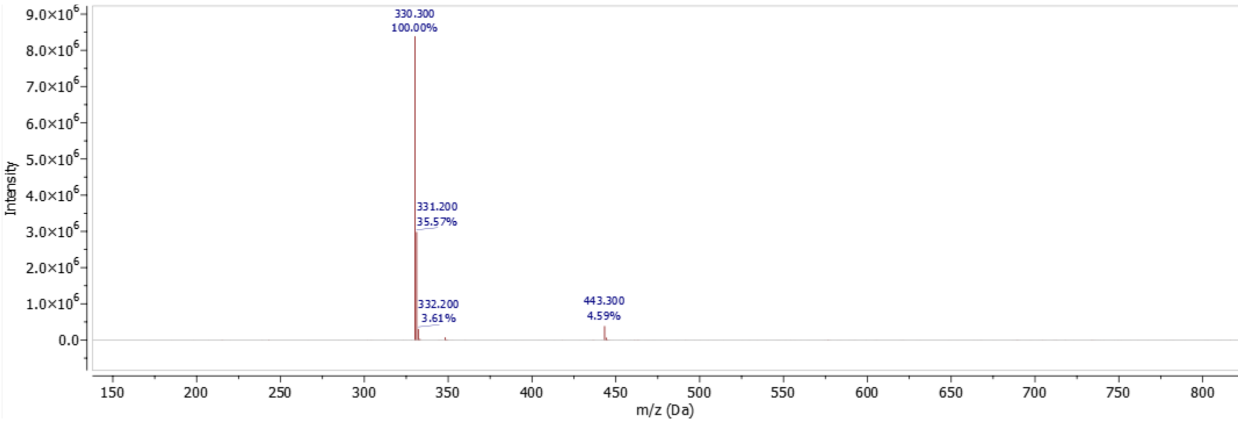

Figure 73. Mass spectrum ESI$^+$ of compound ***19c***

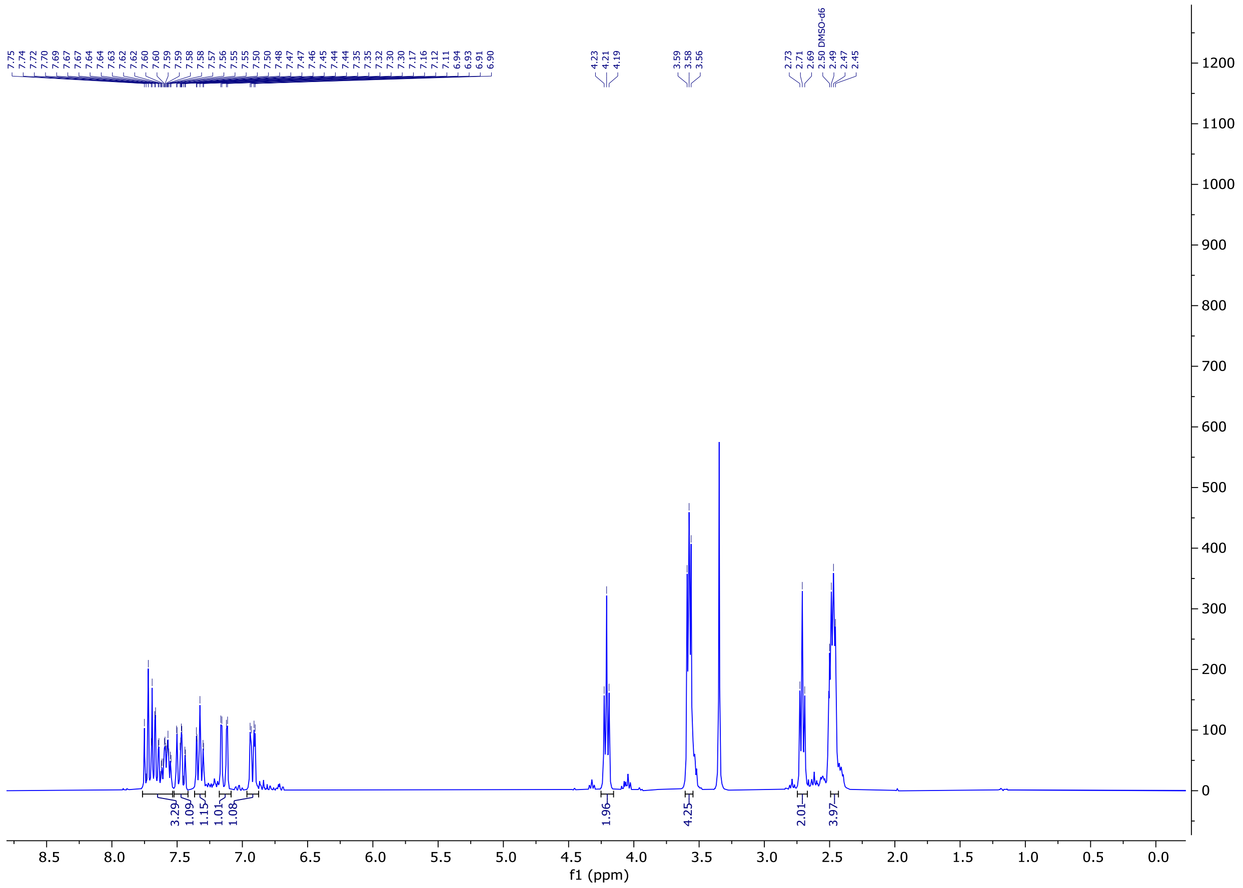

Figure 74. ^{1}H NMR (300 MHz, DMSO-d6) spectrum of compound ***19d***

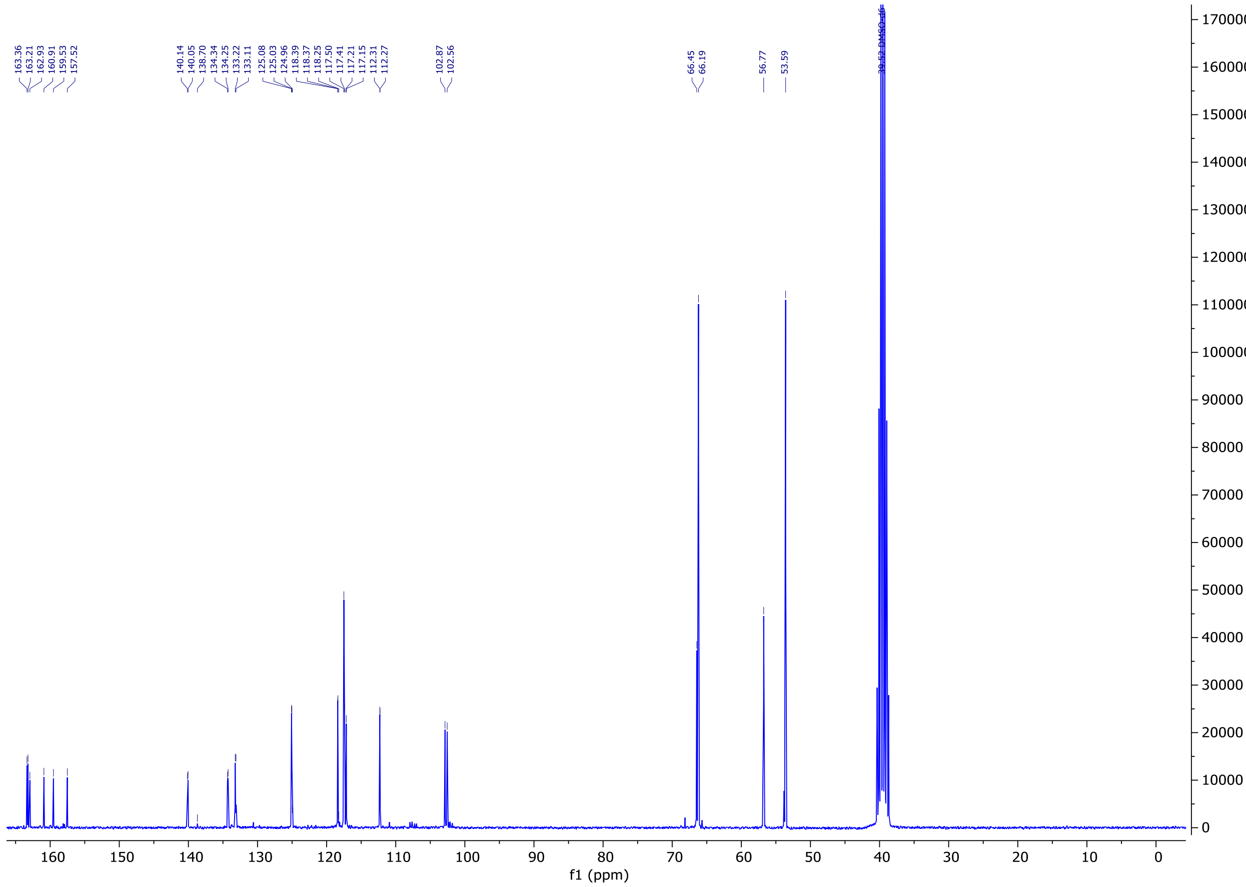

Figure 75. ^{13}C NMR (75 MHz, DMSO-d6) spectrum of compound ***19d***

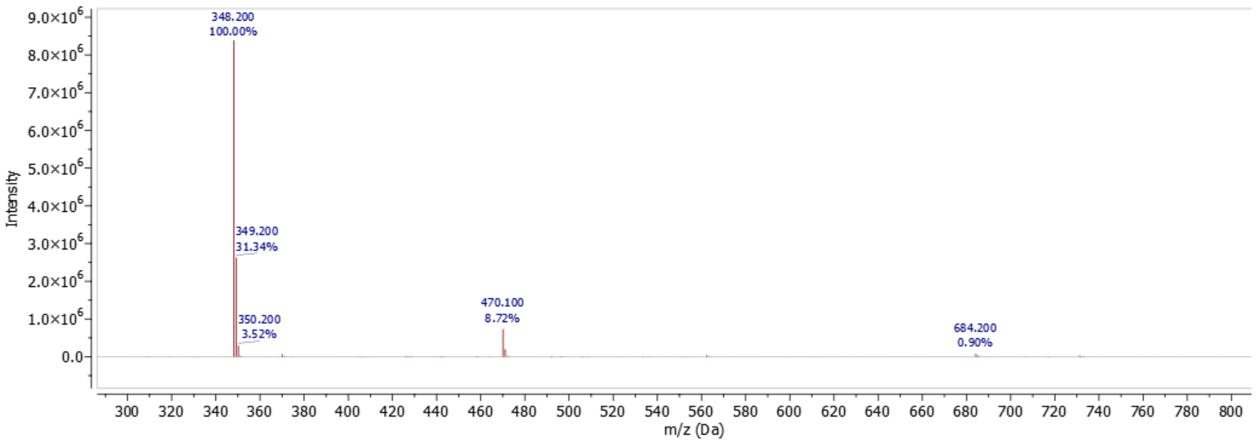

Figure 76. Mass spectrum ESI⁺ of compound ***19d***

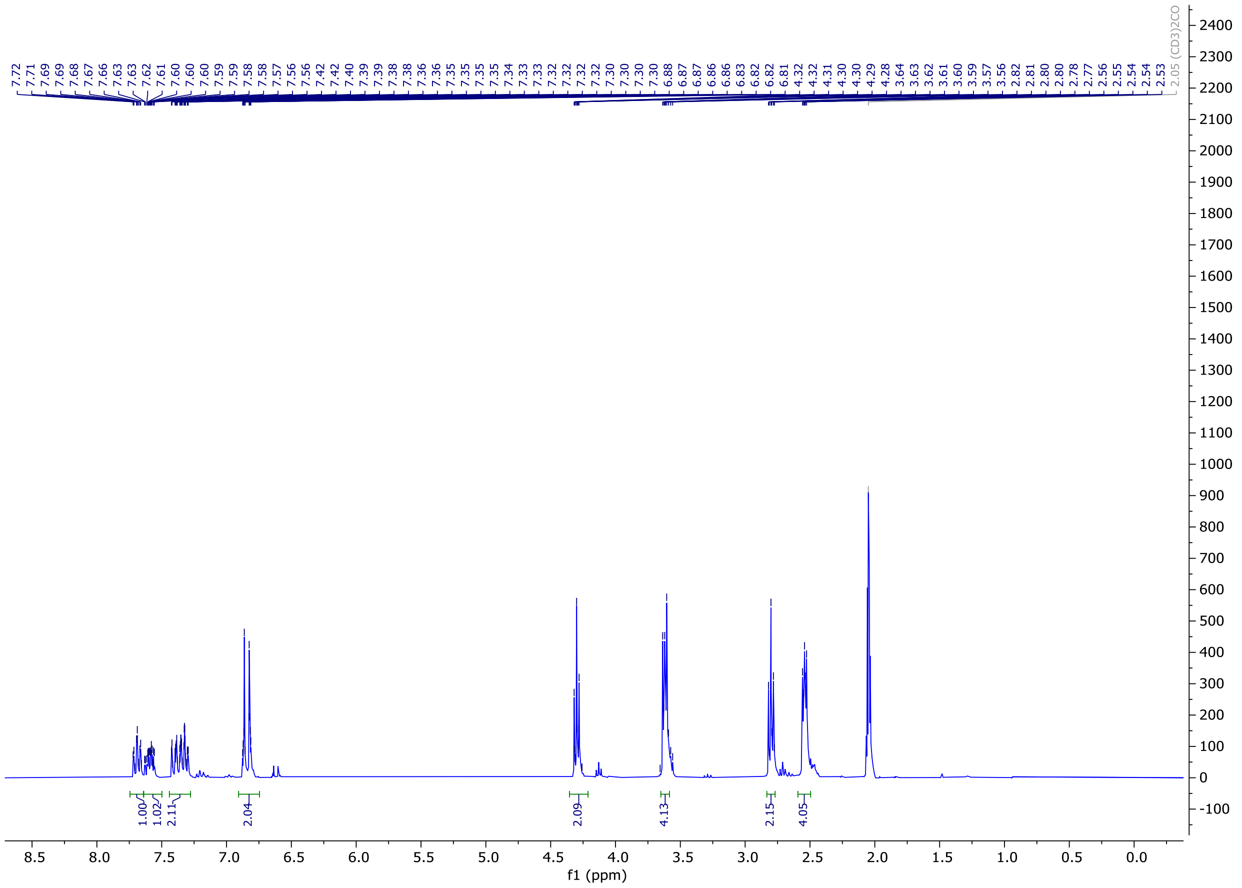

Figure 77. ^{1}H NMR (300 MHz, acetone-d6) spectrum of compound ***19e***

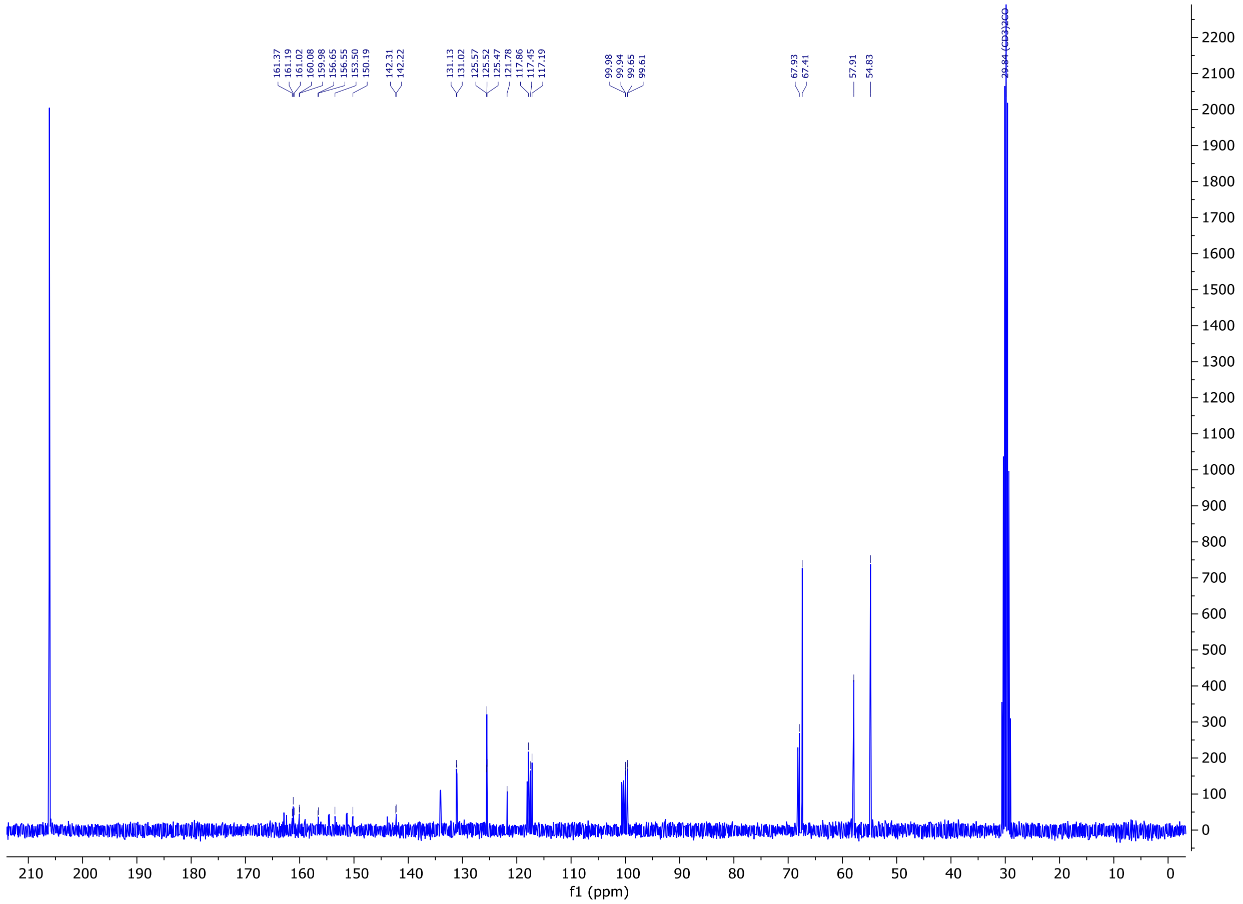

Figure 78. *^{13}C NMR (300 MHz, acetone-d6) spectrum of compound* ***19e***

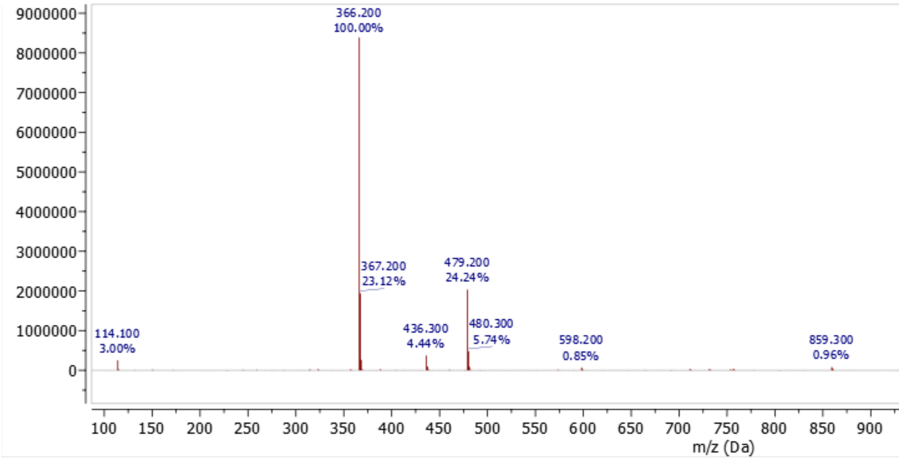

Figure 79. Mass spectrum ESI[+] of compound ***19e***

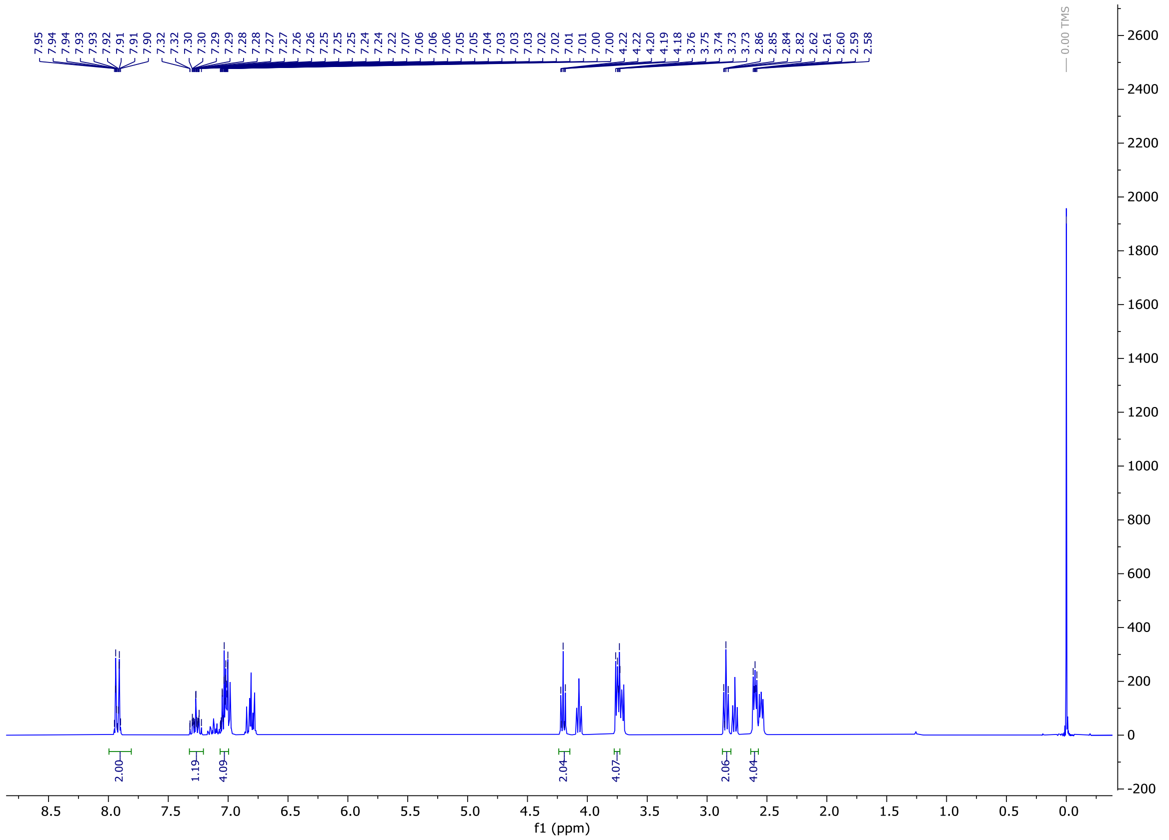

Figure 80. ^{1}H NMR (300 MHz, $CDCl_3$) spectrum of compound **19f**

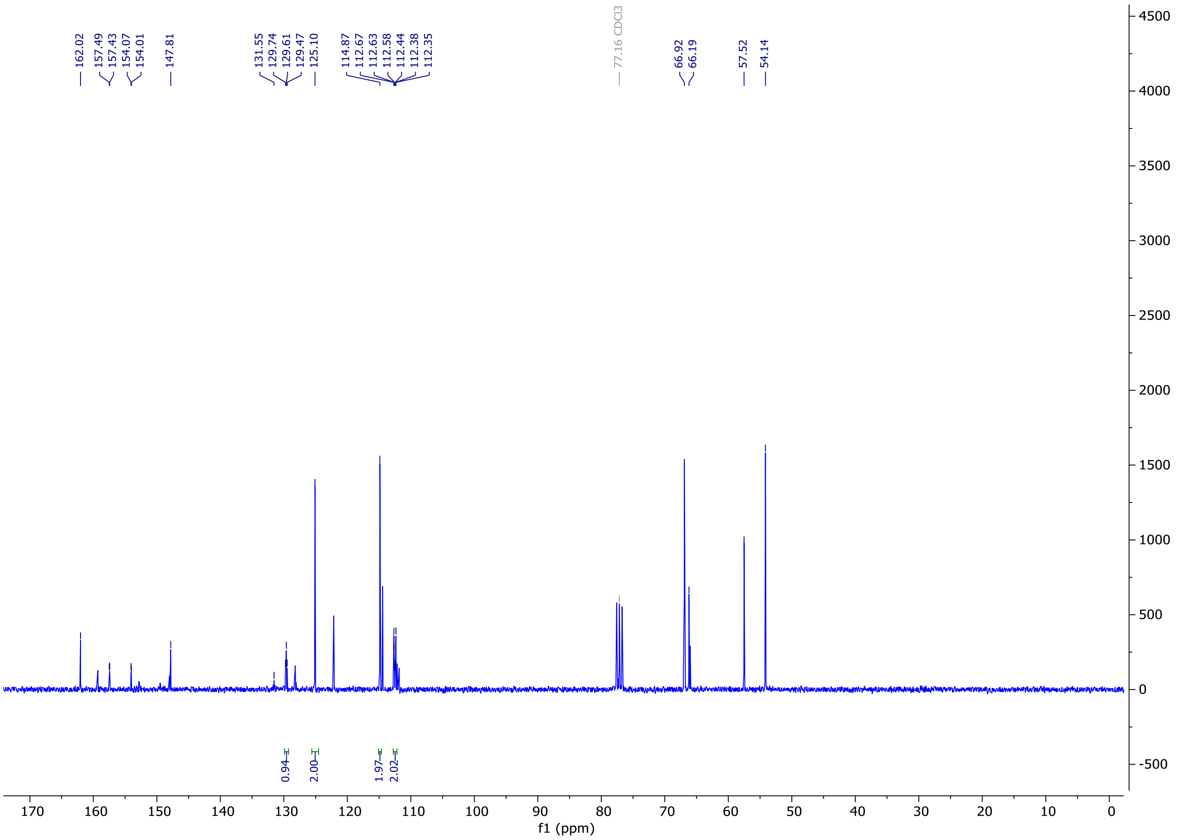

Figure 81. ^{13}C NMR (75 MHz, $CDCl_3$) spectrum of compound **19f**

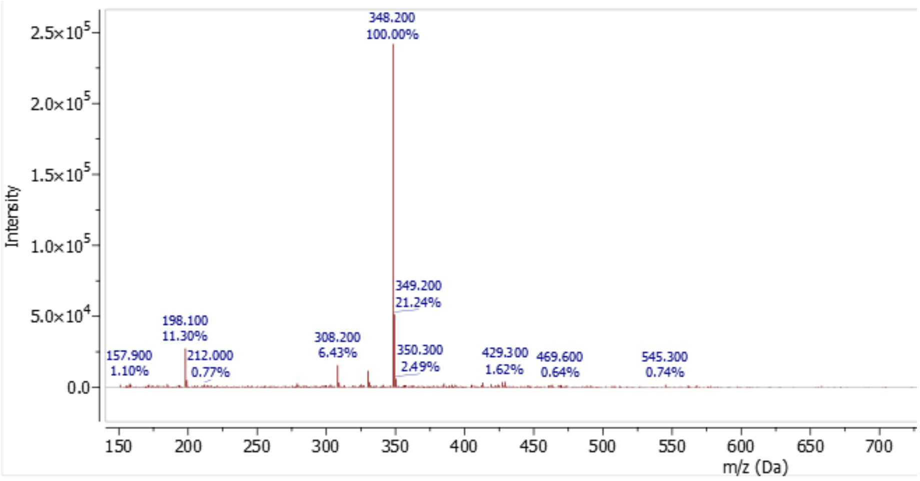

*Figure 82. Mass spectrum ESI⁺ of compound **19f***

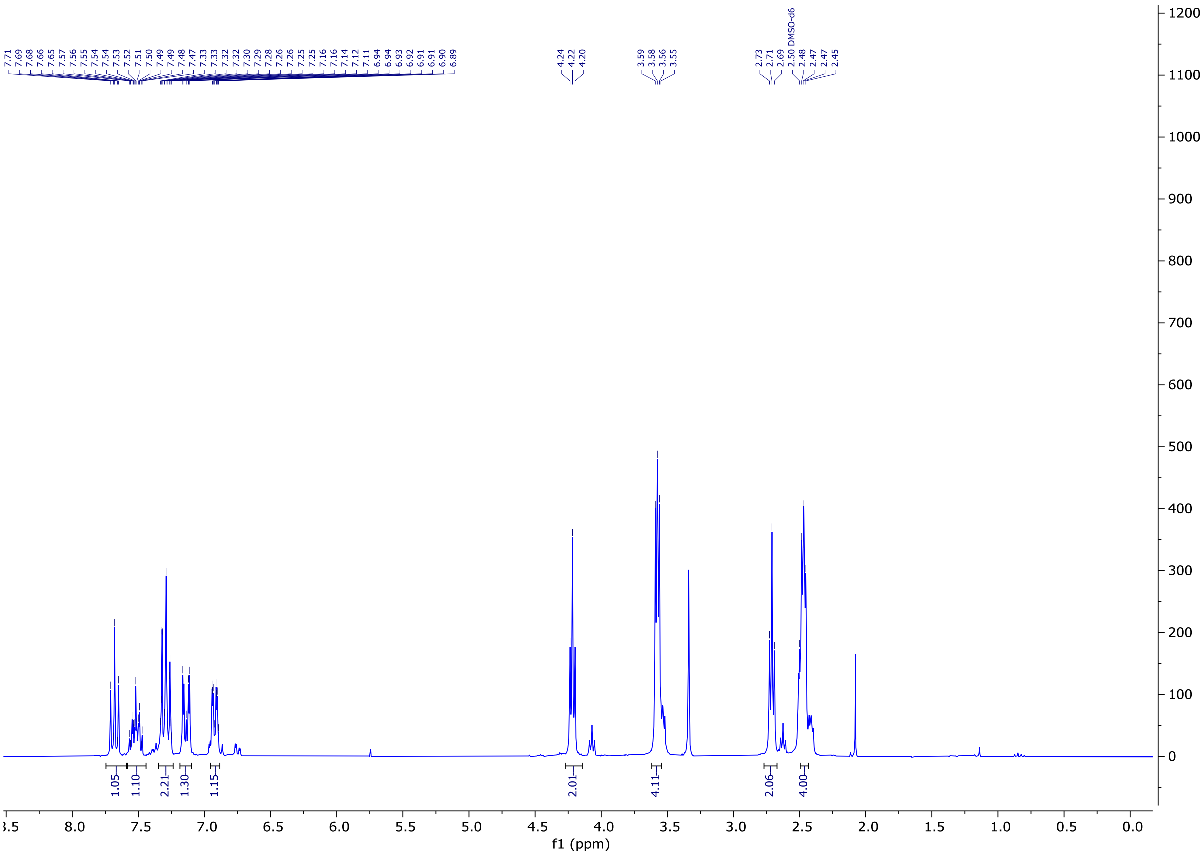

*Figure 83. ¹H NMR (300 MHz, DMSO-d6) spectrum of compound **19g***

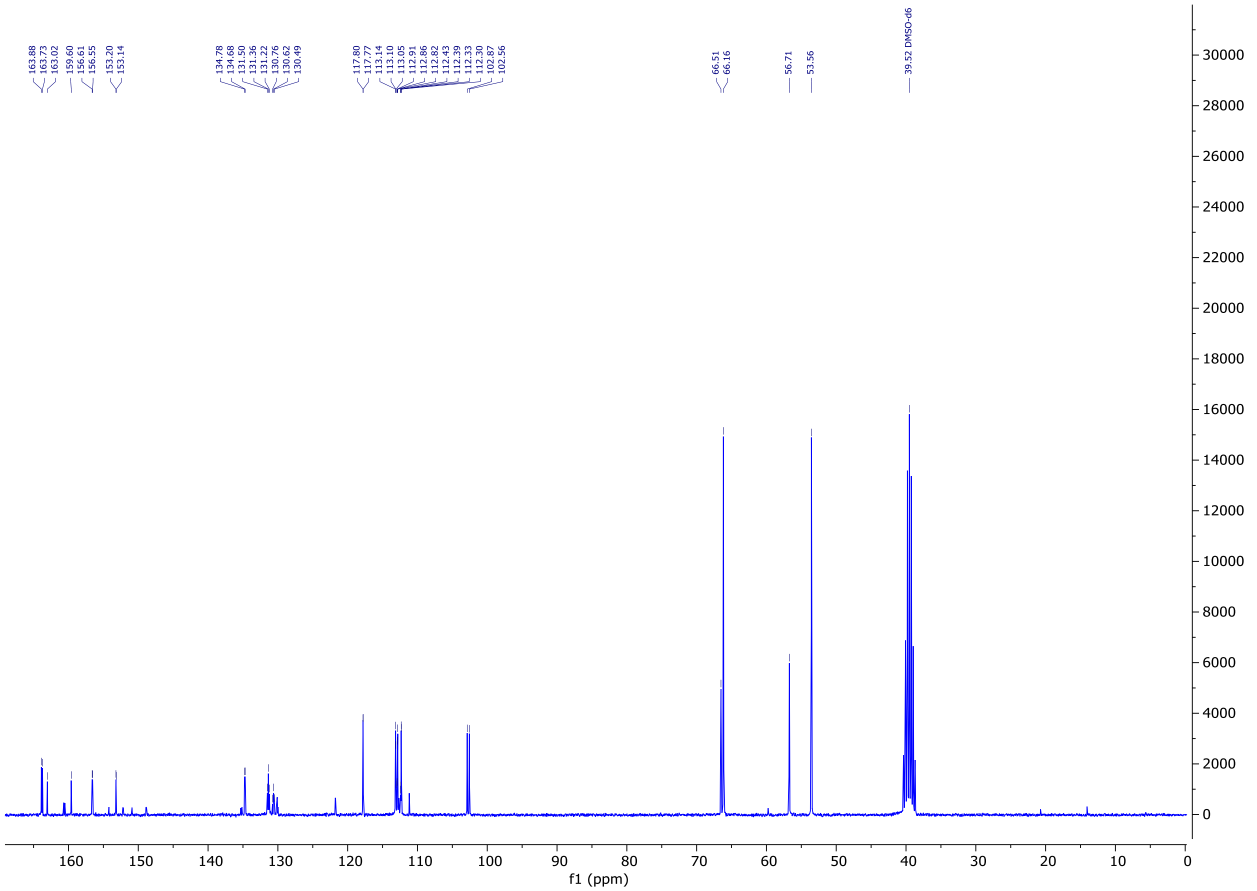

*Figure 84. ^{13}C NMR (75 MHz, DMSO-d6) spectrum of compound **19g***

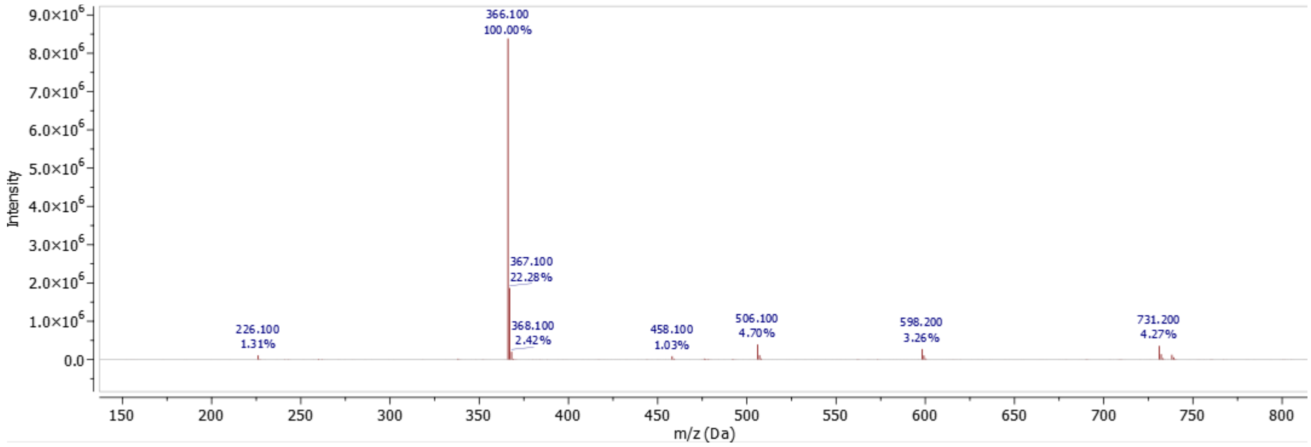

*Figure 85. Mass spectrum ESI$^+$ of compound **19g***

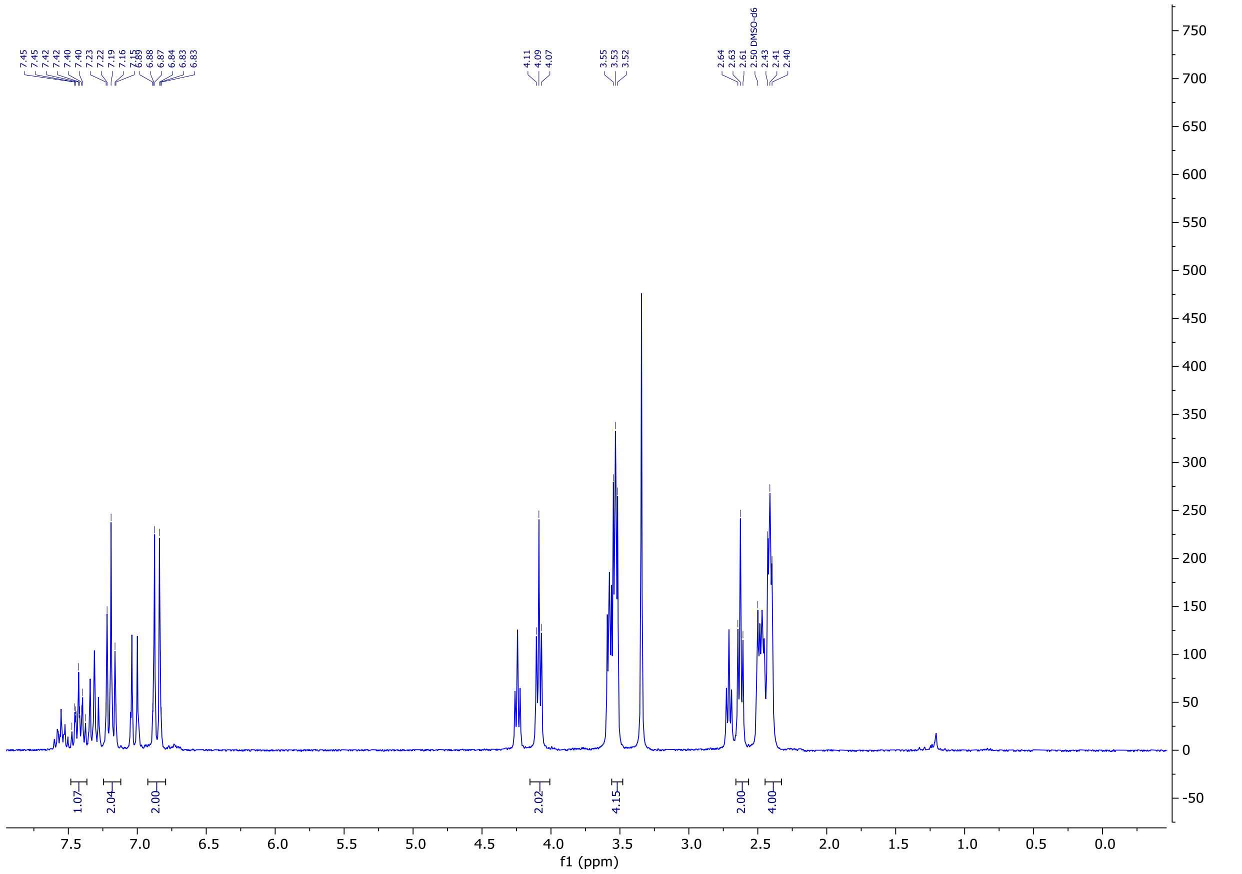

Figure 86. ^{1}H NMR (300 MHz, DMSO-d6) spectrum of compound ***19h***

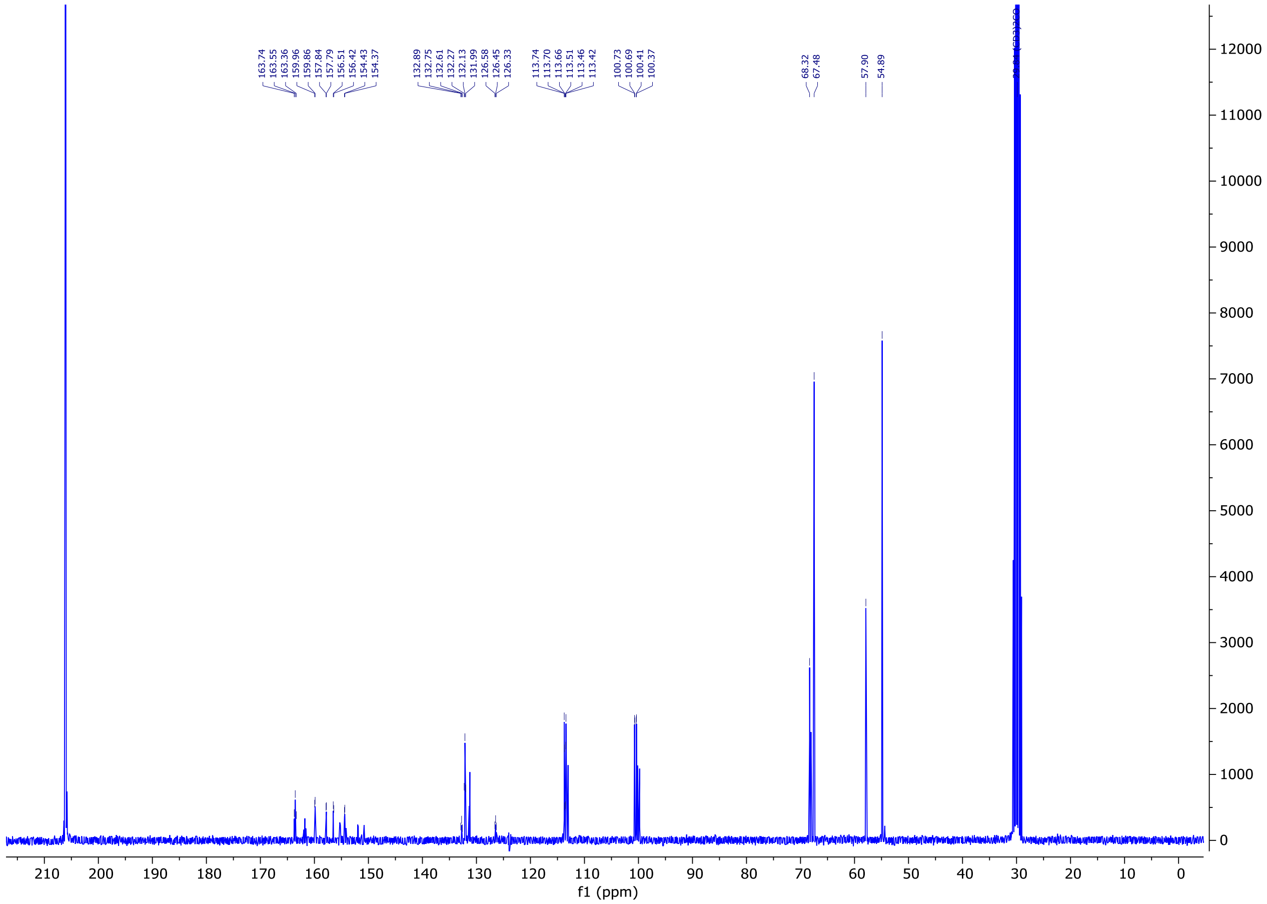

Figure 87. ^{13}C NMR (75 MHz, acetone-d6) spectrum of compound ***19h***

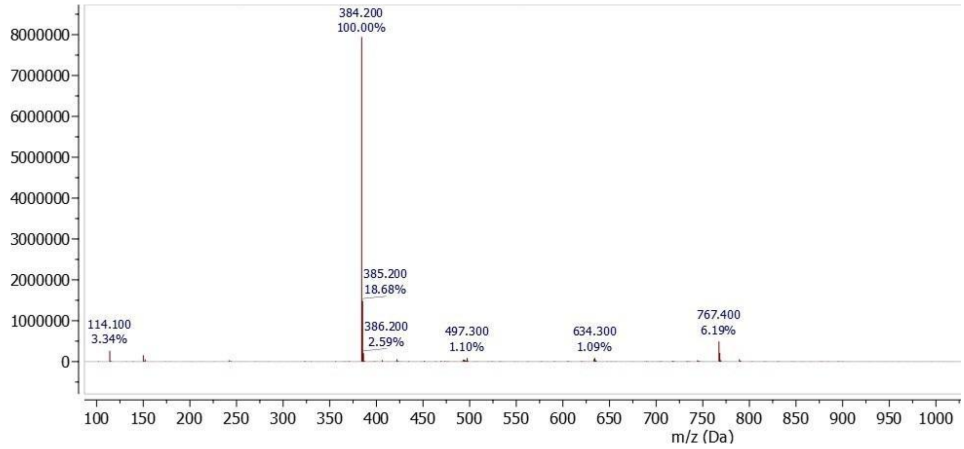

*Figure 88. Mass spectrum ESI⁺ of compound **19h***

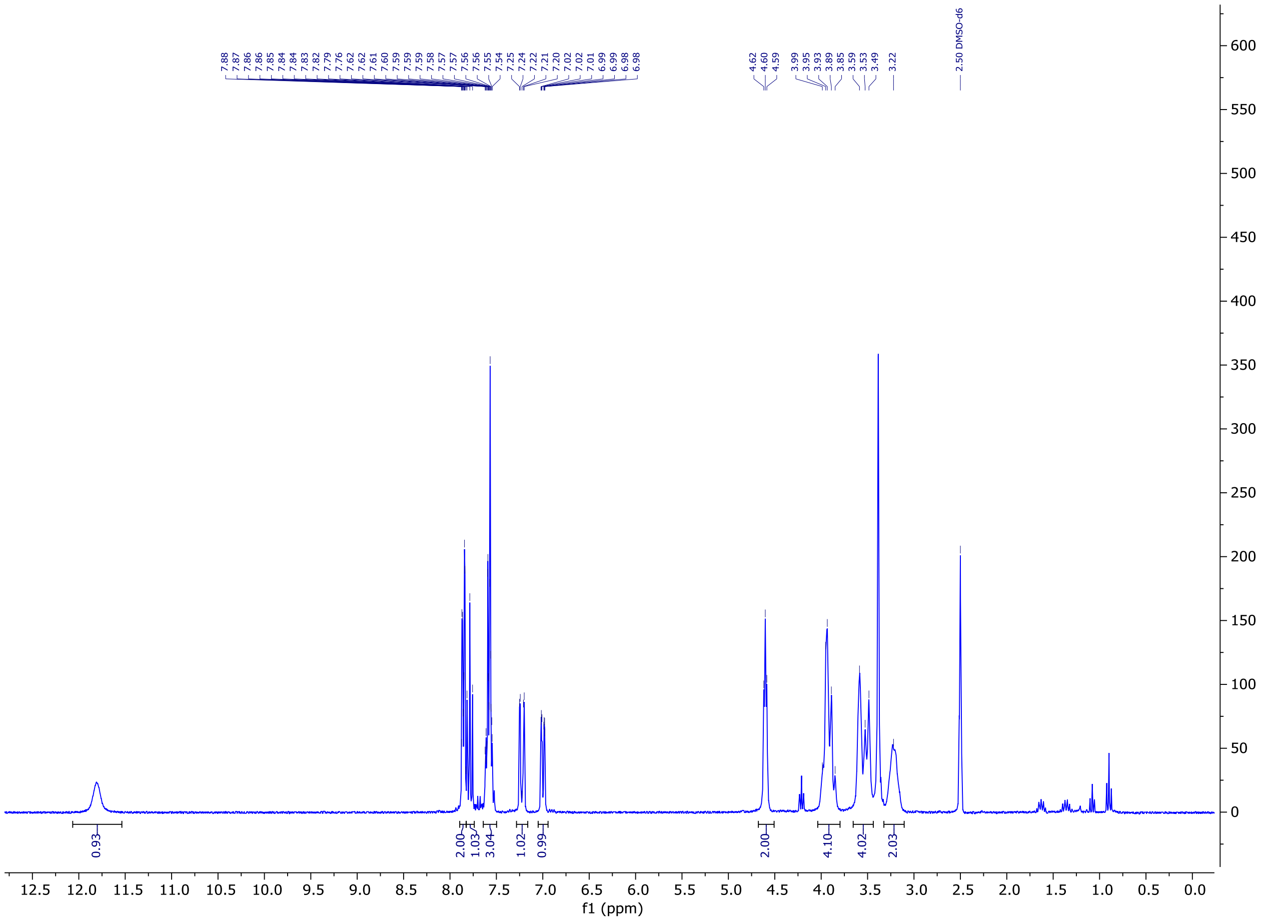

*Figure 89. ^{1}H NMR (300 MHz, DMSO-d6) spectrum of compound **20a***

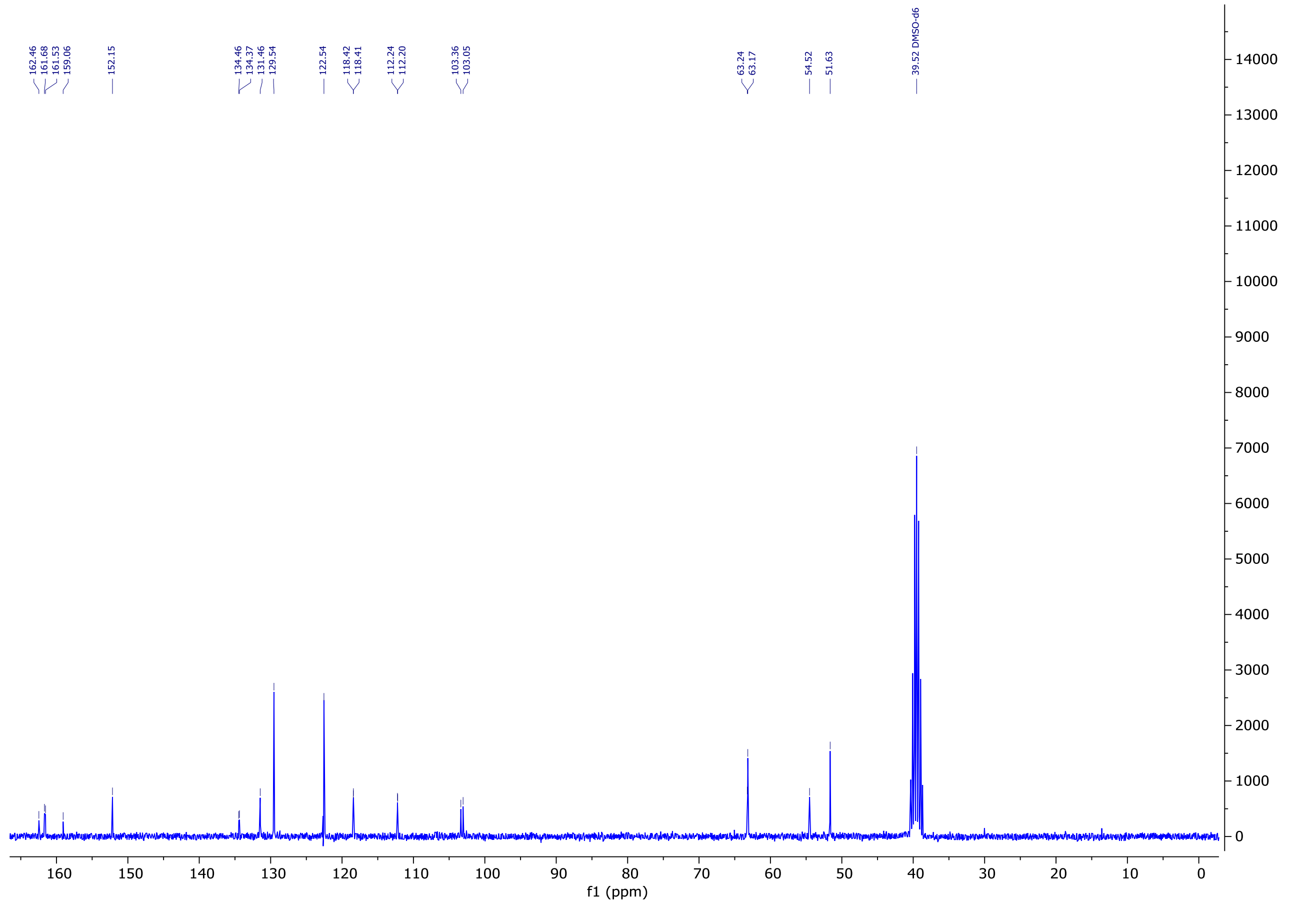

*Figure 90. ^{13}C NMR (75 MHz, DMSO-d6) spectrum of compound **20a***

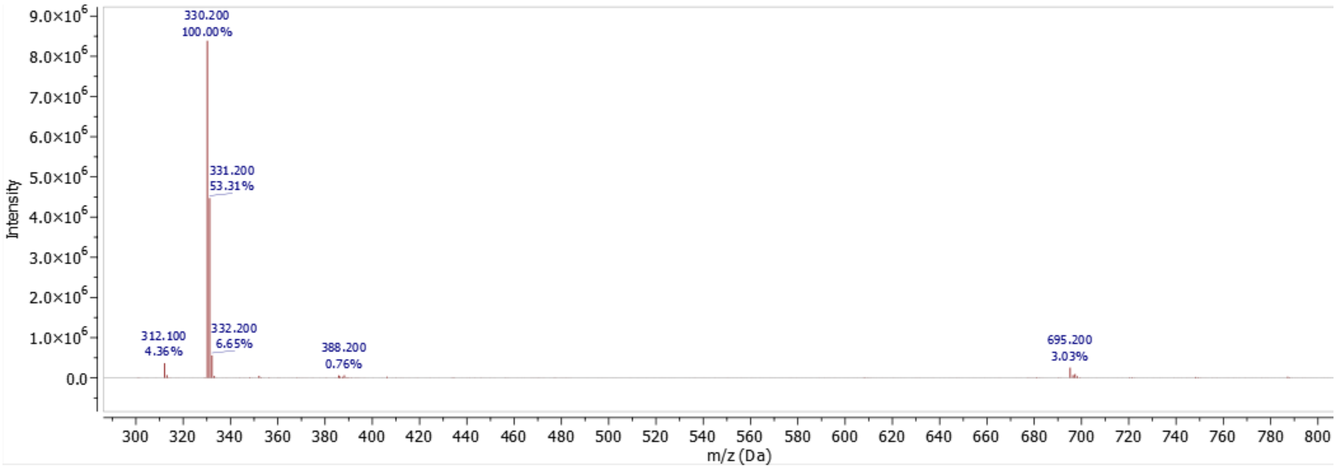

*Figure 91. Mass spectrum ESI^{+} of compound **20a***

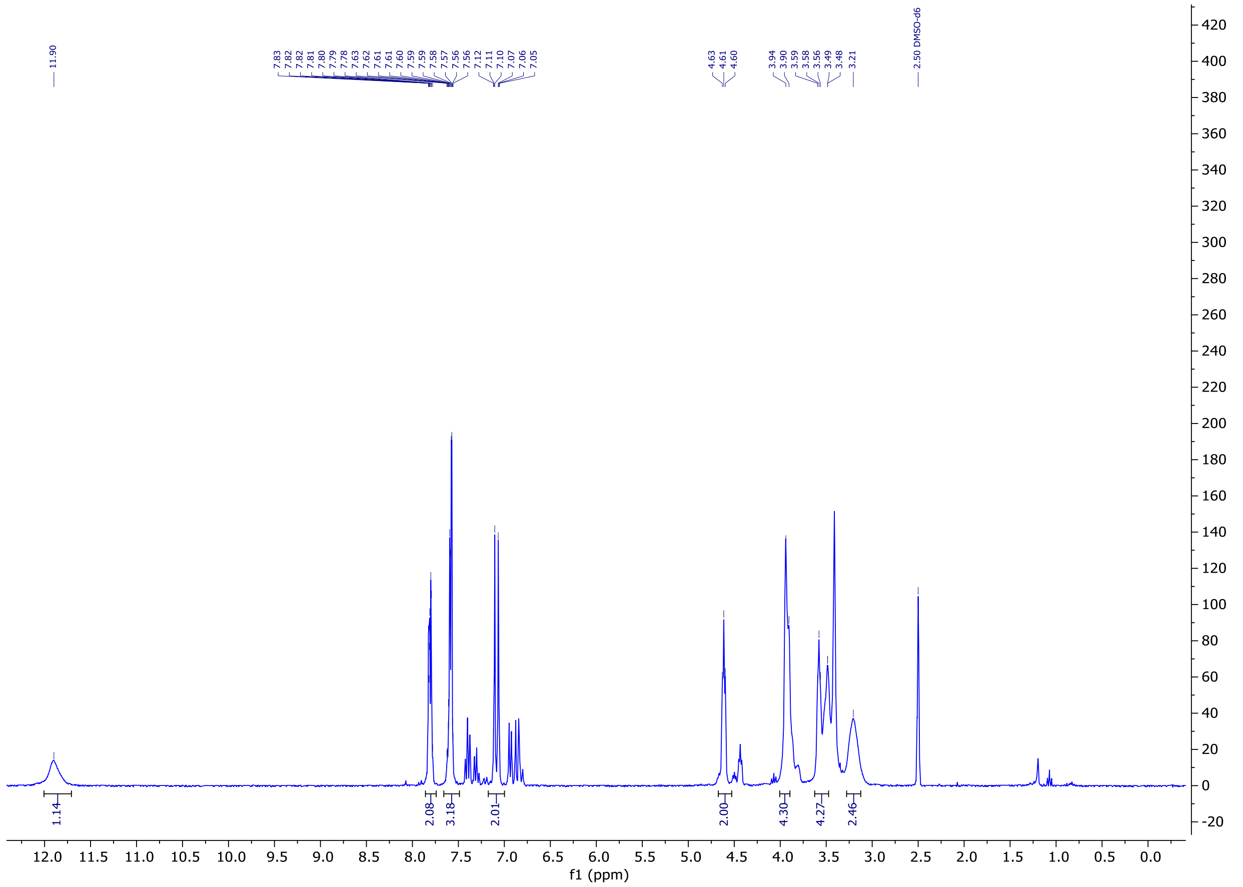

*Figure 92. ^{1}H NMR (300 MHz, DMSO-d6) spectrum of compound **20b***

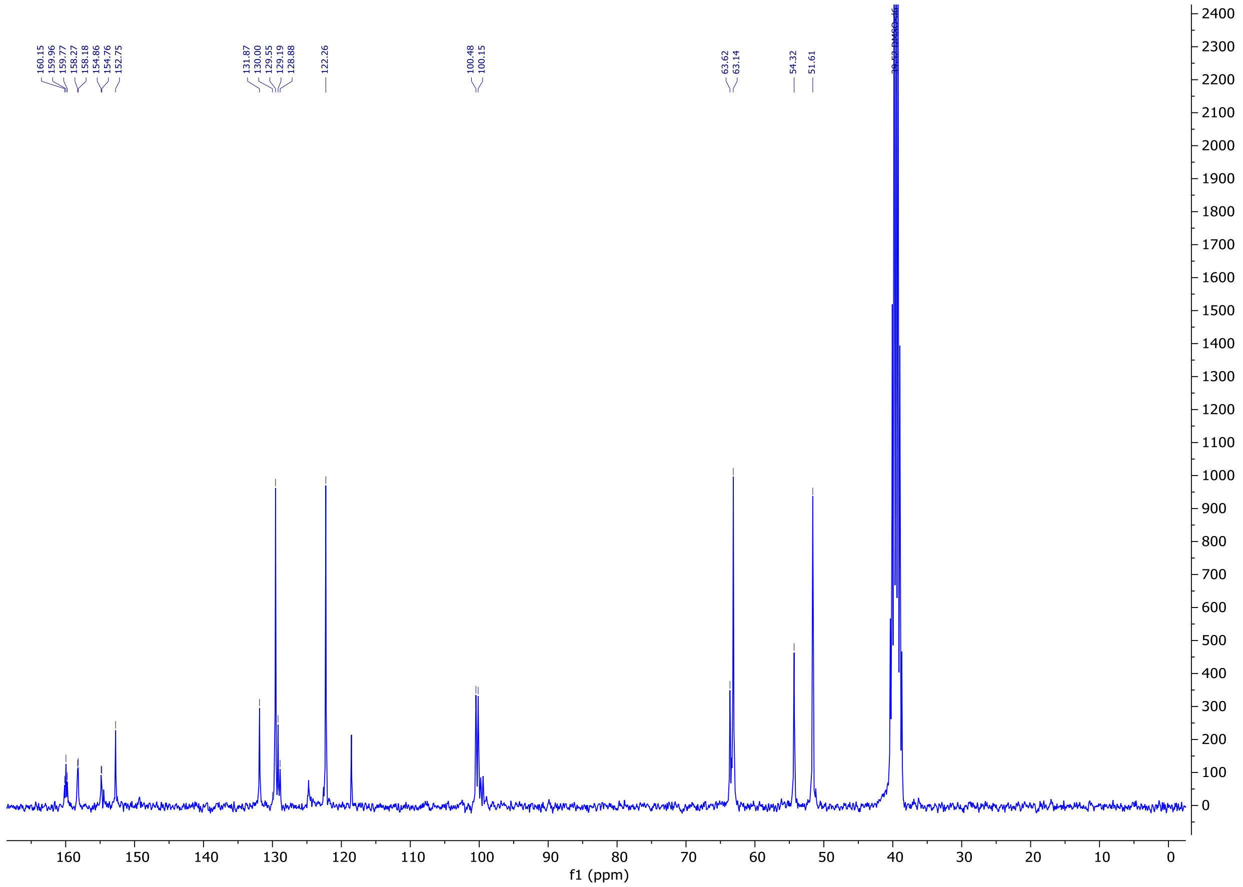

*Figure 93. ^{13}C NMR (75 MHz, DMSO-d6) spectrum of compound **20b***

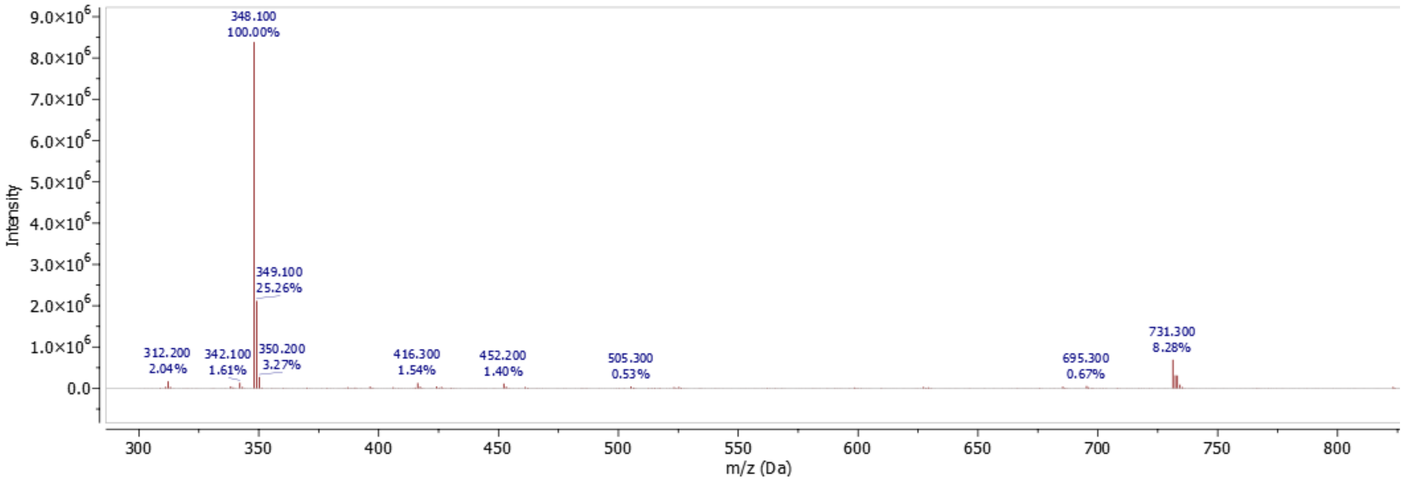

*Figure 94. Mass spectrum ESI⁺ of compound **20b***

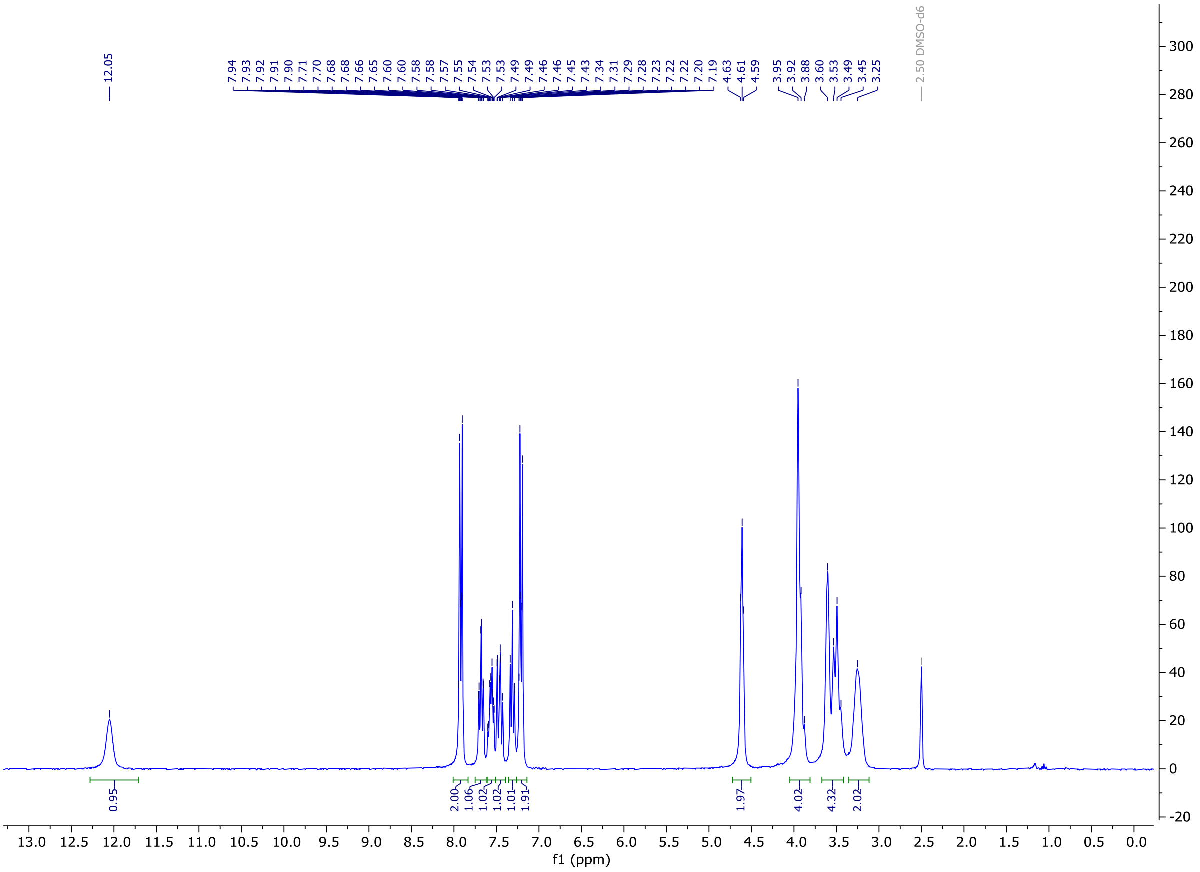

*Figure 95. ^{1}H NMR (300 MHz, DMSO-d6) spectrum of compound **20c***

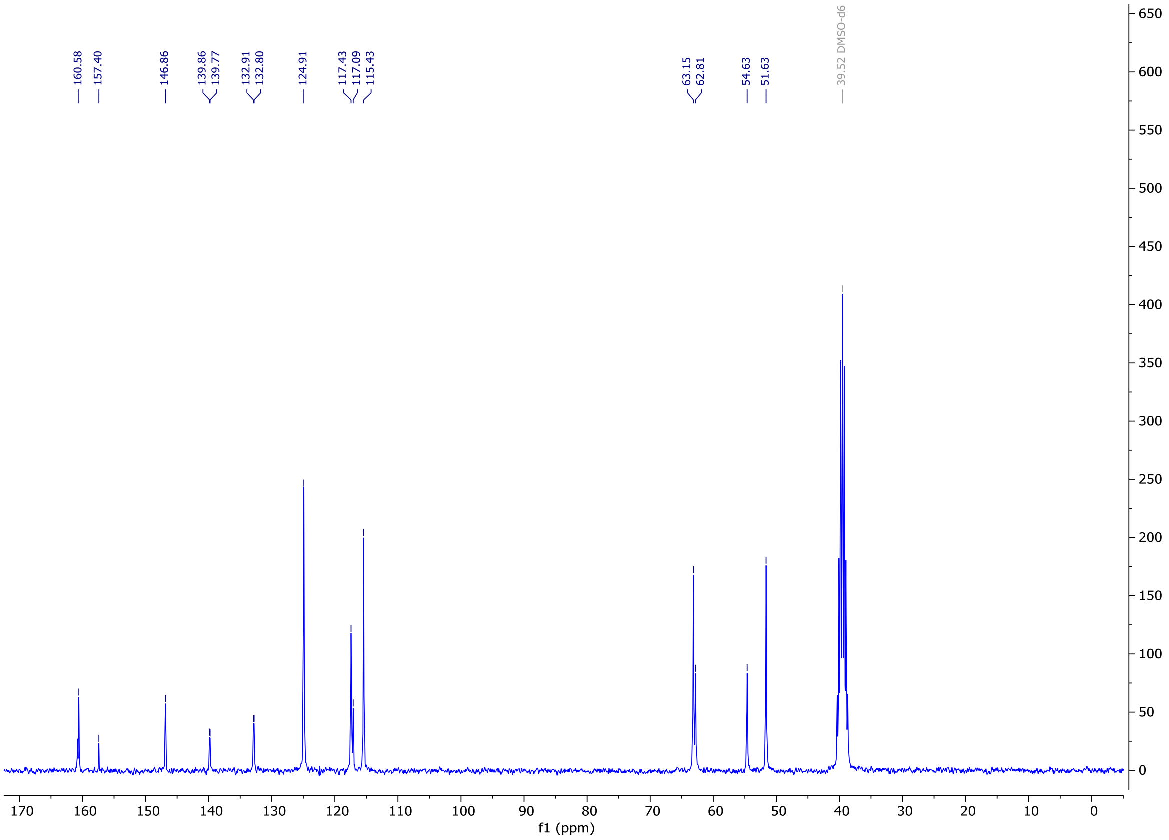

Figure 96. ^{13}C NMR (75 MHz, DMSO-d6) spectrum of compound ***20c***

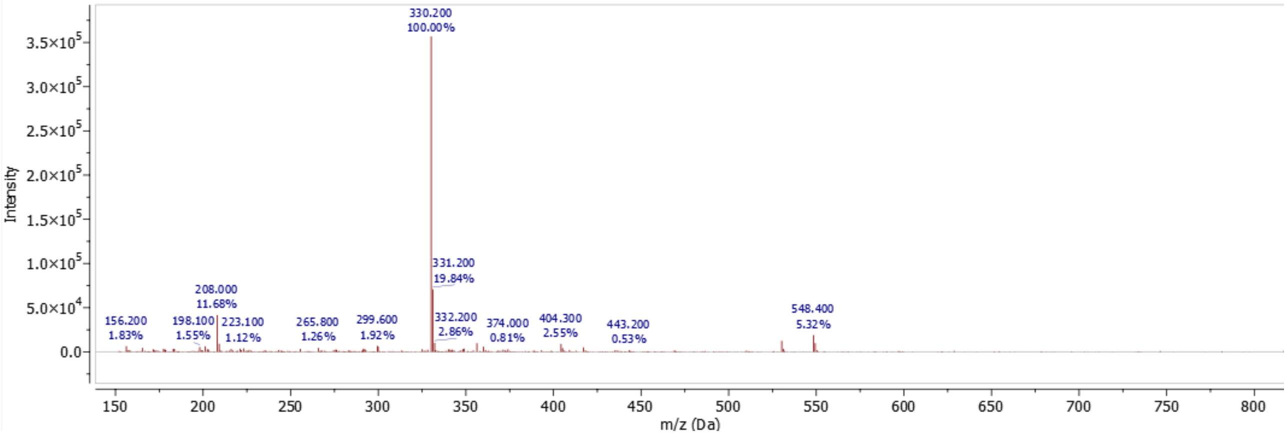

Figure 97. Mass spectrum ESI^{+} of compound ***20c***

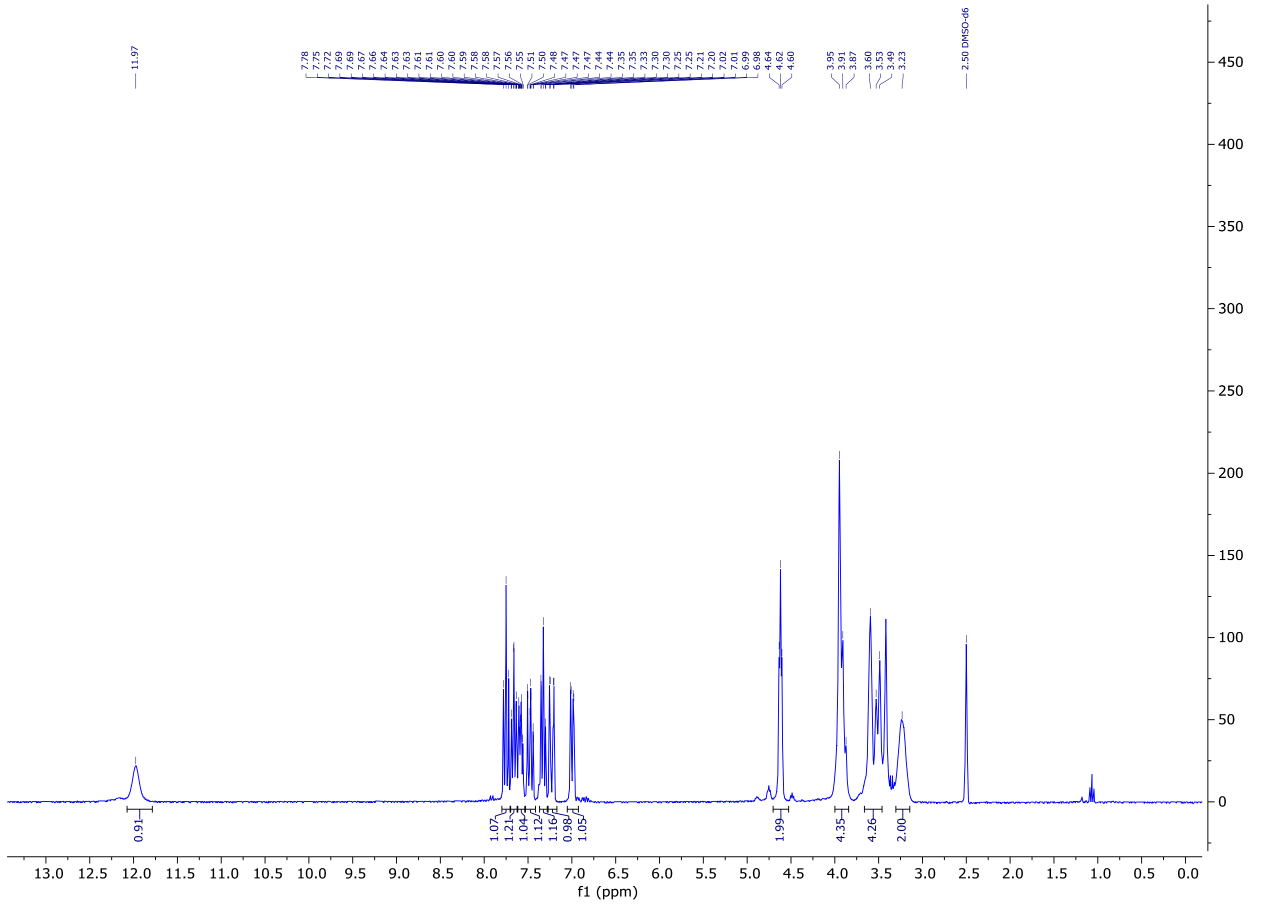

Figure 98. ^{1}H NMR (300 MHz, DMSO-d6) spectrum of compound **20d**

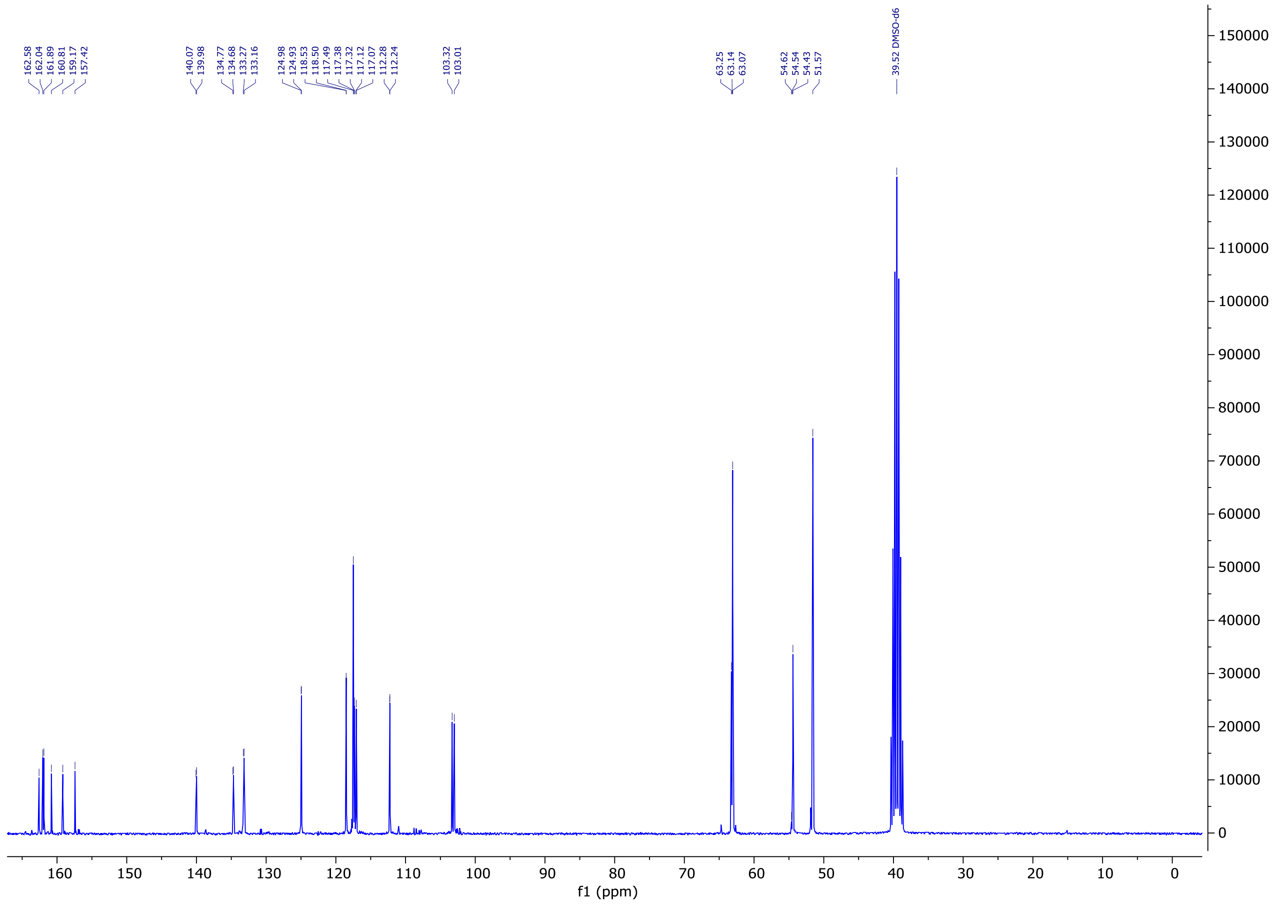

Figure 99. ^{13}C NMR (75 MHz, DMSO-d6) spectrum of compound **20d**

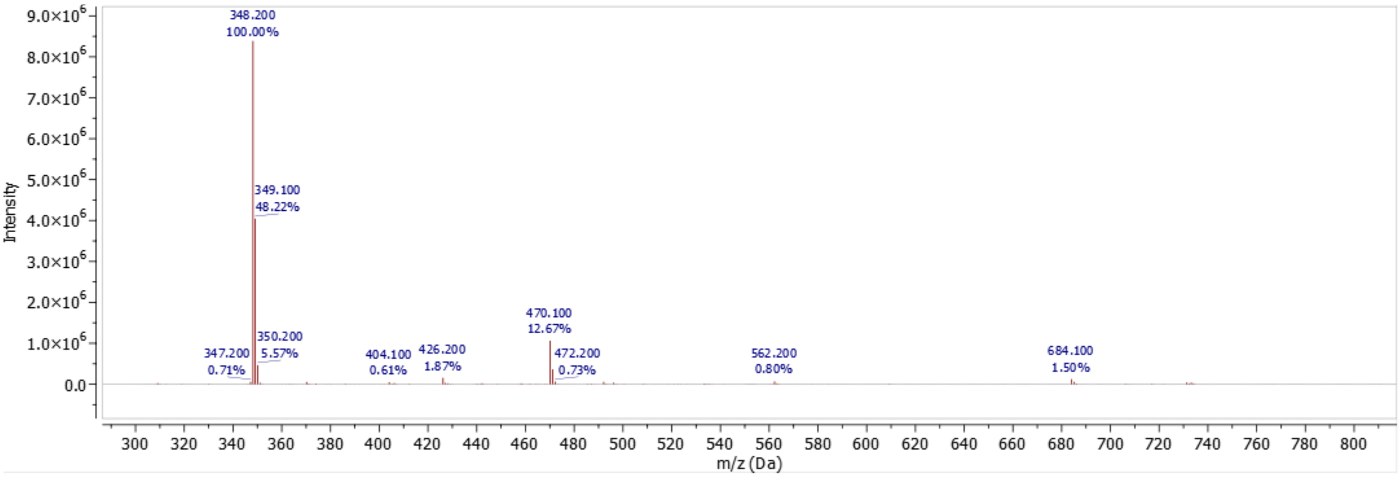

Figure 100. Mass spectrum ESI^{+} *of compound* ***20d***

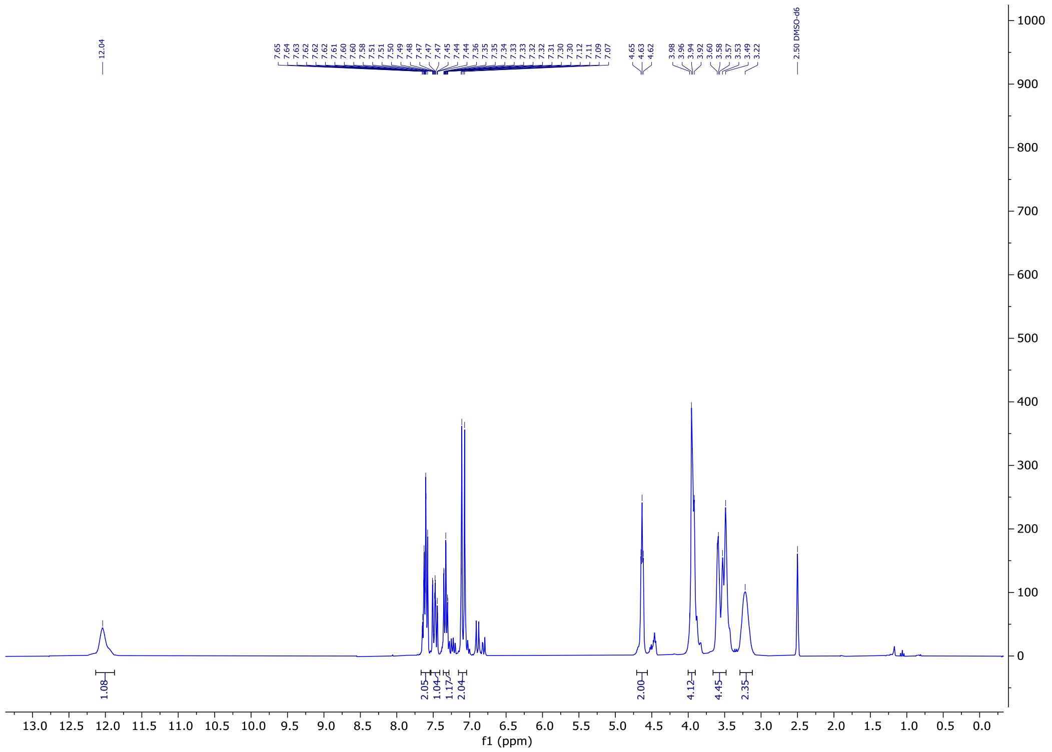

Figure 101. ^{1}*H NMR (300 MHz, DMSO-d6) spectrum of compound* ***20e***

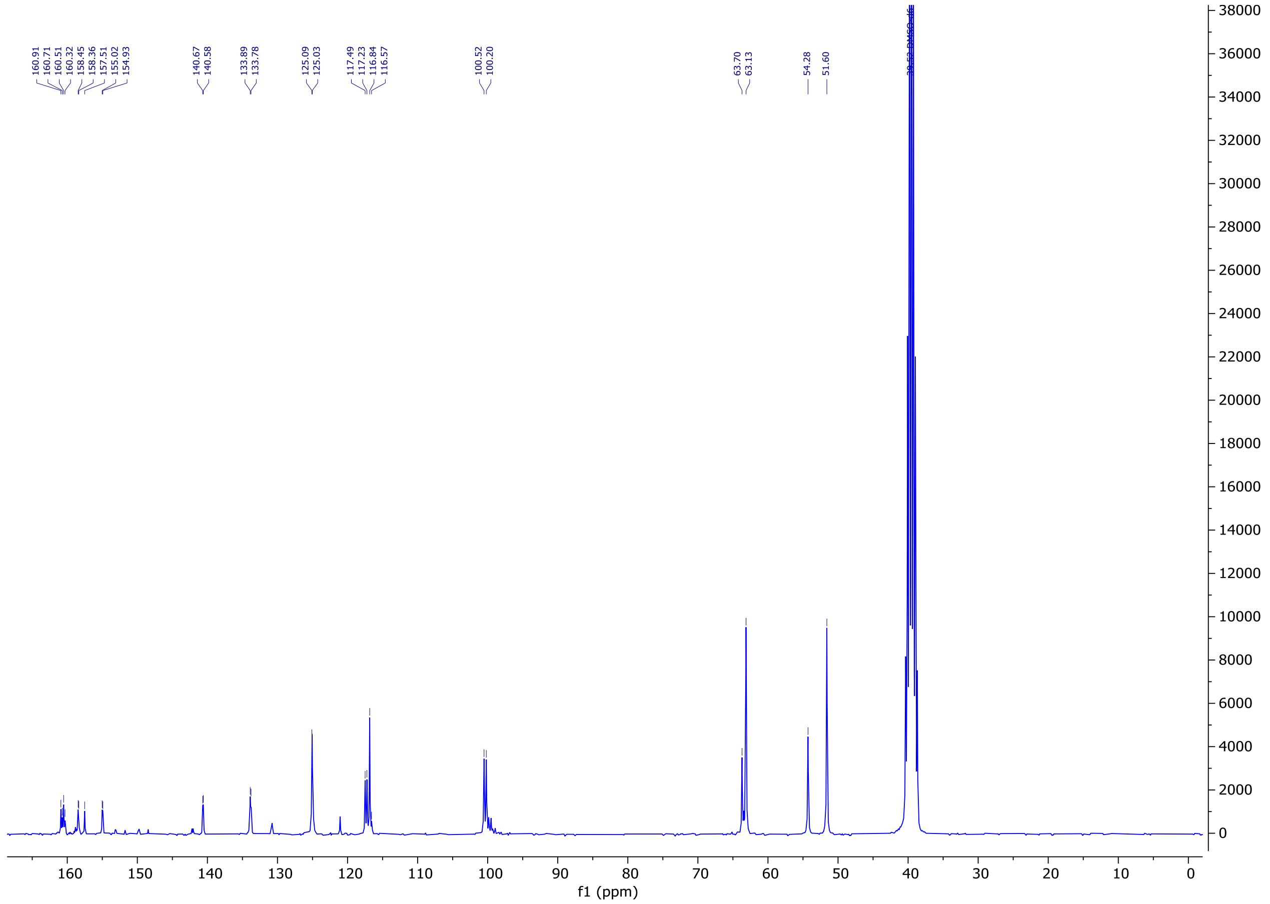

*Figure 102. ^{13}C NMR (75 MHz, DMSO-d6) spectrum of compound **20e***

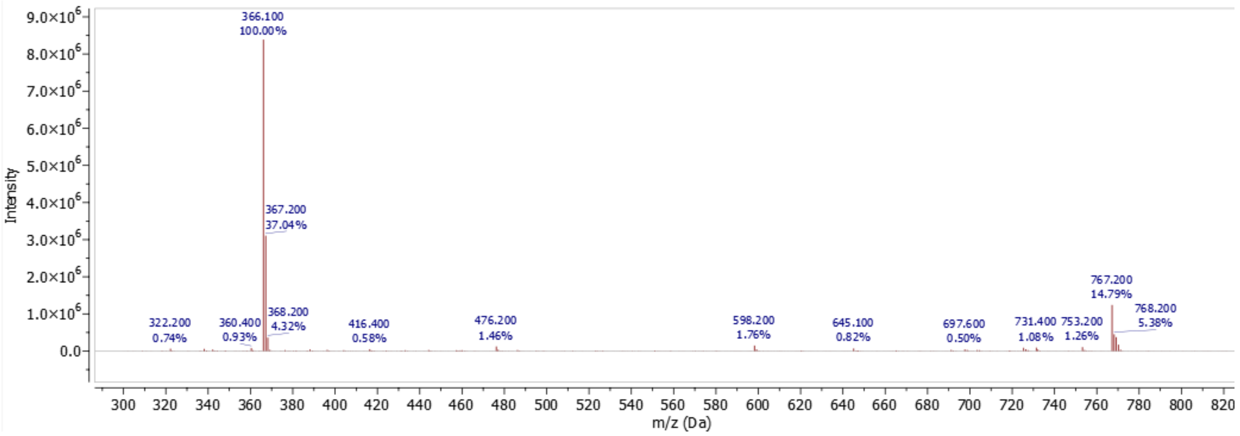

*Figure 103. Mass spectrum ESI$^+$ of compound **20e***

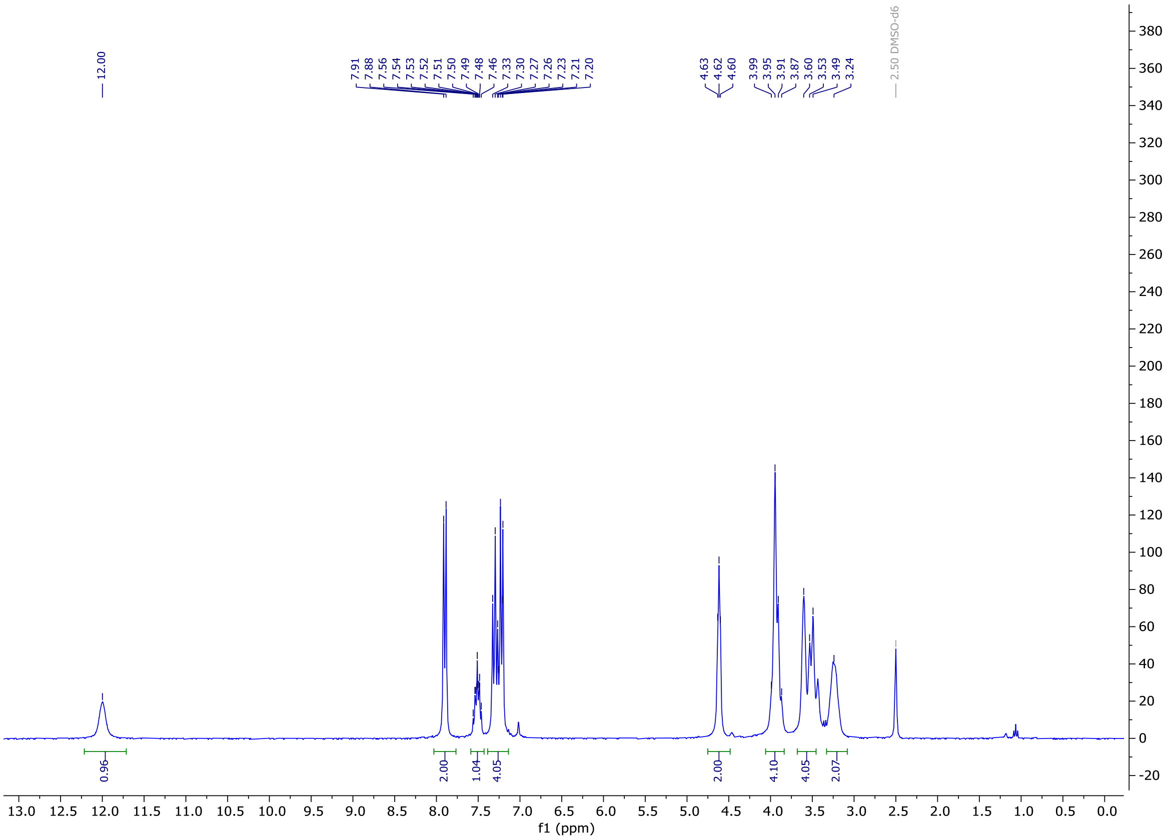

*Figure 104. ^{1}H NMR (300 MHz, DMSO-d6) spectrum of compound **20f***

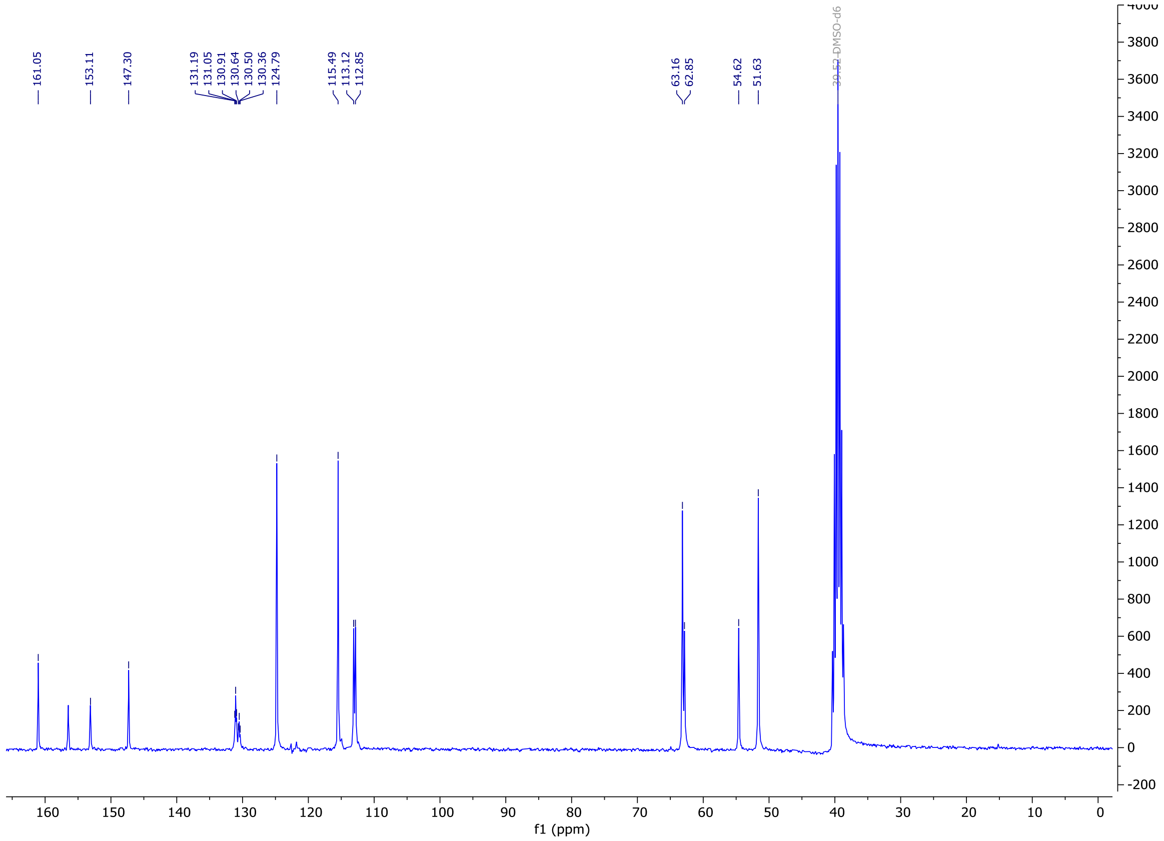

*Figure 105. ^{13}C NMR (75 MHz, DMSO-d6) spectrum of compound **20f***

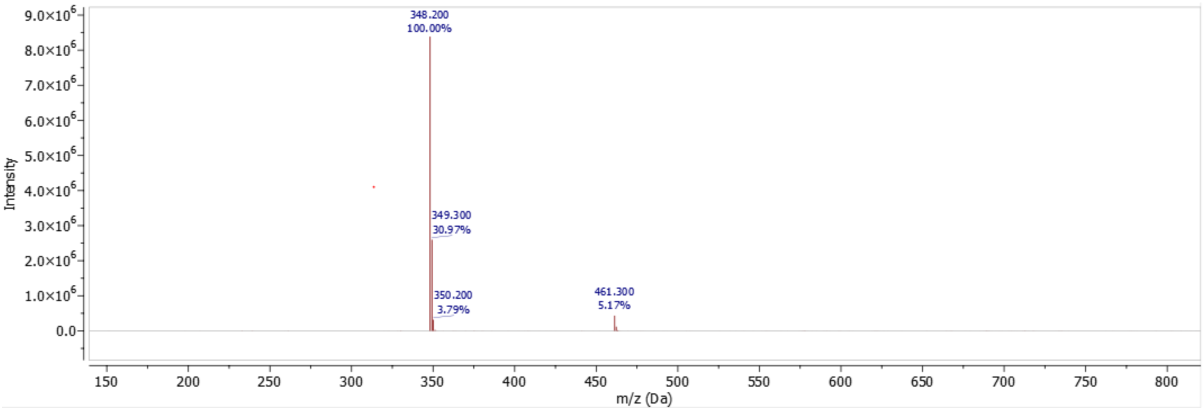

*Figure 106. Mass spectrum ESI$^+$ of compound **20f***

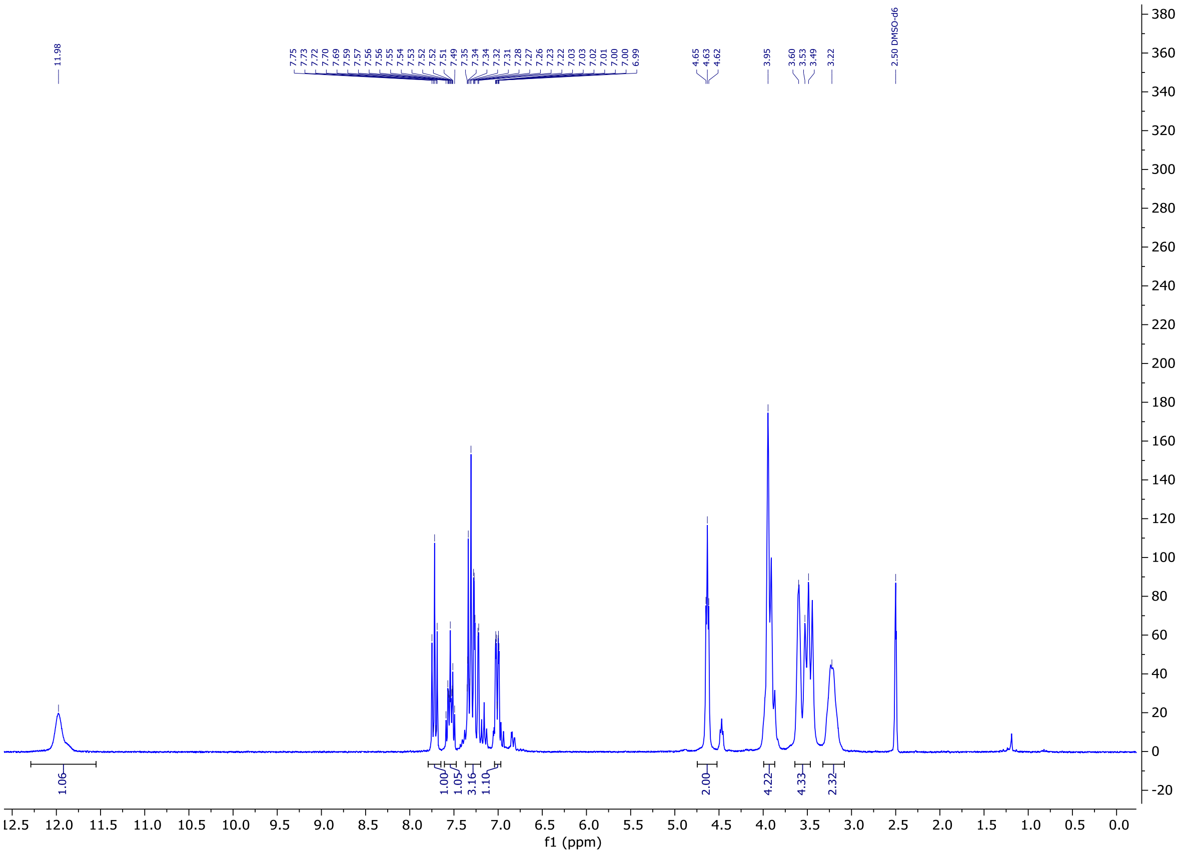

*Figure 107. ^{1}H NMR (300 MHz, DMSO-d6) spectrum of compound **20g***

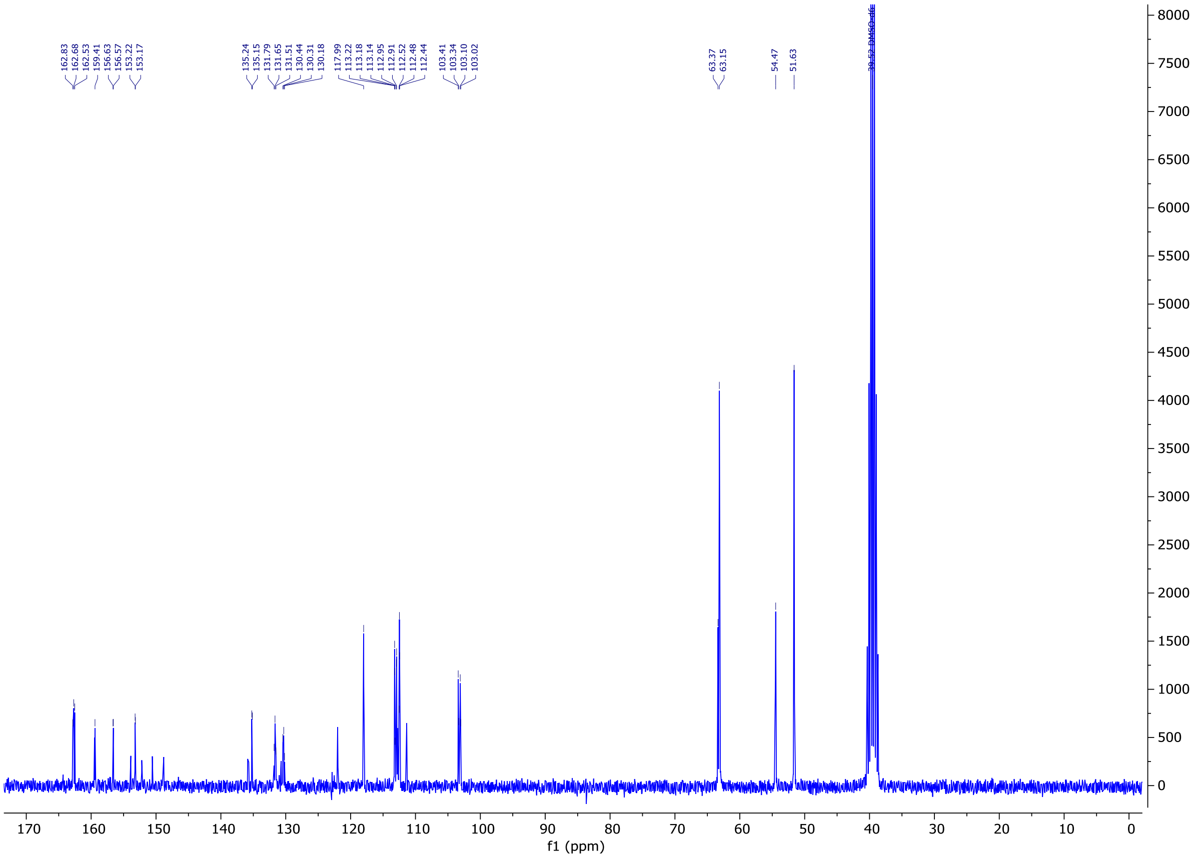

*Figure 108. ^{13}C NMR (75 MHz, DMSO-d6) spectrum of compound **20g***

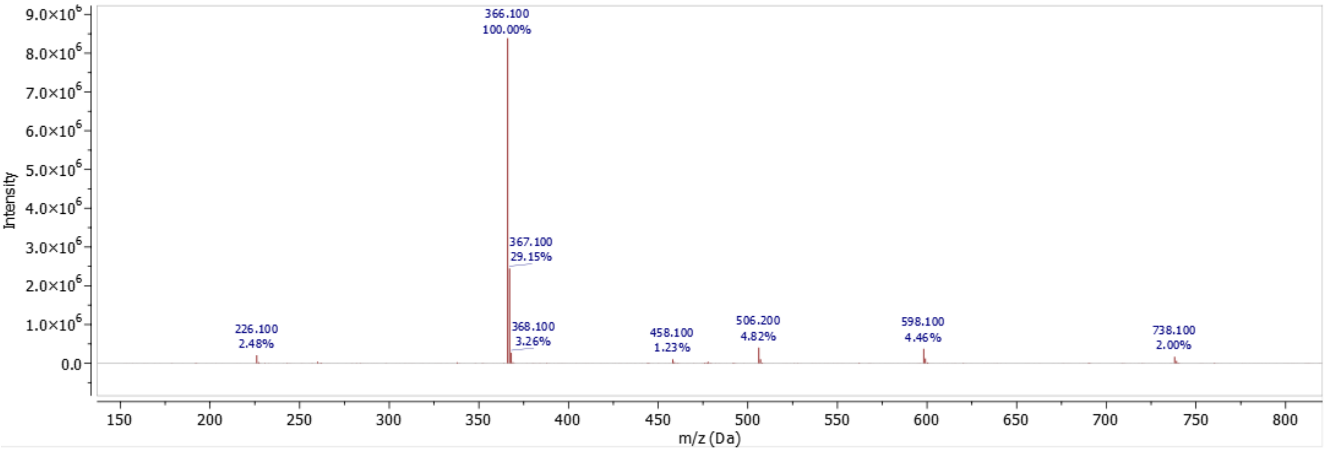

*Figure 109. Mass spectrum ESI^{+} of compound **20g***

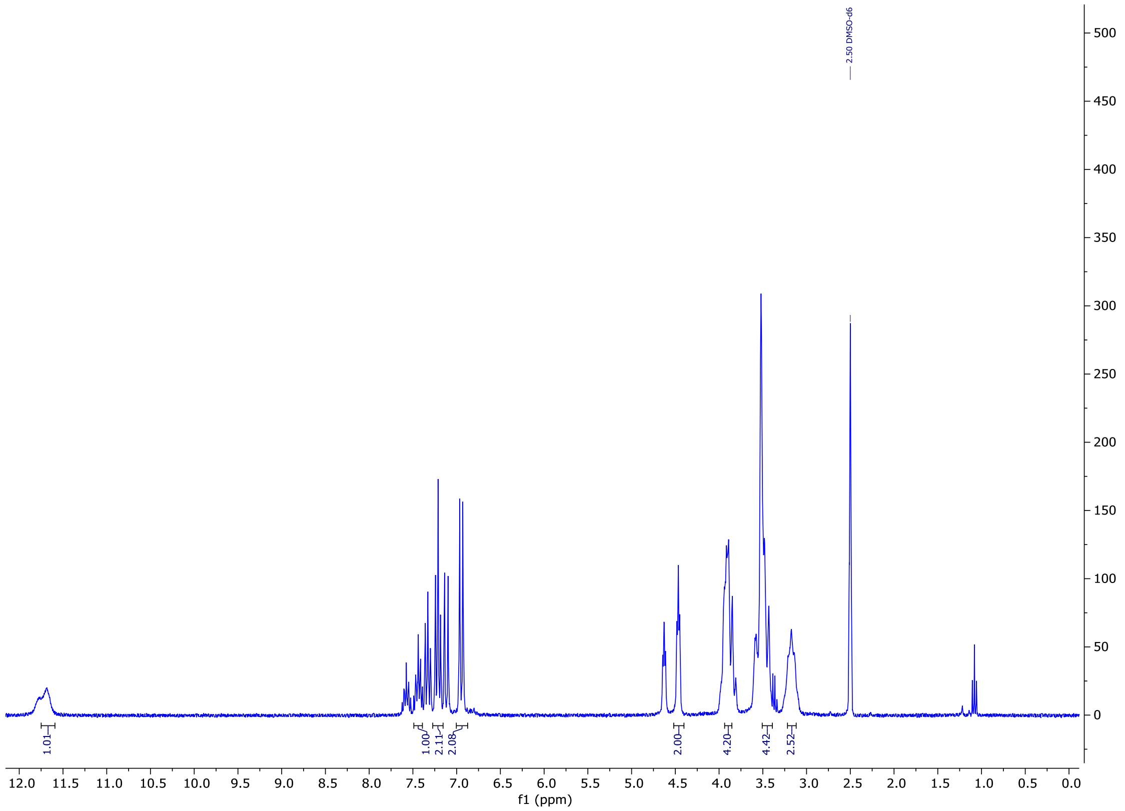

*Figure 110. ^{1}H NMR (300 MHz, DMSO-d6) spectrum of compound **20h***

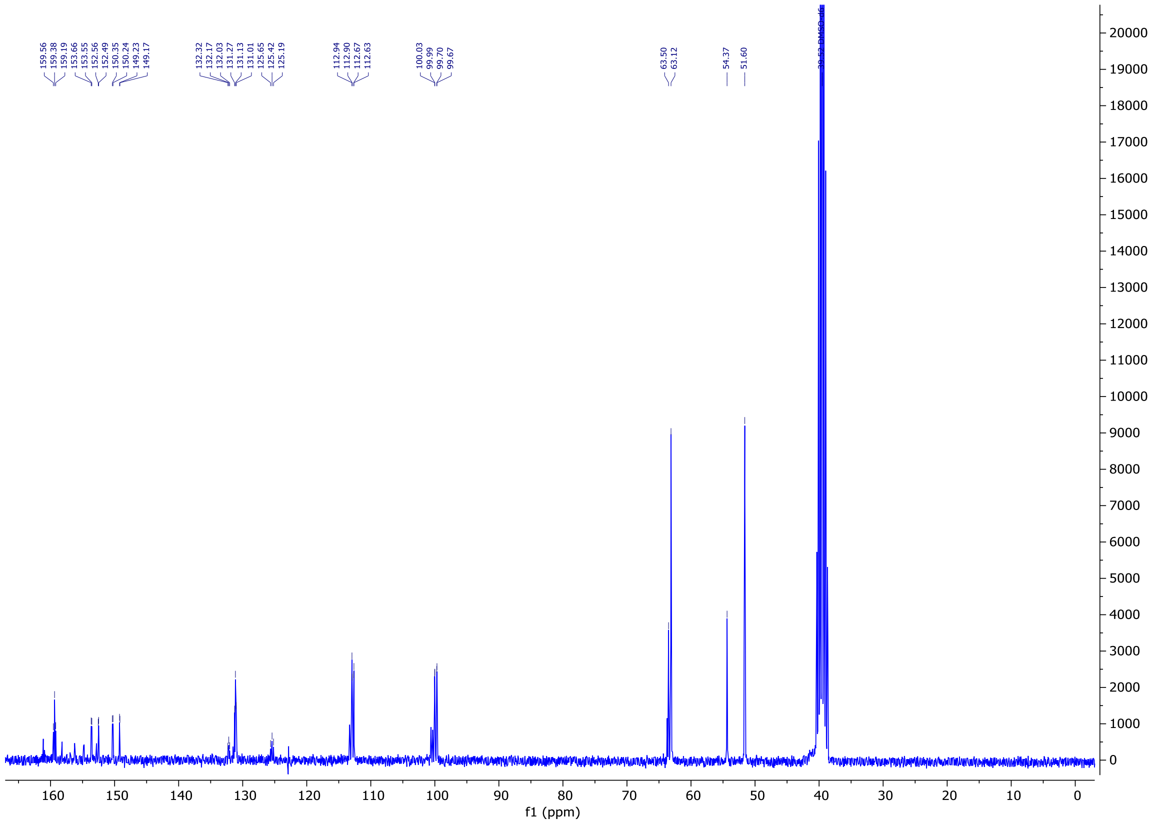

*Figure 111. ^{13}C NMR (75 MHz, DMSO-d6) spectrum of compound **20h***

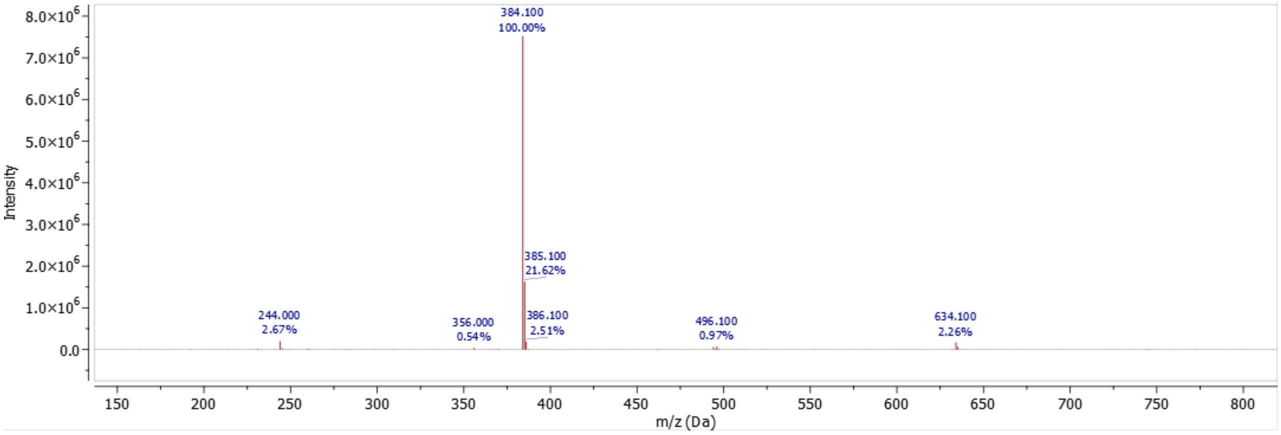

*Figure 112. Mass spectrum ESI⁺ of compound **20h***

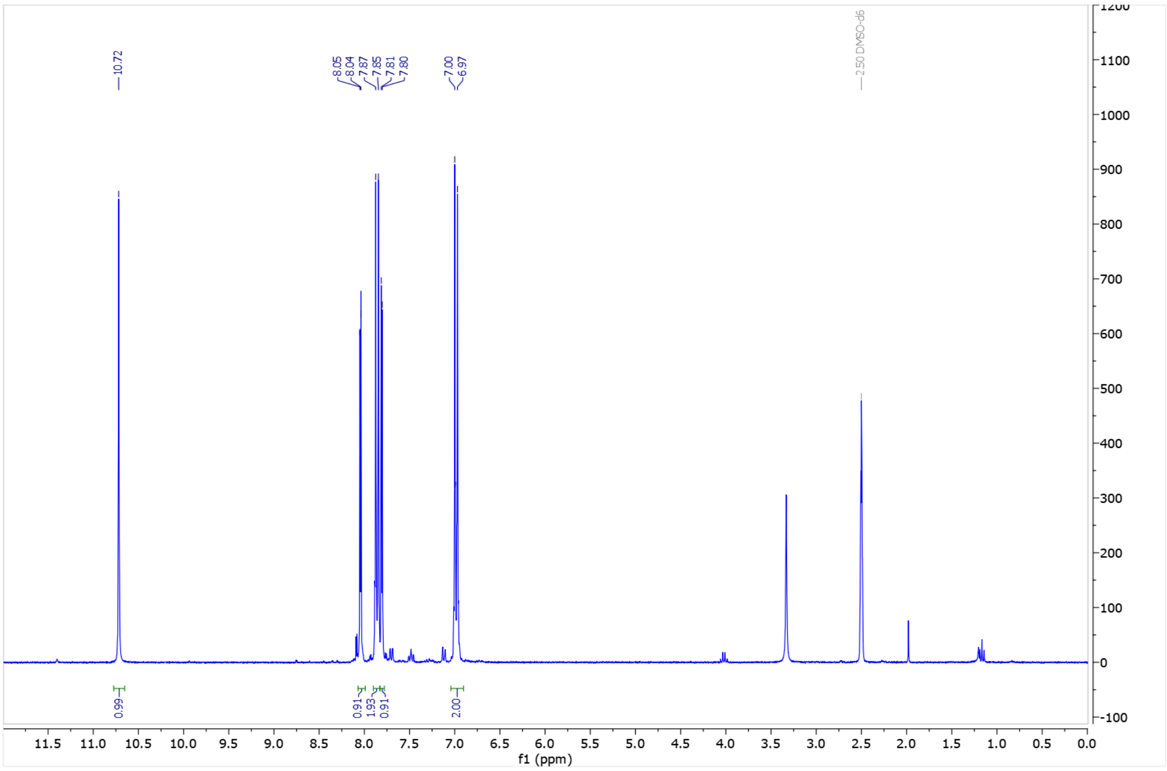

*Figure 113. ^{1}H NMR (300 MHz, DMSO-d6) spectrum of compound **22a***

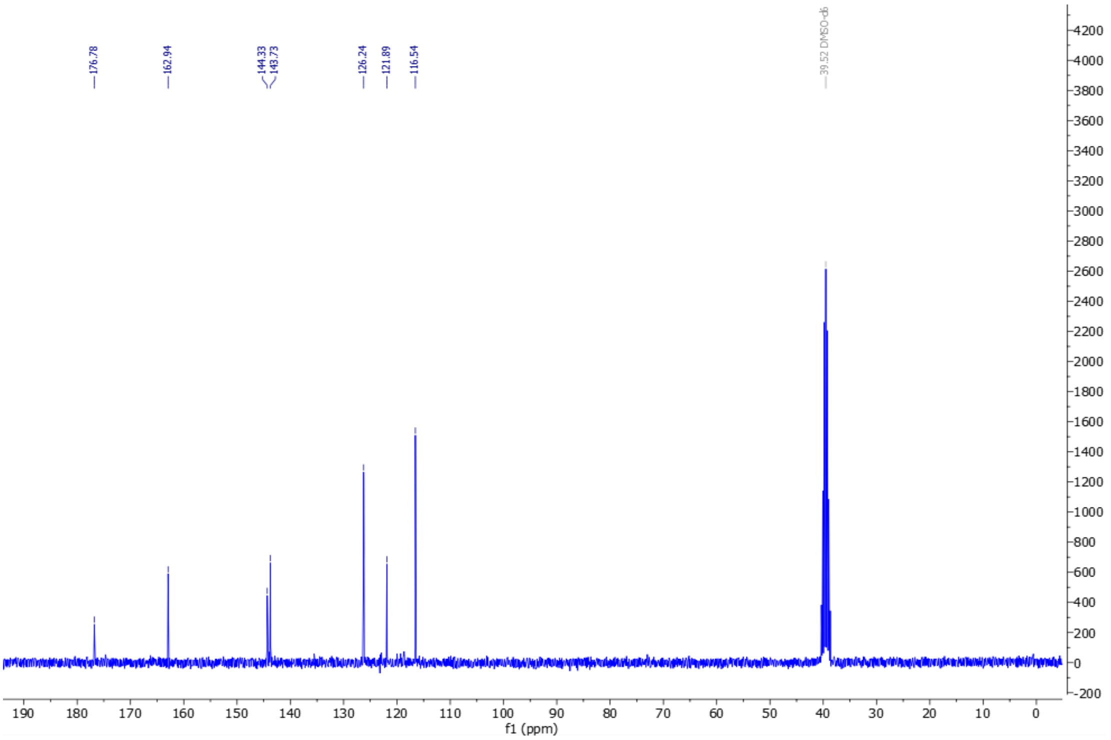

Figure 114. ^{13}C NMR (75 MHz, DMSO-d6) spectrum of compound ***22a***

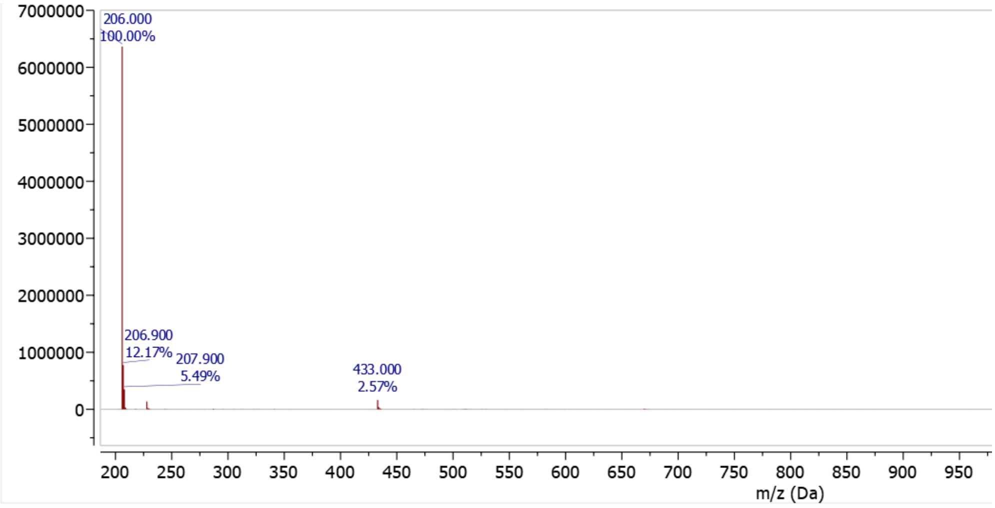

Figure 115. Mass spectrum ESI$^+$ of compound ***22a***

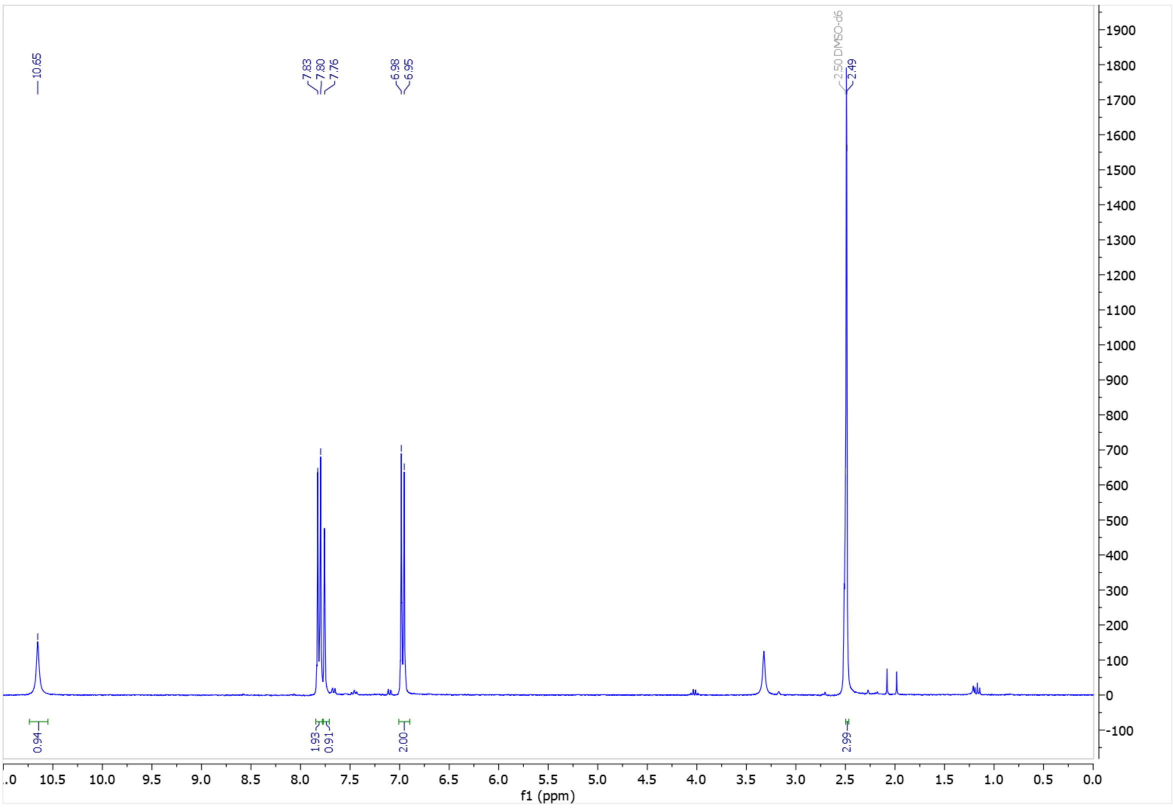

*Figure 116. ^{1}H NMR (300 MHz, DMSO-d6) spectrum of compound **22b***

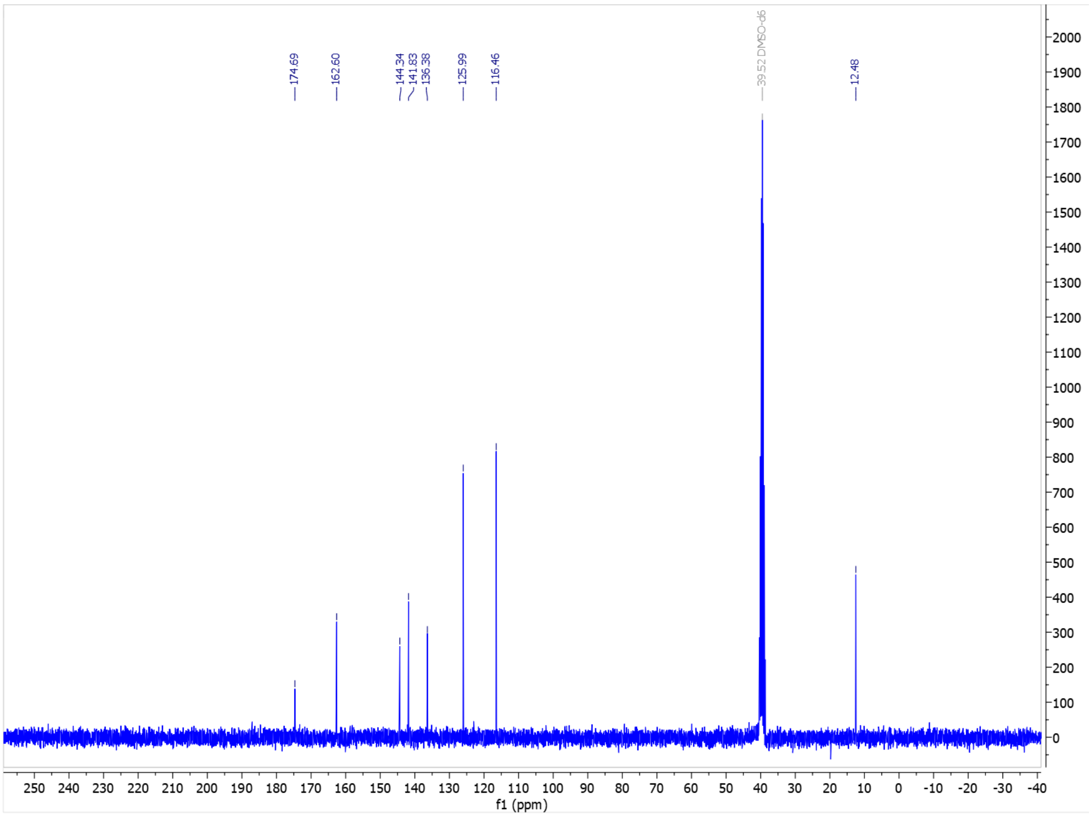

*Figure 117. ^{13}C NMR (75 MHz, DMSO-d6) spectrum of compound **22b***

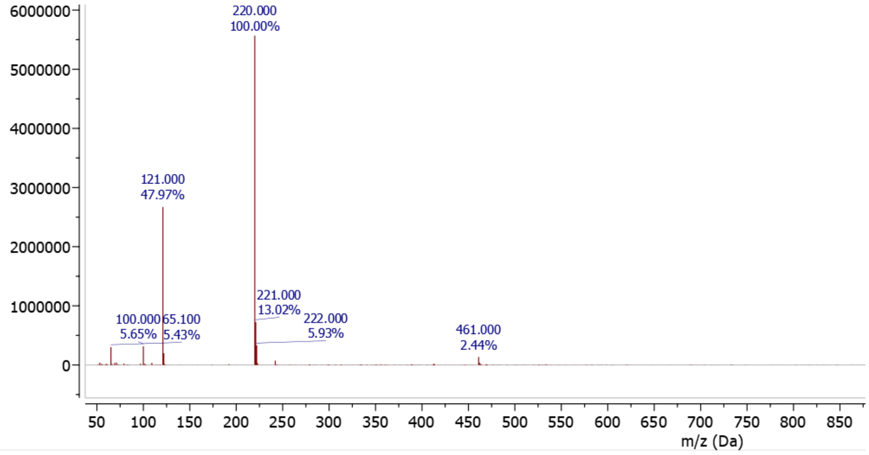

*Figure 118. Mass spectrum ESI⁺ of compound **22b***

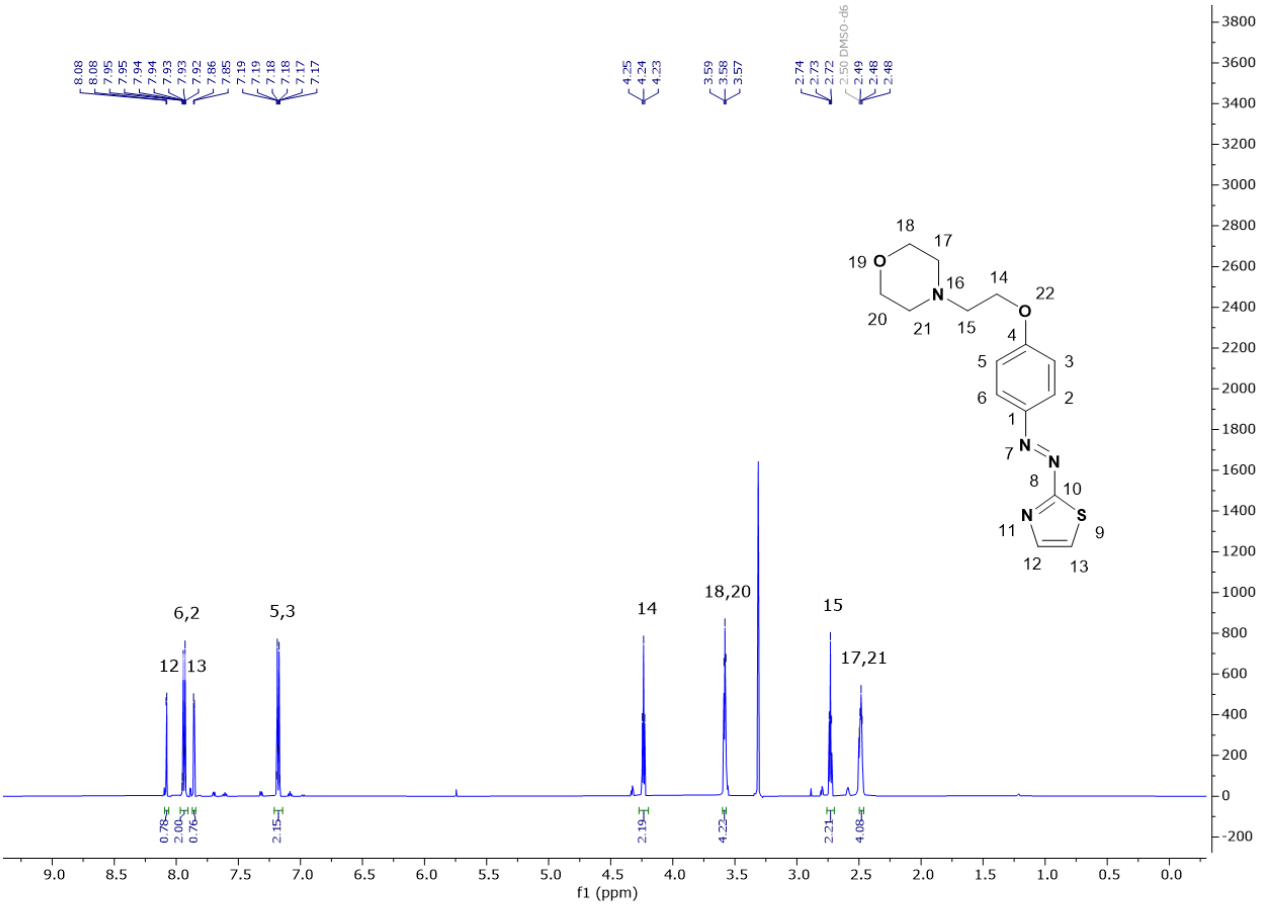

*Figure 119. ¹H NMR (600 MHz, DMSO-d6) spectrum of compound **23a***

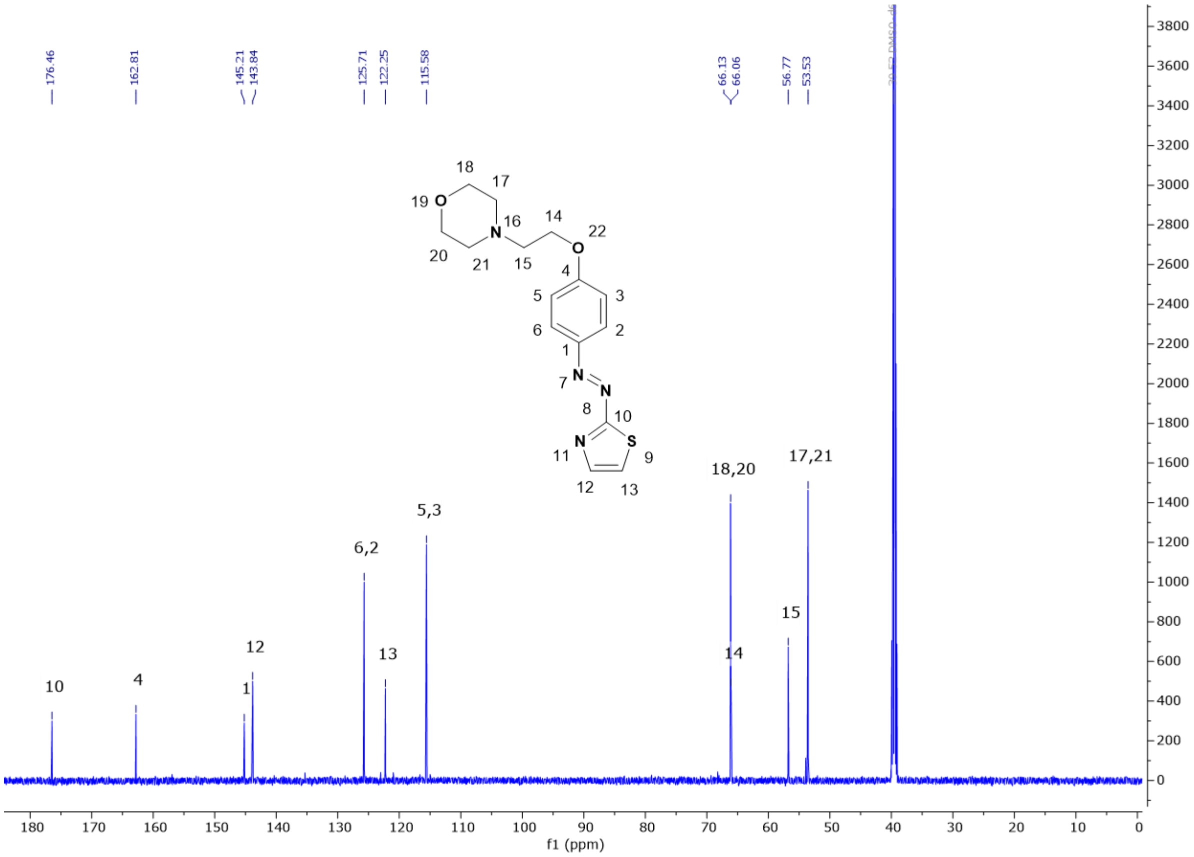

Figure 120. ^{13}C NMR (150 MHz, DMSO-d6) spectrum of compound ***23a***

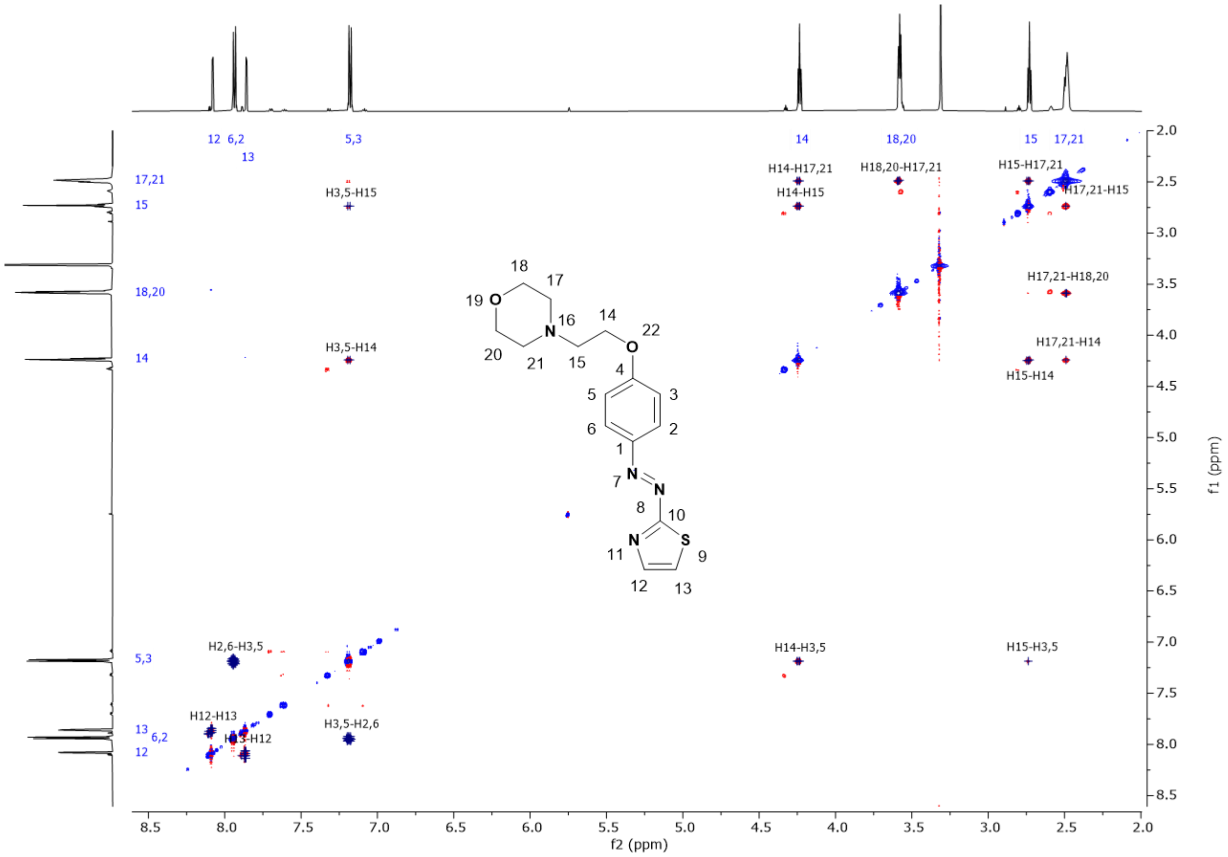

Figure 121. ^{1}H,^{1}H-NOESY NMR (600 MHz, DMSO-d6) spectrum of compound ***23a***

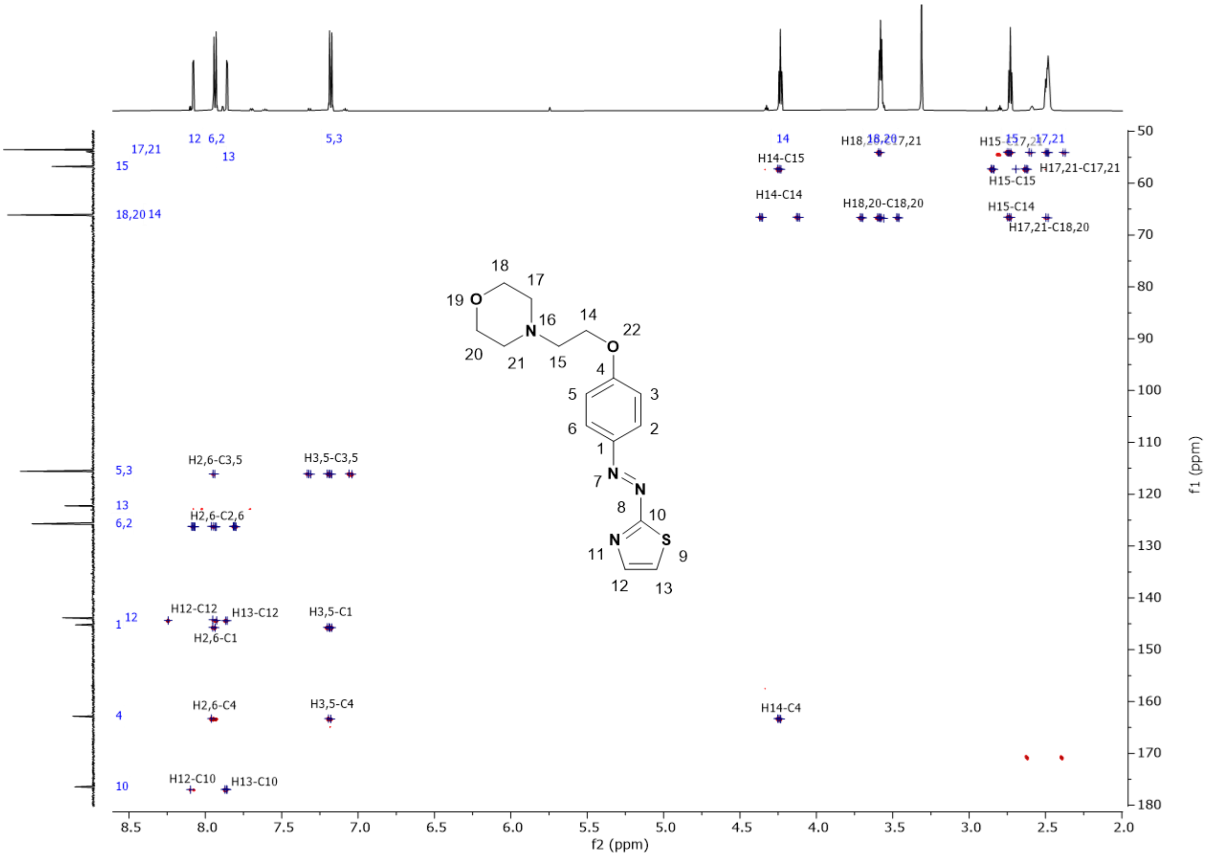

Figure 122. ^{1}H,^{13}C-HMBC NMR (DMSO-d6) spectrum of compound **23a**

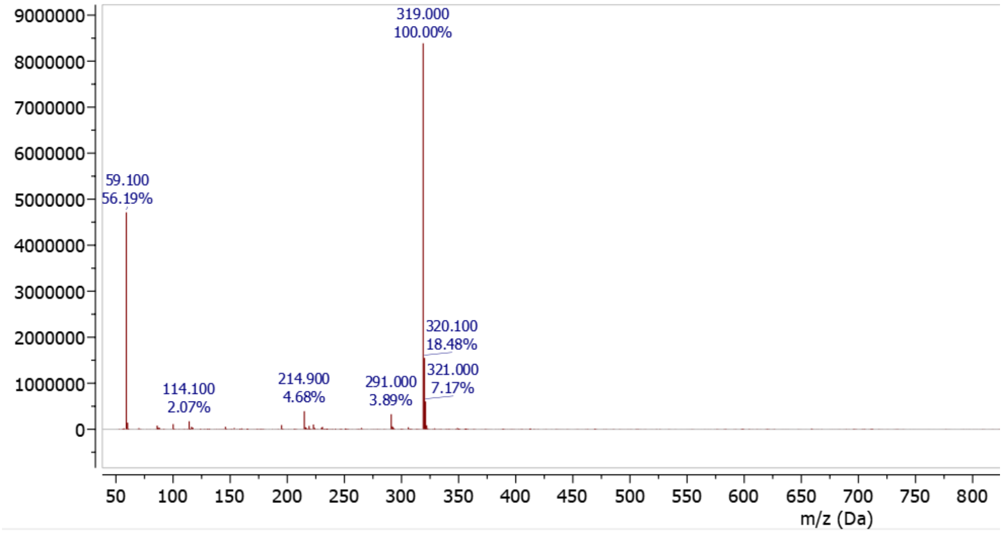

Figure 123. Mass spectrum ESI^{+} of compound **23a**

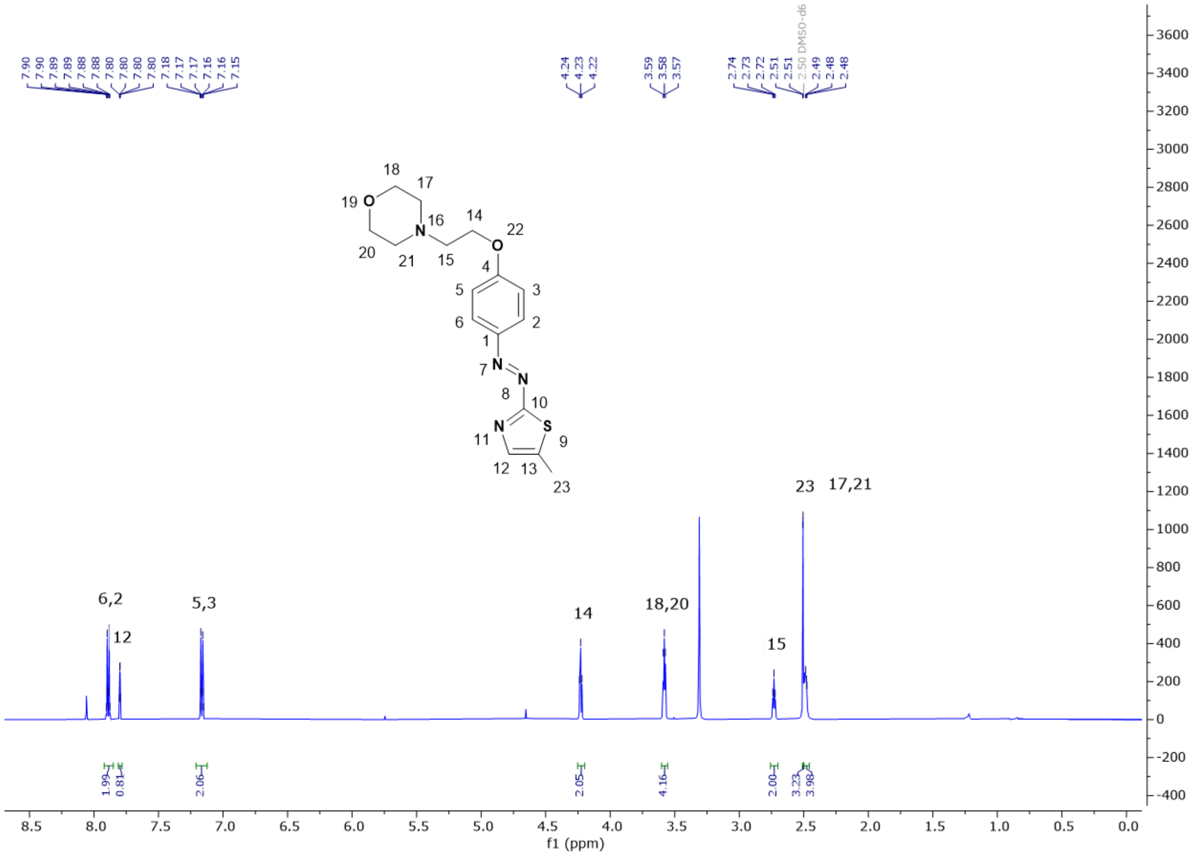

*Figure 124. ^{1}H NMR (600 MHz, DMSO-d6) spectrum of compound **23b***

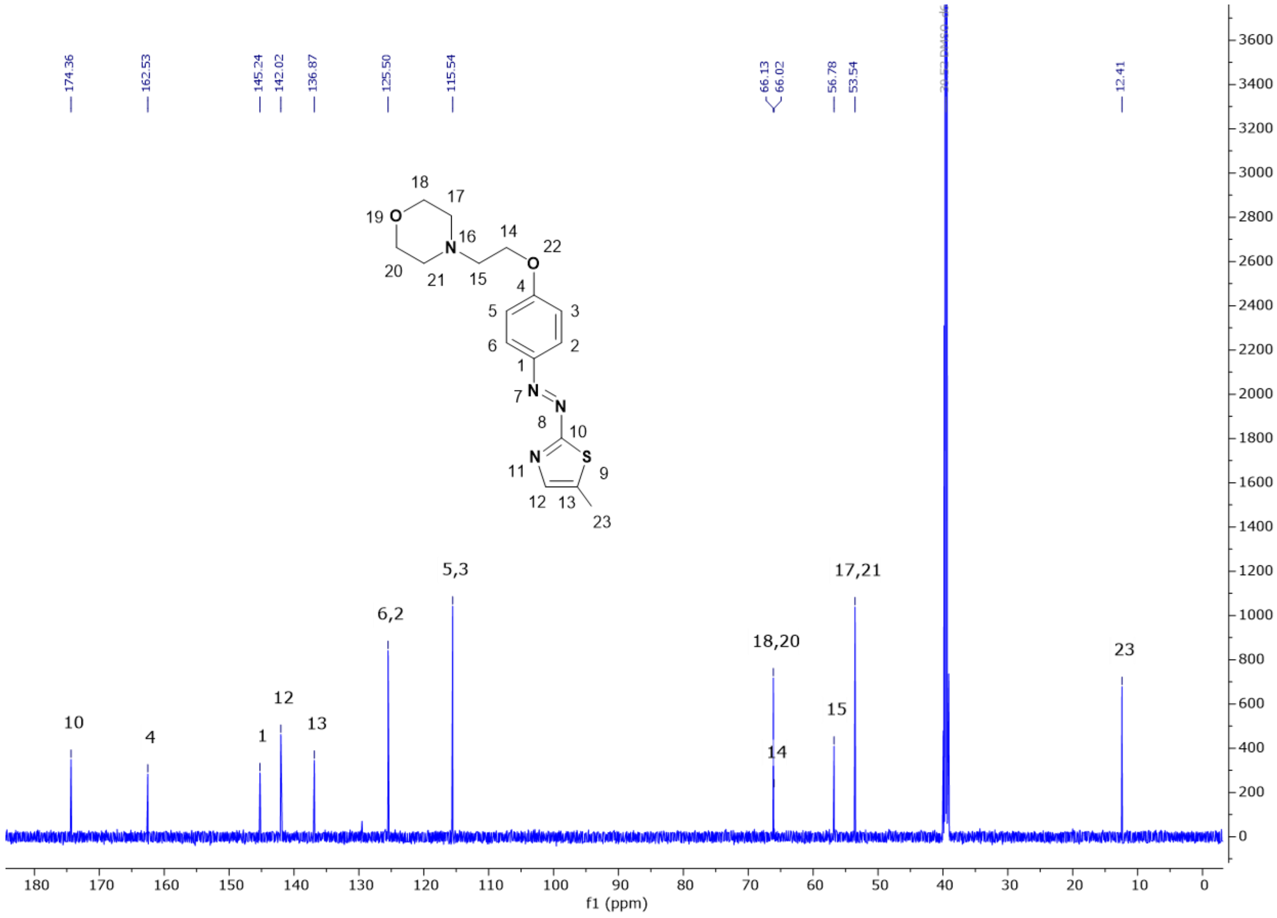

*Figure 125. ^{13}C NMR (150 MHz, DMSO-d6) spectrum of compound **23b***

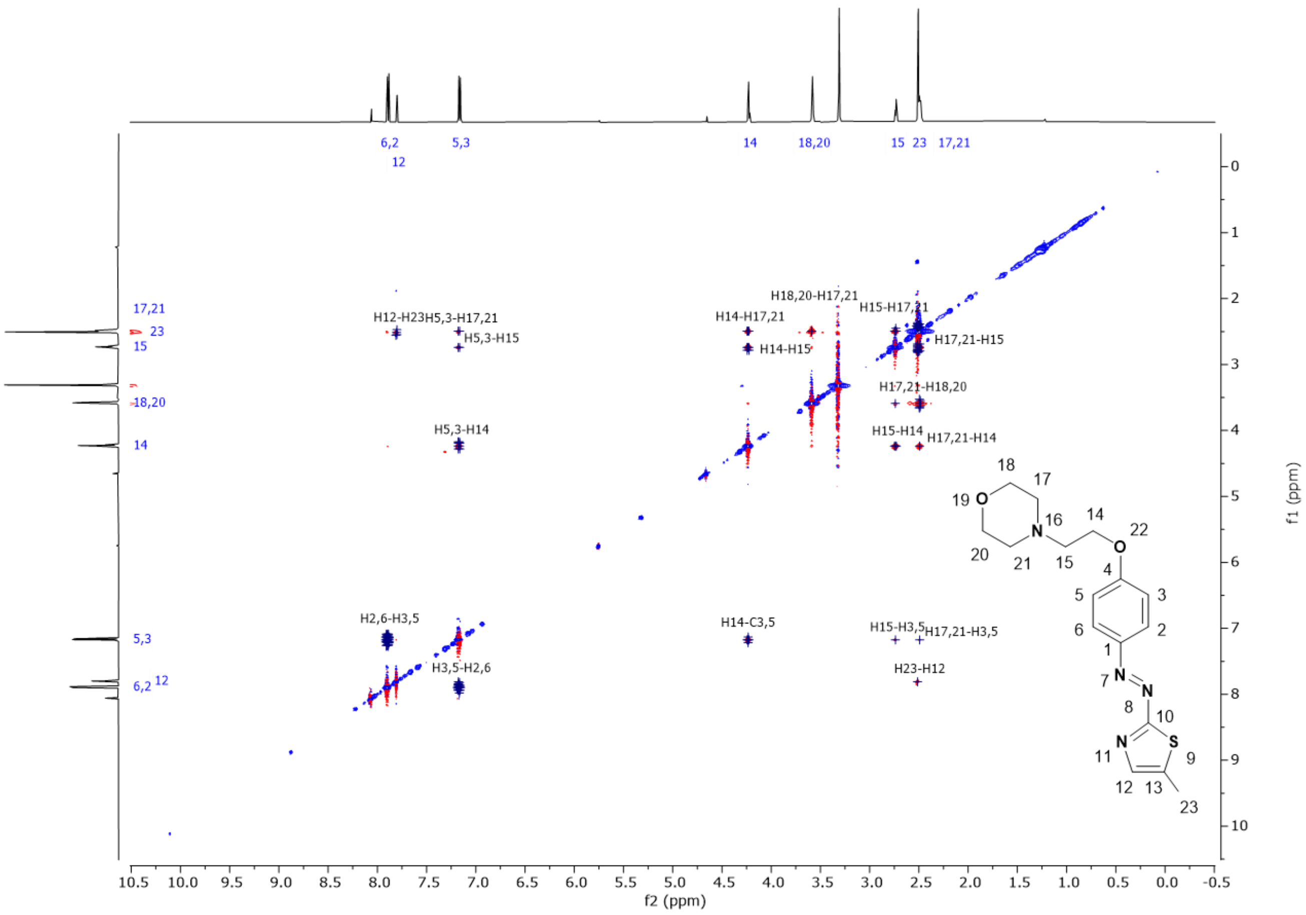

Figure 126. ¹H,¹H-NOESY NMR (600 MHz, DMSO-d6) spectrum of compound ***23b***

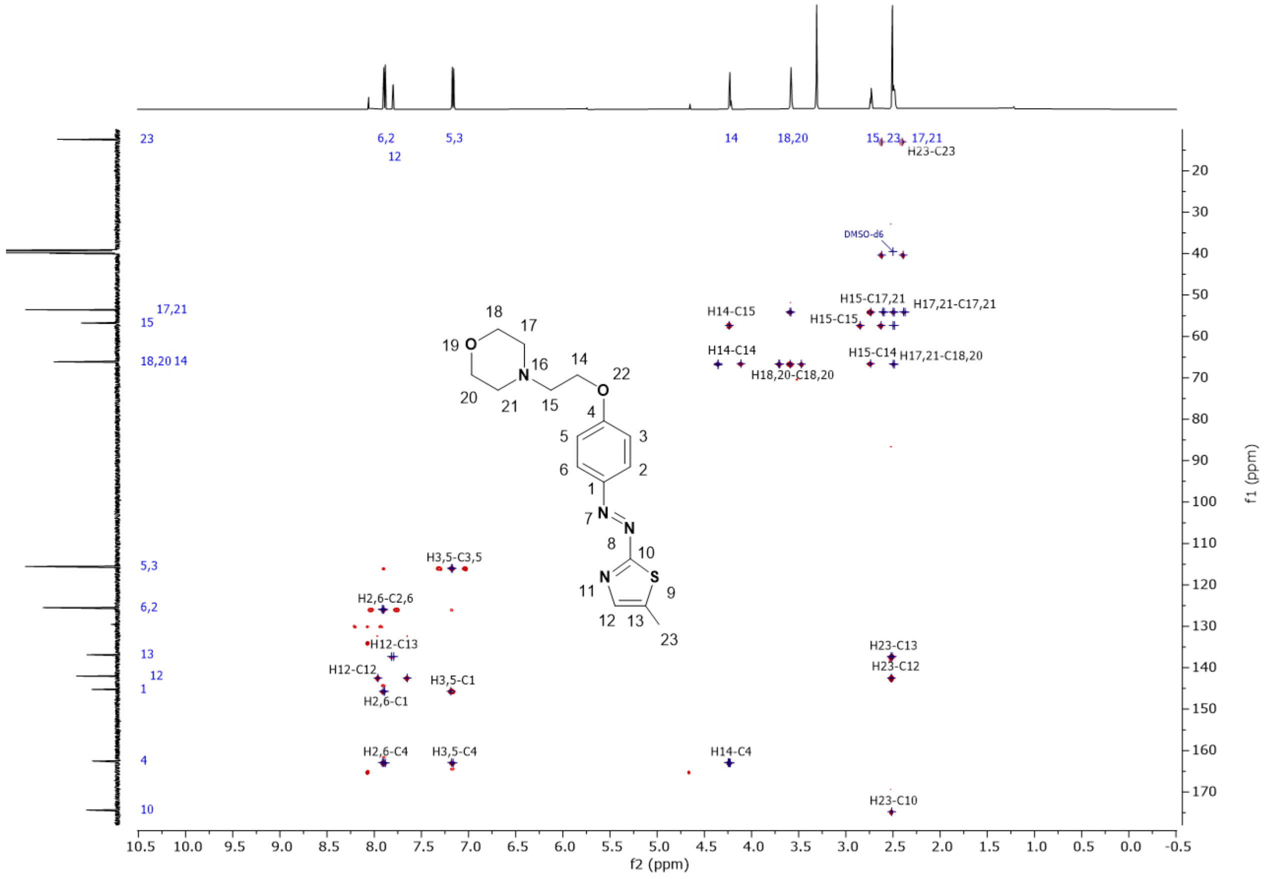

Figure 127. ¹H,¹³C-HMBC NMR (DMSO-d6) spectrum of compound ***23b***

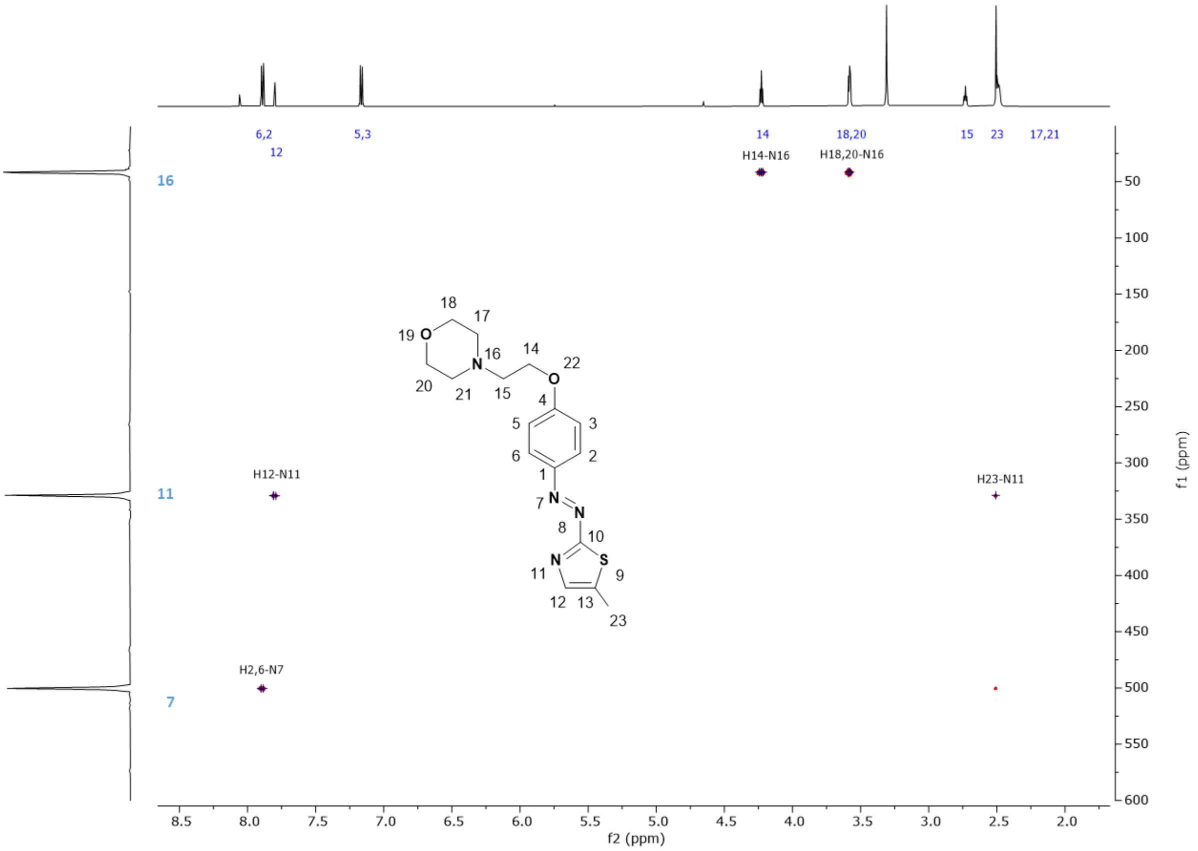

Figure 128. $^{1}H,^{15}N$-HMBC NMR (DMSO-d6) spectrum of compound ***23b***

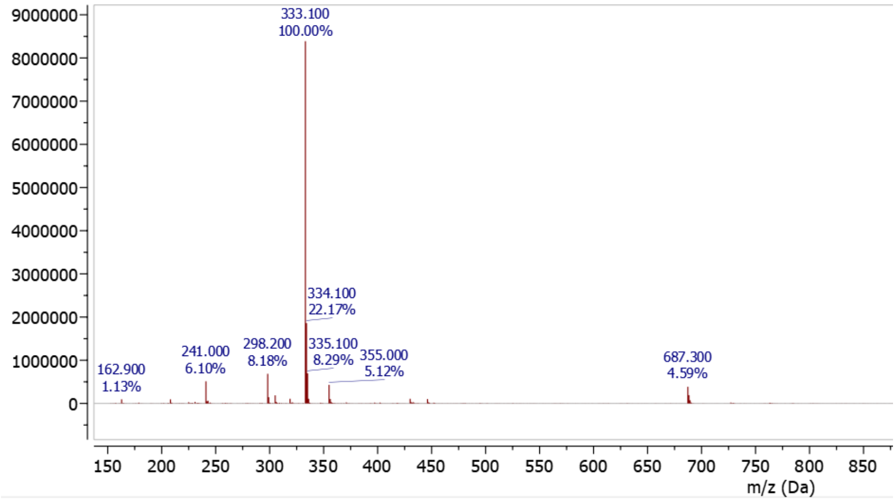

Figure 129. Mass spectrum ESI^{+} of compound ***23b***

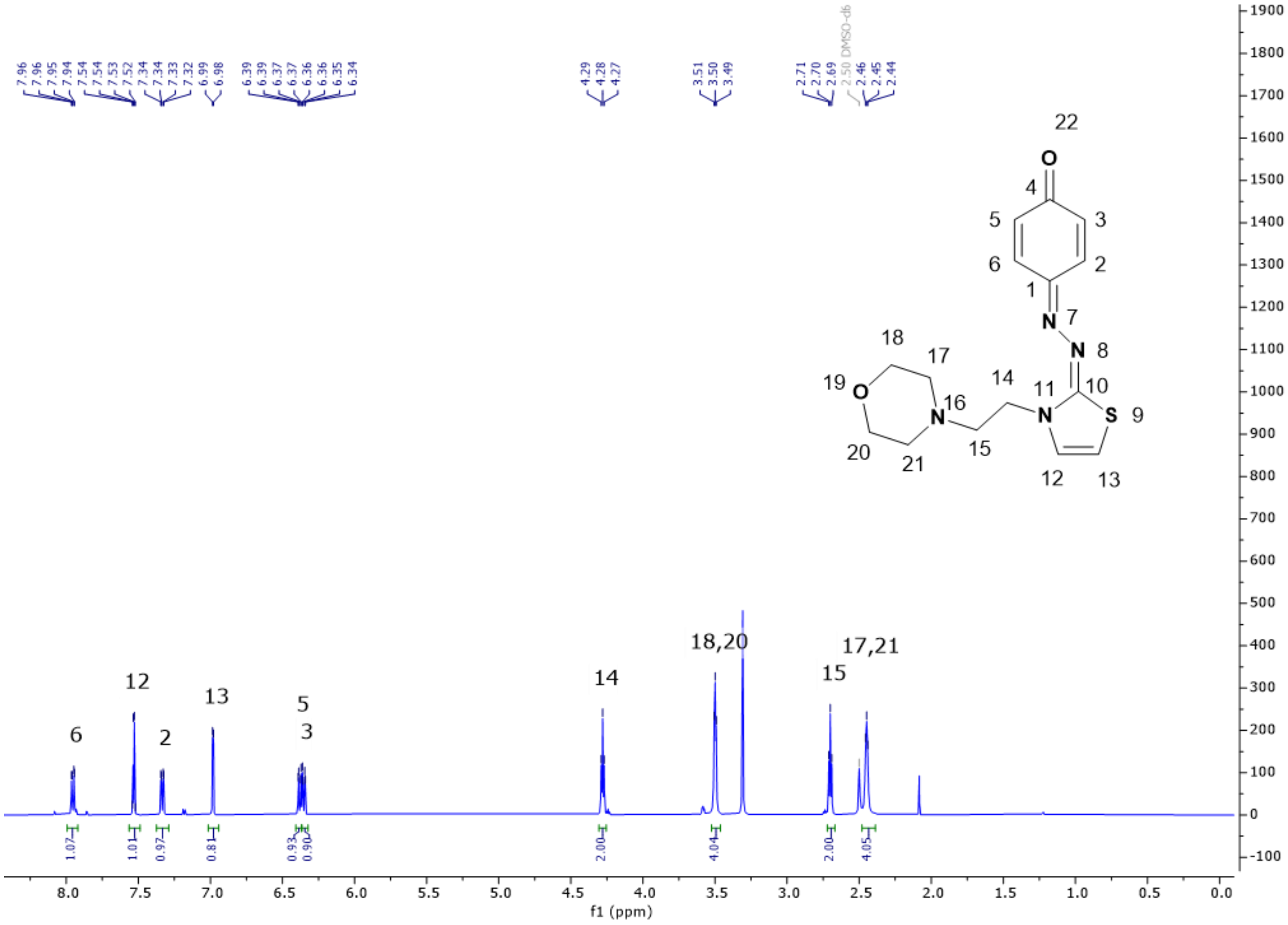

*Figure 130. ^{1}H NMR (600 MHz, DMSO-d6) spectrum of compound **24a***

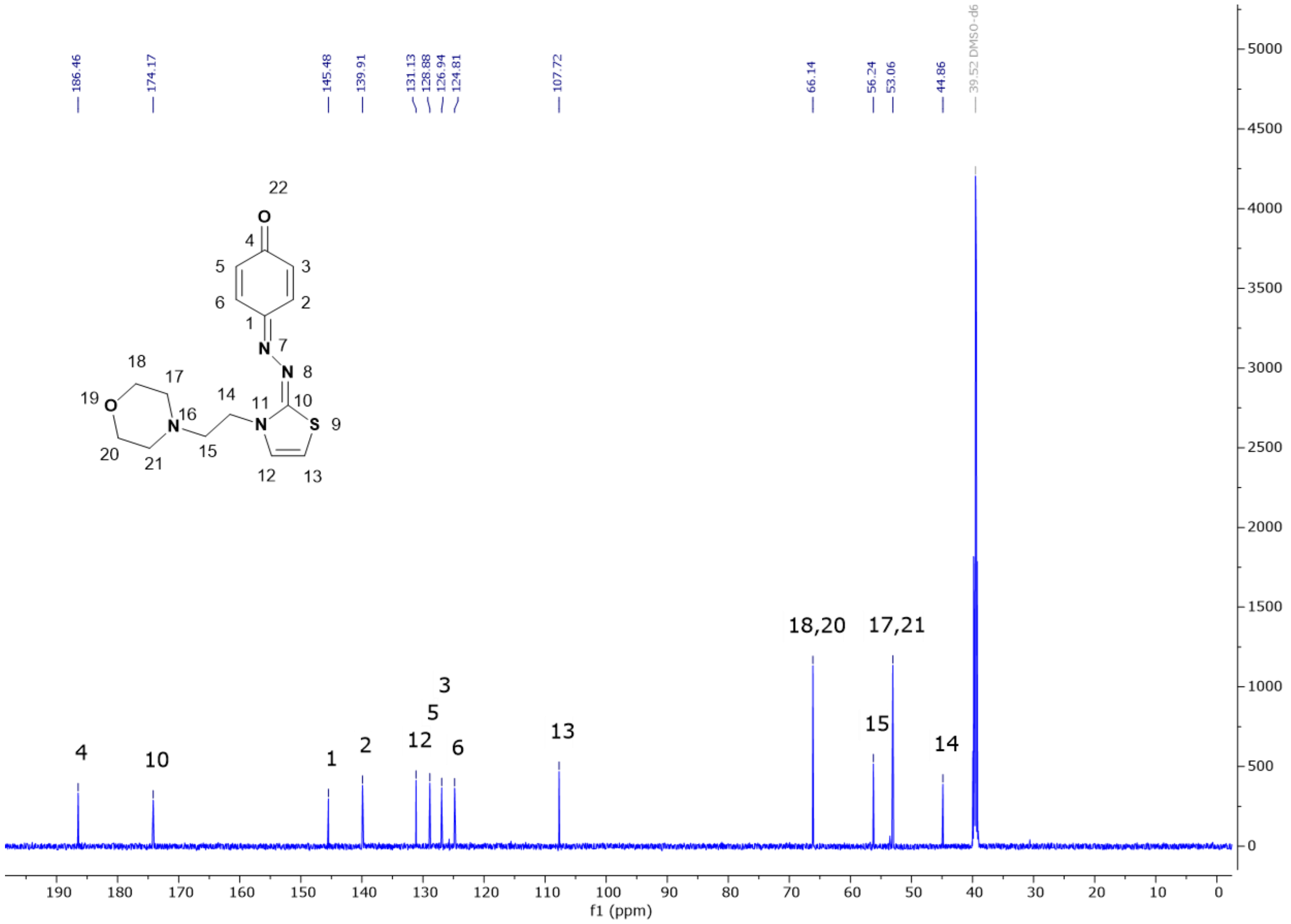

*Figure 131. ^{13}C NMR (150 MHz, DMSO-d6) spectrum of compound **24a***

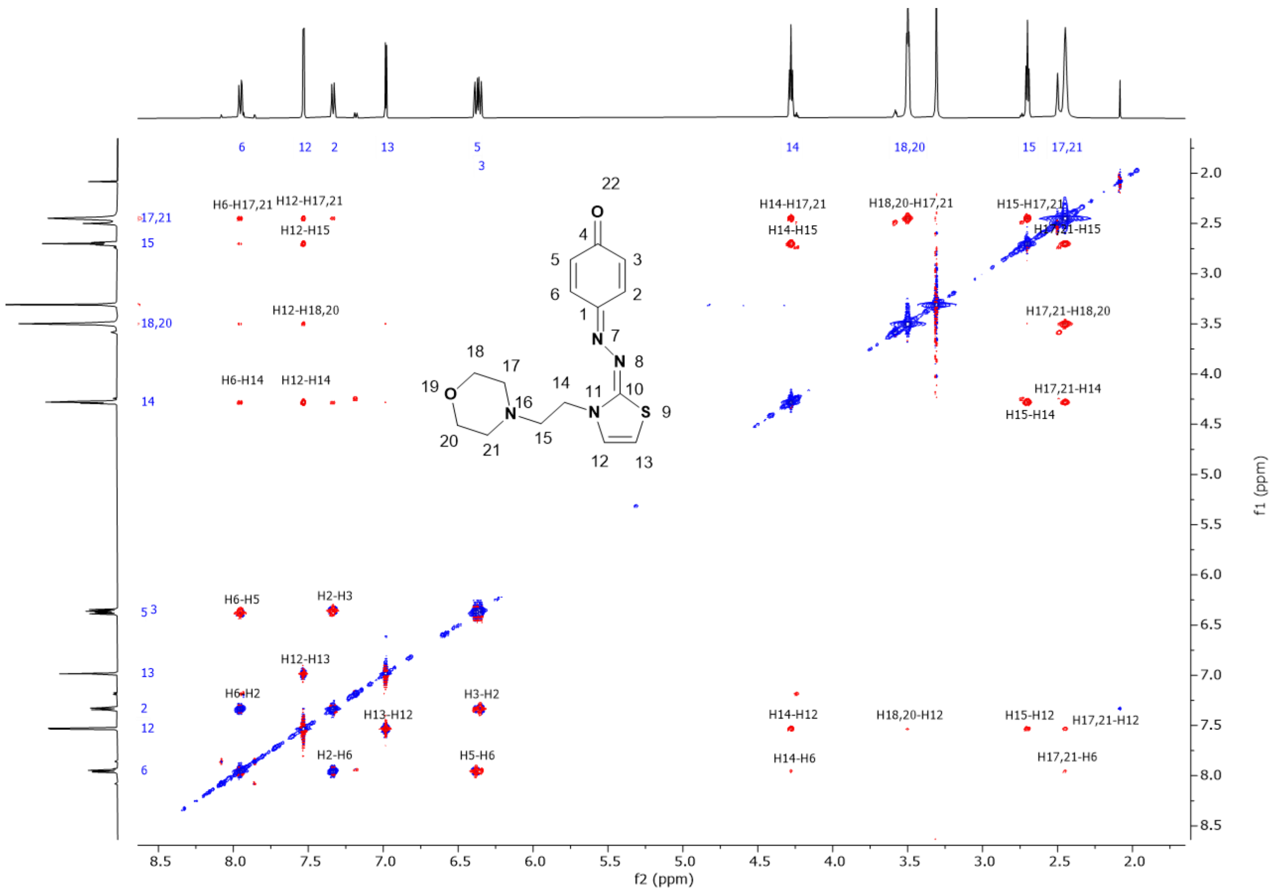

*Figure 132. ¹H,¹H-NOESY NMR (600 MHz, DMSO-d6) spectrum of compound **24a***

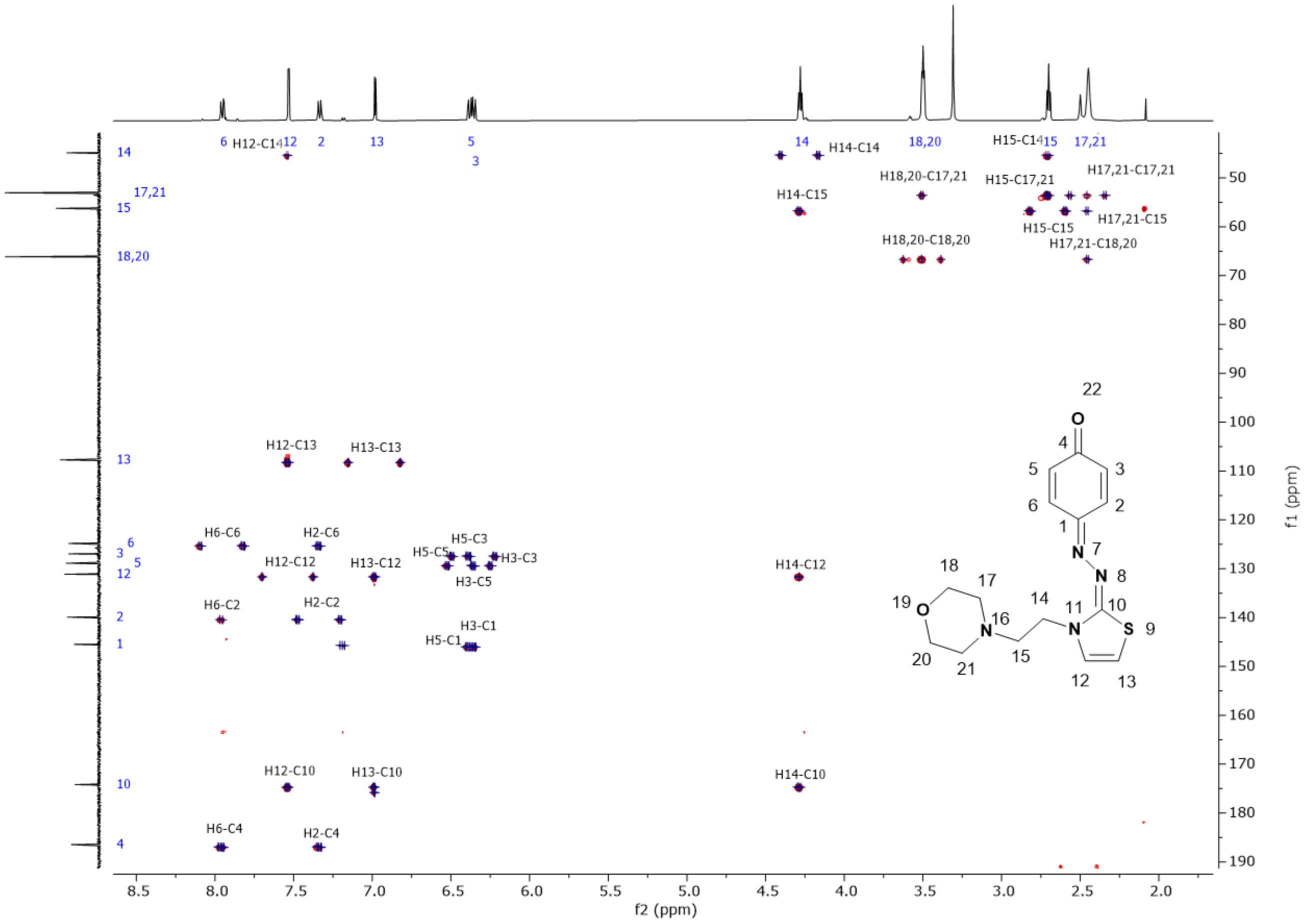

*Figure 133. ¹H,¹³C-HMBC NMR (DMSO-d6) spectrum of compound **24a***

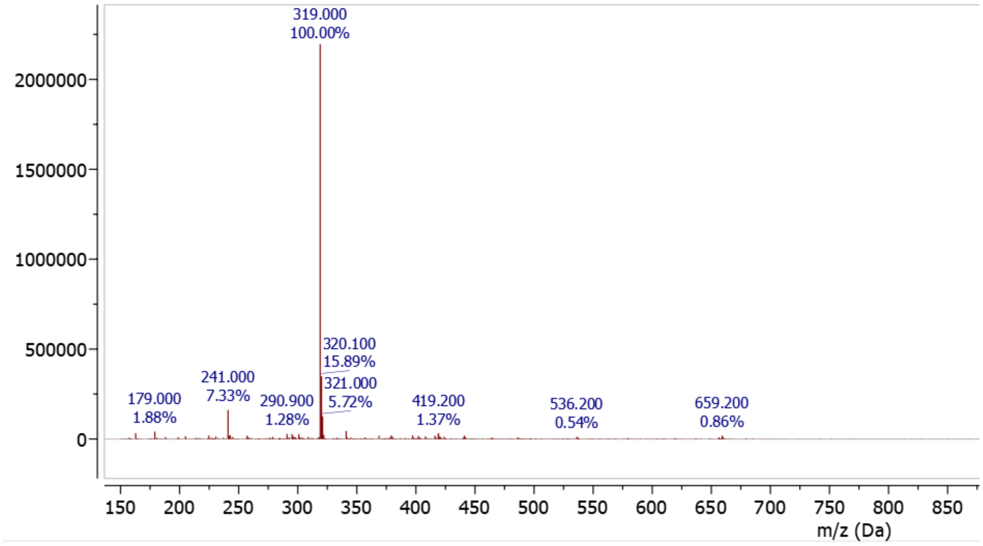

Figure 134. Mass spectrum ESI$^+$ of compound ***24a***

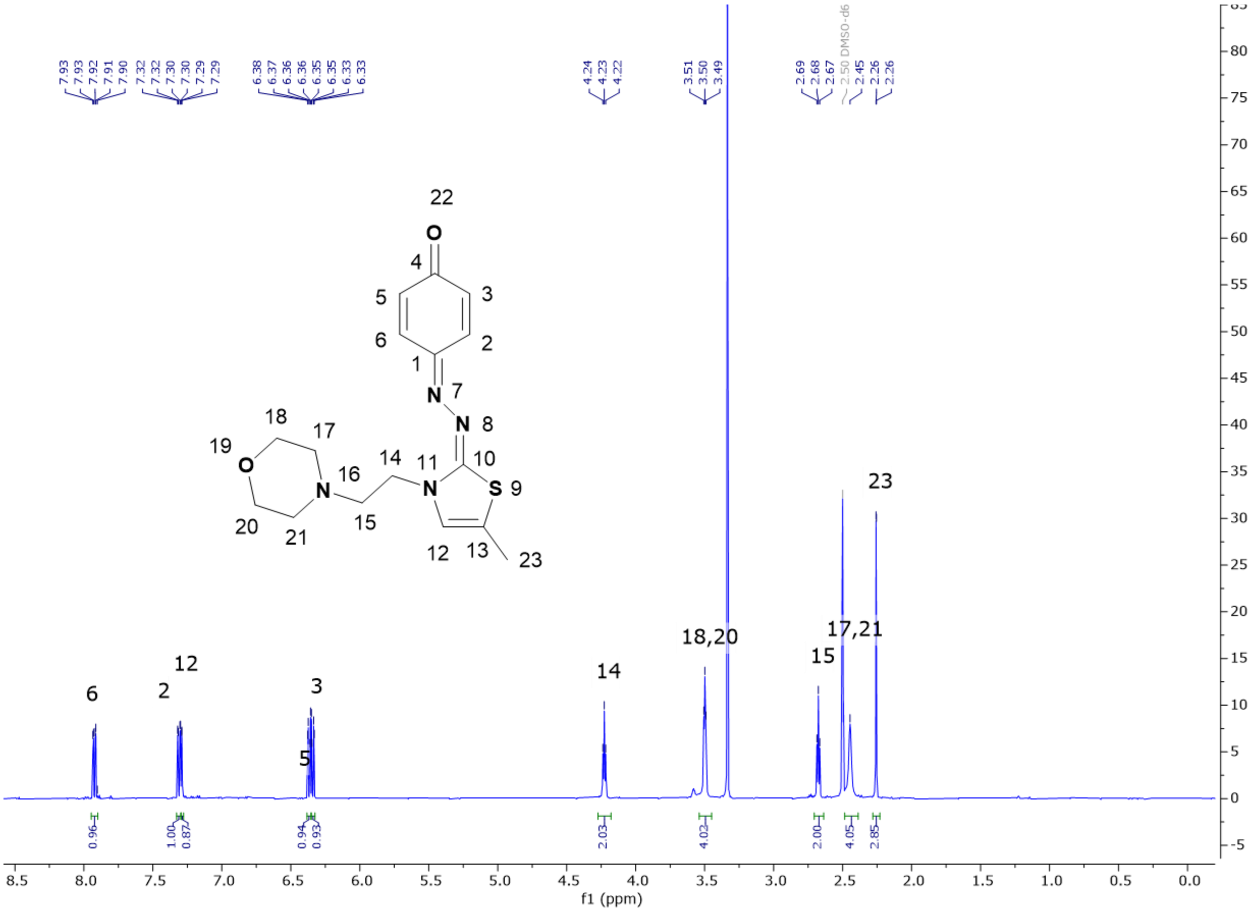

Figure 135. ^{1}H NMR (600 MHz, DMSO-d6) spectrum of compound ***24b***

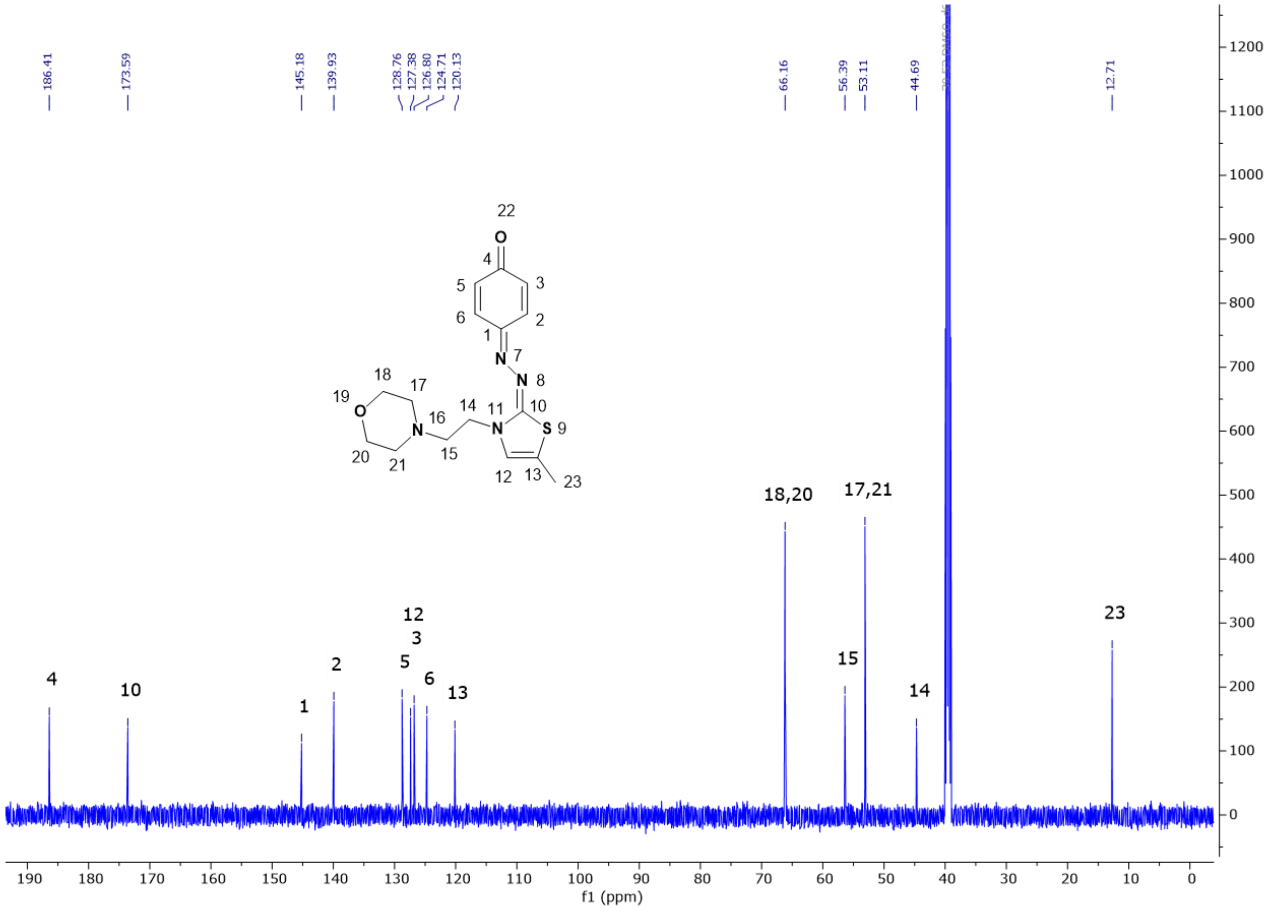

*Figure 136.^{13}C NMR (150 MHz, DMSO-d6) spectrum of compound **24b***

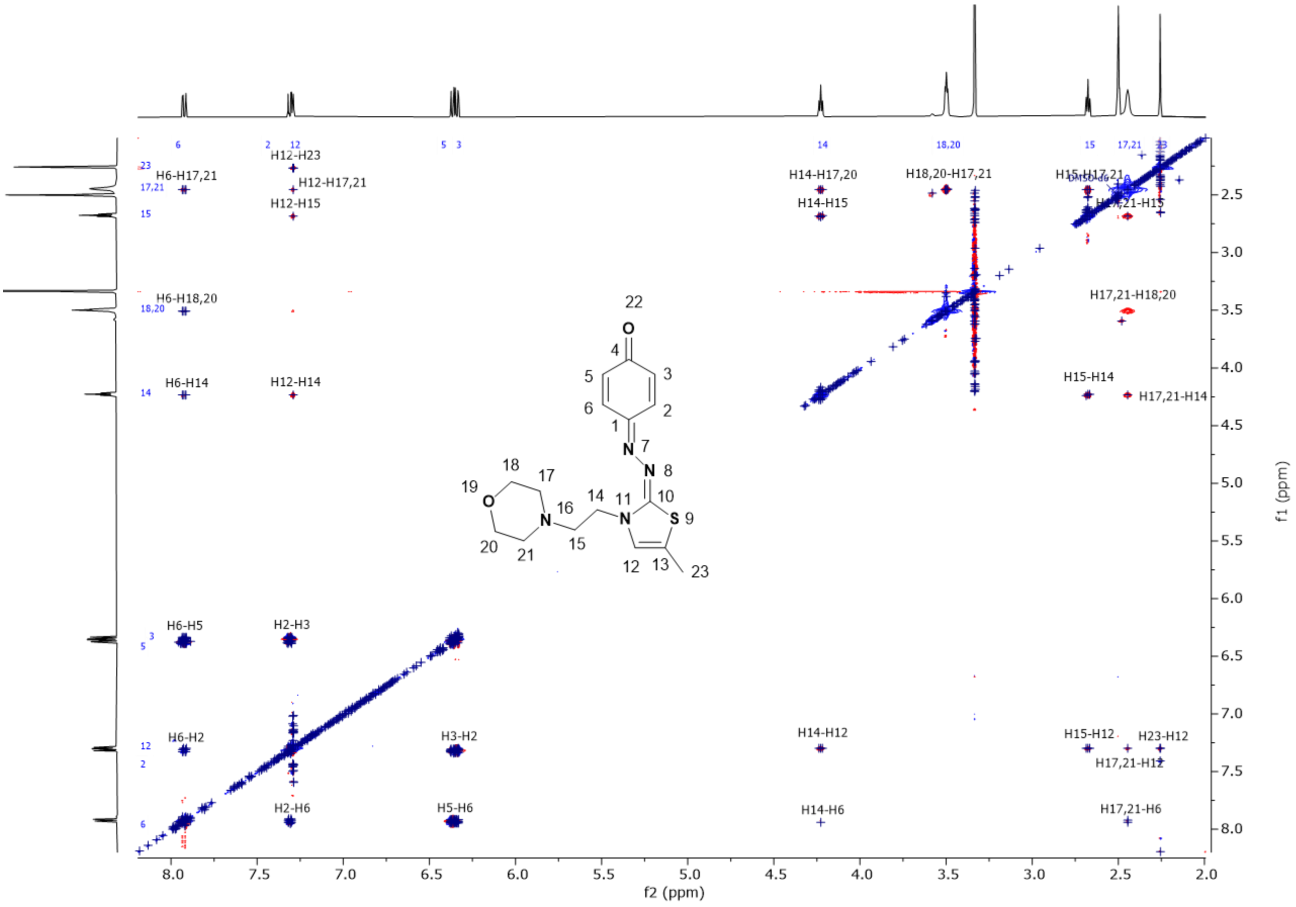

*Figure 137. ^{1}H,^{1}H-NOESY NMR (600 MHz, DMSO-d6) spectrum of compound **24b***

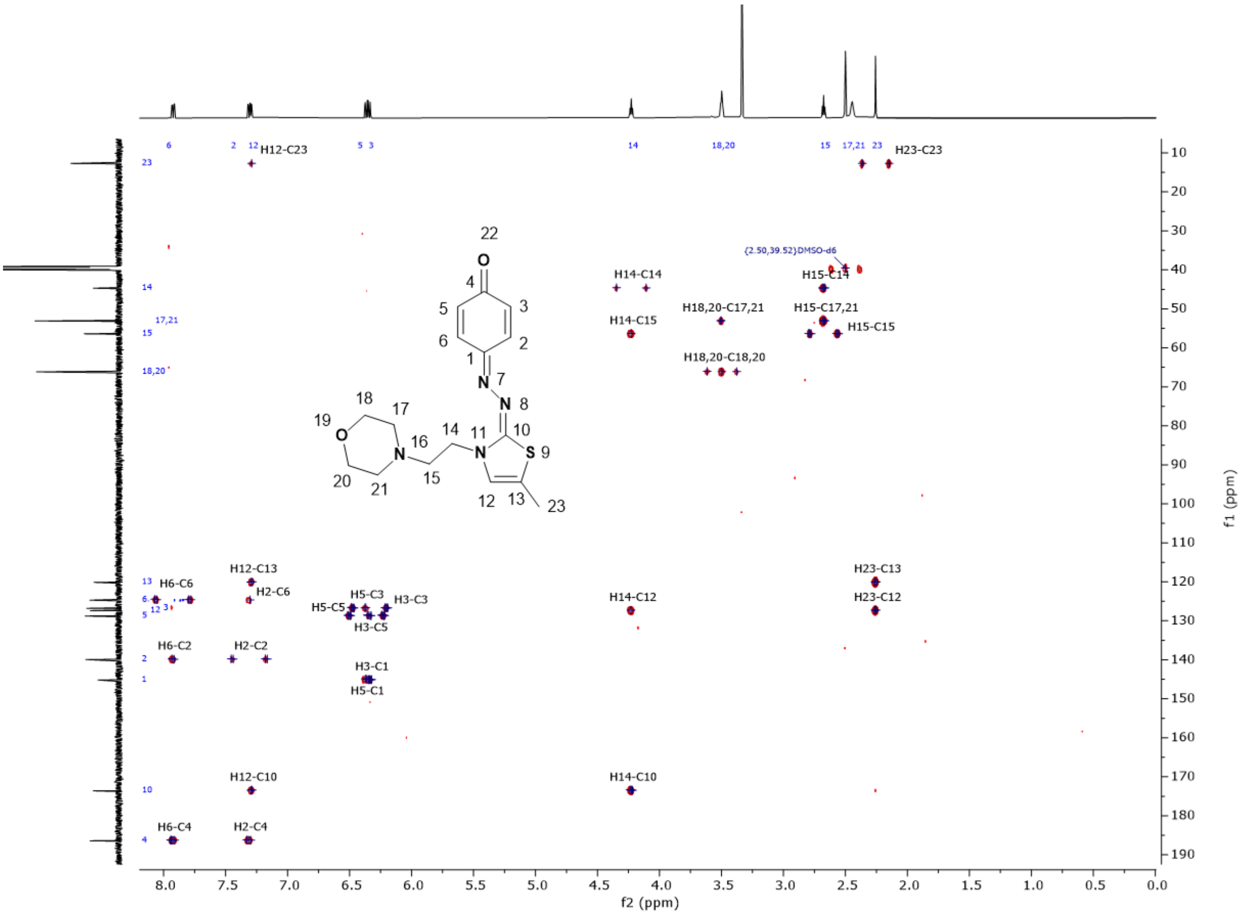

Figure 138. $^{1}H,^{13}C$-HMBC NMR (DMSO-d6) spectrum of compound ***24b***

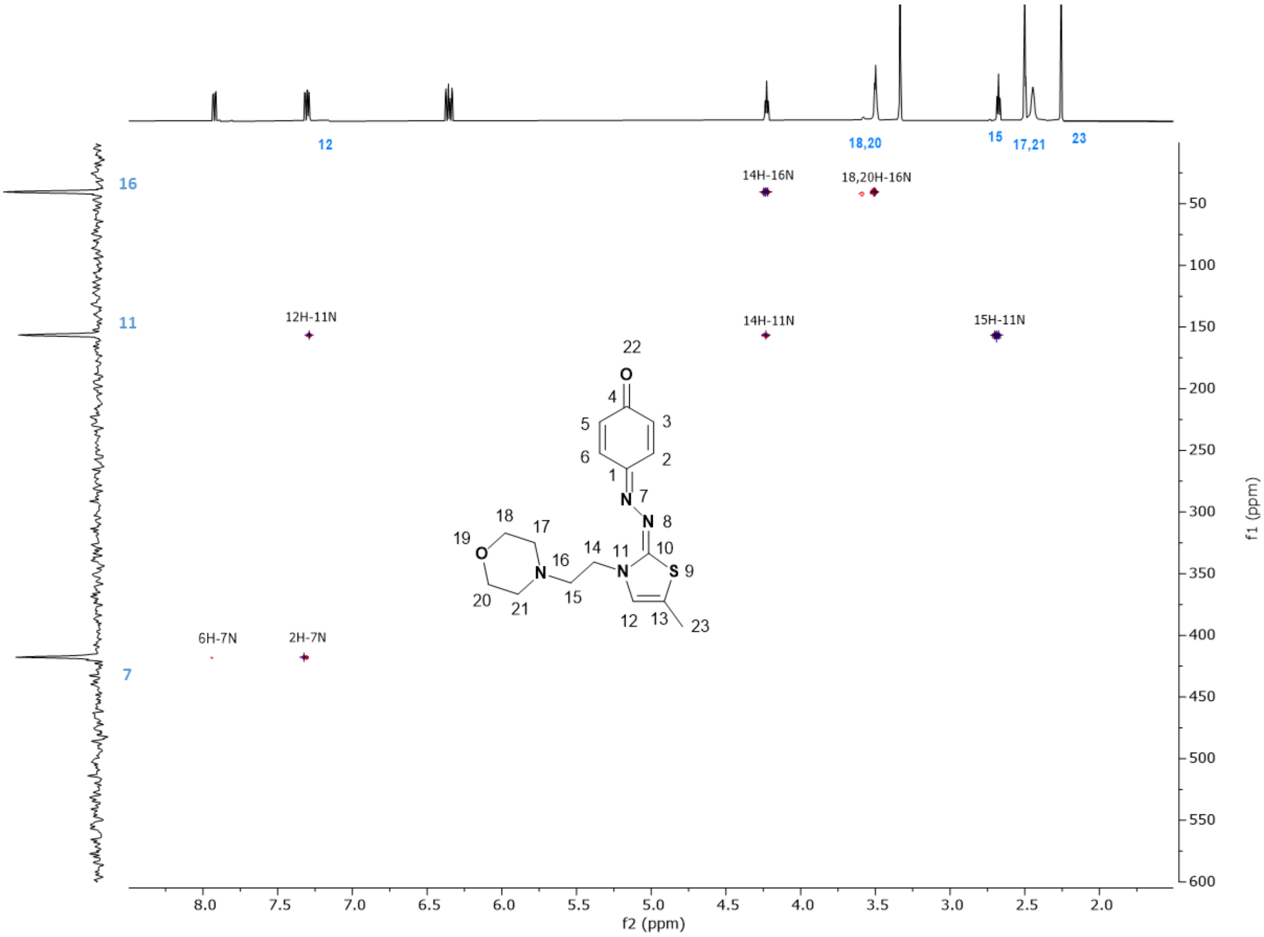

Figure 139. $^{1}H,^{15}N$-HMBC NMR (DMSO-d6) spectrum of compound ***24b***

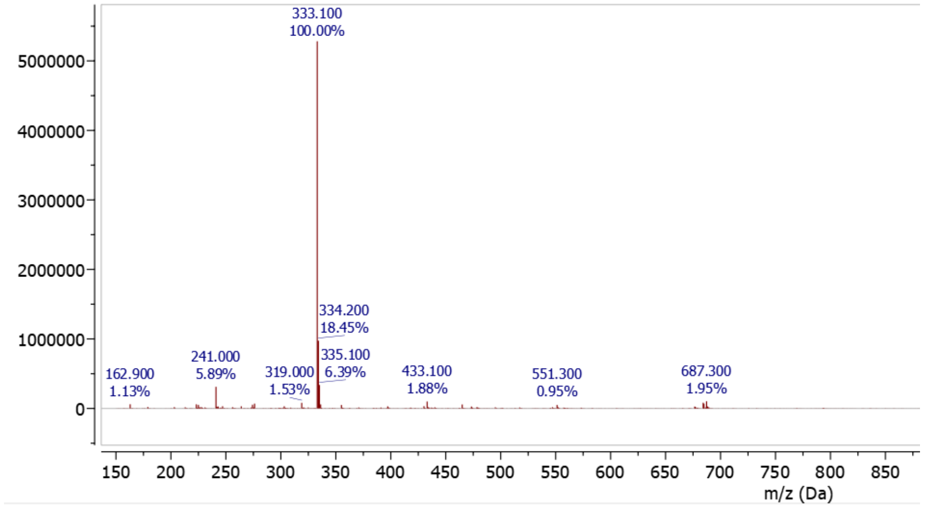

Figure 140. Mass spectrum ESI$^+$ of compound ***24b***

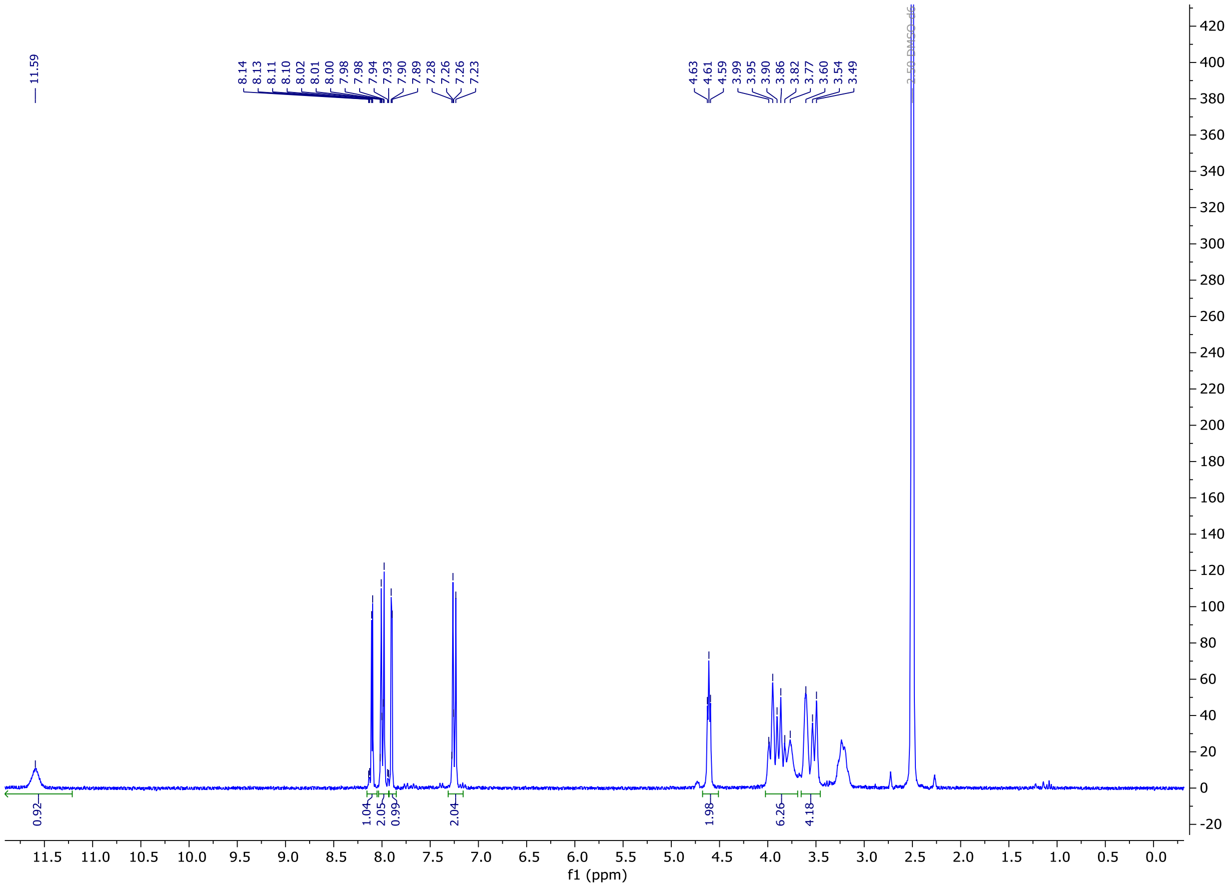

Figure 141.^{1}H NMR (300 MHz, DMSO-d6) spectrum of compound ***25a***

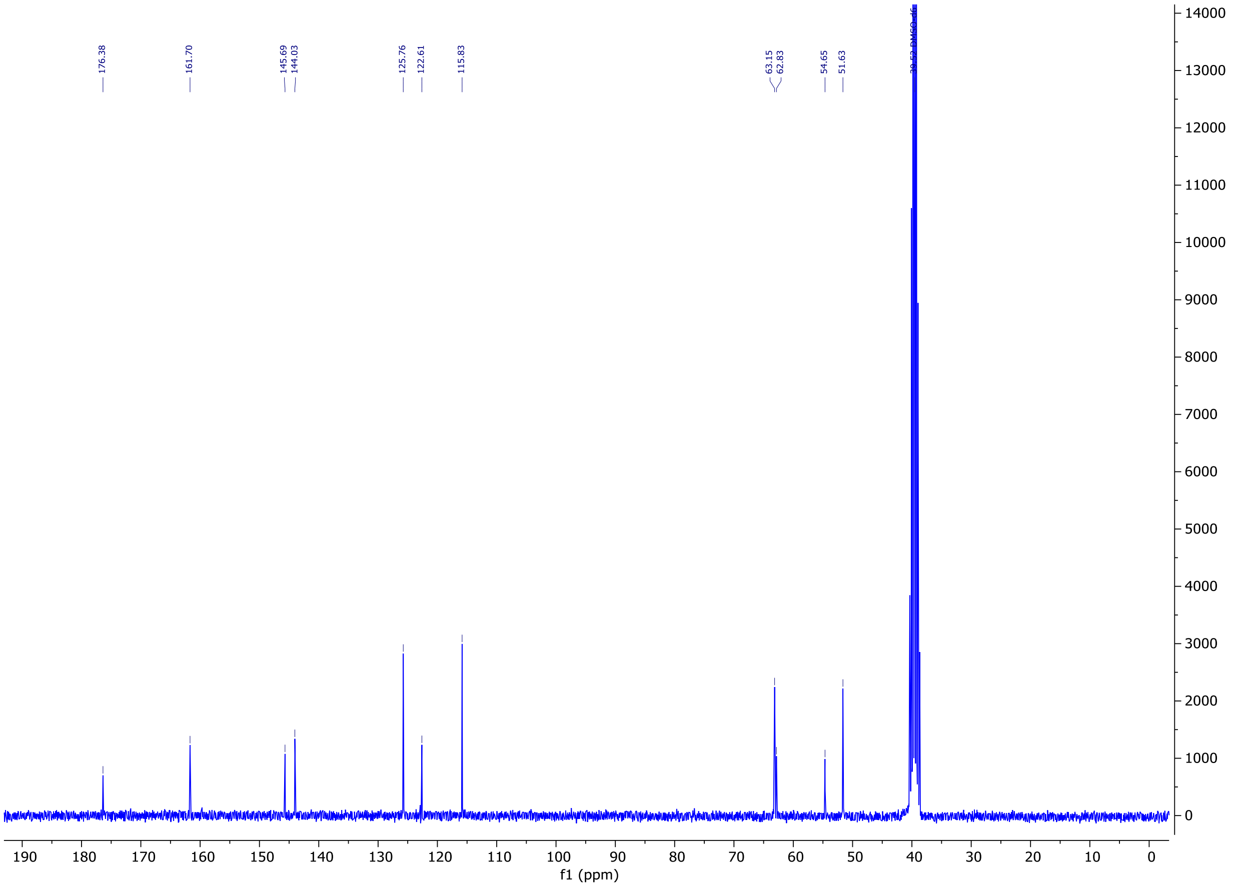

Figure 142. ^{13}C NMR (75 MHz, DMSO-d6) spectrum of compound ***25a***

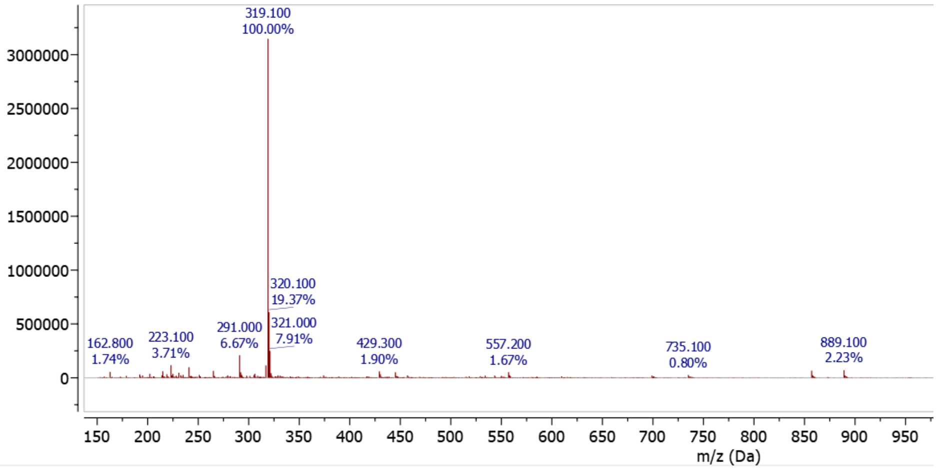

Figure 143. Mass spectrum ESI$^+$ of compound ***25a***

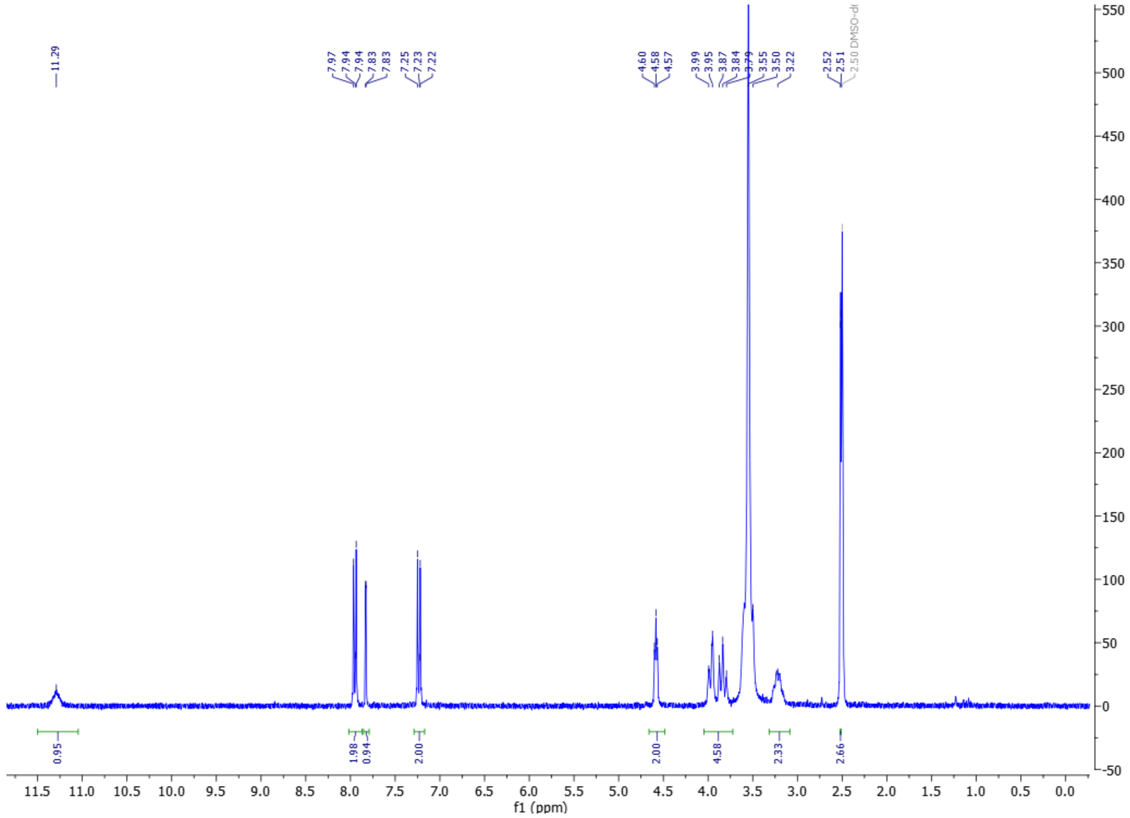

*Figure 144. ^{1}H NMR (300 MHz, DMSO-d6) spectrum of compound **25b***

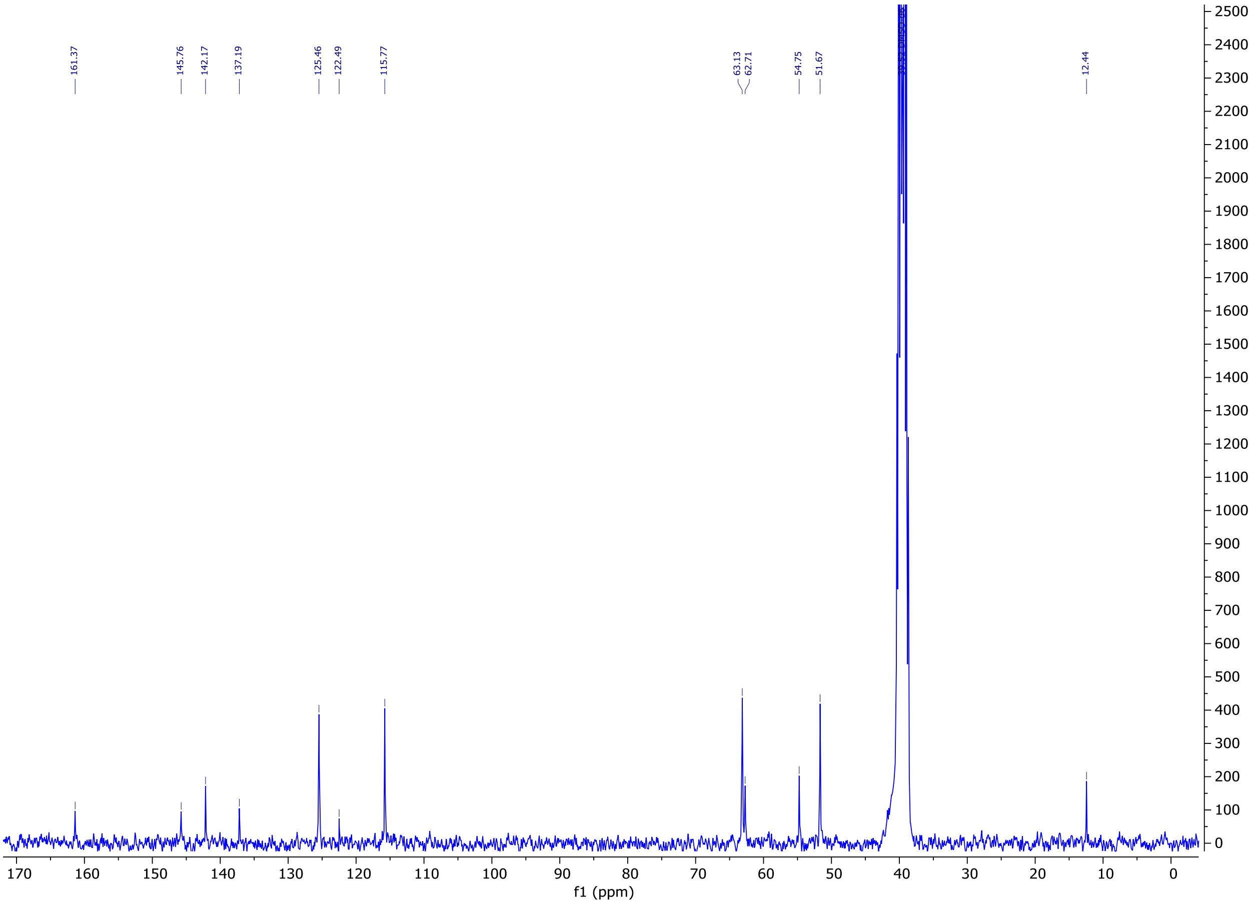

*Figure 145. ^{13}C NMR (75 MHz, DMSO-d6) spectrum of compound **25b***

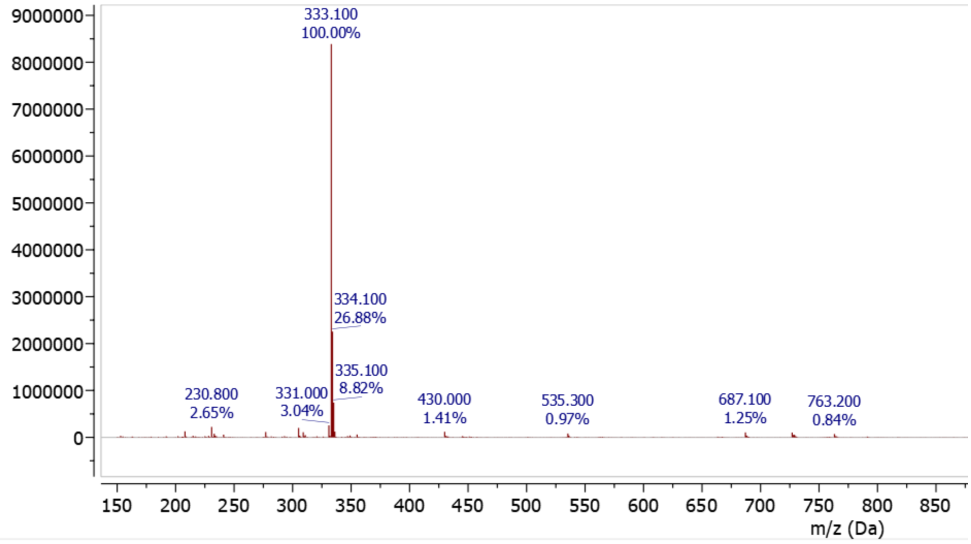

Figure 146. Mass spectrum ESI^{+} of compound ***25***

2. UV/Vis spectra and half-life determination

2.1 UV/Vis spectra and half-life determination in DMSO

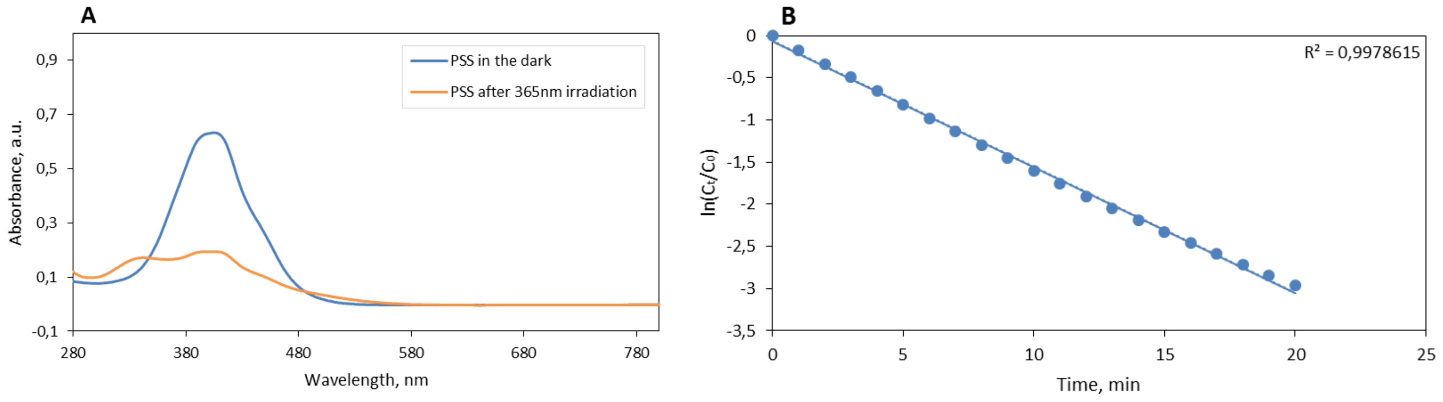

Figure 147. A. UV/Vis absorption spectra of compound **1** (PLA001) before and after irradiation (365 nm, 5 min) of its solution in DMSO. B. Linear fit of the change in absorbance at λ_{max} (350 nm) over time during compound **1** (PLA001) backward (*Z*-*E*) isomerization in DMSO.

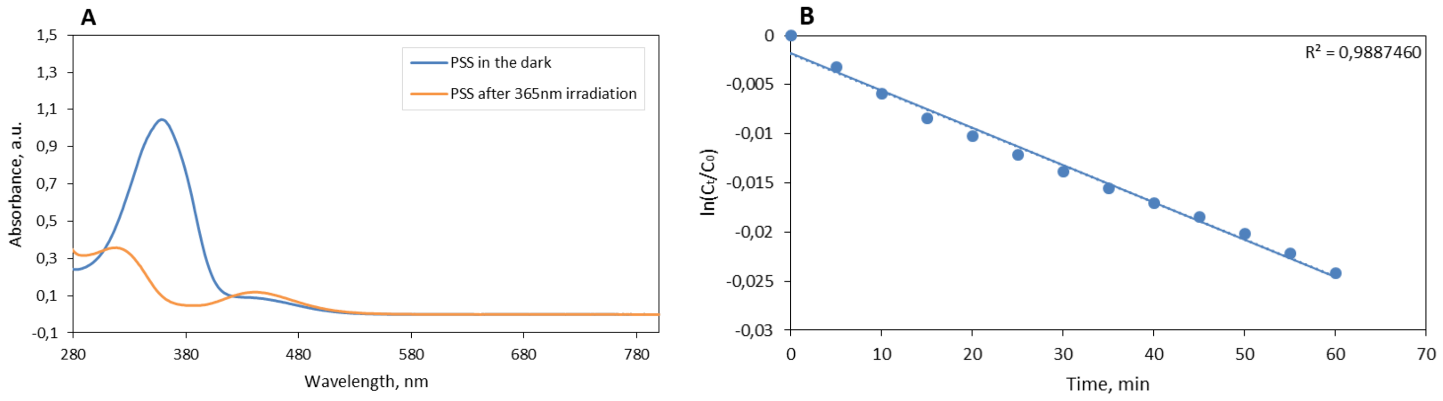

Figure 148. A. UV/Vis absorption spectra of compound **2** (PLA101) before and after irradiation (365 nm, 5 min) of its solution in DMSO. B. Linear fit of the change in absorbance at λ_{max} (359 nm) over time during compound **2** (PLA101) backward (*Z*-*E*) isomerization in DMSO.

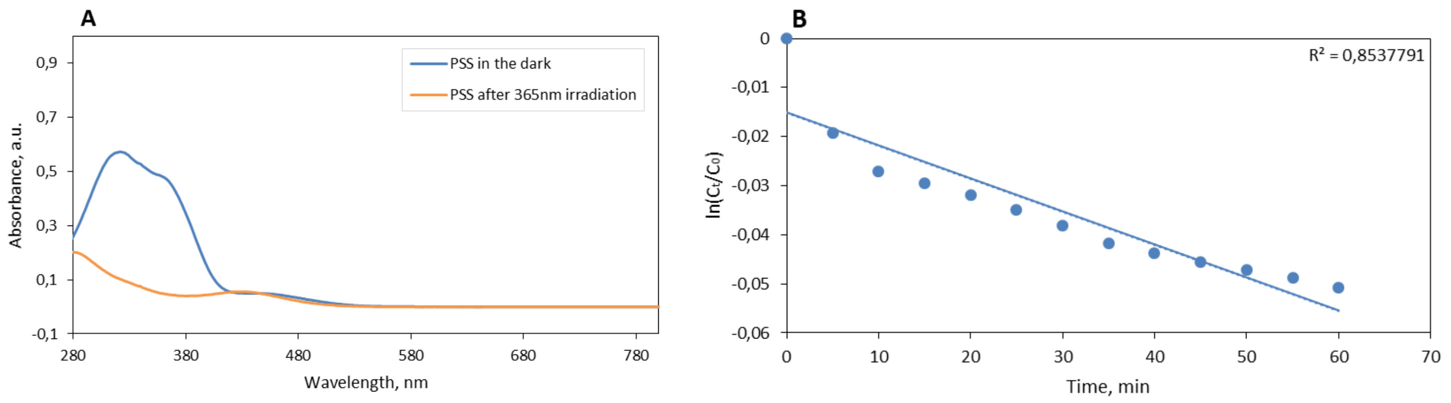

Figure 149. A. UV/Vis absorption spectra of compound **9a** (PLA002) before and after irradiation (365 nm, 5 min) of its solution in DMSO. B. Linear fit of the change in absorbance at λ_{max} (321 nm) over time during compound **9a** (PLA002) backward (*Z*-*E*) isomerization in DMSO.

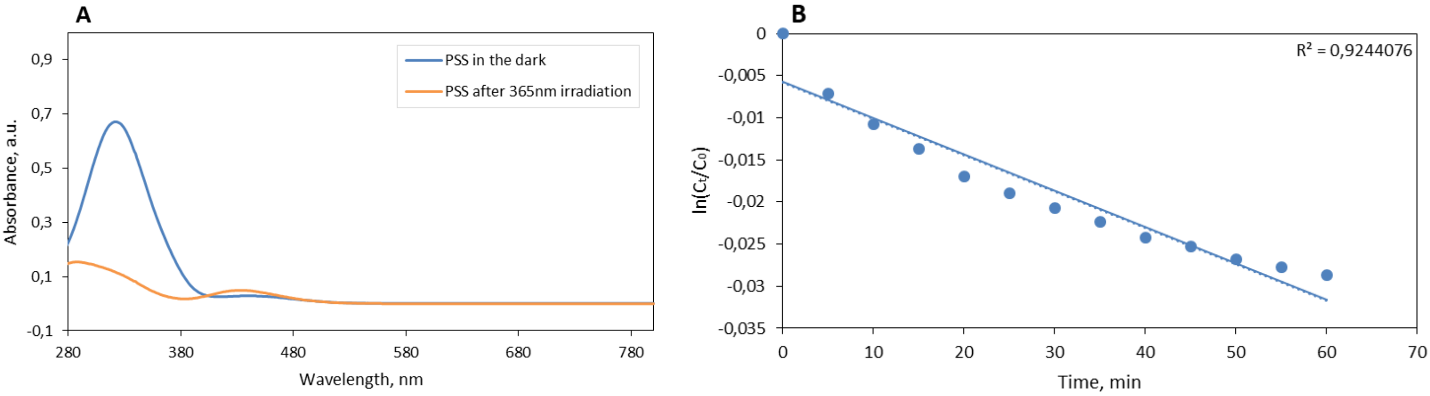

Figure 150. A. UV/Vis absorption spectra of compound **9b** (PLA003) before and after irradiation (365 nm, 5 min) of its solution in DMSO. B. Linear fit of the change in absorbance at λ_{max} (322 nm) over time during compound **9b** (PLA003) backward (Z-E) isomerization in DMSO.

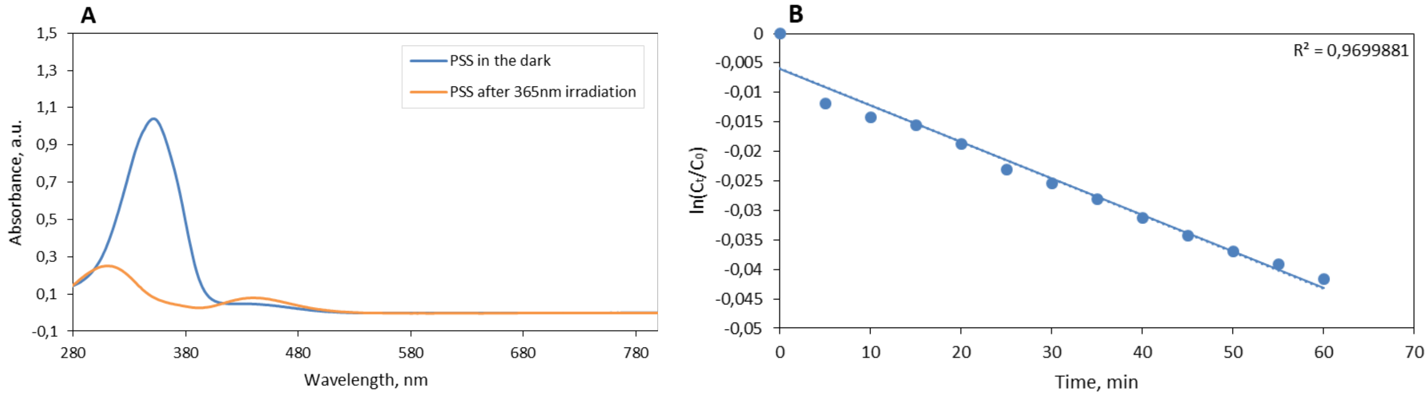

Figure 151. A. UV/Vis absorption spectra of compound **14** (PLA006) before and after irradiation (365 nm, 5 min) of its solution in DMSO. B. Linear fit of the change in absorbance at λ_{max} (352 nm) over time during compound **14** (PLA006) backward (Z-E) isomerization in DMSO.

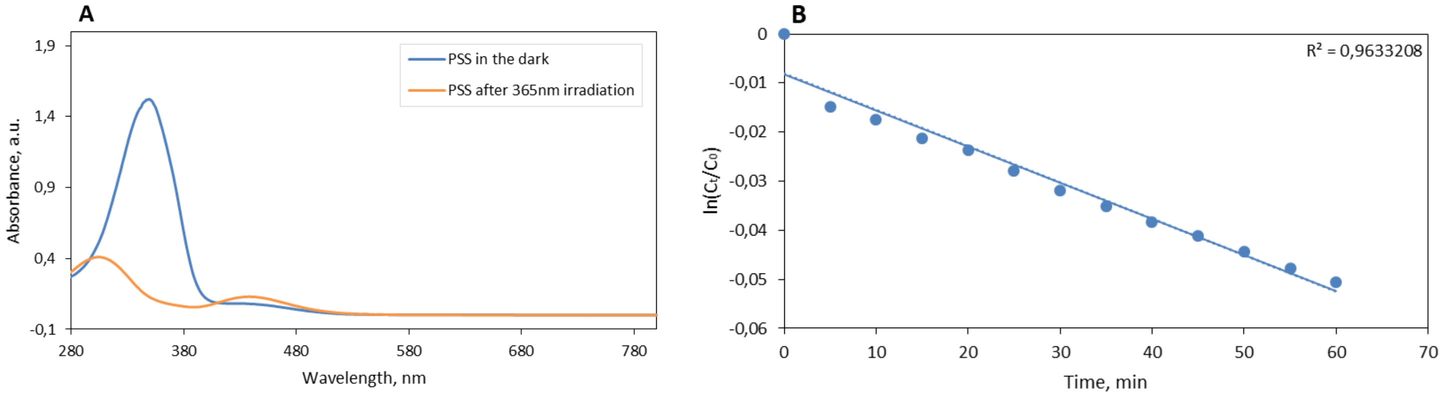

Figure 152. A. UV/Vis absorption spectra of compound **16** (PLA005) before and after irradiation (365 nm, 5 min) of its solution in DMSO. B. Linear fit of the change in absorbance at λ_{max} (349 nm) over time during compound **16** (PLA005) backward (Z-E) isomerization in DMSO.

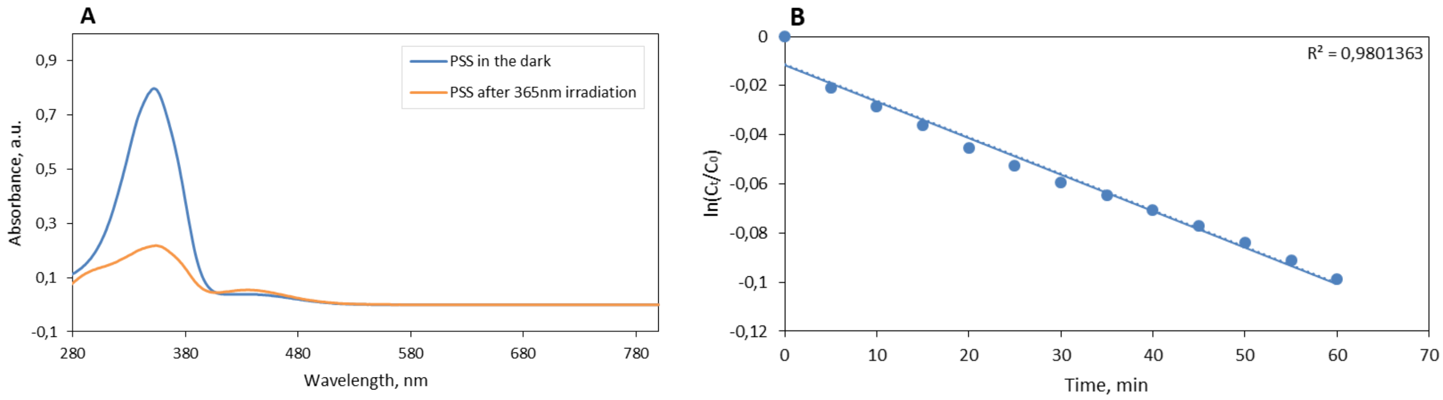

Figure 153. A. UV/Vis absorption spectra of compound **20a** (PLA206) before and after irradiation (365 nm, 5 min) of its solution in DMSO. B. Linear fit of the change in absorbance at λ_{max} (352 nm) over time during compound **20a** (PLA206) backward (Z-E) isomerization in DMSO.

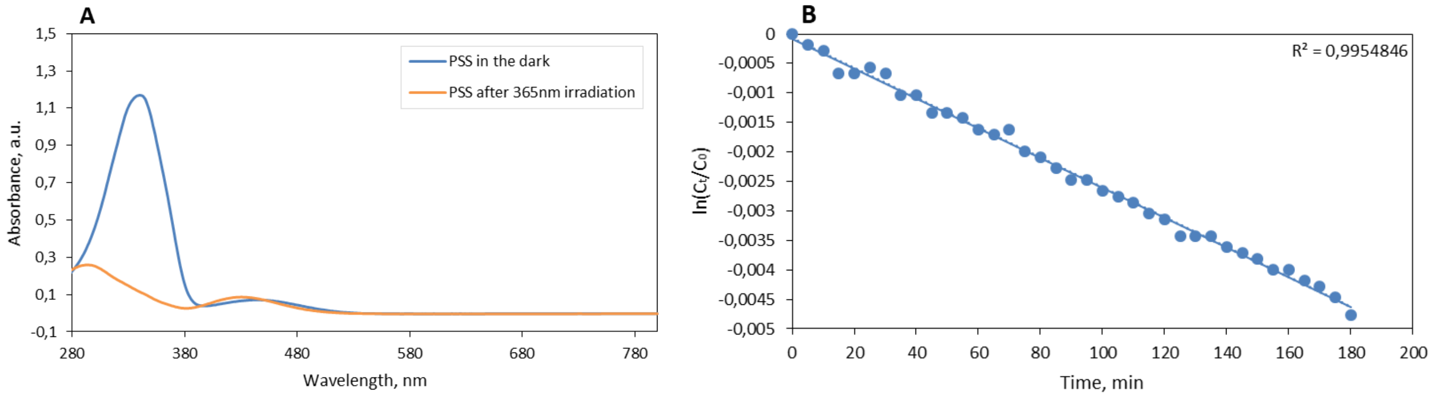

Figure 154. A. UV/Vis absorption spectra of compound **20b** (PLA203) before and after irradiation (365 nm, 5 min) of its solution in DMSO. B. Linear fit of the change in absorbance at λ_{max} (340 nm) over time during compound **20b** (PLA203) backward (Z-E) isomerization in DMSO.

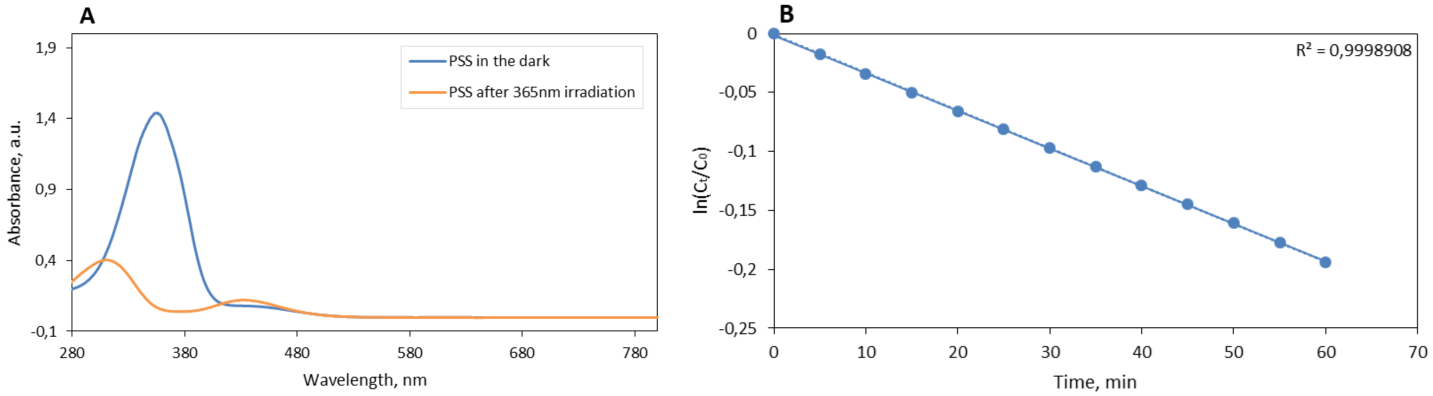

Figure 155. A. UV/Vis absorption spectra of compound **20c** (PLA201) before and after irradiation (365 nm, 5 min) of its solution in DMSO. B. Linear fit of the change in absorbance at λ_{max} (356 nm) over time during compound **20c** (PLA201) backward (Z-E) isomerization in DMSO.

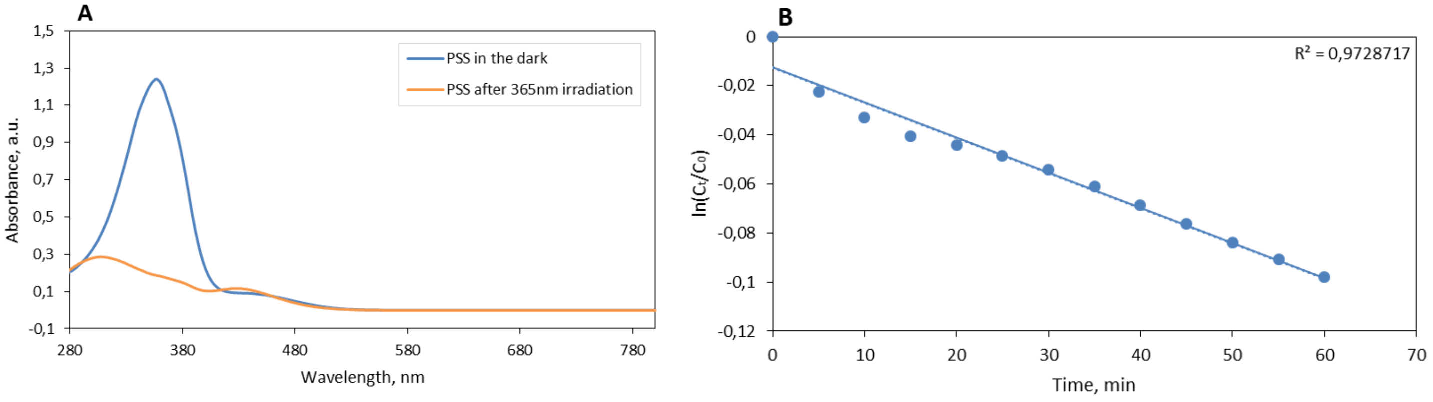

Figure 156. A. UV/Vis absorption spectra of compound **20d** (PLA207) before and after irradiation (365 nm, 5 min) of its solution in DMSO. B. Linear fit of the change in absorbance at λ_{max} (357 nm) over time during compound **20d** (PLA207) backward (Z-E) isomerization in DMSO.

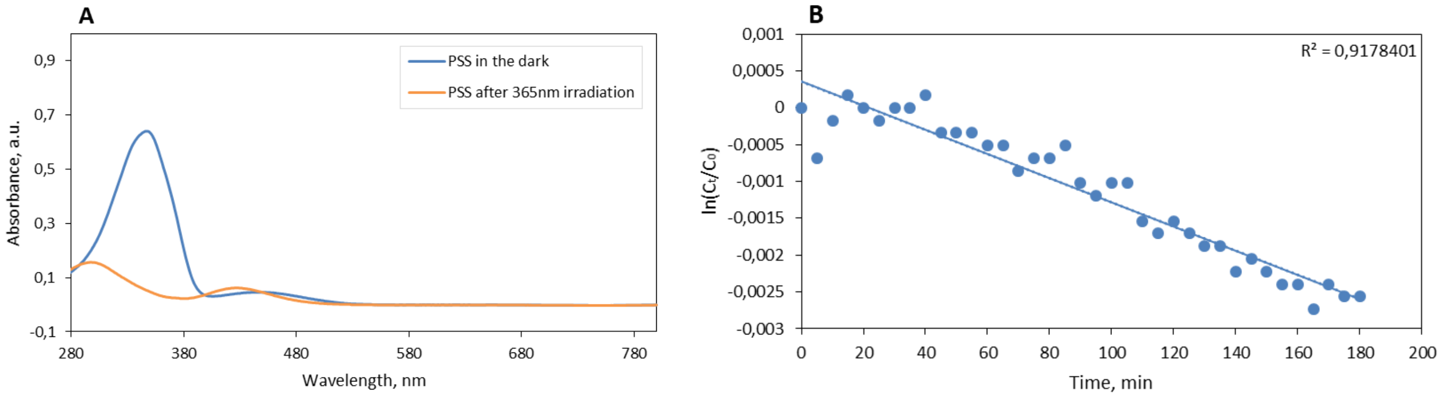

Figure 157. A. UV/Vis absorption spectra of compound **20e** (PLA204) before and after irradiation (365 nm, 5 min) of its solution in DMSO. B. Linear fit of the change in absorbance at λ_{max} (347 nm) over time during compound **20e** (PLA204) backward (Z-E) isomerization in DMSO.

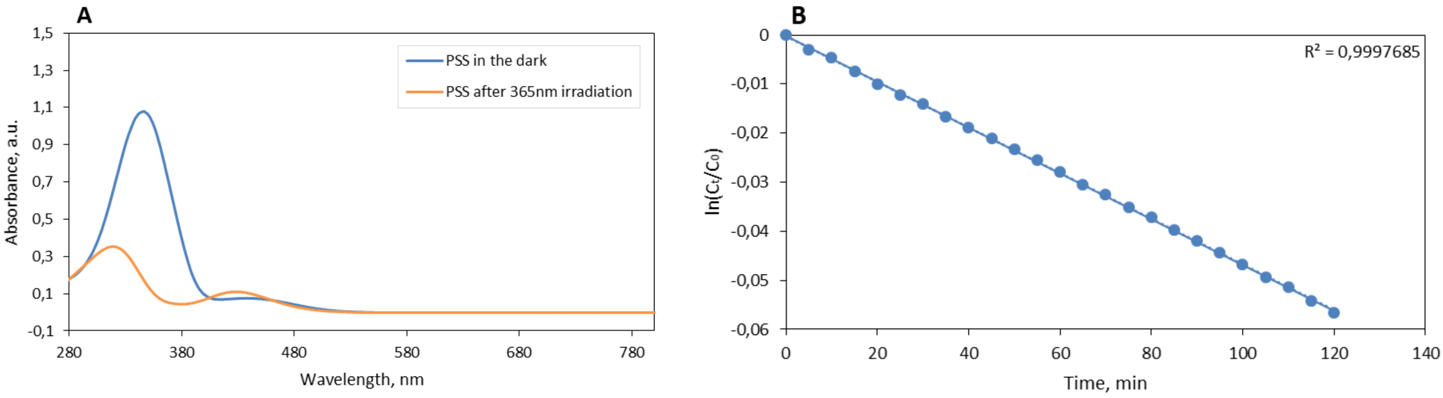

Figure 158. A. UV/Vis absorption spectra of compound **20f** (PLA202) before and after irradiation (365 nm, 5 min) of its solution in DMSO. B. Linear fit of the change in absorbance at λ_{max} (346 nm) over time during compound **20f** (PLA202) backward (Z-E) isomerization in DMSO.

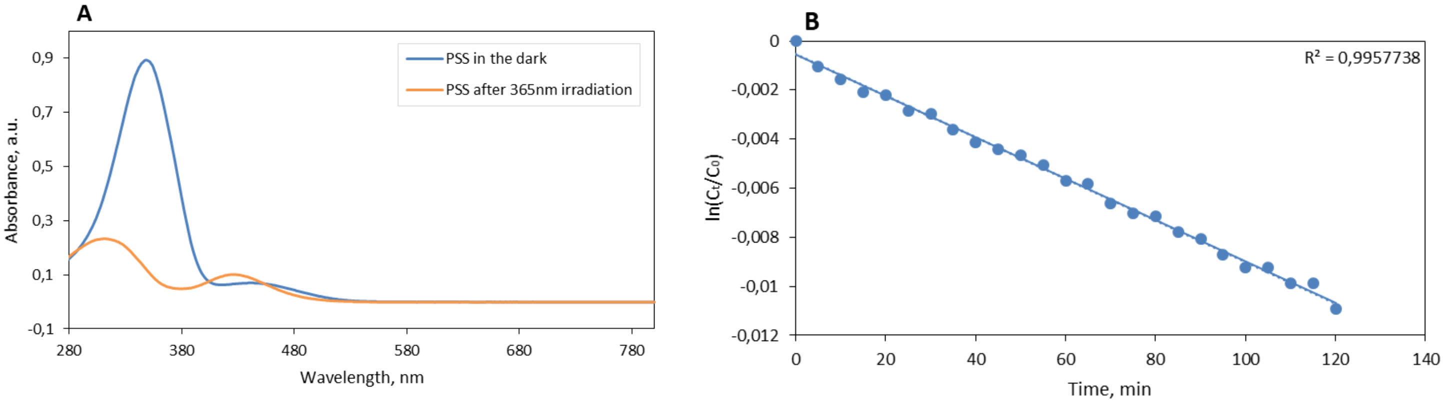

Figure 159. A. UV/Vis absorption spectra of compound **20g** (PLA208) before and after irradiation (365 nm, 5 min) of its solution in DMSO. B. Linear fit of the change in absorbance at λ_{max} (349 nm) over time during compound **20g** (PLA208) backward (Z-E) isomerization in DMSO.

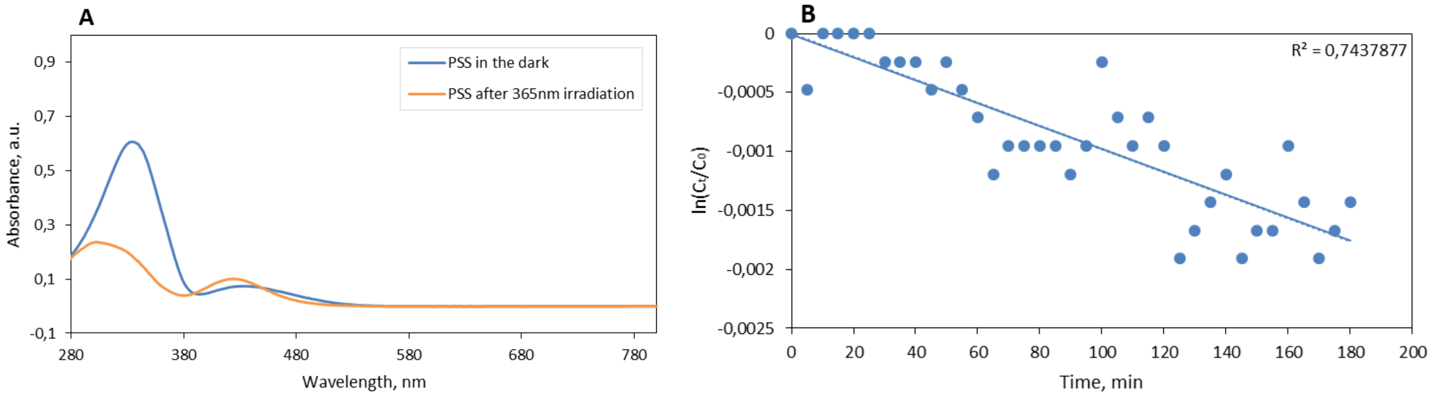

Figure 160. A. UV/Vis absorption spectra of compound **20h** (PLA205) before and after irradiation (365 nm, 5 min) of its solution in DMSO. B. Linear fit of the change in absorbance at λ_{max} (334 nm) over time during compound **20h** (PLA205) backward (Z-E) isomerization in DMSO.

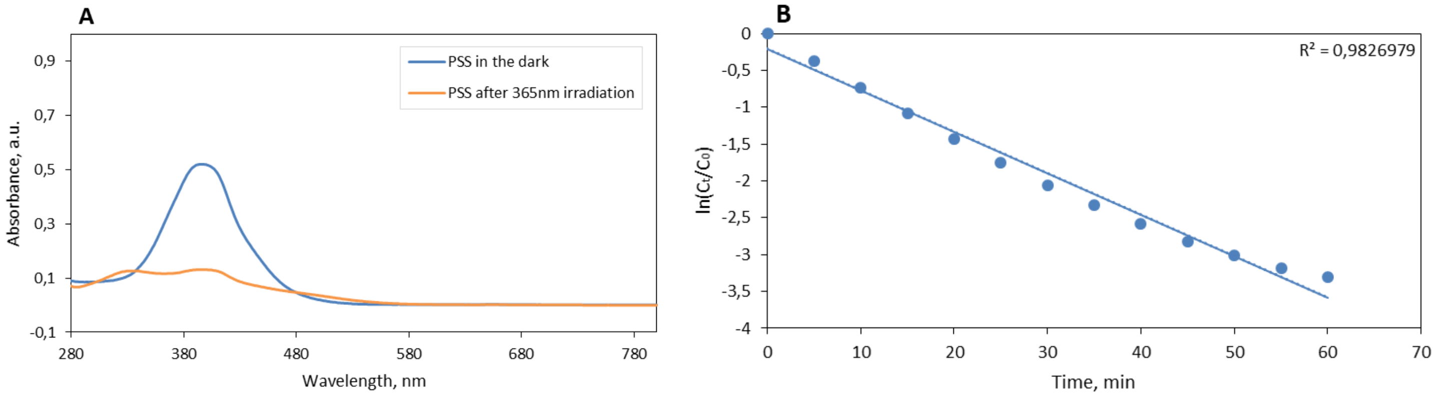

Figure 161. A. UV/Vis absorption spectra of compound **25a** (PLA401) before and after irradiation (365 nm, 5 min) of its solution in DMSO. B. Linear fit of the change in absorbance at λ_{max} (396 nm) over time during compound **25a** (PLA401) backward (Z-E) isomerization in DMSO.

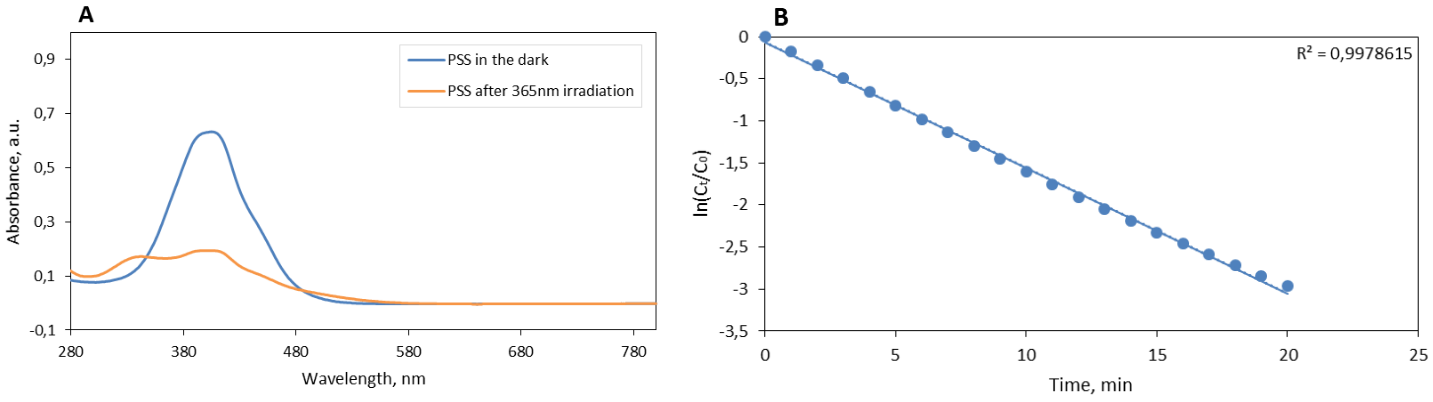

Figure 162. A. UV/Vis absorption spectra of compound **25b** (PLA402) before and after irradiation (365 nm, 5 min) of its solution in DMSO. B. Linear fit of the change in absorbance at λ_{max} (405 nm) over time during compound **25b** (PLA402) backward (Z-E) isomerization in DMSO.

2.2 UV/Vis spectra and half-life determination in aqueous solutions

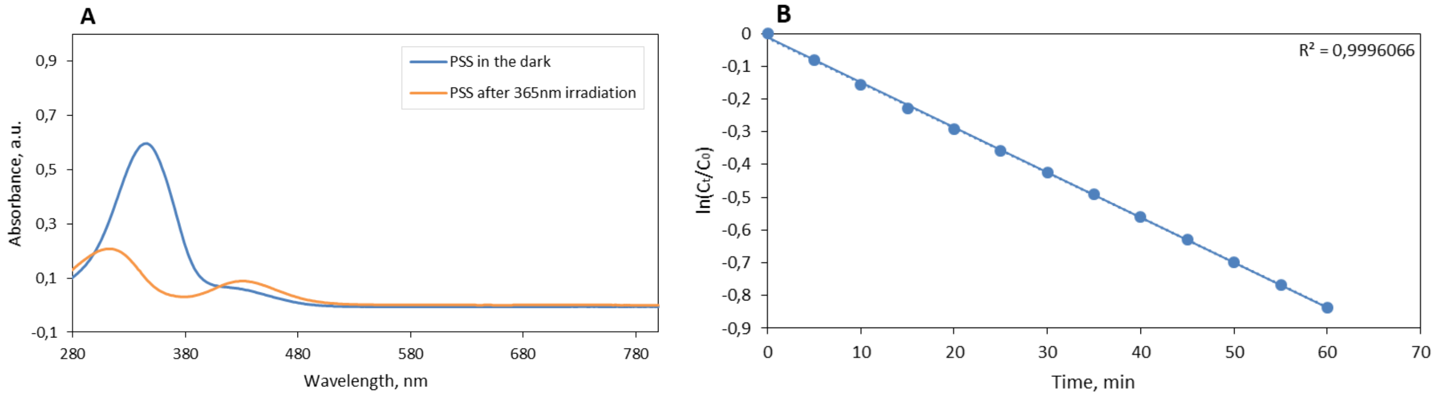

Figure 163. A. UV/Vis absorption spectra of compound **1** (PLA001) before and after irradiation (365 nm, 5 min) of its aqueous Kolliphor ELP solution. B. Linear fit of the change in absorbance at λ_{max} (345 nm) over time during compound **1** (PLA001) backward (*Z-E*) isomerization in aqueous Kolliphor ELP solution.

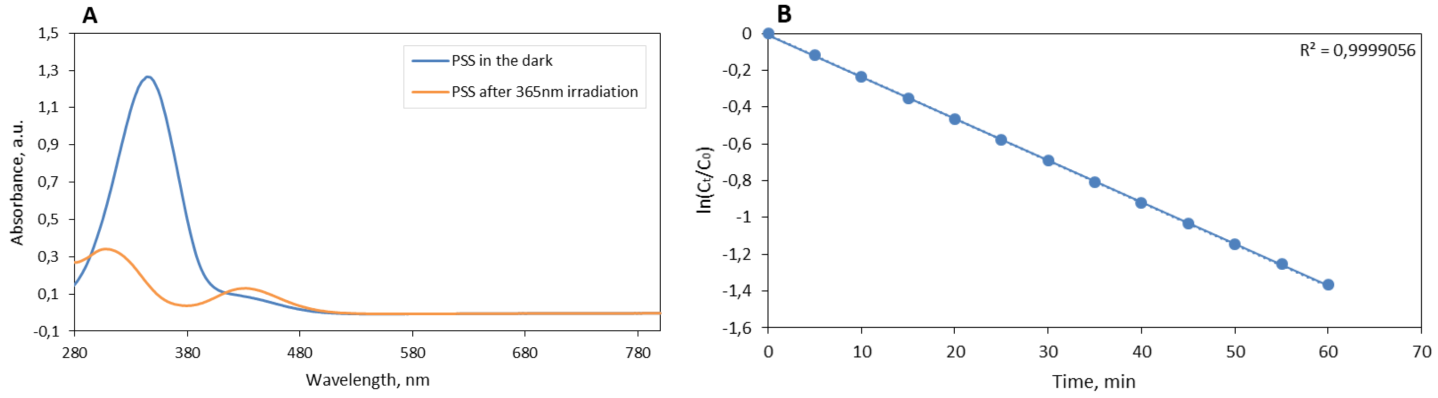

Figure 164. A. UV/Vis absorption spectra of compound **2** (PLA101) before and after irradiation (365 nm, 5 min) of its aqueous Kolliphor ELP solution. B. Linear fit of the change in absorbance at λ_{max} (345 nm) over time during compound **2** (PLA101) backward (*Z-E*) isomerization in aqueous Kolliphor ELP solution.

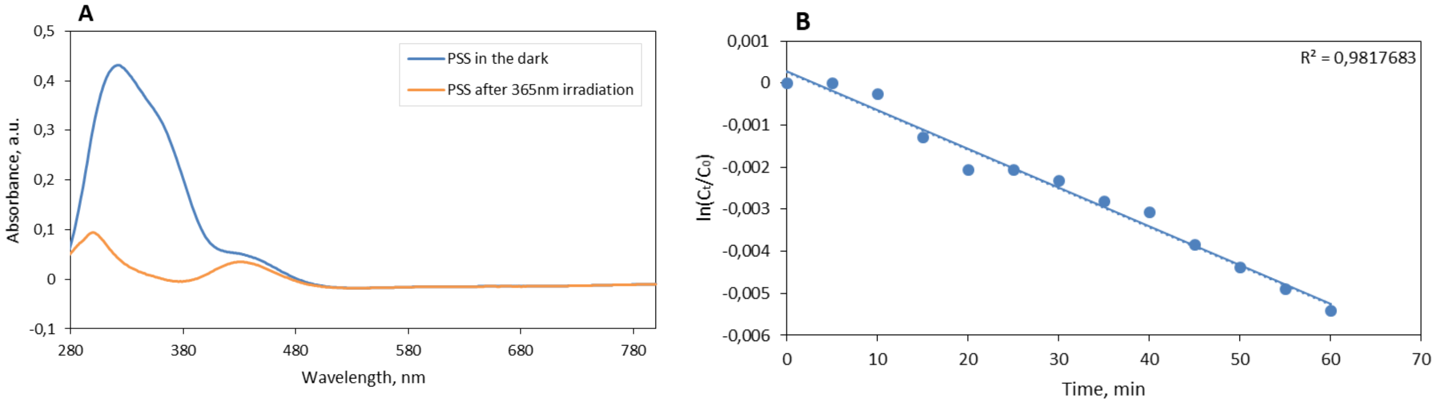

Figure 165. A. UV/Vis absorption spectra of compound **9a** (PLA002) before and after irradiation (365 nm, 5 min) of its aqueous Kolliphor ELP solution. B. Linear fit of the change in absorbance at λ_{max} (323 nm) over time during compound **9a** (PLA002) backward (*Z*-*E*) isomerization in aqueous Kolliphor ELP solution.

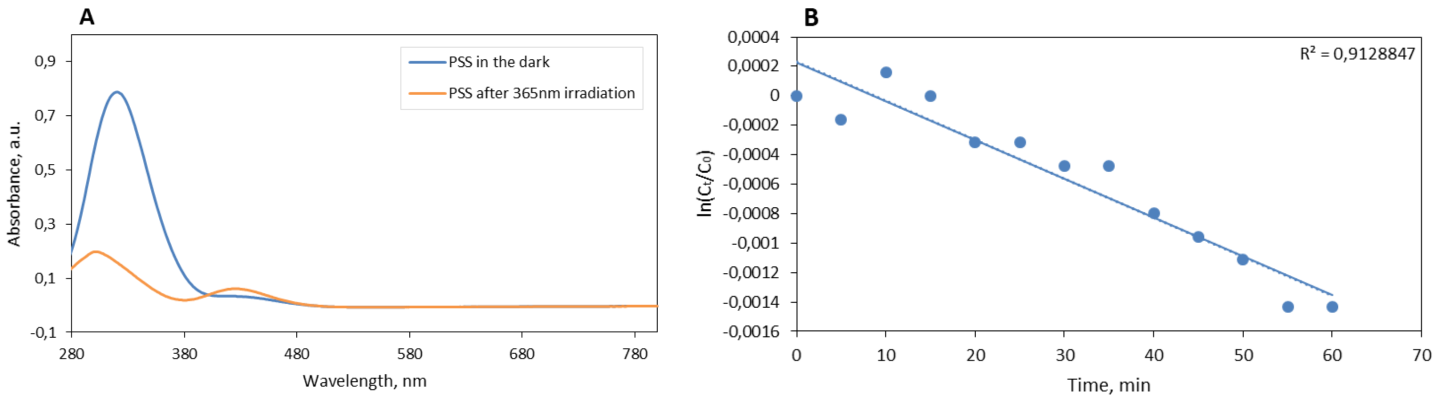

Figure 166. A. UV/Vis absorption spectra of compound **9b** (PLA003) before and after irradiation (365 nm, 5 min) of its aqueous Kolliphor ELP solution. B. Linear fit of the change in absorbance at λ_{max} (320 nm) over time during compound **9b** (PLA003) backward (*Z*-*E*) isomerization in aqueous Kolliphor ELP solution.

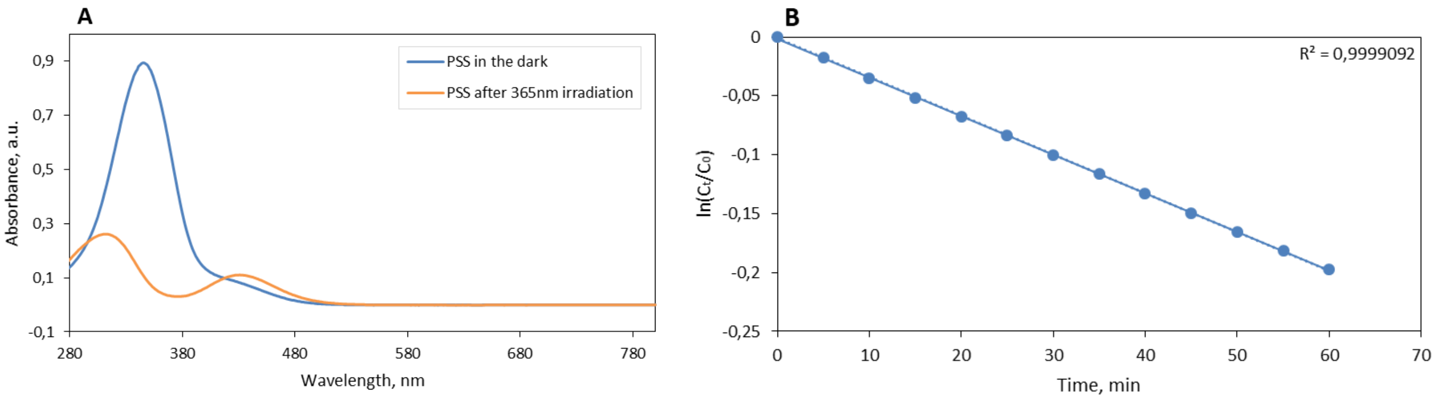

Figure 167. A. UV/Vis absorption spectra of compound **14** (PLA006) before and after irradiation (365 nm, 5 min) of its solution in water. B. Linear fit of the change in absorbance at λ_{max} (345 nm) over time during compound **14** (PLA006) backward (*Z*-*E*) isomerization in water.

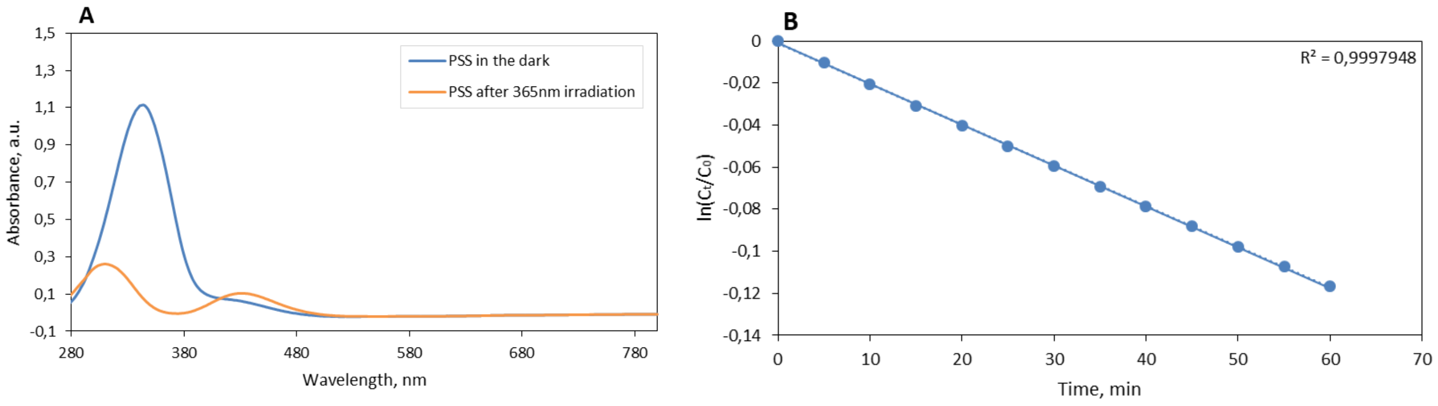

Figure 168. A. UV/Vis absorption spectra of compound **16** (PLA005) before and after irradiation (365 nm, 5 min) of its aqueous Kolliphor ELP solution. B. Linear fit of the change in absorbance at λ_{max} (344 nm) over time during compound **16** (PLA005) backward (*Z*-*E*) isomerization in aqueous Kolliphor ELP solution.

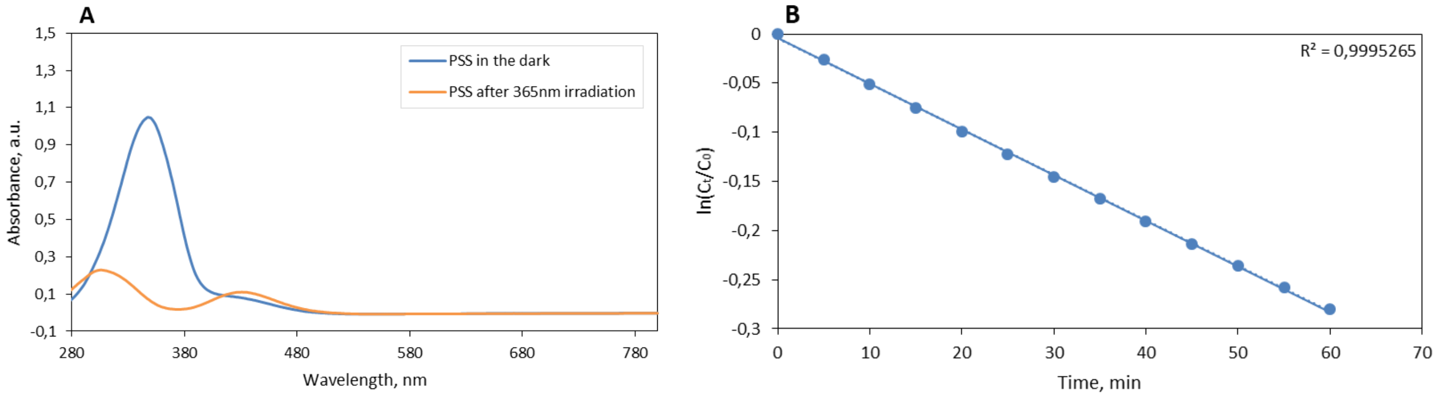

Figure 169. A. UV/Vis absorption spectra of compound **20a** (PLA206) before and after irradiation (365 nm, 5 min) of its aqueous Kolliphor ELP solution. B. Linear fit of the change in absorbance at λ_{max} (348 nm) over time during compound **20a** (PLA206) backward (*Z*-*E*) isomerization in aqueous Kolliphor ELP solution.

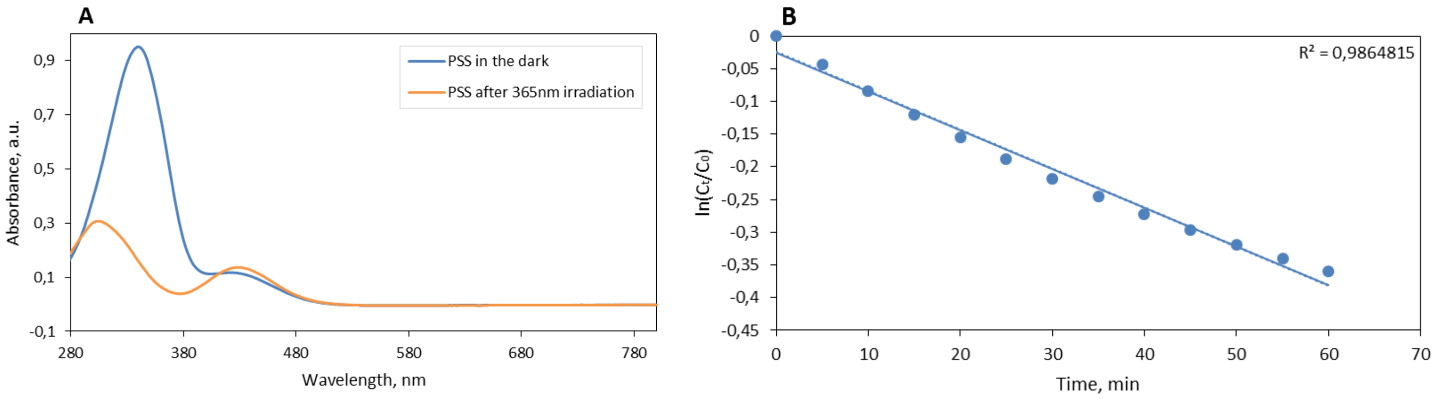

Figure 170. A. UV/Vis absorption spectra of compound **20b** (PLA203) before and after irradiation (365 nm, 5 min) of its aqueous Kolliphor ELP solution. B. Linear fit of the change in absorbance at λ_{max} (339 nm) over time during compound **20b** (PLA203) backward (*Z*-*E*) isomerization in aqueous Kolliphor ELP solution.

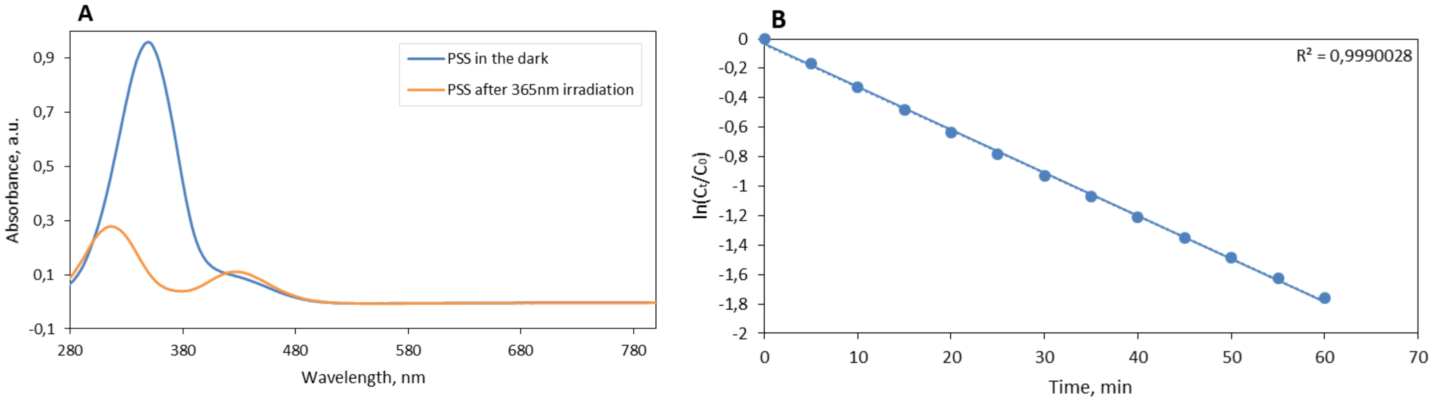

Figure 171. A. UV/Vis absorption spectra of compound **20c** (PLA201) before and after irradiation (365 nm, 5 min) of its aqueous Kolliphor ELP solution. B. Linear fit of the change in absorbance at λ_{max} (349 nm) over time during compound **20c** (PLA201) backward (*Z-E*) isomerization in aqueous Kolliphor ELP solution.

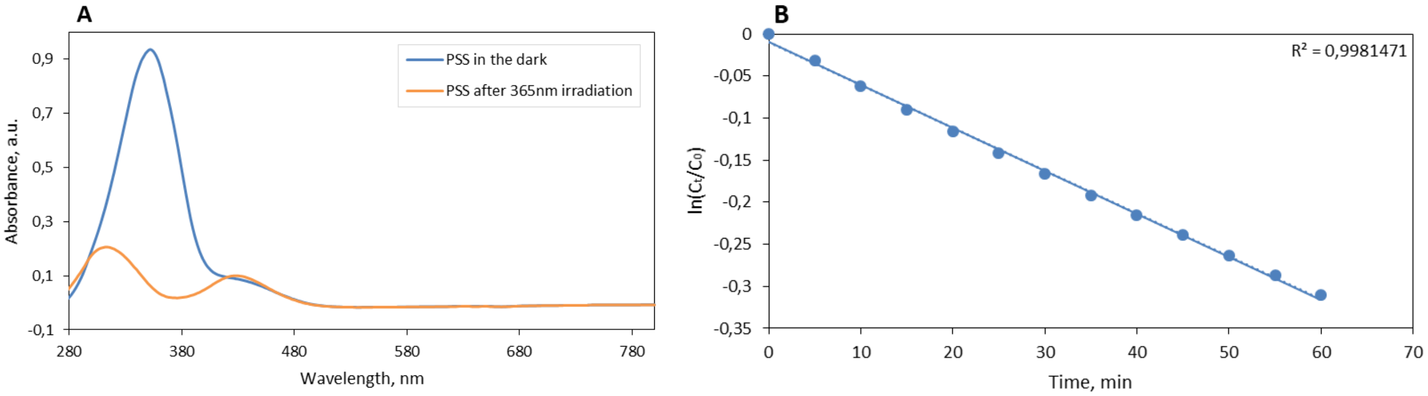

Figure 172. A. UV/Vis absorption spectra of compound **20d** (PLA207) before and after irradiation (365 nm, 5 min) of its aqueous Kolliphor ELP solution. B. Linear fit of the change in absorbance at λ_{max} (352 nm) over time during compound **20d** (PLA207) backward (*Z-E*) isomerization in aqueous Kolliphor ELP solution.

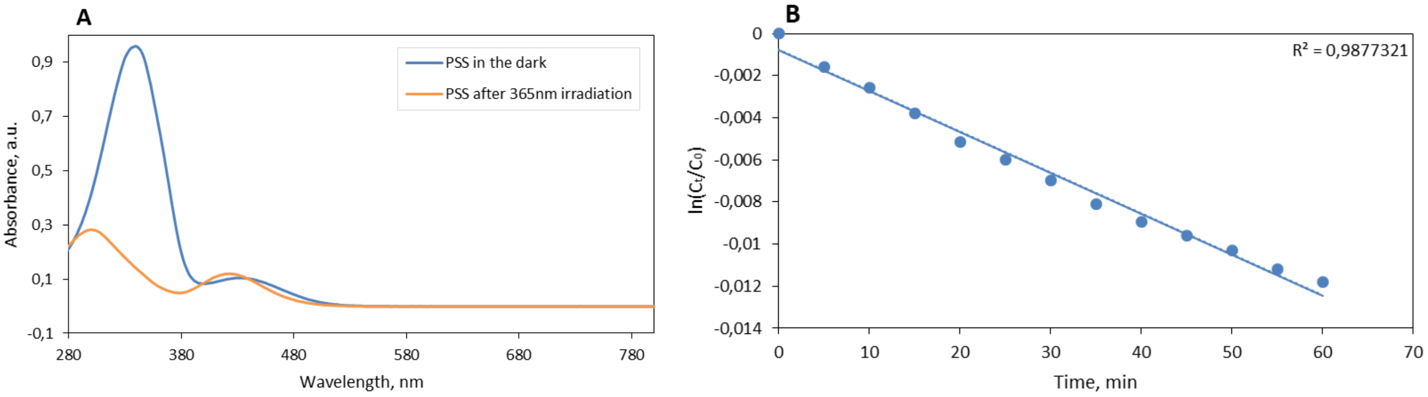

Figure 173. A. UV/Vis absorption spectra of compound **20e** (PLA204) before and after irradiation (365 nm, 5 min) of its aqueous Kolliphor ELP solution. B. Linear fit of the change in absorbance at λ_{max} (340 nm) over time during compound **20e** (PLA204) backward (*Z-E*) isomerization in aqueous Kolliphor ELP solution.

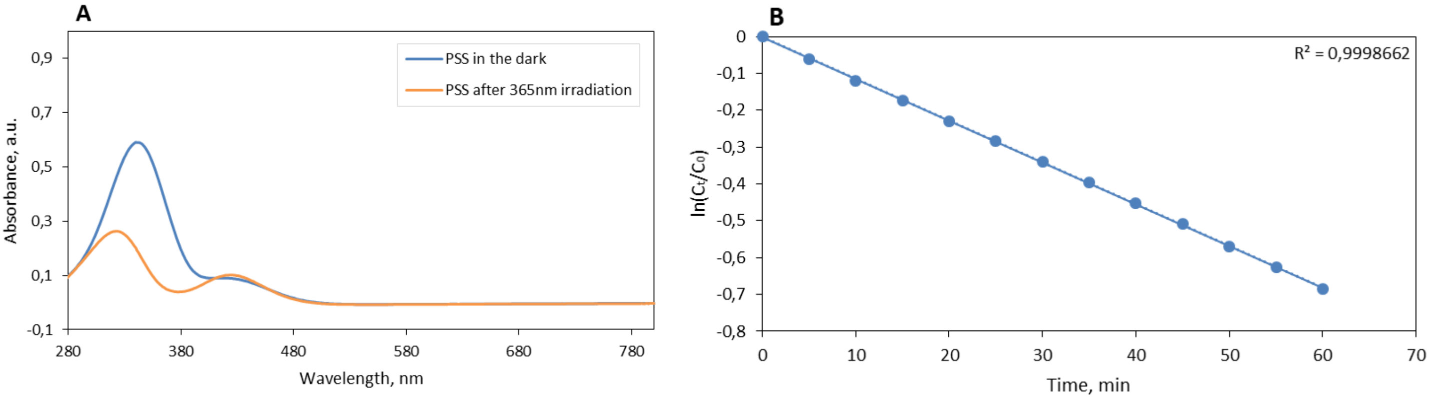

Figure 174. A. UV/Vis absorption spectra of compound **20f** (PLA202) before and after irradiation (365 nm, 5 min) of its aqueous Kolliphor ELP solution. B. Linear fit of the change in absorbance at λ_{max} (341 nm) over time during compound **20f** (PLA202) backward (*Z-E*) isomerization in aqueous Kolliphor ELP solution.

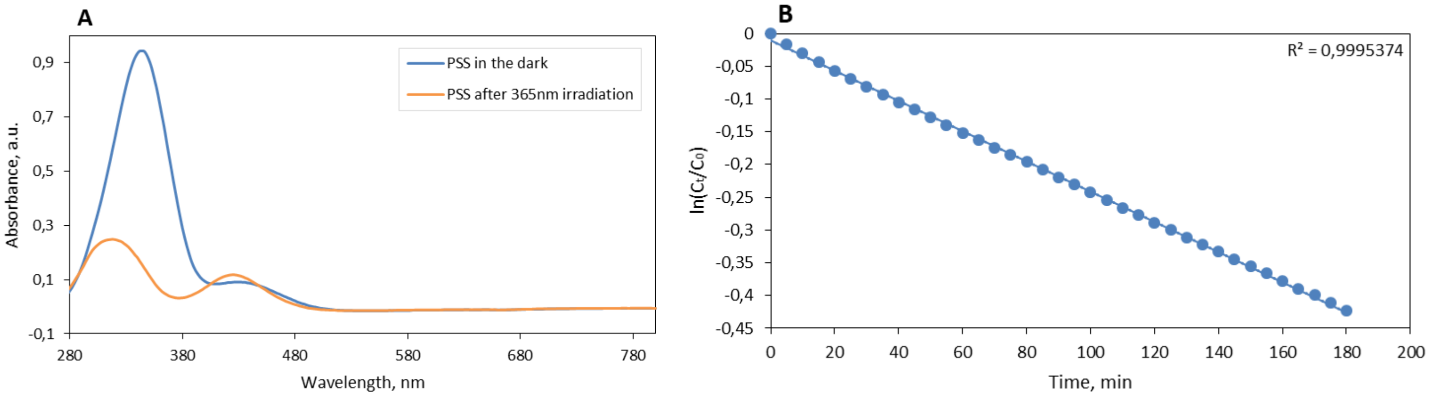

Figure 175. A. UV/Vis absorption spectra of compound **20g** (PLA208) before and after irradiation (365 nm, 5 min) of its aqueous Kolliphor ELP solution. B. Linear fit of the change in absorbance at λ_{max} (344 nm) over time during compound **20g** (PLA208) backward (*Z-E*) isomerization in aqueous Kolliphor ELP solution.

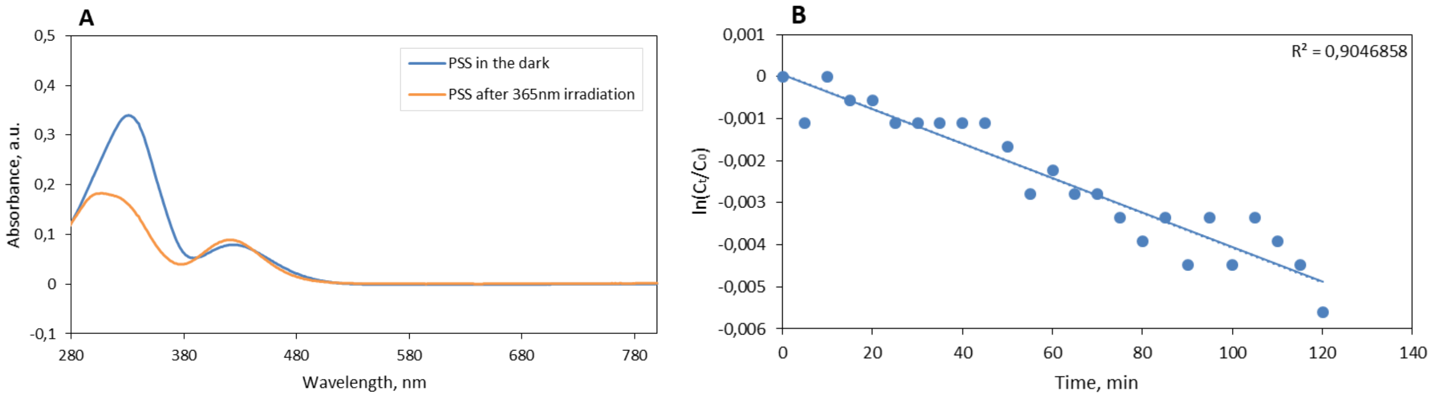

Figure 176. A. UV/Vis absorption spectra of compound **20h** (PLA205) before and after irradiation (365 nm, 5 min) of its aqueous Kolliphor ELP solution. B. Linear fit of the change in absorbance at λ_{max} (331 nm) over time during compound **20h** (PLA205) backward (*Z-E*) isomerization in aqueous Kolliphor ELP solution.

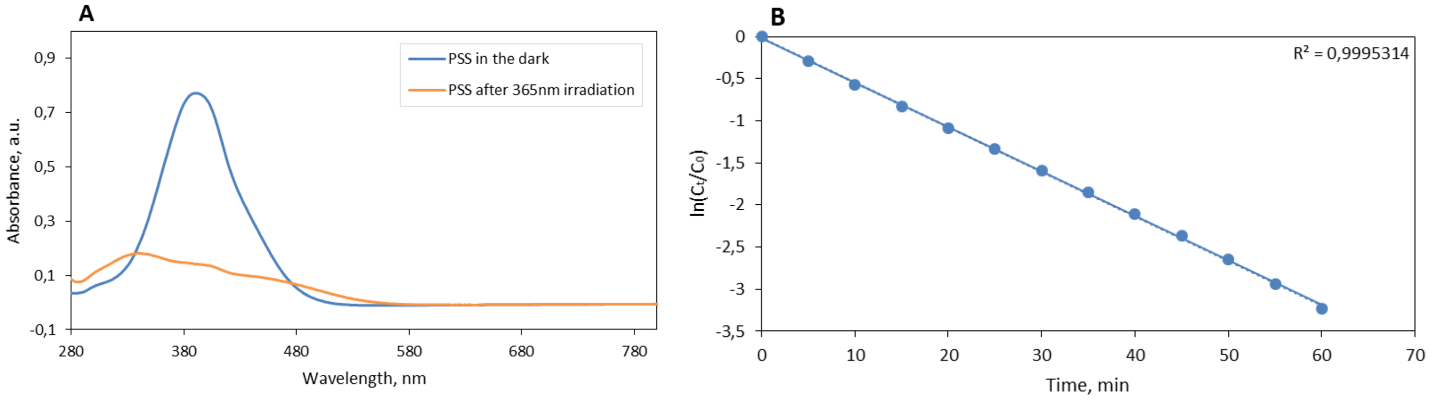

Figure 177. A. UV/Vis absorption spectra of compound **25a** (PLA401) before and after irradiation (365 nm, 5 min) of its aqueous Kolliphor ELP solution. B. Linear fit of the change in absorbance at λ_{max} (391 nm) over time during compound **25a** (PLA401) backward (*Z-E*) isomerization in aqueous Kolliphor ELP solution.

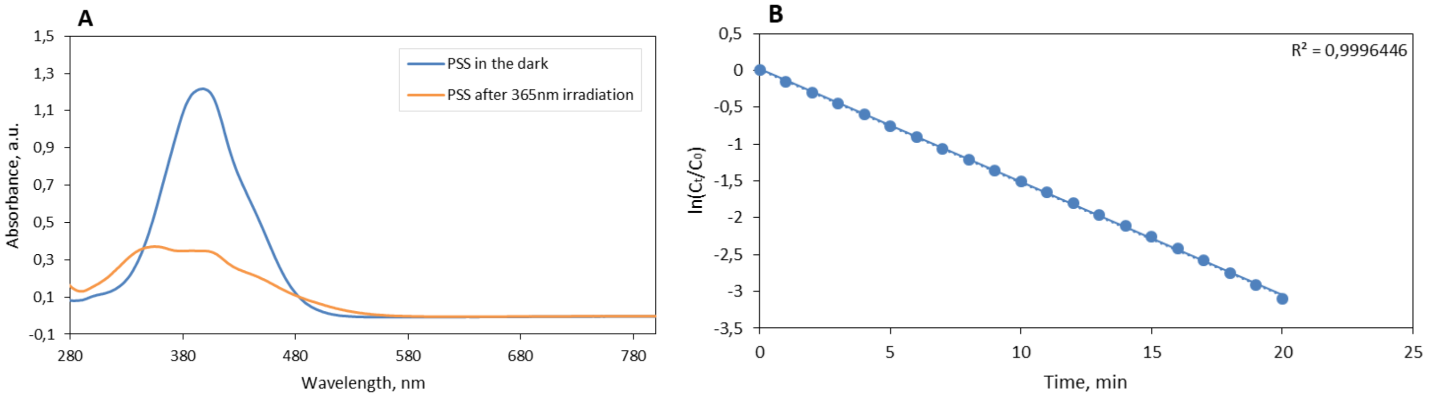

Figure 178. A. UV/Vis absorption spectra of compound **25b** (PLA402) before and after irradiation (365 nm, 5 min) of its aqueous Kolliphor ELP solution. B. Linear fit of the change in absorbance at λ_{max} (398 nm) over time during compound **25b** (PLA402) backward (*Z-E*) isomerization in aqueous Kolliphor ELP solution.
